# Designed, highly expressing, thermostable dengue virus 2 envelope protein dimers elicit quaternary epitope antibodies

**DOI:** 10.1126/sciadv.abg4084

**Published:** 2021-10-15

**Authors:** Stephan T. Kudlacek, Stefan Metz, Devina Thiono, Alexander M. Payne, Thanh T. N. Phan, Shaomin Tian, Lawrence J. Forsberg, Jack Maguire, Ian Seim, Shu Zhang, Ashutosh Tripathy, Joseph Harrison, Nathan I. Nicely, Sandrine Soman, Michael K. McCracken, Gregory D. Gromowski, Richard G. Jarman, Lakshmanane Premkumar, Aravinda M. de Silva, Brian Kuhlman

**Affiliations:** 1 Department of Biochemistry and Biophysics, University of North Carolina, Chapel Hill, NC 27514, USA.; 2 Department of Microbiology and Immunology, University of North Carolina, Chapel Hill, NC 27514, USA.; 3 Department of Pharmacology, University of North Carolina, Chapel Hill, NC 27514, USA.; 4 Curriculum in Bioinformatics and Computational Biology, University of North Carolina, Chapel Hill, NC 27514, USA.; 5 Department of Biology, University of North Carolina, Chapel Hill, NC 27514, USA.; 6 Department of Applied Physical Sciences, University of North Carolina, Chapel Hill, NC 27514, USA.; 7 Viral Diseases Branch, Walter Reed Army Institute of Research, Silver Spring, MD 20910, USA.; 8 Lineberger Comprehensive Cancer Center, University of North Carolina, Chapel Hill, NC 27514, USA.

## Abstract

Dengue virus (DENV) is a worldwide health burden, and a safe vaccine is needed. Neutralizing antibodies bind to quaternary epitopes on DENV envelope (E) protein homodimers. However, recombinantly expressed soluble E proteins are monomers under vaccination conditions and do not present these quaternary epitopes, partly explaining their limited success as vaccine antigens. Using molecular modeling, we found DENV2 E protein mutations that induce dimerization at low concentrations (<100 pM) and enhance production yield by more than 50-fold. Cross-dimer epitope antibodies bind to the stabilized dimers, and a crystal structure resembles the wild-type (WT) E protein bound to a dimer epitope antibody. Mice immunized with the stabilized dimers developed antibodies that bind to E dimers and not monomers and elicited higher levels of DENV2-neutralizing antibodies compared to mice immunized with WT E antigen. Our findings demonstrate the feasibility of using structure-based design to produce subunit vaccines for dengue and other flaviviruses.

## Teaser

A stabilized dimer of the surface protein from dengue virus has been engineered to elicit antibodies that neutralize the virus.

## INTRODUCTION

Despite decades of research and multiple clinical trials, complete vaccine control of dengue virus (DENV), a member of the flavivirus family, has remained elusive (*1*, *2*). About 25% of the ~400 million annual DENV infections worldwide result in dengue fever or dengue hemorrhagic fever, which could be avoided with effective vaccine control of DENV (*2*, *3*). Dengue is caused by four closely related but antigenically distinct virus serotypes. Individuals undergoing a primary infection with one of the DENV serotypes develop serotype-specific protective immunity but remain susceptible to secondary infections with one of the other serotypes (*2*). As DENV serotypes cocirculate in most tropical regions of the world, secondary infections are common (*3*, *4*). These secondary DENV infections increase the risk of developing severe and potentially fatal disease symptoms, such as dengue hemorrhagic fever and shock syndromes (*2*, *5*).

A supported hypothesis for the cause of these severe dengue clinical manifestations is through increased viral burden associated with antibody-dependent enhancement (ADE). Non-neutralizing, serotype cross-reactive antibodies elicited from a primary DENV infection can bind to the secondary DENV serotype and allow for enhanced infection through Fc receptor–mediated endocytosis of the virus (*2*, *5*, *6*). As immunity to only one serotype has the potential to enhance infections caused by other serotypes, leading DENV vaccines are based on tetravalent live-attenuated virus formulations to induce protective responses to all four serotypes. However, these vaccines have notable safety and efficacy concerns, in part, because of uneven replication efficiency of each viral vaccine component. The unbalanced replication causes variable vaccine efficacy between serotypes and, therefore, priming immune responses that increase the severity of wild-type (WT) DENV infections (*7*, *8*). These observations highlight the need for alternative vaccination strategies to protect all groups at risk of infection.

**Fig. 1.**
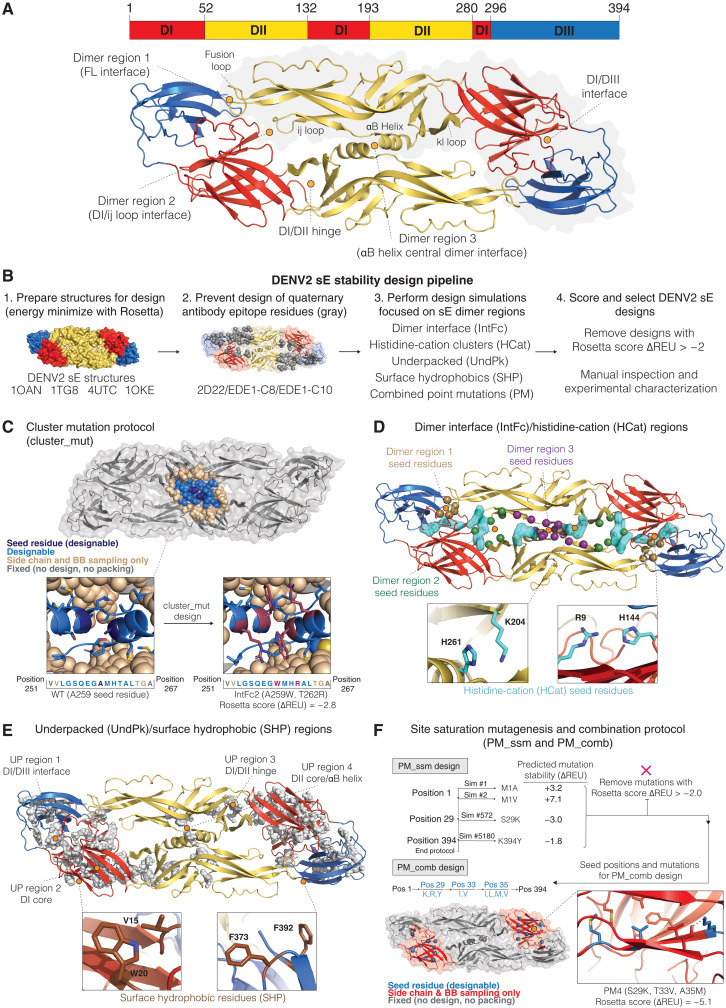
DENV2 sE dimer- and monomer-stabilizing mutations identified using Rosetta computational design. (**A**) Sequence and structural organization of the DENV2 sE dimer (PDB 1OAN). (**B**) Overview of the computational design pipeline. (**C**) The cluster mutation protocol was used to identify small sets of stabilizing mutations adjacent in three-dimensional space. Each simulation centered on a seed residue (dark blue) that, along with residues within 7 Å (light blue), was allowed to mutate. Residues out to 10 Å from the seed residue (tan) were allowed to adopt alternative conformations to accommodate the mutations. (**D** and **E**) Seed residues for the cluster mutation protocol were chosen from four regions in the protein: residues at the dimer interface (IntFc), residues forming histidine-cation interactions (HCat), underpacked regions (UndPk), and surface-exposed hydrophobic amino acids (SHP). The underpacked regions [shown as gray spheres in (E)] were identified using the RosettaHoles protocol (*30*). (**F**) In addition to the cluster mutation protocol, in silico site-saturation mutagenesis was used to scan through the protein to identify favorable point mutations. Point mutations predicted to be favorable for different regions of the protein (entire domains, domain interfaces, or the dimer interface) were then tested in combination using the PM_Comb protocol. BB, backbone.

The DENV lipid envelope contains 180 copies of the envelope glycoprotein (E), which covers the surface of the virus particle (*9*, *10*). On the mature infectious virus particle, E is arranged in a herringbone pattern as antiparallel homodimers that pack laterally into a lipid raft containing three dimers (*9*, *10*). The E protein monomer contains three predominantly β sheet domains (DI, DII, and DIII) connected to a transmembrane domain by an amphipathic helix (Fig. 1A) (*9*). As DENV enters the cell, the E protein undergoes a pH-induced conformational change from a dimeric to a trimeric form that triggers fusion of viral and endosomal membranes required for releasing the viral genome into the cytoplasm of the cell (*9*–*12*). This critical conformational change provides selective pressure during viral evolution that favors E protein sequences that are adept at transitioning between different structures and are not overly stable in a single conformation such as the prefusion dimer (*10*).

The DENV E protein is the primary target of neutralizing antibodies, which has led to a large body of work to assess the immunogenicity and efficacy of this protein as a subunit vaccine (*10*, *13*, *14*). Recent studies have shown that human antibodies that target quaternary epitopes that span across both chains of the E dimer are potently neutralizing and provide protection against DENV infection (*15*–*21*). However, E protein–based subunit vaccines have had limited success in clinical trials (*14*). The poor performance of DENV E protein–based subunit vaccines may be partially attributed to not presenting the important quaternary epitopes responsible for eliciting neutralizing and protective antibodies. Recombinantly expressed soluble DENV2 E proteins (sE) exhibit a dynamic equilibrium between monomer and dimer with a dissociation constant (*K*_d_) of 12 μM at 37°C (*9*, *14*, *22*–*24*) and, thus, are predominately in the monomeric state at the typical concentrations used for vaccination.

Here, we use molecular modeling to design variants of DENV2 sE that are dimeric at physiological conditions and present quaternary epitopes that are recognized by human antibodies that potently neutralize DENV2. Our work makes use of new methods developed for computational protein design in the software package Rosetta (*25*). Similar strategies have been used recently to stabilize and present selected epitopes from respiratory syncytial virus (RSV) and HIV (*26*–*29*). Our work demonstrates how structure-based design can be used to focus the immune response toward critical features on the surface of pathogenic flaviviruses.

## RESULTS

### Molecular modeling simulations to design DENV2 sE–stabilized dimers

To stabilize the DENV2 sE dimer, we used the molecular modeling program Rosetta to identify amino acid mutations that either strengthen contacts across the dimer interface or stabilize the monomer in the conformation that it adopts when forming a dimer (Fig. 1B). Starting from crystal structures of the DENV2 sE dimer, a large set of Rosetta FastDesign (*25*) simulations (>7000 per structure, ~28,000 total) was performed in which each simulation focused on optimizing a different region of the protein or dimer interface. The goal was to identify small sets of localized mutations (one to four mutations) that could be tested experimentally and then later combined to create larger gains in stability. The design protocol (cluster_mut) incorporated a design sphere in which all residues within 7 Å of a seed residue (including the seed residue) were allowed to mutate to any amino acid except cysteine (Fig. 1C). Surrounding the design sphere was a 3-Å layer in which side chains could adopt new conformations during the simulation, but the amino acid identities were fixed. Outside of the two spheres, the side chains were fixed. In all design runs, mutations were not allowed at residues known to interact with potently neutralizing antibodies (Fig. 1B).

To focus the cluster_mut design simulations, we identified seed residues in regions within the DENV2 sE protein that we hypothesized to contribute to protein dimer and/or monomer instability (Fig. 1, D and E). Four sets of simulations were performed: The “IntFc” simulations incorporated seed residues directly at the dimer interface, the “UndPk” simulations focused on buried regions in the protein identified as being underpacked, the “HisCat” simulations replaced interactions between histidines and cationic amino acids (arginine and lysine), and the “SHP” design runs focused on removing hydrophobic patches on the surface of the protein. Underpacked regions were identified using the RosettaHoles protocol (*30*), which detects buried regions in a protein where amino acids do not closely interact. Large underpacked regions were identified in the highly flexible “hinge” regions that connect DI with DII and DIII. During the viral life cycle, the E protein adopts several alternative structures that involve large conformational changes in the hinges. Thus, it is likely that these regions of the protein are not optimized to maintain a dimer conformation. We also focused our simulations on histidine-cation interactions as these residues are “pH-sensing” residues that mediate the E conformational change from prefusion dimer to postfusion trimer but are unlikely to be optimal for high dimer stability (*9*, *31*).

We also used Rosetta to scan through the protein and calculate the change in protein energy for all possible point mutations (Fig. 1F). The most favorable point mutations were combined for further computational testing (“PM_comb”), and favorable combinations were selected for experimental testing.

After performing the various design simulations, from the pool of designs that had improved Rosetta energy scores compared to WT, eight designs were selected from each of the protocols: PM_Comb (PM), UndPk (underpacked), HCat (histidine-cation) and IntFc (interface), and two designs from the SHP (surface hydrophobic to polar) simulations for experimental studies. Recent studies have also demonstrated that a covalently cross-linked sE protein dimer can be formed by introducing disulfides across the dimer interface (*32*, *33*). We included two of these variants, Cm1 (A259C) and Cm2 (L107C and A313C), in our studies. We also selected two mutants (Mnmer1 and Mnmer2) from the point mutant scan that were predicted to destabilize the dimer with the goal of creating sE protein that would remain a monomer even at higher concentrations and low temperature for use as controls in our experiments.

### Mammalian surface display screening of sE variants for quaternary antibody epitope presentation

**Fig. 2.**
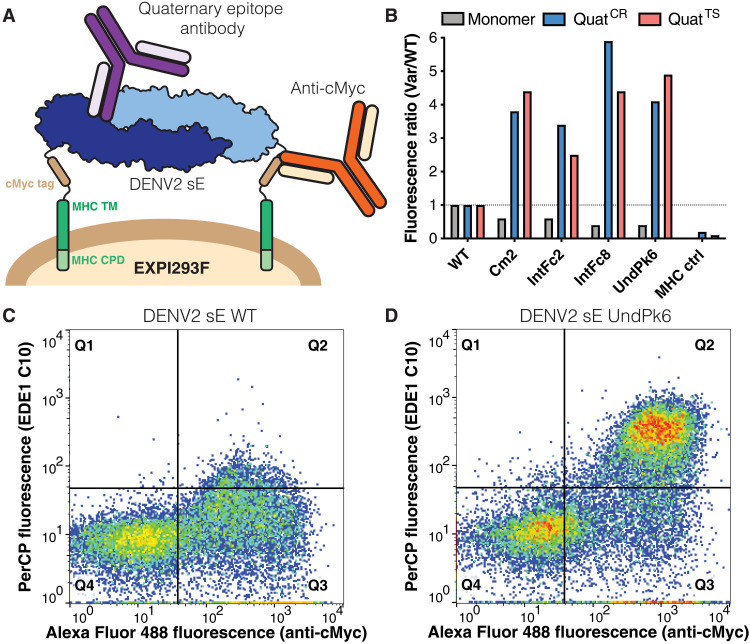
Screening of DENV2 sE designs for binding to quaternary epitope antibodies with mammalian cell surface display. **A**) DENV2 sE (blue/light blue) proteins were displayed on the surface of EXPI293F cells by genetically fusing their C termini to the MHC Iα cytoplasmic and transmembrane (TM) domains (green) via a glycine-serine (GS) linker. A cMyc epitope tag (brown) was inserted for detection of successful surface display by anti-cMyc antibody binding (orange). Quaternary epitope presentation by the DENV2 sE variants was assessed by testing for binding of representative quaternary epitope antibodies (purple). CPD, cytoplasmic domain. (**B**) Flow cytometric analysis of monomer epitope antibody 3H5 (gray) and cross-reactive EDE1 C10 (Quat^CR^; blue) and type-specific 2D22 (Quat^TS^; red) quaternary epitope antibody binding to surface-displayed DENV2 sE WT, DENV2 sE variants (Cm2, IntFc2, IntFc8, and UndPk6), and a membrane anchor–negative control (MHC ctrl). The geometric mean fluorescence ratios of sE variant to sE WT surface displayed cells after antibody immunostaining are plotted. (**C** and **D**) Flow cytometry data for cells displaying DENV2 sE WT (C) or DENV2 sE UndPk6 (D) at 40°C labeled with an anti-cMyc antibody (*x* axis) and the EDE1 C10 quaternary epitope antibody (*y* axis).

To screen for sE variants that form a dimer at temperatures relevant to vaccination, we expressed the Rosetta-designed variants on the surface of EXPI293F cells and used flow cytometry to measure binding between the variants and antibodies that target quaternary epitopes on DENV2. To enable cell surface display, we fused the transmembrane and cytoplasmic domains of the major histocompatibility complex I (MHC-I) α chain to the C terminus of the E protein ectodomain (residues 1 to 394) and used an N-terminal human serum albumin signal peptide to direct the protein to the cell surface (*32*). A cMyc tag was also included in the linker between E protein and the MHC-I transmembrane domain to measure the amount of protein expressed on the cell surface. Epitope presentation was assessed by measuring the binding of DENV2 type-specific (TS) antibody (2D22) and cross-reactive (CR) neutralizing quaternary epitope antibodies (EDE1 C8 or EDE1 C10) to cells displaying membrane-anchored sE protein at 23° or 40°C [peridinin-chlorophyll-protein (PerCP)–positive fluorescence; Fig. 2 and fig. S1]. We also probed for the binding of single-domain epitope antibodies including 3H5 (DIII) and 1C19 (DII/bc loop) as a readout for proper folding of the variants (Fig. 2B and fig. S1). At 23°C, most of the design variants and WT sE were efficiently recognized by dimer-specific monoclonal antibodies 2D22 and EDE1 C8, indicating that, at 23°C, most variants were displayed as properly assembled dimers (fig. S1). Next, we analyzed the integrity of quaternary epitopes at 40°C. In contrast to the 23°C screen, 2D22 and EDE1 C10 bound poorly to WT sE (Fig. 2C and fig. S1). Of 34 design variants tested, 23 bound more 2D22 and EDE1 C10 than WT sE, and 8 design variants (HCat3, IntFc2, IntFc8, UndPk6, PM2, PM4, SHP1, and SHP2) had a threefold or higher binding, suggesting that these variants were forming more stable dimers compared to WT sE at >37°C (Fig. 2, B and D, and fig. S1).

### Design variants increase DENV2 sE dimer stability and expression yields

**Fig. 3.**
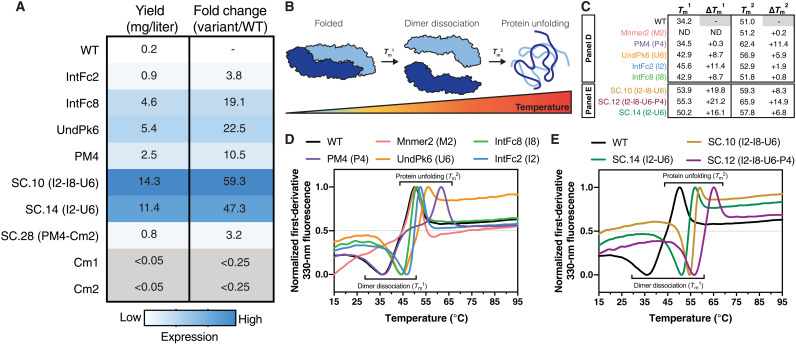
DENV2 sE designs have higher production yields and thermostability. (**A**) Protein yields following expression in EXPI293F cells and purification with metal affinity chromatography. (**B**) Two transitions were detected when monitoring the thermal unfolding of DENV2 sE with nanoscale differential scanning fluorimetry (nanoDSF), dissociation from dimer to monomer (*T*_m_^1^), followed by complete protein unfolding and aggregation (*T*_m_^2^) (*24*). (**C**) Melting temperatures for selected DENV2 sE variants. (**D** and **E**) DENV2 sE protein nanoDSF thermal melt experiments for Rosetta designs and combination variants (protein concentrations represented are 8 μM). ND, not detected.

On the basis of the mammalian cell surface antibody epitope display results, we selected 22 design variants, Mnmer2, and Cm1 and Cm2 for further characterization as soluble secreted proteins. The sE variants were expressed in EXPI293F cells and purified using His-tag affinity chromatography (fig. S2). Sixteen of the 22 Rosetta design variants had expression yield levels comparable to or greater than WT sE (Fig. 3A and table S1). Three variants in particular showed a large increase in expression yield, PM4 (10-fold), IntFc8 (19.1-fold), and UndPk6 (22.5-fold). In general, the Rosetta variants that had increased quaternary antibody epitope display at 40°C on the mammalian cell surface also had increased expression yields as a soluble protein. The disulfide variants, Cm1 and Cm2, expressed at much lower levels than WT sE.

Next, we measured dimer *K*_d_s and thermal unfolding temperatures of the sE variants using nanoscale differential scanning fluorimetry (nanoDSF). In a previous study, we observed two transitions in the DENV2 sE WT thermal unfolding curve. Using various methods, we showed that the midpoint of the first transition, which we refer to as *T*_m_^1^, reports on the dissociation of the sE dimer, and the second transition, *T*_m_^2^, corresponds to unfolding of the sE monomer (Fig. 3B). *T*_m_^1^ is dependent on the concentration of the protein, and by performing the nanoDSF experiment at various concentrations, it is possible to determine the *K*_d_ for the monomer-dimer equilibrium at 37°C. For DENV2 sE WT at 4 μM, *T*_m_^1^ is 34.2°C and *T*_m_^2^ is 51.0°C (Fig. 3, C and D, and table S1) (*24*). Aside from Mnmer2 and the disulfide variants, *T*_m_^1^ and *T*_m_^2^, transitions were observed for all Rosetta variants (table S1). No *T*_m_^1^ transitions were observed for Mnmer2 and the disulfide dimers, consistent with them being constitutively monomeric or covalently linked dimers, respectively.

Four design variants—IntFc2, IntFc8, UndPk6, and PM4—produced particularly large increases in *T*_m_^1^ and/or *T*_m_^2^ (Fig. 3, C and D, and table S1). IntFc2 (A259W/T262R) creates two new pi-cation interactions across the underside (i.e., the side of the dimer facing the membrane) of the αB helix central dimer interface and raised *T*_m_^1^ by 10.5°C. IntFc8 (G106D) is located in the fusion loop (FL) and introduces new electrostatic interactions at the interchain interface between DII and DIII and raises *T*_m_^1^ by 8.1°C. The variant UndPk6 (F279W/T280P) is particularly interesting as it stabilizes the dimer and monomer simultaneously (+8.7°C *T*_m_^1^ and +5.9°C *T*_m_^2^) (table S1). These mutations are located in the hinge between DI and DII and are predicted to stabilize the hinge in the conformation the E protein adopts when forming the dimer. As prolines are particularly effective at locking protein loops in specific conformations, we tested the point mutation T280P (UndPk6.1) alone and observed increases in *T*_m_^1^ of 6.4°C and *T*_m_^2^ of 2.6°C, indicating that T280P accounts for much of the increased dimer stability from UndPk6 (table S1). Last, the PM4 mutations (S29K, T33V, and A35M) are located in the core of domain I and do not interact at the dimer interface or contribute to interactions in the E protein hinges. Consistent with this observation, the PM4 mutations markedly raised *T*_m_^2^ by +11.4°C but had no effect on *T*_m_^1^ (Fig. 3, C and D, and table S1). This result indicates that domain I is a key factor in the low overall thermostability of the sE monomer.

**Fig. 4.**
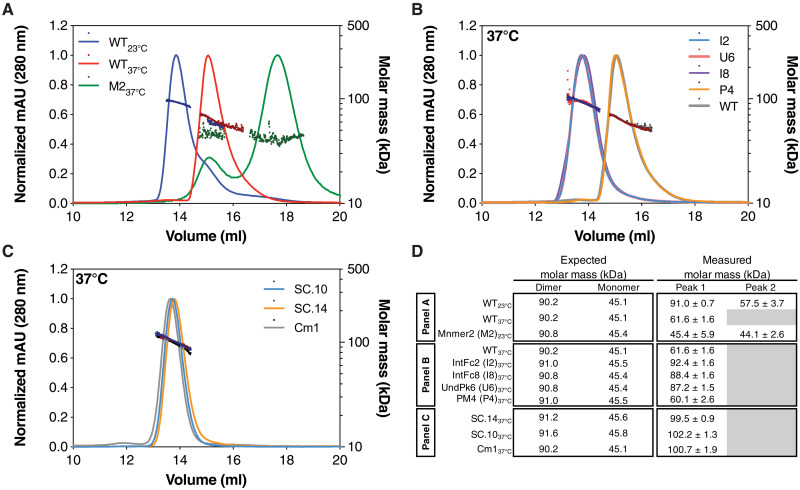
SEC-MALS confirms that DENV2 sE designs form dimers at 37°C. (**A** to **C**) SEC-MALS experiments were performed at room temperature and 37°C. Protein concentrations were between 30 and 55 μM. The molar mass as measured by light scattering is shown by the dots (values indicated by the right *y* axis of each plot). Abbreviations used for the variants in (A) and (B) are the following: WT, M2 (Mnmer2), I2 (IntFc2), I8 (IntFc8), U6 (UndPk6), and P4 (PM4). (**D**) A comparison of the expected and measured molar masses for the dimeric and monomeric forms of the sE variants. The expected molar mass does not account for glycosylation. While the WT protein has a measured mass at 37°C (61.6 kDa) that indicates that it is in equilibrium between a monomer and dimer, the combination designs, SC.10 and SC.14, and the disulfide dimer, CM1, have measured masses consistent with the formation of a stable dimer. mAU, milli absorbance units.

We measured the homodimer affinity of the most stable dimer variants by performing nanoDSF at protein concentrations ranging from 2 to 16 μM, allowing us to use a van’t Hoff analysis to estimate the equilibrium *K*_d_ at 37°C (*24*). For all variants and WT sE, we observed increasing *T*_m_^1^ values with increasing protein concentrations. WT DENV2 sE had a *K*_d_ of 12.9 μM, and IntFc2 had a *K*_d_ of 23 nM at 37°C, demonstrating a >500-fold increase in the variant’s dimer affinity over WT (table S1). Similarly, for IntFc8 and UndPk6, we measured *K*_d_ values of 219 nM (~17-fold increase) and 238 nM (~18.5-fold increase), respectively (table S1). To further validate the design variants that were forming dimers at 37°C, we used size exclusion chromatography coupled with multiangle light scattering (SEC-MALS) to measure the oligomeric state of these variants at 37°C. Unlike the sE WT that elutes predominantly as a monomer at 37°C (Fig. 4A), IntFc2, IntFc8, and UndPk6 all had MALS-measured molecular masses (MMs) consistent with dimer formation (Fig. 4B). Mnmer2 eluted as two peaks, both with measured MM nearly identical to the theoretical monomer weight, indicating that the protein is a constitutive monomer, and it may be adopting two alternative conformations (*33*). A similar SEC-MALS profile has been previously observed for WT DENV2 sE.

### Combining stabilizing mutations further increases DENV2 sE dimer stability and expression yields

To determine whether combinations of Rosetta-designed mutations can raise dimer stability even further, we tested 28 stable combination (SC) variants built from our most stabilizing designs: IntFc2, IntFc8, UndPk6, PM4, and HCat3. The SC variants were expressed, purified, and tested for dimer and monomer stability using nanoDSF thermal melts. All SC variants had improved expression yields, with some increasing yield >50-fold (variant) compared to WT (table S1). In general, increases in *T*_m_^1^ and *T*_m_^2^ were additive, and many of the SC variants exhibited large increases in *T*_m_^1^ and *T*_m_^2^. Variant SC.14 containing just four mutations from two of the best stabilizing dimer mutation sets, IntFc2 and UndPk6, had a *T*_m_^1^ of 50.2°C (+16.1°C relative to WT) and a *K*_d_ of 12 nM at 37°C (table S1). Variant SC.10, which builds on SC.14 with a single additional mutation G106D (IntFc8), further raised *T*_m_^1^ to 53.9°C and lowered the *K*_d_ to less than 100 pM at 37°C (table S1). The exceptional stability of the SC.10 dimer is likely due to simultaneously stabilizing the central dimer interface (IntFc2), the hinge (UndPk6), and the FL dimer interface (IntFc8), all critical regions that regulate dimer stability. Consistent with the nanoDSF analysis, both SC.14 and SC.10 eluted as a single peak upon analysis with SEC-MALS at 37°C with MM of 99.5 and 102.2 kDa, respectively, similar to the disulfide stabilized sE dimer, Cm1 (Fig. 4, C and D).

### DENV2 sE SC.10 adopts the same dimer conformation as the EDE1 C8 bound sE dimer

**Fig. 5.**
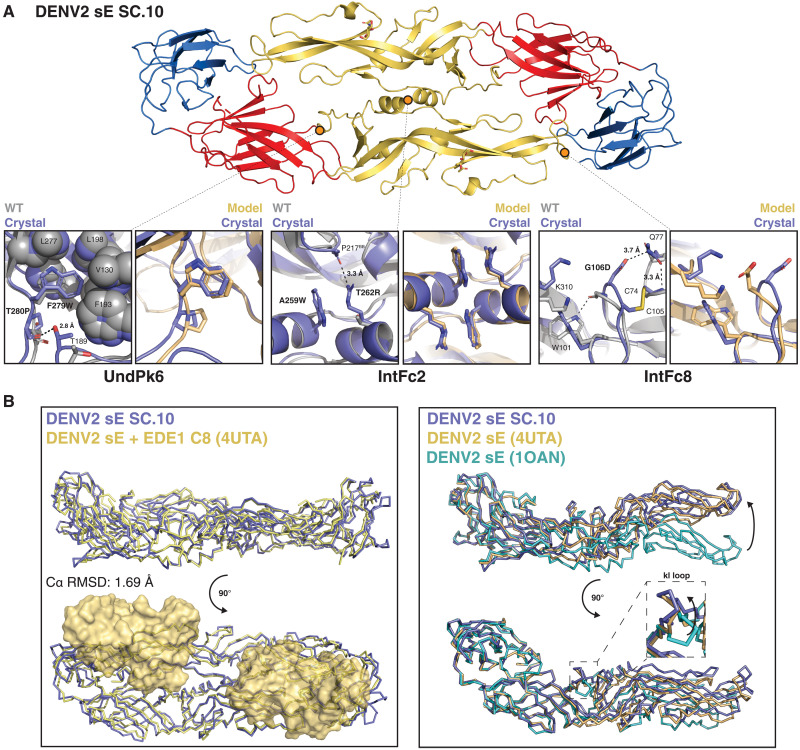
DENV2 sE SC.10 crystal structure comparison to Rosetta model and EDE1 C8/sE cocrystal structure. (**A**) Top: DENV2 sE SC.10 dimer conformation observed in the crystal structure, colored by domain (DI, red; DII, yellow; DII, blue). Bottom: Comparison of UndPk6 (left), IntFc2 (middle), and IntFc8 (right) mutations observed in the SC.10 crystal structure (purple) to DENV2 sE WT crystal structure (gray; PDB 1OAN) and the Rosetta-predicted computational model (yellow). Hydrogen bonds are shown as black dotted lines. (**B**) Left: Structure superposition of the DENV2 sE SC.10 dimer (purple) with the DENV2 sE WT dimer (yellow) cocrystallized with EDE1 C8, a DENV broadly neutralizing quaternary epitope antibody. EDE1 C8 Fabs are shown as a surface representation. Right: Structure superposition of the DI and DIII domains from DENV2 sE WT sE (PDB 1OAN, teal; and 4UTA, yellow) and DENV2 sE SC.10 (purple). Inset shows a closer view of the kl loop conformation observed in each structure after DI and DIII alignment.

To compare SC.10 and WT sE structures and to validate the accuracy of the Rosetta design models, we determined the crystal structure of SC.10 at 3.42 Å using molecular replacement (MR) (table S2). Despite growing the crystal at pH 4.4, SC.10 retained the prefusion DENV2 sE dimer conformation (Fig. 5A). This contrasts with the WT DENV1 sE protein, which crystallized as the postfusion trimer at pH 4.5 (*34*). The fact that SC.10 remains a dimer at low pH is consistent with the increased dimer stability of SC.10. We structurally aligned the SC.10 dimer structure to the previously solved crystal structures of WT DENV2 sE protein. The closest match was to the sE protein cocrystallized with human monoclonal antibody EDE1 C8 [Protein Data Bank (PDB) 4UTA], a DENV broadly neutralizing quaternary epitope antibody (*16*), with a Cα root mean square deviation (RMSD) of 1.7 Å (Fig. 5B). The Cα RMSD with the unliganded DENV2 WT sE protein crystallized at pH 9.0 (PDB 1OAN) is 3.5 Å.

Further comparison of the SC.10 structure with the WT sE crystal structure showed that the small structural differences were due to changes in the DI/DII hinge angle (Fig. 5B). Analysis of multiple DENV sE crystal structures and cryo–electron microscopy (cryo-EM) structures of the mature virion has revealed that the E protein DI/DII hinge angle is variable, consistent with the intrinsic dynamics and multiple conformations that the E protein can adopt (*9*, *12*, *22*, *23*, *35*). In the cryo-EM structures of the virion, the DI/DII hinge bends, allowing the E dimer to match the curvature of the virion, while in previously determined crystal structures of the sE dimer, the dimer crystallizes in a more straight or flat conformation (*35*). The SC.10 crystal structure follows this pattern and has a DI/DII hinge angle that supports a conformation that is flatter than what is observed on the virion. However, these changes do not appear to have a large influence on the binding of potently neutralizing EDE1 human monoclonal antibodies because the WT DENV2 sE protein crystallized with EDE1 antibodies adopts a conformation that is similar to the structure we observed for SC.10 (*16*, *36*).

Clear electron density was observed for all sets of mutations (IntFc2, IntFc8, and UndPk6) in the SC.10 crystal structure (fig. S3A). The two UndPk6 mutations, F279W and T280P, are in the kl loop of the DI/DII hinge. F279W fills an underpacked region in the hinge as predicted (Fig. 5A). T280P is at the base of the kl loop, and in the SC.10 structure, the backbone at residue 280 is shifted to create a new backbone side chain hydrogen bond between the P280 backbone carbonyl and the T189 side chain hydroxyl in an adjacent loop. In addition to the structural rigidity that prolines can provide, the new hydrogen bond may be helping to stabilize the sE dimer (*37*, *38*). As predicted by the Rosetta model, the two mutations in IntFc2, T262R and A259W, lead to canonical pi-cation interactions across the central dimer interface between R262 on one chain and W259 on the other chain (Fig. 5A). IntFc8 contains a single mutation in the FL, G106D, which we predicted to form a new salt bridge with K310 across the dimer interface. Instead, in the SC.10 structure, D106 forms an intradomain hydrogen bond with Q77, and alternative rotamers are observed for K310 and W101 (Fig. 5A). These changes may be a consequence of low pH as in the SC.10 structure the ij loop, and the N termini have conformational changes near the FL that match more closely to the DENV sE structures also solved at low pH (fig. S3B). In addition, the low pH makes protonation of D106 more likely, which could favor hydrogen bonding to Q77 over formation of a salt bridge with K101 (*39*). Thus, from our low pH structure, it is not clear how G106D is enhancing dimer stability.

### DENV2 sE SC.10 and SC.14 bind to quaternary epitope antibodies at 37°C and have reduced affinity for FL antibodies

**Fig. 6.**
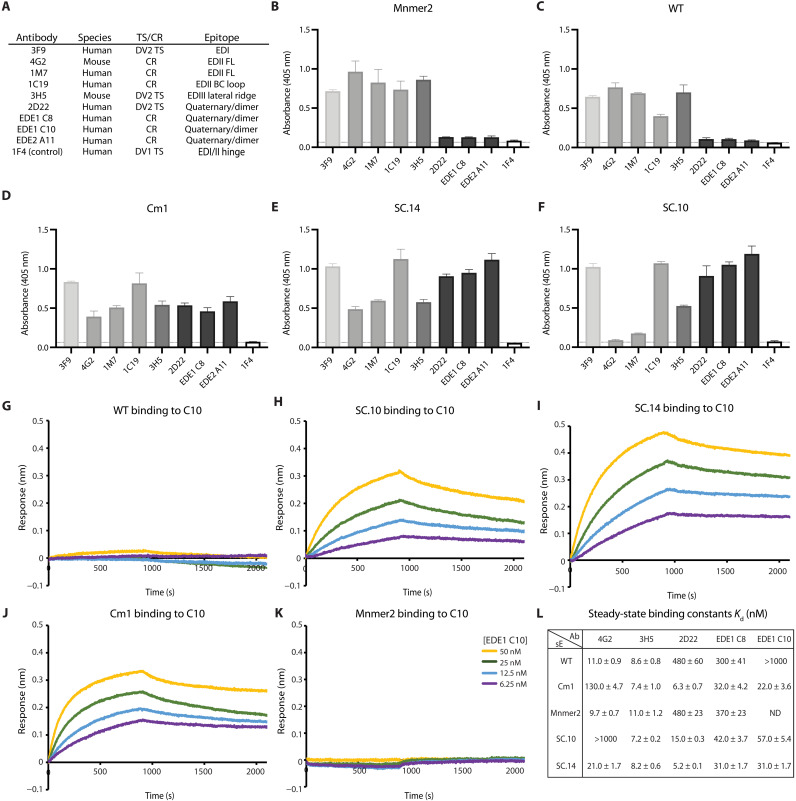
Epitope presentation of DENV2 sE designs assessed by binding of monomer and quaternary epitope antibodies. ELISA antibody binding analysis at 37°C of DENV E monomer and quaternary epitope antibodies (**A**) with the indicated DENV2 sE variants (**B** to **F**). sE protein (100 ng) at 45 nM was immobilized on a nickel-coated ELISA plate and incubated with 100 ng at 0.2 ng/ml of each antibody. The binding signal for the antibodies that bind quaternary epitopes (2D22, EDE1 C8, and EDE2 A11) are shown in black. Binding measurements were performed in triplicate, with error bars representing the mean 405-nm signal ± SD of the mean. BLI sensorgrams showing the binding of EDE1 C10 to DENV2 sE variants at 37°C (**G** to **K**). sE (10 nM) was loaded on Ni-NTA sensors and analyzed across different concentrations of EDE1 C10 (6.25 to 50 nM). Steady-state binding affinities were calculated from BLI binding responses between DENV2 sE variants and different anti-E antibodies (**L**). “ND,” not determined due to the inability to fit. Errors to the fit are indicated for each measurement.

With the crystal structure confirming the dimeric structure of SC.10, we tested the binding of DENV2 antibodies to SC.10 and SC.14. Enzyme-linked immunosorbent assay (ELISA) experiments were performed at 37°C by incubating a panel of DENV2 TS and DENV CR domain, and quaternary epitope–neutralizing antibodies with DENV2 sE variants (Mnmer2, WT, Cm1, SC.14, and SC.10) were immobilized on a nickel-coated ELISA plate (Fig. 6A). The sE variants were loaded onto the plates by incubating 45 nM sE protein with the plate for 1 hour at 37°C. At this E protein concentration and temperature (which is representative of vaccination conditions), the WT sE protein is primarily monomeric, while the stabilized sE variants are dimeric. Binding of DIII-specific (3H5) (*40*), DI-specific (3F9) (*17*), and DII-specific (1C19) (*41*) antibodies was observed for all five DENV2 sE proteins (Fig. 6, B to F). Unlike the domain-specific antibodies, the quaternary epitope antibodies, EDE2 A11, 2D22, and EDE1 C8 (*17*) (*15*, *16*) only bound to Cm1, SC.14, and SC.10 (Fig. 6, D to F). We also tested binding of the FL epitope (FLE) antibodies, 4G2 (*42*) and 1M7 (*41*), to each of the DENV2 sE proteins. The FLE antibodies are poorly neutralizing and cross-reactive with other DENV serotypes, and antibodies of this type have been implicated in ADE during infection (*43*). Both 4G2 and 1M7 bound well to Mnmer2 and WT (Fig. 6, B and C) but had reduced binding to Cm1 and SC.14 (Fig. 6, D and E), consistent with the previous observation that sE dimer stabilization reduces FLE exposure (*33*, *44*). Little or no binding was observed to SC.10 (Fig. 6F). Mutations at G106 are known to eliminate 4G2 binding (SC.10 contains G106D) (*45*), but it has been reported that 1M7 is not sensitive to mutations at G106 (*41*). This indicates that the reduced binding of 1M7 is due to increased stabilization of the dimer.

To further probe the binding properties of the stabilized E dimers, we performed biolayer interferometry (BLI) experiments at 37°C. DENV2 sE variants (Mnmer2, WT, Cm1, SC.14, and SC.10) were immobilized on nickel nitrilotriacetic acid (Ni-NTA) biosensors and tested for binding with a panel of DENV2 antibodies (Fig. 6, G to L, and fig. S4). Consistent with the ELISA experiments, the dimer-specific antibodies (2D22, EDE1 C8, and EDE1 C10) bound more tightly to the stabilized E dimers SC.10, SC.14, and Cm1 (*K*_d_ < 60 nM) than WT E and Mnmer2 (*K*_d_ > 300 nM) (Fig. 6L). As expected, all the E variants bound tightly to the domain III–specific antibody 3H5. As observed in the ELISA experiments, SC.10, which contains the mutation G106D, did not bind to the FLE antibody, 4G2. Together, the ELISA and BLI experiments demonstrate that the stabilized E dimers, SC.10 and SC.14, present quaternary structure epitopes recognized by human antibodies that potently neutralize DENV.

### DENV2 sE SC.14 and SC.10 elicit DENV2 E dimer–specific antibodies in mice

**Fig. 7.**
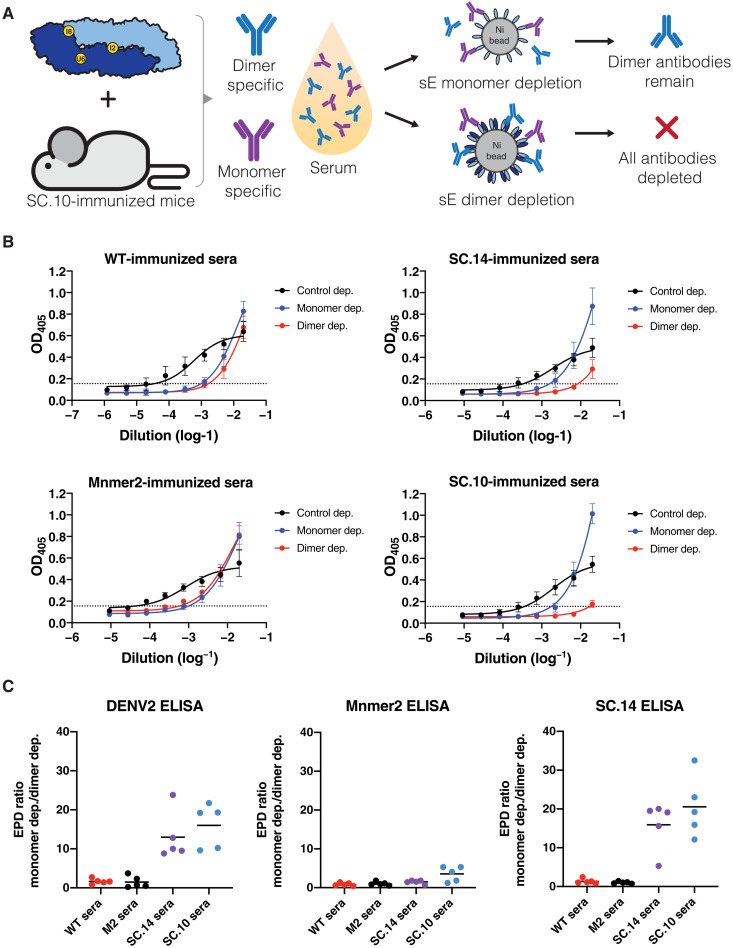
DENV2 sE SC.14 and SC.10 stable dimers elicit DENV2 E dimer–specific antibodies in mice. (**A**) Mice were immunized with DENV2 sE WT, Mnmer2, SC.14, or SC.10 proteins (without adjuvant). To identify whether elicited antibodies target E monomer epitopes (purple) or dimer epitopes (blue), beads coated with sE monomers or dimers were used to deplete antibodies binding to each antigen. Antibodies binding to epitopes present on the monomer should be depleted by either sE monomer- or dimer-conjugated beads. Dimer-specific antibodies should be depleted by sE dimer but not monomer-conjugated beads. (**B**) ELISA to measure DENV2-binding antibodies in mouse immune sera after control depletion (black) or removal of sE monomer (blue)– or dimer (red)–binding antibodies. The graphs depict the absorbance ± SD of the mean for all mice (*n* = 5) in each group. OD_405_, optical density at 405 nm. (**C**) To compare levels of E dimer–specific antibodies in mice immunized with different antigens, sera were depleted of sE monomer- or dimer-binding antibodies and tested by ELISA for DENV2-, sE monomer (Mnmer2)–, and sE dimer (SC.14)–binding antibodies. The graphs depict the ratio of the end point dilution (EPD) titer after depleting monomer-binding antibodies divided by the EPD titer after dimer depletion. A ratio > 1 is indicative of antibodies binding to epitopes displayed on the dimer only.

Next, we tested whether mice immunized with stable dimers developed antibodies to epitopes displayed on dimers but not on monomers. Mice were primed and boosted with WT sE, Mnmer2, SC.14, and SC.10, without the addition of adjuvant to evaluate the impact of antigen structure on antibody specificity. All antigens stimulated DENV2-binding antibodies (fig. S5). We performed antigen-specific antibody depletions to determine whether mice immunized with stabilized dimers developed dimer-specific antibodies. The mouse immune sera were incubated with magnetic beads coated with monomers (Mnmer2) or dimers (SC.14) to deplete specific antibody populations and then tested for binding to monomers, dimers, and DENV2 virions (Fig. 7A). Mice immunized with WT sE or Mnmer2 developed antibodies that were equally effectively depleted using monomers or dimers (Fig. 7, B and C). This result indicates that animals immunized with these antigens mainly develop antibodies that target single-domain epitopes that are present on both monomers and dimers. Animals immunized with stable dimers (SC.14 and SC.10) developed DENV2-binding antibodies that were efficiently depleted with the dimer (Fig. 7, B and C). Dimer-immune sera consisted of antibody populations that were sensitive and refractory, respectively, to depletion using monomers (Fig. 7, B and C). These results establish that immunization with stable dimers induced antibodies to shared epitopes between monomers and dimers and a new population of antibodies that only bound to the dimer.

The DENV binding signals for the monomer-depleted SC.10 and SC.14 immune sera at the lowest dilutions were higher than the control-depleted sera (Fig. 7B). This unexpected result can be explained by assuming that the antigens induce a mixture of abundant low-affinity (Abs^low^) and less-abundant high-affinity (Abs^high^) antibodies that are either directed to share epitopes on monomers and dimers or to dimer-specific epitopes. In undepleted or control-depleted serum samples, the abundant Abs^low^ will outcompete the minor population of Abs^high^ during initial binding to virus. However, the Abs^low^ are more prone to be washed off during the subsequent wash steps, resulting in lower binding signals. The monomer depletion will selectively remove the Abs^low^ and promote the binding of dimer-specific Abs^high^. The Abs^high^ remain bound during the wash steps, leading to higher binding signal at high concentrations of monomer-depleted sera. Similar increases in ELISA binding signal following depletion have been observed when examining sera from mice immunized with antigens from tick-borne encephalitis, and in this case, the explanation also involved a mixed population of higher- and lower-affinity antibodies (*46*). To further support our hypothesis, we created a mathematical binding model that simulates lower-affinity (*K*_d_ = 10 nM) and higher-affinity (*K*_d_ = 1 nM) antibody populations binding to virus and then unbinding during subsequent wash and incubation steps. Both populations of antibodies were given the same association rate constants typically observed with antibody-antigen binding, but the higher-affinity antibody population was assigned an off-rate that was 10-fold slower (fig. S6, A to C). When incorporating association times (*T*_A_) and dissociation times (*T*_D_) that match the incubation times and wash times used in the ELISA protocol into the model, we were able to reproduce the trends observed in the DENV2 ELISA (fig. S6, B and C). This suggests that the E dimer–specific antibodies elicited by SC.14 and SC.10, although lower in abundance, may be higher-affinity antibodies and compete for binding to similar regions on the virus as the more abundant, Abs^low^ whose epitopes shared between monomers and dimers.

**Fig. 8.**
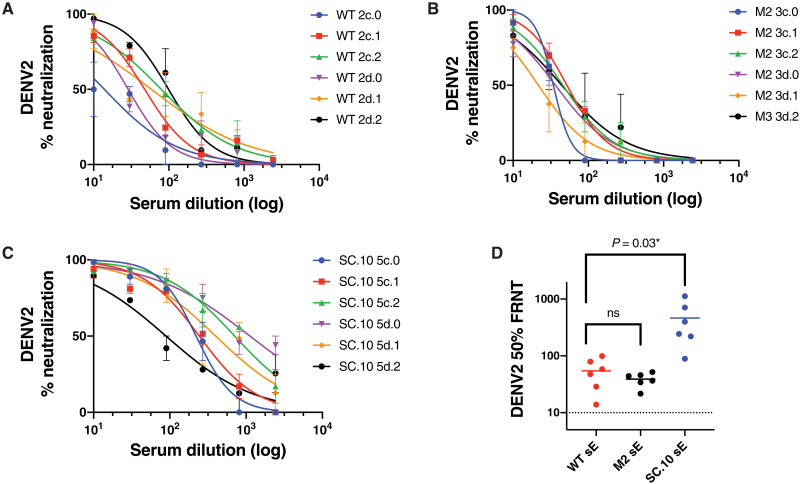
DENV2-neutralizing antibody levels in mice immunized with WT sE and stabilized dimer variants in the presence of Alhydrogel. Groups of six C57BL/6J mice were primed and boosted at 3 weeks with 5 μg of WT sE (**A**), Mnmer2 (M2) (**B**), or the SC.10 stable dimer (**C**) with 500 μg of Alhydrogel. All the mice were bled 5 weeks after the boost for antibody testing. To measure virus-neutralizing antibodies, each serum sample was serially diluted and tested in duplicate in a DENV2 focus reduction neutralization assay (A to C). The 50% DENV2-neutralizing antibody titers were significantly greater in mice immunized with the SC.10 sE antigen compared to titers in animals immunized with the WT sE antigen (**D**). The means of the antibody responses induced by the different antigens were compared by an unpaired *t* test. ns, not significant.

To characterize the levels of DENV2-neutralizing antibodies induced by these antigens, we repeated the immunization studies with WT sE, Mnmer2, and SC.10 antigens formulated with Alhydrogel as an adjuvant. Groups of six mice were primed and boosted 3 weeks later. The animals were bled 5 weeks after the boost (study week 8) to measure levels of DENV2-binding and DENV2-neutralizing antibodies. As anticipated, in the presence of adjuvant, the antigens stimulated higher levels of DENV2-binding antibodies compared to animals immunized with no adjuvant (Fig. 8 and figs. S5 and S8). With adjuvant, mice that received the SC.10 stable dimer variant developed significantly (*P* = 0.03) higher levels of DENV2-neutralizing antibodies compared to animals immunized with WT sE or Mnmer2 antigens (Fig. 8). In a second immunogenicity study, groups of five mice were immunized with WT sE and the SC.14 stable dimer variant formulated with Alhydrogel as an adjuvant. The group that received the SC.14 dimer variant had a trend (*P* = 0.08) of more DENV2-neutralizing antibody compared to the group that was immunized with the WT sE antigens (fig. S8).

## DISCUSSION

During the natural life cycle of flaviviruses, the E protein undergoes large conformational changes and interacts with a variety of macromolecular partners. These diverse functional requirements constrain the evolution of E protein sequences, and, therefore, native E protein sequences are unlikely to be optimized for any one conformation or interaction. One potential advantage of subunit vaccines compared to live attenuated viruses is that viability is no longer a constraint that restricts the sequence space that can be utilized. Instead, the focus can be on engineering molecules that are optimized to elicit a specific immune response. In our study, we demonstrate that structure-based protein design can be used to identify small numbers of amino acid mutations that markedly stabilize the E protein dimer and raise secretion yields more than 50-fold. Unlike the WT DENV2 sE protein, our design variants form dimers at 37°C and present quaternary epitopes recognized by human potently neutralizing antibodies. Our results highlight the degree to which native E protein sequences are not optimized for thermodynamic stability or dimer formation and suggest that a similar structure-based approach could be used to stabilize E proteins from other DENV serotypes and other flaviviruses. We anticipate that many of the stabilizing mutations that we have identified will also stabilize the E protein dimer from other DENV serotypes and Zika virus as the mutation sites are highly conserved (fig. S9).

One notable aspect of our results is the finding that mutations in a variety of regions within the E protein can stabilize the monomer and/or dimer. Interchain interactions in the E dimer are concentrated in two regions, the central αB dimer interface and the DII/DIII cross-chain interface. We show that mutations at either region can lead to a large increase in dimer affinity. IntFc2 lowers the *K*_d_ for dimerization more than 500-fold and is located on the underside of the central αB dimer interface. IntFc8 introduces a single mutation in the FL at the DII/DIII interface and lowers the *K*_d_ 59-fold (table S1). One likely reason why mutations similar to these are not observed in WT DENV sequences is that they would destabilize the energetic balance between the dimeric and trimeric postfusion conformations of the E protein and impair membrane fusion. In support of this hypothesis, the crystal structure of SC.10, which contains IntFc2 and IntFc8, adopts a dimer configuration even at low pH (Fig. 5A). UndPk6 is interesting because it lowers the *K*_d_ for dimerization by 54-fold, even though the mutations are in the DI/DII hinge and do not make interactions at the dimer interface (Fig. 5A and table S1). As the DII/DIII hinge is inherently flexible (*9*, *35*), it is likely that the UndPk6 mutations, especially T280P, are stabilizing the hinge in a conformation compatible with dimer formation and are lowering the entropic cost for forming the dimer. To examine these mutations further, we modeled the UndPk6 mutations (F279W and T280P) onto the structure of DENV2 sE in the postfusion trimer conformation, and the mutations are predicted to be destabilizing for the trimer. In the postfusion conformation, the phi/psi angles for residue 280 are −94°/101°, which is not within the allowed region of the Ramachandran plot for proline. Our studies also reveal that the low overall thermostability of the WT DENV2 sE monomer is, in large part, due to the low stability of DI. The PM4 mutation set, which contains three mutations within DI, raises the *T*_m_ for protein unfolding from 51.0° to 62.4°C but has little effect on the monomer-dimer equilibrium (Fig. 3B and table S1).

All the mutation sets that markedly raised monomer or dimer stability also increased the yield of recombinant sE. Different mechanisms may explain the increase in yield. From circular dichroism temperature melts with the WT protein, it is evident that sE rapidly aggregates when unfolded (*24*). Mutations that stabilize DI, i.e., PM4, may reduce transient unfolding events and also promote more efficient folding. Both of these factors would minimize aggregation during synthesis and folding. It is also known that secretion of the sE protein and DENV from cells can be hindered if the FL on DII embeds into the membrane of the host cell (*34*). During natural secretion of DENV, the FL is sequestered from the host membrane by its interaction with premembrane (prM) on the surface of the immature virus. In our studies, prM is not present; however, stabilization of the dimer sequesters the FL at the DI/DIII interface and may raise the energetic barrier for membrane association. In addition, IntFc8 introduces a new charged amino acid in the FL, which is likely to disfavor insertion of the FL into membranes (*34*). In general, we found that combining mutations from different regions of the protein led to additive increases in production yield.

A primary purpose of designing stabilized DENV2 sE dimers was to assess whether these stabilized dimers can elicit quaternary epitope antibodies that target the E dimer. Our results suggest that stabilized DENV2 sE dimers can elicit E dimer–specific antibodie*s* that appear to compete with monomer-specific antibodies, a phenomenon that has also been observed in sera of naturally infected individuals. Monomer-specific FL (FLE) antibodies and dimer-specific 2D22 and EDE antibodies have partially overlapping epitope regions on the DENV E protein and can thereby compete for binding. Furthermore, it has been shown that the dimer-specific antibodies have a higher affinity to DENV compared to monomer-specific FL antibodies (*15*). This is consistent with our observation that depleting serum of monomer-specific antibodies following immunization with a stabilized dimer leaves a population of antibodies that display increased binding to DENV2 (Fig. 7, B and C).

While the FL dimer interface regions contains the most well-characterized DENV2 type-specific quaternary antibody epitopes (such as 2D22), DENV-specific quaternary epitope antibodies that target epitopes at the αB helix central dimer interface, near the IntFc2 mutations, have also been isolated (*45*, *47*). Isolation and epitope mapping of the quaternary epitope antibodies elicited by SC.14 and SC.10 would be insightful to identify if stabilizing the central dimer interface with IntFc2 helps to focus the elicitation of antibodies to this region.

Our first set of immunization experiments were designed to specifically answer the question of whether the DENV2 sE monomers (Mnmer2 and WT) or the DENV2 sE dimer (SC.14 and SC.10) antigen conformations affect the epitope focus of antibody responses, rather than assessing the vaccinogenic potential of the novel E subunits. We intentionally performed the immunizations without adjuvant, which is known to affect the stability and epitope presentation of protein antigens (*46*, *48*–*50*). To assess whether the stabilized dimers (SC.10 and SC.14) also induced DENV2-neutralizing antibodies, we repeated the immunogenicity studies after formulating antigens with Alhydrogel as an adjuvant. Mice immunized with the SC.10 and SC.14 dimer variants developed higher levels of DENV2-neutralizing antibodies than mice immunized with the WT sE antigen.

Further studies are needed to define how the binding specificity and Fc effector properties of antibodies induced by different antigens are related to functional DENV neutralization and long-term protection in vivo. Large-scale production of recombinant flavivirus E antigens that retain native structure is challenging because the proteins, which must be produced in eukaryotic cells, are secreted at low levels. The DENV2-stable dimers, which were highly thermostable and secreted from cells at much higher levels compared to WT sE, overcome these practical limitations of antigens used in the past. Given the overall structural similarity of flavivirus virions and E proteins, our results will also guide the development of subunit vaccines to other emerging pathogenic flaviviruses of humans such as Zika, West Nile, and Yellow Fever viruses.

## MATERIALS AND METHODS

### Cell lines and viruses

EXPI293F (Thermo Fisher Scientific, catalog A14527) cells were maintained in EXPI293 expression medium at 37°C with 8% CO_2_ at 250 rpm without antibiotics until passage 25 to improve batch-to-batch reproducibility. Vero-81 (CCL-81) cells were obtained from the American Type Culture Collection. Vero cells were grown at 37°C with 5% CO_2_ in Dulbecco’s modified Eagle’s medium (DMEM), 5% fetal bovine serum (FBS), penicillin (100 U/ml), streptomycin (100 mg/ml), 1% GlutaMAX, 1% sodium bicarbonate, and 1% nonessential amino acids. The RNA genome of DENV2 strain 16681 was used for cloning of stabilizing mutations for transfection into C6/36 for virus induction.

### Rosetta computational design methods for predicting DENV2 sE–stabilizing mutations

#### DENV2 sE dimer structure preparation and selection of seed residues

We used four DENV2 sE crystal structures as input for design—PDB 1OAN, 1OKE, 1TG8, and 4UTC (*16*, *35*, *51*)—to capture the structural differences present in each DENV2 sE dimer crystal structure. All glycan atoms, waters, and metal ions were removed and not considered during simulations. For use of 4UTC and 1TG8 models containing all 394 residues, the unmodeled loops in 4UTC in chain A (residues 189 to 187 and 192 to 195) and chain B (residues 14 to 19) were modeled by aligning the chain A to chain B, extracting the coordinates of the chain containing the modeled loop and adding them to the chain with the missing residues. For 1TG8, the coordinates of residues 17 to 18 from chain A of 4UTC and residues 222 to 232 from 1OAN were added and replaced, respectively, into 1TG8. All four structures were minimized five times using the Rosetta FastRelax protocol with coordinates constrained to the starting crystal structure coordinates.

Seed residues were selected manually with and without the use of Rosetta. For the IntFc simulations, seed residues present at the sE dimer interface, which allowed for overlapping design spheres, were selected for complete sampling of the dimer interface. The HCat simulation seed residues were influenced by the previous reports highlighting the role of pH “sensing” histidine residues present in flavivirus E involved in the low pH conformational change of E (*9*, *52*). We identified histidine residues present at the dimer interface (H144, H27, and H261) that were in close proximity to cationic residues, suggested to be transiently stable histidine-cation clusters, HisCat, and selected these as seed residues for the HCat simulations. The UndPk simulations were led by the hypothesis that we could stabilize the sE dimer by stabilizing the sE monomer conformation observed in the dimer crystal structure. We used the RosettaHoles application to identify “underpacked” regions that contain nonideal residue packing that produce small cavities in the protein that cannot be satisfied by water, within the sE monomer that contribute to protein instability (*30*). A subset of residues that defined and allowed for complete sampling of these regions were selected as seed residues for the UndPk simulations.

#### IntFc, HCat, and UndPk local cluster mutation (cluster_mut) design simulations

We used two computational design strategies to identify DENV2 sE–stabilizing mutations. The first strategy focused on designing residues that locally cluster within 10 Å (cluster_mut) of each other with a selected “seed residue” that defined the center of the cluster. Within this 10-Å cluster, we created a “design sphere” that selected all residues present within 7 Å of, and including, the seed residue and designated these residues as “designable,” allowing Rosetta to mutate these residues together to any of the canonical 20 amino acids, except cysteine. We also created a “repacking sphere” that selected residues 3 Å outside the design sphere, which do not change the amino acid identity but allow for new side chain conformations (or rotamers) and backbone sampling to relieve clashes from the new mutations in the design sphere. This simulation favors multiple mutations to promote introduction of new interactions that may not be attainable with a single mutation to native neighboring residues. The designed sequences for each chain were forced to be identical to ensure that the final design model was a sequence-symmetric homodimer, but the backbone movements in either chain are not forced to be symmetric, accounting for the asymmetry observed in the crystallographic dimers. To prevent large backbone changes, the simulation was designed to deviate minimally (± 2-Å deviation) from the backbone conformation observed in the crystal structure and to reduce noise in the simulations; all residues outside the 10-Å cluster were not sampled and remained fixed. During the simulations, we applied an artificial increase in the WT amino acid Rosetta score (using FavorNativeBonus values of 0.0, 0.75, and 1.5), at a given residue position, which applies stringency during the simulation and increases the probability of the Rosetta score function accepting favorable mutation sets while restricting the number of mutations per simulation to a reasonable number of mutations (~1 to 5). One hundred models were created for each run, and the scores were compared to the average top three nonmutated model control run Rosetta total scores, where the protocol was performed on the same selected residues, but the amino acid sequence was not changed to obtain the ∆REU score (analogous to ∆∆*G*_mut_).

#### Point mutations in silico site-saturation mutagenesis (PM_ssm), combination (PM_comb), and SHP simulations

The second strategy is the point mutation site-saturation mutagenesis protocol (PM_ssm) that starts at the first position of the protein, and Rosetta mutates the WT amino acid to each of the 20 amino acids in independent simulations. This is repeated in both chains simultaneously until Rosetta walks through the entire primary sequence. During the simulation, the residue position to mutate and the residues before (*i* − 1) and after (*i* + 1) in primary sequence are selected. At the mutated position, the backbone can move with no constraints, while the two neighboring residues can sample side chain conformations and backbone conformations ±2 Å from the starting coordinates. All residues within 10 Å of the mutated residue and its neighbors are allowed to sample side chain conformations and backbone conformations with backbone constraints (±2-Å deviation). A total of 10 models were generated for each point mutant simulation, and the total Rosetta score for mutating to the WT amino acid was used as reference to calculate the ∆REU score (∆∆*G*_mut_) with an arbitrary cutoff ∆REU = −2 per monomer per mutation, indicating predicted stabilizing mutations.

In addition to the PM_ssm, we used multiple sequence alignment (MSA) from representative DENV2 strains to identify residue positions that varied in amino acid identity in the alignment, similar to (*53*), to identify additional stabilizing point mutations. We then took the output of the mutations identified in the PM_ssm simulations, and the MSA analysis used those predicted mutations as input for combining point mutation (PM_comb) simulations. The seed residue positions for the PM_comb simulations were based on the mutated positions identified in the MSA analysis, named “atMSA” positions, and all other positions identified from the PM_ssm simulations were named “notatMSA”. The PM_comb SHP simulations used different seed residue logic as these positions were manually selected surface hydrophobic positions, and the simulations were restricted to only the polar mutations from the PM_ssm at those positions. We ran simulations where only mutations that were found from the MSA analysis were allowed, but manual inspection eliminated further consideration of these mutations. We then focused on the mutations obtained from PM_ssm, either atMSA or notatMSA. These PM_comb simulations used the selection logic from the PM_ssm simulations, but we allowed to simultaneously mutate multiple positions in the entire domain, domain-domain interfaces, or dimer interface. For the domain simulations, all residues in the domain had constrained backbone sampling (±2-Å deviation) and side chain sampling. For the interdomain and dimer interfaces, residues within a sphere of 10 Å around the combined mutated positions had constrained backbone conformation (±2-Å deviation) and side chain sampling. The same WT score artificial increase used in cluster_mut was applied to all PM_Comb simulations. The SHP simulations were divided by domain but followed the interdomain/dimer interface selection and sampling logic. Ten models were generated for each PM_comb and SHP simulation, and ∆REU was calculated as indicated in the PM_ssm description.

### DENV2 sE protein construct design, cloning and plasmid preparation for cell surface display, and soluble expression

DNA encoding the DENV2 (strain 16681) sE protein (residues 1 to 394), lacking the stem and transmembrane domains, were cloned into the modified pαH mammalian expression vector, originally derived from the pHLSec vector (*54*) by substitution of an alternative multiple cloning site, containing a CAG promoter and human serum albumin signal peptide, replacing the original protein tyrosine phosphatase α (PTPα) signal sequence. DENV2 sE was expressed for both mammalian cell surface display and soluble expression using the same pαΗ vector backbone, and plasmids were renamed to differentiate function. For mammalian surface display, sE genes were cloned into the pD2sE_Dsp display vector, which genetically fuses a glycine-serine (GS) GS_3x_-cMyc-GS_3.5x_ linker and the MHC Iα transmembrane/cytoplasmic domains to the C terminus of sE, for membrane anchoring, following a modified system, previously reported (*32*), where we added a cMyc epitope tag for independent detection of sE surface display. For soluble expression, sE genes were cloned into the pD2sE_EV8 expression vector containing a C-terminal GS_6x_-His_8x_ tag for immobilized metal affinity chromatography purification. All DENV2 sE Rosetta variant and SC variant cloning were performed by Twist Bioscience. DENV2 sE cloned plasmids were heat-shock transformed into *Escherichia coli* DH5α cells for amplification, miniprepped or midiprepped using DNA endotoxin-free kits, and stored in endotoxin-free H_2_O at −20°C (Macherey-Nagel).

### Small- and large-scale DENV2 sE protein expression and purification

#### Small-scale expression and purification of DENV2 sE variants

EXPI293F cells were transiently transfected according to the manufacturer’s protocol with a modified cell density of 3.0 × 10^6^ cells/ml and 75% of the total final volume (i.e., 90 ml for 120 ml of final culture), with 1 μg of DENV2 sE protein containing plasmid per milliliter of cell culture, for 18 hours at 37°C with 8% CO_2_ at 250 rpm. Following transfection, enhancers 1 (600 μl at 120-ml scale) and 2 (6 ml at 120-ml scale) and 12 ml of 3.5% of Hyclone Cell Boost 1 (GE Healthcare) were added to the cells to increase protein expression and reduce cell death. Cell culture supernatant was harvested after 24 hours by pelleting cells at 1000 relative centrifugal force (rcf) at room temperature (RT) for 10 min and further clarified using a 0.22-μm vacuum filter (Millipore). DENV2 sE His-tagged proteins were affinity purified by batch-binding clarified medium with pre-equilibrated penta-Ni resin (Marvelgent) for 1 hour at 4°C and then transferred into a column to collect the resin. A two-step wash was performed by gravity at RT (~23°C) to eliminate protein contaminants. The first wash was performed three times with 5 CV (column volume) of high-salt tris buffer [50 mM tris (pH 8.0) and 1 M sodium chloride with 25 mM imidazole], and the second was performed with 1 CV wash with 1× PBS (pH 7.4) with 50 mM imidazole. Bound proteins were eluted in one step using 5 CV (two times with 400 μl and two times with 600 μl) of 1× PBS (pH 7.4) and 500 mM imidazole to collect the protein and then sequential concentration at 4°C using a 10K MWCO Amicon-4 followed by an Amicon-0.5 centrifugal filter at maximum allowed rcf for each filter. The concentrated elution was buffer exchanged using a 1Å~ PBS (pH 7.4) pre-equilibrated 7K 0.5-ml MWCO ZebaSpin desalting column (Thermo Fisher Scientific). Purity was assessed by SDS–polyacrylamide gel electrophoresis (SDS-PAGE), by loading 3.5mg. Concentrations were measured and calculated by measuring the absorbance at 280 nm and using the ProtParam-derived theoretical extinction coefficient. Yields are reported as yield after purification in milligrams of protein per liter of culture.

#### Large-scale production for crystallography and immunogenicity studies

Mammalian expression vectors encoding for soluble DENV2 sE proteins were transfected into EXPI293F cells, as per the manufacturer’s protocols (Thermo Fisher Scientific), and culture supernatants were harvested on day 4 after transfection. Supernatants were then buffer exchanged by tangential flow filtration into TALON binding buffer [50 mM sodium phosphate (pH 7.4), 500 mM NaCl, 5 mM imidazole, 0.02% sodium azide, and 10% glycerol] and then run through hand-packed TALON columns with flow adapters, recirculating overnight at 4°C. The next day, columns were switched to flow through, and after complete loading, columns were washed with 10 CV of binding buffer, followed by 10 CV of binding buffer with 10 mM imidazole. Bound proteins were eluted with 10 CV of elution buffer (binding buffer with 150 mM imidazole). Wash and elution fractions were analyzed by SDS-PAGE, and DENV2 protein–containing fractions were pooled and concentrated before further purification by SEC on Superdex 200 16/600 into PBS with 10% glycerol. Fractions containing DENV2 proteins were then pooled, concentrated, aliquoted, and flash-frozen in liquid nitrogen and stored at −80°C. Endotoxin levels were measured at 1:20 dilution in endotoxin-free water with a nexgen-PTS reader (Charles River Laboratories).

### Epitope presentation screen of DENV2 sE Rosetta variants using mammalian surface display

Mammalian display vectors containing Rosetta DENV2 sE variant genes were transiently transfected 1 μg of plasmid into 1 ml of EXPI293F cell culture using the manufacturer’s protocols for 18 hours at 37°C in 8% CO_2_ in a 2-ml 96-well deep well plate (Axygen, #P-2ML-SQ-C-S) shaking at 1000 rpm using an Orbishaker MP 3-mm orbital plate shaker. After transfection, 5 μl of enhancer 1 and 50 μl of enhancer 2 were added to each transfected culture and incubated for an additional 24 hours. After 42 hours after transfection (24 hours after enhancement), cell densities were measured using a LUNA-II cell counter (Logos Biosystems) by mixing 1:1 volume of cell culture to trypan blue stain from six randomly selected wells on the plate to calculate the average cell density and normalize the cell amount for immunostaining. Cells were harvested by centrifugation at 1000 rcf for 3 min at RT and washed one time with 800 μl of RT flow buffer, 1× PBS (pH 7.4), 1 mM EDTA, and 2% heat-inactivated FBS. After washing, the cells were resuspended with RT flow buffer to a final density of 5.0 × 10^6^ cells/ml, and 1.5 × 10^6^ cells were transferred to a 1-ml 96-well deep well plate (Eppendorf, #951032603). Cells were then labeled with 100 μl of chicken anti-cMyc immunoglobulin Y (IgY) fraction polyclonal antibody (10 μg/ml) at RT for 10 min at 1000 rpm using an Eppendorf ThermoMixer C with SmartBlock DWP1000 adapter. Cells were centrifuged at 1000 rcf for 3 min at RT and washed two times with RT flow buffer (one time with 500 μl and one time with 600 μl). All wash steps followed this protocol except where noted. Cells were then labeled with 100 μl of anti-chicken antibody (2 μg/ml), labeled with Alexa Fluor 488, at RT for 15 min at 1000 rpm. Cells were washed two times with RT flow buffer and labeled with 100 μl of DENV anti-E antibody (3H5, 1C19, EDE1 C8, EDE1 C10, or 2D22) (1 μg/ml) for 1 hour at either RT or 40°C at 1000 rpm using ThermoMixer C and deep well adaptors to ensure homogenous mixing and heating. Cells were then centrifuged at the same temperature used for labeling and washed two times (one time with 600 μl and one time with 700 μl) with either RT flow buffer for RT stained cells or pre-equilibrated 40°C flow buffer for 40°C stained cells. Cells were then left at RT for 15 min to allow for equilibration to RT and stained with 100 μl of anti-human IgG PerCP (5 μg/ml) or anti-mouse IgG PerCP (2 μg/ml) for 10 min at RT. Cells were washed two times with RT flow buffer and resuspended in 500 μl of flow buffer for analysis. Cells were analyzed using a Millipore Sigma Guava 5HT flow cytometer with excitation of both Alexa Fluor 488 and PerCP with 488-nm laser and gating on the Alexa Fluor 488–positive fluorescence in the green fluorescence channel (525/30), and 20,000 events were collected in the red fluorescence channel (695/50). Cells transfected with only the GS linkers, cMyc, and MHC Iα anchor (MHC ctrl) and stained with anti-cMyc, 3H5, anti-chicken Alexa Fluor 488 was used as fluorescence minus one (FMO_green_) controls to set the green fluorescence channel voltages baseline, and the same cells stained with anti-cMyc, 3H5, and anti-mouse PerCP were used to set red fluorescence channel baseline (FMO_red_). FMO_green_ controls were also used for compensation of the red fluorescence channel to eliminate Alexa Fluor 488 fluorescence spillover. Files were converted to .fcs format using Guava InCyte software and gating and using MHC ctrl–stained cells, and geometric mean fluorescence intensities (GMFIs) were performed and obtained using FlowJo and Microsoft Excel. Fluorescence ratio was calculated by dividing the PerCP GMFI of the sE variant–displayed cells stained with anti-E antibody by the GMFI of the WT sE-transfected cells stained with the same antibody. Anti-DENV antibody stained cells with <1000 events and had no observable signal upon manual review were considered negative, and the PerCP-negative GMFI (Q4) was used for Fr calculations.

### Assessment of DENV2 sE variant dimer and monomer protein stability using nanoDSF

sE proteins were stored in 1× PBS (pH 7.4) and diluted to 2, 4, or 8 μM into the same buffer and maintained on ice before analysis. Thermal melts were performed using a NanoTemper Prometheus NT.48, in duplicate, by transferring 10 μl of diluted protein into a capillary and monitoring fluorescence at 330 or 350 nm, as designated, with a ramping rate of 1°C min^−1^ from 15° to 95°C. Melting transition points (*T*_m_) were calculated using Nanotemper PR.ThermControl software. For van’t Hoff analysis, thermal melts were performed at concentrations ranging from 2 to 16 μM with same settings as single concentration measurements. The concentration tested and the measured *T*_m_^1^ were used for 1/*T* and ln(*K*_d_) values, respectively, as previously determined (*24*). Ninety-five percent confidence limits for the homodimer *K*_d_s at 37°C were obtained using error propagation and the linear regression fits used for van’t Hoff analysis.

### SEC-MALS to validate DENV2 sE variant dimer stability

Proteins were centrifuged at 14,000 rcf for 10 to 12 min at 4°C to remove any debris or aggregates, and concentrations were obtained by measuring the absorbance at 280 nm and using theoretical molar extinction coefficients with monomer molecular weights before analysis. Ice-cold DENV2 sE proteins at 2.5 mg/ml (250 μg), with exception for DENV2 sE Mnmer2 (1.5 mg/ml, 150 μg) and DENV2 sE IntFc2 (1.6 mg/ml, 160 μg), were loaded at a flow rate of 0.5 ml min^−1^ with 1× PBS (pH 7.4) + 200 mM sodium azide onto a GE Superdex 200 10/300 GL column–equipped Agilent FPLC system, interfaced with a Wyatt DAWN HELEOS II light scattering instrument, Wyatt T-rEX refractometer, and a Wyatt dynamic light scattering module. All buffers and equipments were maintained at either RT (~23°C) or 37°C for the entire experiment. For 37°C experiments, buffers were placed in a temperature-controlled water bath, and all WYATT equipments were set to 37°C. The column was wrapped in 12-inch, THG Thermal Tape (Reptile Basics Inc.) and regulated by a digital thermostat (RINGDER RC-112R) set at 37°C with 0.01°C difference (diff = 0.01)s and 5-second delay (delay = 005). The entire system was equilibrated for ~2 hours before protein analysis to ensure equilibration, and WAYTT systems reached baseline signal. MALS data were collected and analyzed using Wyatt ASTRA (version 6) software.

### Crystal structure of DENV2 sE SC.10

SC.10 was concentrated to 3.3 mg/ml, aliquoted, and stored at −80°C in 1× PBS (pH 7.4) + 10% glycerol. Before laying drops, the protein was quickly hand-thawed and buffer exchanged into 25 mM tris (pH 7.4) with 0.1 M sodium chloride using a Thermo Fisher Scientific Zeba 7K MWCO desalting column. Crystallization screening was performed by sitting drop vapor diffusion method by mixing 100 nl of SC.10 and with 100 nl of precipitant solution using a SPT Labtech mosquito LV robot. Diffraction quality crystals of SC.10 were grown by mixing 200 nl of protein solution with 200 nl of precipitant solution containing 15% polyethylene glycol 3350 (pH 4.4), 0.1 M sodium acetate, and 0.1 M sodium iodide. SC.10 crystals were formed after 3 days at 21°C. The crystals were cryo-protected with the mother liquor containing 25% glycerol. The crystals diffracted to a final resolution of 3.42 Å. The diffraction data were processed and scaled using XDS (*55*) and Aimless (*56*). The phases were obtained by MR with Phaser (*57*) by using chain A of PDB: 1OAN, the structural model used for the design of IntFc2, IntFc8, and UndPk6. The MR failed when using the entire 1OAN chain A but was successful when using two separated models (DI/DII and DIII) generated from 1OAN chain A. The final model was built using Coot (*58*, *59*) and iterative refinement with Phenix (*60*) to a *R*_free_/*R*_work_ of 33.7/31.8, with the asymmetric unit containing a single sE monomer. Among structures of similar resolution, PDB 6WY1 (SC.10) has the lowest MolProbity clashscore (*61*) and has a model quality consistent with a resolution of 2.4 Å. Electron density for all mutations was observed, with the strongest density observed for G106D, F279W, T280P, and A259W, including the first *N*-acetylglucosamine moiety of N67. Residues 148 to 158 consisting of the DI glycan loop residues were not modeled because of insufficient density. A strong positive density is observed in the map near residue 144 that was not modeled in the PDB-deposited model; however, the N-terminal methionine conformation present in the previously reported DENV2 sE dimer crystal structures matches the position of the unmodeled positive density present in our map. An alternative model, modeled with the N-terminal methionine matching the 1OAN N terminus, satisfies this positive density but leaves an even stronger positive density near the FL unsatisfied (fig. S3C). Modeling alternative conformations for residues 1 to 3 in both conformations does not satisfy either density. The deviation of the N terminus in our structure is consistent with the N-terminal conformational change observed in the low pH structure of DENV2 sE complexed with prM (PDB 3C5X) and also observed in nearby regions in our structure such as near the ij loop and FL. Chain D in the PDB 4UT9 EDE1 C10 antibody–bound sE structure also contains a N-terminal conformation that deviates from the canonical DENV2 sE N-terminal conformation, giving further evidence to the potential for multiple N-terminal conformations (*16*). The density near the FL is also weaker in comparison to the rest of the structure, suggesting that this region is likely affected by low pH-induced mobility. The deviation of the FL W101 conformation has also been observed in a ZIKV sE crystal structure complexed with an antibody bound to DI/DIII (PDB 6NIU, chain A) (*62*).

### Direct ELISA to measure monoclonal antibody binding to DENV2 sE variants

Ni^2+^-coated ELISA plates (Thermo Fisher Scientific, PI15142) were used to capture 45 nM sE protein in 1× tris-buffered saline (TBS) [50 mM tris-Cl (pH 7.5) and 150 mM NaCl] for 1 hour at 37°C. The plate was washed three times with 1× TBS + 0.2% Tween 20 (wash buffer). Next, the plate was blocked using 1× TBS + 3% skim milk + 0.05% Tween 20 (blocking buffer) for 1 hour at 37°C and subsequently washed three times with wash buffer. The plate was then incubated for 1 hour at 37°C with 2 ng/μl of CR or TS human monoclonal antibody—1M7 (Flavi CR), 1C19 (DENV CR), 3F9 (DV2 TS), 2D22 (DV2 TS), EDE1 C8 (DENV/ZIKV CR), and EDE2 A11 (DENV CR)—or mouse monoclonal antibody—3H5 (DV2 TS) and 4G2 (CR)—in blocking buffer and washed three times with wash buffer. The wells were accordingly treated with either alkaline phosphatase (AP)–conjugated anti-mouse IgG (1:1000; Sigma-Aldrich) or AP-conjugated anti-human IgG (1:2500; Sigma-Aldrich) for 45 min at 37°C. Last, the plate was washed and developed with AP substrate (Sigma-Aldrich), and the absorbance was measured at 405 nm.

### Binding analysis between DENV2 sE variants and anti-E antibodies using BLI

BLI experiments were carried out using the ForteBio Octet Red384 system with DENV2 sE proteins immobilized on Ni-NTA biosensors (Satorius, #18-5101) and antibodies as analytes. All protein solutions used for BLI experiments were prepared in 1× kinetics buffer (Satorius, #18-1105) at RT. Ten nanomolar sE proteins (WT, Cm1, Mnmer2, SC.10, and SC.14) were used for loading. Antibody samples were prepared by twofold serial dilutions with monomer epitope antibodies (4G2 and 3H5) concentrations ranging from 50 to 3.125 nM and quaternary epitope antibodies (2D22, EDE1 C8, and EDE1 C10) from 50 to 6.25 nM. Measurements in the absence of analyte were used as reference for data subtraction. Kinetics buffer (1×) was used to equilibrate the sensors before loading of sE. Each solution (250 μl) was added to a sample tray (Greiner, #655209). To start the experiment, Ni-NTA biosensors were soaked in water at RT for 15 min, while the sample tray was incubated at 37°C. All the following steps were performed at 37°C. The sensors were then equilibrated for 15 min, loaded with sE proteins for 5 min, and washed for 2 min in 1× kinetics buffer. The immobilized proteins were incubated with antibody solutions for 15 min (association) and then in buffer for 25 min (dissociation). BLI sensorgrams were analyzed using the ForteBio Data Analysis 11.0 software. Sensorgrams were subtracted by the reference data and processed after aligning the *y* axis by baseline and correcting the interstep by aligning to dissociation. Steady-state binding constants were calculated using the maximum response (*R*_max_) for each antibody concentration.

### Mouse immunogenicity studies

#### Immunogenicity studies at the University of North Carolina

Mouse experiments were performed under protocols approved by the Institutional Animal Care and Use Committee of the University of North Carolina. All experiments followed ethical and federal regulations according to the Public Health Service Policy on Humane Care and Use of Laboratory Animals, Animal Welfare Act, and the Guide for the Care and Use of Laboratory Animals. In the first immunogenicity study performed without adjuvant, 6-week-old female Balb/c mice (Jackson Laboratory) were immunized subcutaneously with DENV2 rE Mnmer2 (*n* = 5), rE WT (*n* = 5), rE SC.14 (*n* = 5), rE SC.10 (*n* = 5) antigens, or vehicle (isotonic 9.25% sucrose/H_2_O) alone (*n* = 3). All groups were primed and boosted at weeks 3 and 8 (5 μg of antigen per dose per mouse), and serum samples were collected at week 16 by submandibular bleed to characterize DENV2-specific antibodies. In the second study performed with Alhydrogel as an adjuvant, 6-week-old female *C75BL/6J* mice (Jackson Laboratory) were immunized subcutaneously with 10 μg of rE WT (*n* = 5), rE SC.14 (*n* = 5), or vehicle (isotonic 9.25% sucrose/H_2_O) alone (*n* = 4) formulated with Alhydrogel (Invitrogen) as an adjuvant (250 μg per dose). All groups were primed and boosted at week 3, and serum samples were collected at week 9 by submandibular bleed to characterize DENV2-specific antibodies.

#### Immunogenicity studies at the Walter Reed Army Institute for Research

Research was conducted under an animal use protocol approved by the Walter Reed Army Institute for Research/Naval Medical Research Center Institutional Animal Care and Use Committee in an AAALAC (American Association for Accreditation of Laboratory Animal Care) International–accredited facility, in compliance with the Animal Welfare Act and other federal statutes and regulations relating to animals and experiments involving animals, and adheres to principles stated in the *Guide for the Care and Use of Laboratory Animals*, NRC Publication, 2011 edition. Groups of six 8-week-old female C57BL/6J mice (Jackson Laboratory) were subcutaneously immunized with DENV2 rE WT, rE Mnmer2, or rE SC.10 antigens (5 μg of antigen per dose per mouse) with or without Alhydrogel (Invitrogen) (500 μg of adjuvant per dose). All groups were primed and boosted at week 3, and blood samples were collected at week 8 by submandibular bleed to characterize DENV2-binding and DENV2-neutralizing antibodies.

#### Depletion of variant E antigen-specific antibodies from mouse immune sera

DENV2 sE Mnmer2 or SC.14 (35.2 μg) at 75 μg/ml in equilibration buffer [50 mM HNa_2_O_4_P (pH 8), 0.3 M NaCl, and 10 mM imidazole] was loaded into 880 μg of Ni-NTA magnetic beads (HisPur, Thermo Fisher Scientific, #88832), in a 1.7-ml Eppendorf tube, at a 4-μg antigen to 100-μg bead ratio, and incubated at 37°C for 30 min, mixing 1000 rpm using a ThermoMixer C. Antigen-loaded beads were washed two times with 400 μl of equilibration buffer. Serum diluted (1:50) was depleted by incubating 440 μg of antigen-loaded beads for 1 hour at 37°C while mixing at 1000 rpm, then briefly centrifuged for 30 s at 2400 rcf at 37°C and placed onto a magnetic strip to collect beads, and repeated for one to two more rounds of depletion. Sera depletion was considered complete when, compared to undepleted sera, ELISA analysis of depleted sera at 1:100 against the homologous depletion antigen resulted in reduction of signal to background levels. Depleted sera were stored at 4°C until further analysis.

##### Characterization of depleted mouse immune sera by ELISA

Undepleted and depleted mouse sera were characterized by antigen-capture ELISA. To evaluate post-depletion binding to DENV2 virus, 96-well ELISA plates (Greiner, #655061) were coated with 1M7 (2 μg/ml) in 0.1 M bicarbonate buffer (pH 9.6) overnight (~18 hours) at 4°C and then washed three times with wash buffer. Next, the plates were blocked with blocking buffer for 1 hour at 37°C. After washing, DENV2 in culture supernatant was diluted 1:10 in blocking buffer and added to each well and incubated for 1 hour at 37°C for capture by 1M7. To determine post-depletion binding to Mnmer2 (M2) or SC.14 antigens, Ni^2+^-coated ELISA plates (Pierce) were incubated with 50 μl of Mnmer2 or SC.14 protein, at 2 μg/ml, for 1 hour at 37°C and were blocked with blocking buffer for 1 hour at 37°C and washed three times with washing buffer. Following blocking, the DENV2 capture and Mnmer2/SC.14 direct ELISAs all followed the same protocol. Undepleted or depleted mouse serum was serially diluted in blocking buffer and incubated for 1 hour at 37°C. After incubation, the plates were washed in wash buffer and incubated with AP-conjugated anti-mouse IgG (1:1000 dilution; Sigma-Aldrich) for 45 min at 37°C. Next, the plates were washed and developed with the AP substrate (Sigma-Aldrich), and the absorbance was measured at 405 nm. End point dilution (EPD) is determined by the dilution where detection levels reach background levels obtained from data fitting using a linear regression curve with a sigmoidal slope, calculated by the GraphPad Prism software.

### DENV2 neutralization assay

Ninety-six-well flat-bottom plates were coated with 2 × 10^4^ Vero-81 cells per well and incubated at 37°C overnight. Neutralization titers were determined by threefold serial dilutions of sera mixed with 30 to 40 focus-forming units per well of DENV2 in DMEM/F12 supplemented with 2% FBS. The sera-virus mixtures were incubated at 37°C for 1 hour before transferring to the 96-well plates containing confluent Vero-81 monolayers. Following an additional 1 hour of incubation at 37°C, the medium on the monolayers was replaced by Opti-MEM (Gibco, Thermo Fisher Scientific) containing 2% FBS and 1% (w/v) carboxymethyl cellulose (Sigma-Aldrich). Infected cells were left to incubate for 44 hours at 37°C with 5% CO_2_. Next, cells were washed with 1× PBS and fixed with 4% paraformaldehyde. Fixed cells were permeabilized and followed by blocking with 5% nonfat dried milk in permeabilization buffer. The cells were then incubated with a mix of 4G2 and 2H2 primary antibodies for 1 hour at 37°C, washed, followed by secondary horseradish peroxidase–conjugated anti-mouse IgG [Kirkegaard & Perry Laboratories (KPL) antibodies, LGC Clinical Diagnostics, Inc.] for 1 hour at 37°C, washed again, and developed with TrueBlue peroxidase substrate (KPL). The reaction was stopped with water, and the plates were air-dried before counting the foci on an ImmunoSpot S6 analyzer (Cellular Technology Limited) using the ImmunoSpot double-count software. We calculated 50% focus reduction neutralization test (FRNT_50_) values using the sigmoidal dose response (variable slope) equation in GraphPad Prism software.

## Notes

View/request a protocol for this paper from *Bio-protocol*.
