# Supplementary material for: Designed, highly expressing, thermostable dengue virus 2 envelope protein dimers elicit quaternary epitope antibodies
Source: Sci Adv. 2021 Oct 15;7(42):eabg4084. doi: 10.1126/sciadv.abg4084 (PMC8519570; doi:10.1126/sciadv.abg4084)
Supplement: Supplementary file 1 — Figs. S1 to S9 Tables S1 and S2 [file sciadv.abg4084_sm.pdf]

Science Advances
AAAS

Supplementary Materials for

Designed, highly expressing, thermostable dengue virus 2 envelope protein dimers elicit quaternary epitope antibodies

Stephan T. Kudlacek, Stefan Metz, Devina Thiono, Alexander M. Payne, Thanh T. N. Phan, Shaomin Tian, Lawrence J. Forsberg, Jack Maguire, Ian Seim, Shu Zhang, Ashutosh Tripathy, Joseph Harrison, Nathan I. Nicely, Sandrine Soman, Michael K. McCracken, Gregory D. Gromowski, Richard G. Jarman, Lakshmanane Premkumar, Aravinda M. de Silva*, Brian Kuhlman*

*Corresponding author. Email: bkuhlman@email.unc.edu (B.K.); aravinda_desilva@med.unc.edu (A.M.d.S.)

Published 15 October 2021, *Sci. Adv.* **7**, eabg4084 (2021)
DOI: 10.1126/sciadv.abg4084

The PDF file includes:

Figs. S1 to S9
Tables S1 and S2

Other Supplementary Material for this manuscript includes the following:

Data files S1 to S4

	RT (~23˚C)					40˚C				
	cMyc	1C19	3H5	EDE1 C8	2D22	cMyc	1C19	3H5	EDE1 C10	2D22
HCat1	0.9	0.7	0.8	0.6	0.7	0.3	0.5	0.7	0.9	0.7
HCat2	1.3	1.4	0.9	1.4	1.3	1.1	1.4	0.7	1.3	1.5
HCat3	1.2	1.5	0.7	1.9	1.6	1.1	2.5	0.6	3.0	2.8
HCat4	0.7	1.4	0.7	1.9	1.4	1.0	1.7	0.7		2.0
HCat5	1.6	2.7	0.9	1.4	1.7	0.2	0.3	0.5	0.1	0.6
HCat6	0.3	0.4	0.4	0.4	0.6	0.2	0.4	0.6	0.2	0.6
HCat7	0.9	1.2	1.0	1.0	1.0	0.1	0.3	0.4	0.2	0.6
HCat8	0.4	0.6	0.9	0.6	0.6	1.1	1.6	0.7	1.9	1.6
IntFc1	1.2	1.1	0.8	1.1	1.1	1.2	1.3	0.7	1.0	1.2
IntFc2	1.1	1.4	0.8	1.3	1.3	1.3	2.5	0.6	3.4	2.5
IntFc3	1.1	0.8	1.0	0.8	0.8	1.1	1.4	0.6	1.7	1.5
IntFc4	1.4	1.1	0.9	1.2	1.2	1.2	1.4	0.6	1.6	1.4
IntFc5	1.3	1.3	0.9	1.5	1.4	1.1	1.6	0.6	1.6	1.4
IntFc6*	0.4	0.8	0.6	1.3	1.0	0.2	0.0	0.0	0.0	0.0
IntFc7	1.0	1.4	1.0	1.2	1.2	1.1	1.9	0.8	1.9	1.9
IntFc8	2.0	2.0	0.5	3.3	2.5	2.4	3.5	0.4	5.9	4.4
UndPk1	1.3	1.0	1.0	1.1	1.1	1.2	1.4	0.6	1.3	1.4
UndPk2	1.0	1.2	0.8	1.2	1.2	0.4	0.4	0.8	0.2	0.6
UndPk3	1.2	0.9	0.9	1.3	1.0	1.0	1.3	0.6	1.2	1.6
UndPk4	0.7	1.2	0.5	1.4	1.4	0.6	1.0	0.4	1.5	1.3
UndPk5	1.3	1.4	1.0	1.4	1.8	1.2	1.7	0.6	2.7	2.8
UndPk6	1.6	2.4	0.8	2.3	2.7	1.6	2.9	0.4	4.1	4.9
UndPk7	1.0	1.3	1.0	2.2	2.0	1.3	2.1	0.6	2.4	2.6
UndPk8	0.9	1.3	1.0	1.3	1.4	1.1	1.8	0.7	1.9	1.9
PM1	0.9	0.4	1.1	1.0	0.8	1.0	0.3	0.9	0.1	0.1
PM2	1.4	1.7	0.7	2.4	2.5	1.6	2.5	0.5	3.7	2.8
PM3	0.7	1.3	0.6	1.4	1.3	0.7	1.0	0.4	1.2	1.2
PM4	1.8	2.1	0.8	3.2	3.0	1.7	3.1	0.4	9.3	7.0
PM5	0.7	0.8	0.8	0.9	1.0	0.6	0.7	0.9	1.0	0.8
PM6	1.1	1.1	0.9	1.1	1.0	1.3	1.5	0.6	1.2	1.1
PM7	0.7	0.6	0.9	0.8	0.6	0.6	0.3	0.8	0.1	0.1
PM8	1.1	1.0	0.8	0.8	0.9	1.6	1.4	0.5	1.2	1.3
SHP1	1.1	1.3	0.7	2.9	3.0	1.0	1.8	0.5	5.6	4.4
SHP2	1.1	1.2	0.6	2.3	2.4	1.0	1.9	0.5	3.8	3.1
Mnmer1*	0.4	0.2	0.3	0.7	0.5	0.2	0.0	0.0	0.0	0.0
Mnmer2	1.0	0.8	0.8	2.7	2.8	1.0	0.8	0.8	0.1	0.8
Cm1	0.3	0.9	0.5	0.9	0.8	2.0	3.2	0.6		1.6
Cm2	0.3	0.6	0.4	1.7	1.9	0.2	1.6	0.6	3.8	4.4
WT	1.0	1.0	1.0	1.0	1.0	1.0	1.0	1.0	1.0	1.0
MHC Ctrl	0.0	0.0	0.0	0.1	0.1	0.0	0.0	0.0	0.2	0.1

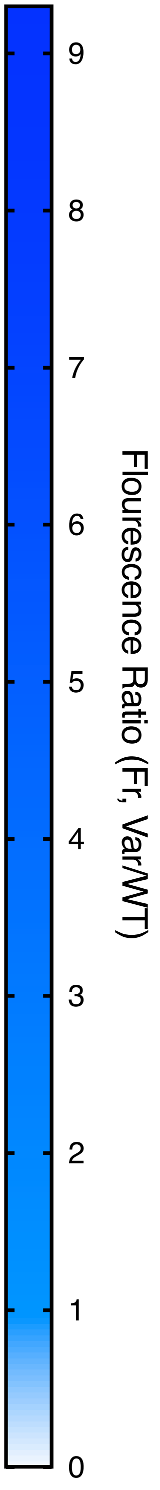

SFig. 1. Antibody binding data of mammalian surface displayed DENV2 sE variants at RT and 40˚C

Flow cytometry data of cells displaying DENV2 sE variant proteins bound to cMyc, 1C19, 3H5, 2D22, EDE1 C8 and EDE1 C10 antibodies, represented by geometric mean fluorescence intensity ratio (Fr) of DENV2 sE variant to DENV2 sE WT displayed cells. Fr data for cells stained at RT (23˚C, left) or 40˚C (right) are presented. Fr values > 1 indicate improved antibody binding to DENV2 sE variants, while Fr values ≈ 1 and <1 represent similar and reduced antibody binding, respectively. Black squares indicate antibody binding was not tested for those sE variants. Asterisks indicate DENV2 sE variants (IntFc6 and Mnmer1) were unfolded after incubation at 40˚C evidenced by no observed binding of the DENV antibodies.

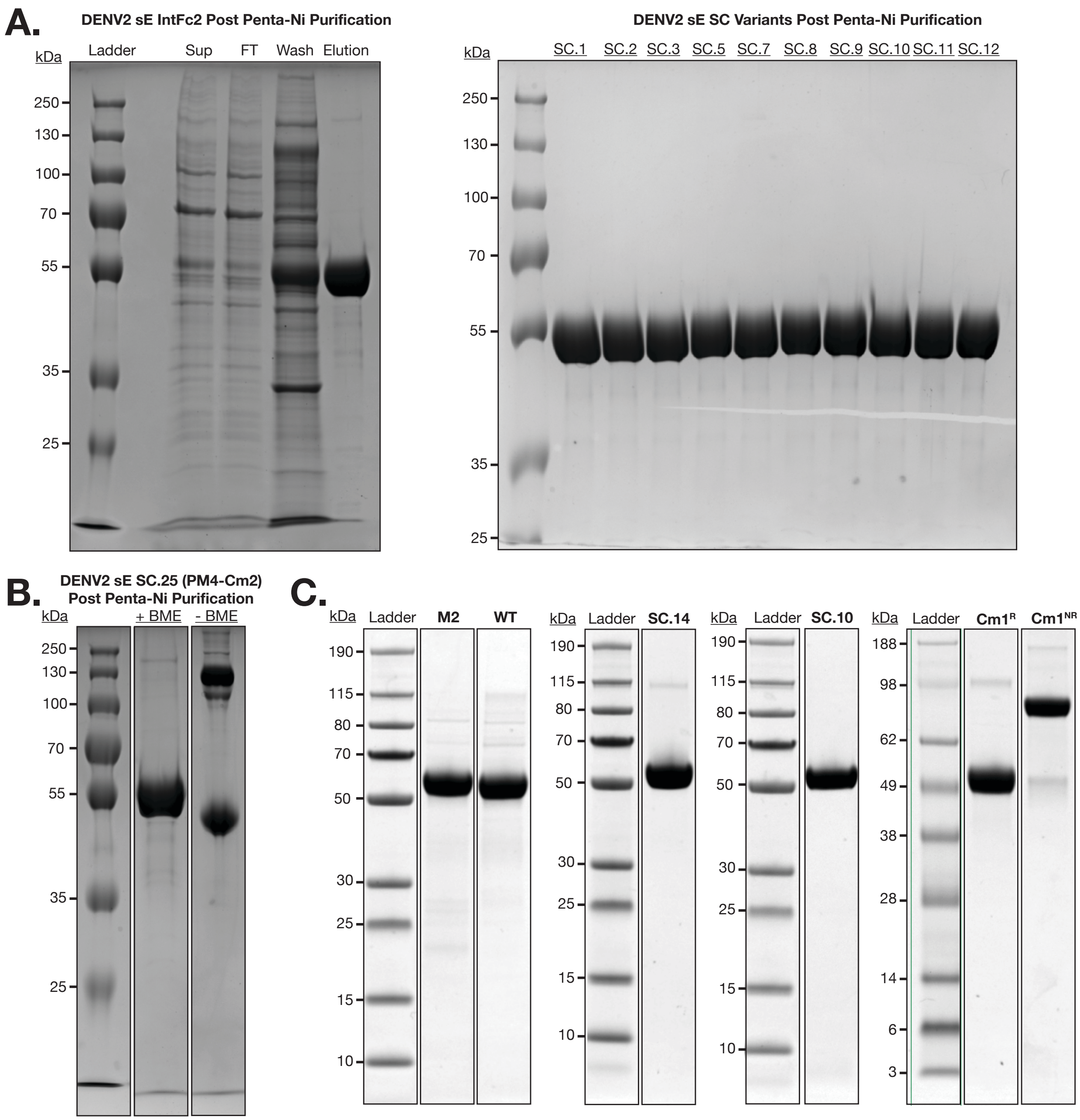

SFig. 2. SDS-PAGE analysis of DENV2 sE proteins used for biophysical characterization, crystallography and mouse immunizations

Coomassie blue stained SDS-PAGE analysis of DENV2 sE proteins post small scale expression and 3 step affinity penta-Ni resin purification from supernatant (A,B) or large-scale expression and purification using a coupled cobalt-resin affinity and size exclusion

chromatography purification protocol (C). A) Purification of DENV2 sE IntFc2, representing the small scale (60-120mL) soluble expression and direct capture of 8x his-tagged DENV2 sE variants with penta-Ni resin (Marvelgent) directly from EXPI293 culture supernatant. Purification samples, including supernatant (Sup), flow through (FT) combined wash (W) and elution (E), were reduced with β-mercaptoethanol (BME) and boiled for 10min at 95˚C then analyzed via SDS-PAGE with 5μg of eluted protein loaded to determine purity and level of host-cell contaminants prior to stability analysis. B) Reduced SDS-PAGE analysis of representative elution fractions of stable combination variants (SC) affinity purified directly from supernatant. 5μg of each protein were loaded to assess purity prior to stability analysis. C) Reduced and non-reduced SDS-PAGE gel of DENV2 sE SC.25, containing PM4 and Cm2 loaded with 3μg per lane. Non-reduced analysis of SC.25 reveals protein band migrating between the 100 and 130kDa markers consistent with the presence of covalent dimers through formation of the L107/A313C disulfide and single band at the expected monomer MW upon reduction. C) Reduced SDS-PAGE analysis of 5μg of DENV2 sE WT, Mnmer2, SC.14 and SC.10 protein used for mouse immunization experiments and x-ray crystallography (SC.10).

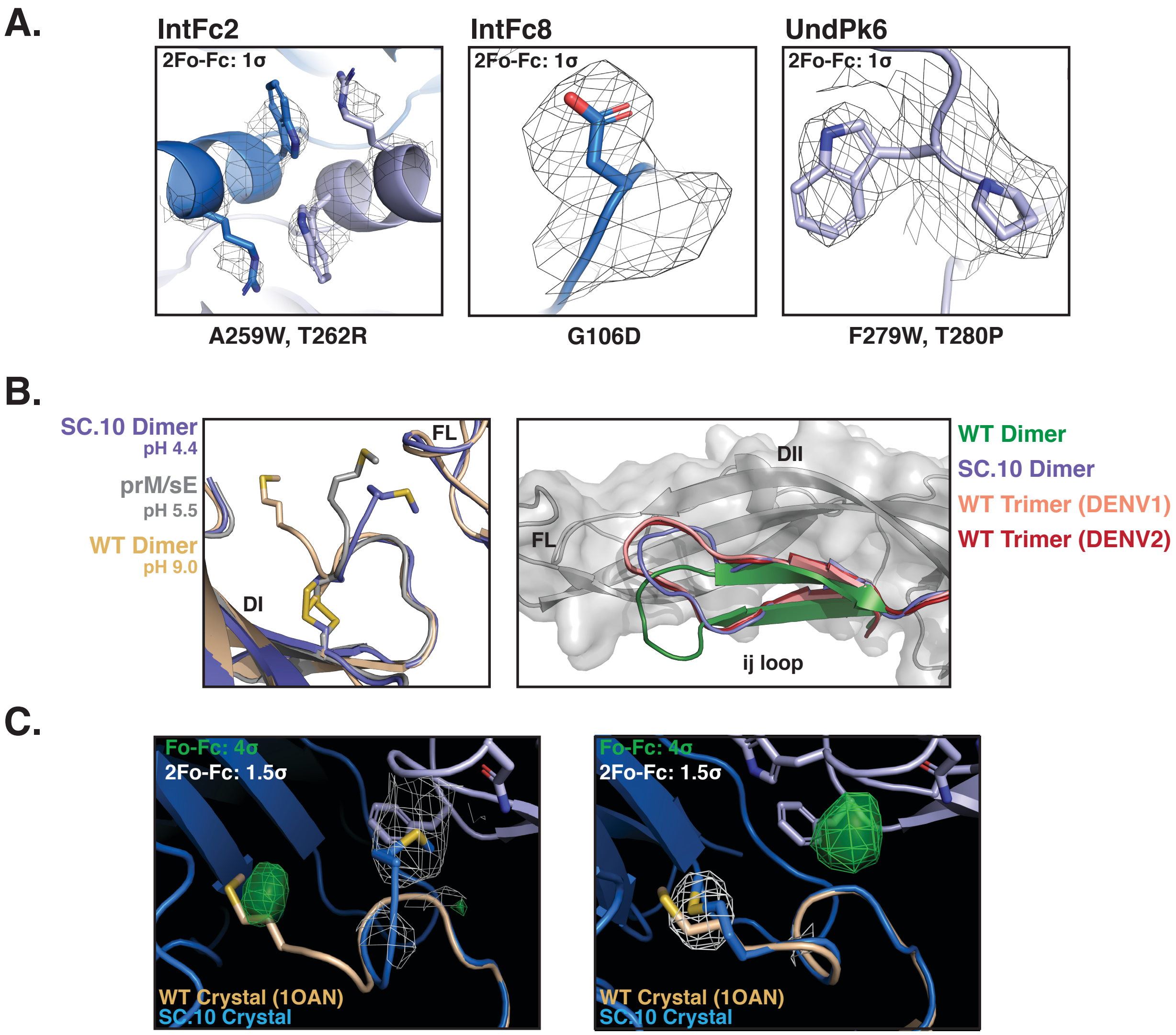

SFig. 3. DENV2 sE SC.10 mutation electron density and low pH effected regions

A) 2Fo-Fc difference electron density map at 1 sigma for DENV2 sE SC.10 IntFc2, UndPk6 and IntFc8 mutations. B) (Left) Alignment of the DENV2 sE N-terminus conformations observed in the crystal structure of SC.10 (purple) solved at pH 4.4, prM/sE co-crystal structure solved at pH 5.5 (gray, PDB 3C5X) and DENV2 sE (tan, PDB 1OAN) solved at pH 9.0. (Right) Alignment of sE ij loop conformation of SC.10 (purple) to WT dimer (green, PDB 1oan) DENV1 sE postfusion trimer (pink, PDB 4GSX), solved under similar conditions as SC.10, and DENV2 sE postfusion trimer (red, PDB 1OK8). C) (Left)

A region of strong electron density, observed at > 4σ in the Fo-Fc and >1.5σ in the 2Fo-Fc maps, present near the N-terminus in DI, was not satisfied by our deposited SC.10 model. Lower R values (both free and work) are observed by modeling the N-terminal methionine (M1) at the first position in the SC.10 N-terminus into the density present near the FL, suggesting the formation of a methionine-aromatic interaction between M1 and F108 and interactions of the M1 sulfur atom with the fusion loop backbone amides, which similar interactions have been observed in other native proteins and are considered favorable interactions (Rao Mundlapati et al., 2015; Valley et al., 2012). However, when aligning of PDB 1oan DI to SC.10 DI, the N-terminal methionine in the canonical DENV sE N-terminus conformation overlaps with the unsatisfied positive density observed in our deposited SC.10 model. (Right) Modeling the SC.10 N-terminal methionine and N-terminus to match the N-terminus observed in 1OAN satisfies the unmodeled positive density observed in our deposited model (left), however, the R values increase by ~1% and the previously satisfied positive density near the FL reappears. Modeling the N-terminus as two alternative conformations does not satisfy either densities. Taken together, the authors deposited the model with a new N-terminus conformation as the model satisfied the density and produced lower statistical R-values.

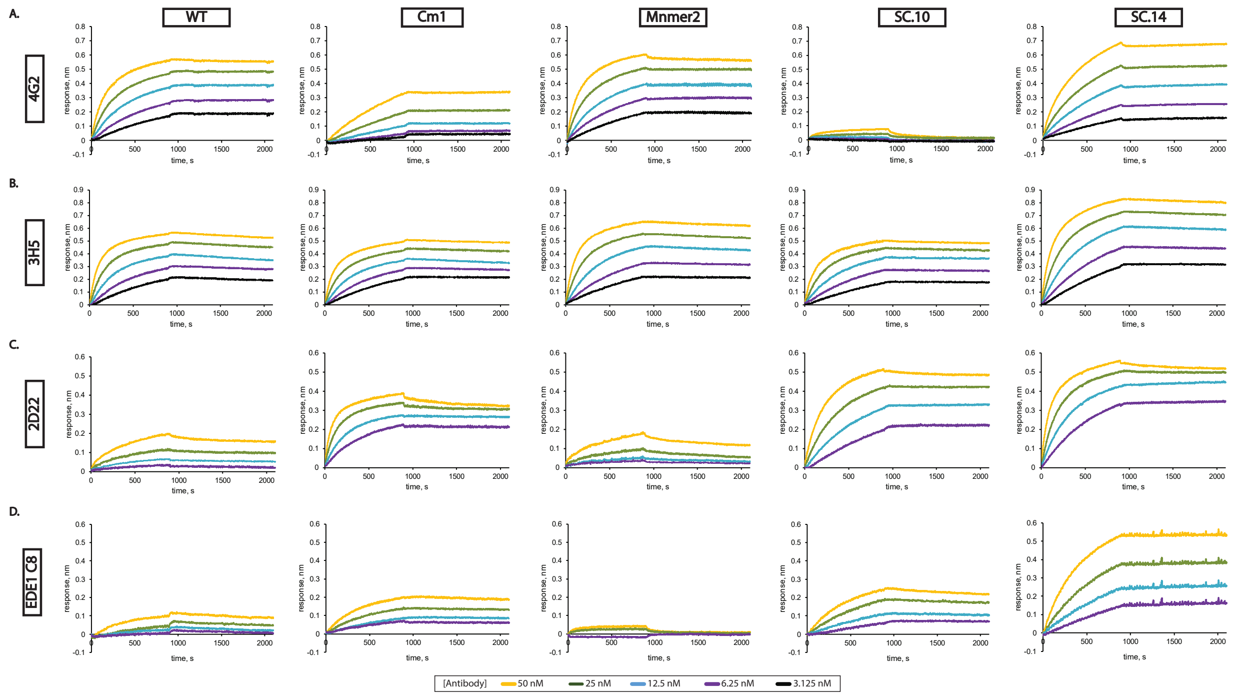

SFig. 4 Binding between DENV2 sE variants and monomer or quaternary epitope antibodies at 37˚C using biolayer interferometry. 10 nM sE was loaded on Ni-NTA sensors and incubated with 3.125 – 50 nM monomer epitope antibodies 4G2 (panel A) and 3H5 (panel B) or 6.25 – 50 nM quaternary epitope antibodies 2D22 (panel C) and EDE1 C8 (panel D).

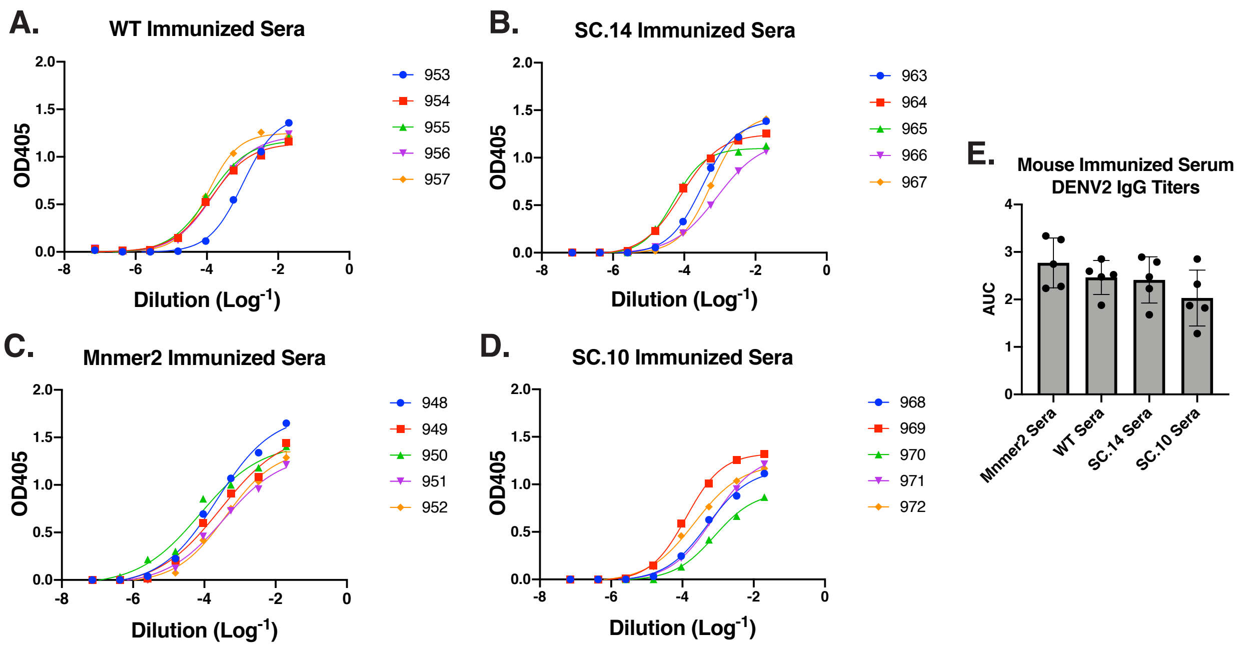

SFig. 5. DENV2 IgG Titers of Non-adjuvanted DENV2 sE WT, Mnmer2, SC.14 and SC.10 mice immunized sera

Sera of mice immunized with DENV2 sE WT (A), SC.14 (B), Mnmer2 (C) and SC.10 (D) were titrated against DENV2 via capture ELISA. Mouse IgG bound to DENV2 was detected using AP-conjugated anti-mouse IgG measuring substrate turnover absorbance at 405nm. E) Plotted are the absolute area under the curve (AUC) values obtained from non-linear regression fit to the serially diluted sera ELISA data for each individual mouse immunized sera and mean for each group (n=5).

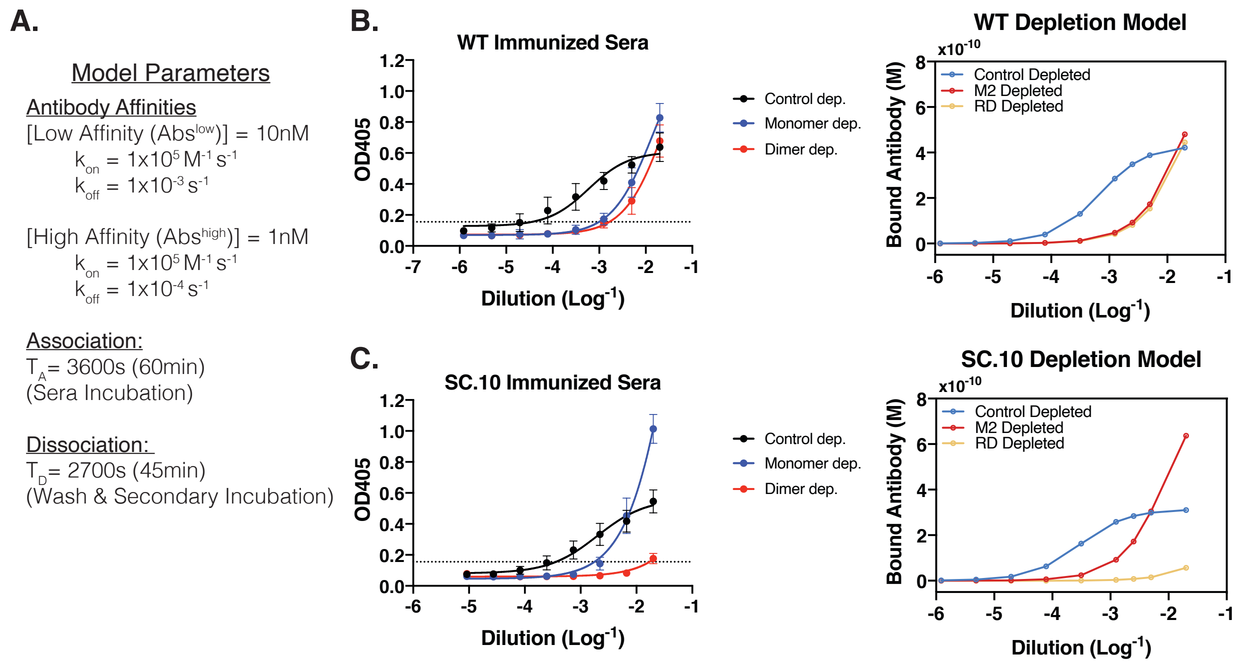

SFig. 6. Comparison of antibody competition model with sE monomer and dimer depleted DENV2 ELISA data

The observed increase in sE monomer depleted sera signal against DENV2 compared to undepleted sera, suggests competition between low affinity and high affinity antibodies elicited by DENV2 sE SC.10 may be occurring. A) We generated a mathematical model (Matlab code provided below figure legend) describing competitive binding of two antibody populations modeling the binding kinetics of both antibody populations, similar to the models used to obtain binding kinetic parameters in protein binding experiments such as SPR and BLI. Using commonly observed antibody-antigen affinities, both low and high low affinity antibody populations were assumed to have the same binding on-rates (k_{on}), with differences in K_d due to different off-rates (k_{off}). The ELISA serum incubation and combined wash and secondary incubation times were used to model the association and dissociation times, T_A and T_D respectively. B and C) Plotted DENV2

ELISA analysis of DENV2 sE WT (B) and SC.10 (C) undepleted and depleted mice immunized sera (left) data qualitatively matches the simulated data from the competition mathematical model (right). This lends support to the hypothesis that low affinity antibodies, specific to the sE monomer, are elicited by DENV2 sE SC.10 and outcompete binding of elicited, and lower abundant, high affinity dimer-specific antibodies to DENV2. Reduced concentration of the low affinity antibodies by depletion with sE monomer, favors binding of the high affinity antibodies to DENV2 in the ELISA, allowing for increase in signal, relative to undepleted sera, at the lowest sera dilutions, that is rapidly lost upon dilution due to low concentrations of the high affinity antibodies.

Matlab script for antibody competition model:

```
% all concentrations in M, time in seconds
Kd1 = 10e-9;          % Kd weak antibodies (M)
kon1 = 1e5;           % on rate for weak antibodies(M-1 s-1)
koff1 = Kd1 * kon1;    % off rate for weak antibody (s-1)

Kd2 = 1e-9;          % Kd strong antibodies
kon2 = 1e5;           % on rate for strong antibodies
koff2 = Kd2 * kon2;    % off rate for strong antibody

epitope = 3e-9;      % concentration of antibody epitopes on "plate"

tmix = 3600;              % time for mixing antibody with virus (seconds)
tspan = [0 900 tmix];     % used for simulating binding to plate

% concentrations of antibodies in serum
% experiment 1: undepleted
% experiment 2: M2 depleted (weak antibodies removed)
% experiment 3: dimer depleted (weak and strong antibodies removed)
nexp = 3;
ab1_in_sera = [5e-5 1e-7 1e-7];    % exp[1,2,3]
ab2_in_sera = [0.6e-7 0.6e-7 0.3e-8];

%ab1_in_sera = [1e-5 1e-7 1e-7];    % exp[1,2,3]
%ab2_in_sera = [4e-7 1e-7 1e-8];

% dilution series for ELISA
dilution = [50 150 500 1500 5000 15000 50000 150000];
ndil = length(dilution);
```

```
% wash time for ELISA
time_wash = 2700;    %seconds

ab_bound = zeros(2,nexp,ndil);         % for storing output
tot_ab_bound = zeros(nexp,ndil);

for j = 1:nexp            % experiments
    for i = 1 : ndil

        ab1 = ab1_in_sera(j) / dilution(i);
        ab2 = ab2_in_sera(j) / dilution(i);

        % ab binding to Elisa plate
        y0 = [ab1 ab2 epitope 0.0 0.0];  % [ab1, ab2, epitope, ab1_bound,
ab2_bound]
        opts = odeset('AbsTol',1e-13);
        [t,y] = ode45(@(t,y) odefcn(t,y,kon1,koff1,kon2,koff2), tspan, y0,
opts);

        % ab being washed off Elisa plate
        ab_bound(1,j,i) = y(3,4)*exp(-koff1*time_wash);  %y("2",x) is time =
tmix
        ab_bound(2,j,i) = y(3,5)*exp(-koff2*time_wash);
        tot_ab_bound(j,i) = ab_bound(1,j,i) + ab_bound(2,j,i);
    end
end

xs = -log10(dilution);  % x_axis fpr plottingex
plot(xs,tot_ab_bound(1,:),'-o',xs,tot_ab_bound(2,:),'-
s',xs,tot_ab_bound(3,:),'-d','Linewidth',2);
xlabel('Dilution Log-1','fontsize',16);
ylabel('Bound antibody','fontsize',16);
axis([-5.5 -1.5 -0.1e-9 0.8e-9])
lgd=legend('no depletion','weak antibodies depleted ("M2")', 'most antibodies
depleted ("RD")');
lgd.FontSize = 16;


function dydt = odefcn(t,y, kon1, koff1, kon2, koff2)
dydt = zeros(5,1);
dydt(1) = -kon1*y(1)*y(3) + koff1*y(4);   % ab1
dydt(2) = -kon2*y(2)*y(3) + koff2*y(5);    % ab2
dydt(3) = dydt(1) + dydt(2);              % epitope
dydt(4) = -dydt(1);                       % ab1_bound
dydt(5) = -dydt(2);                       % ab2_bound
end
```

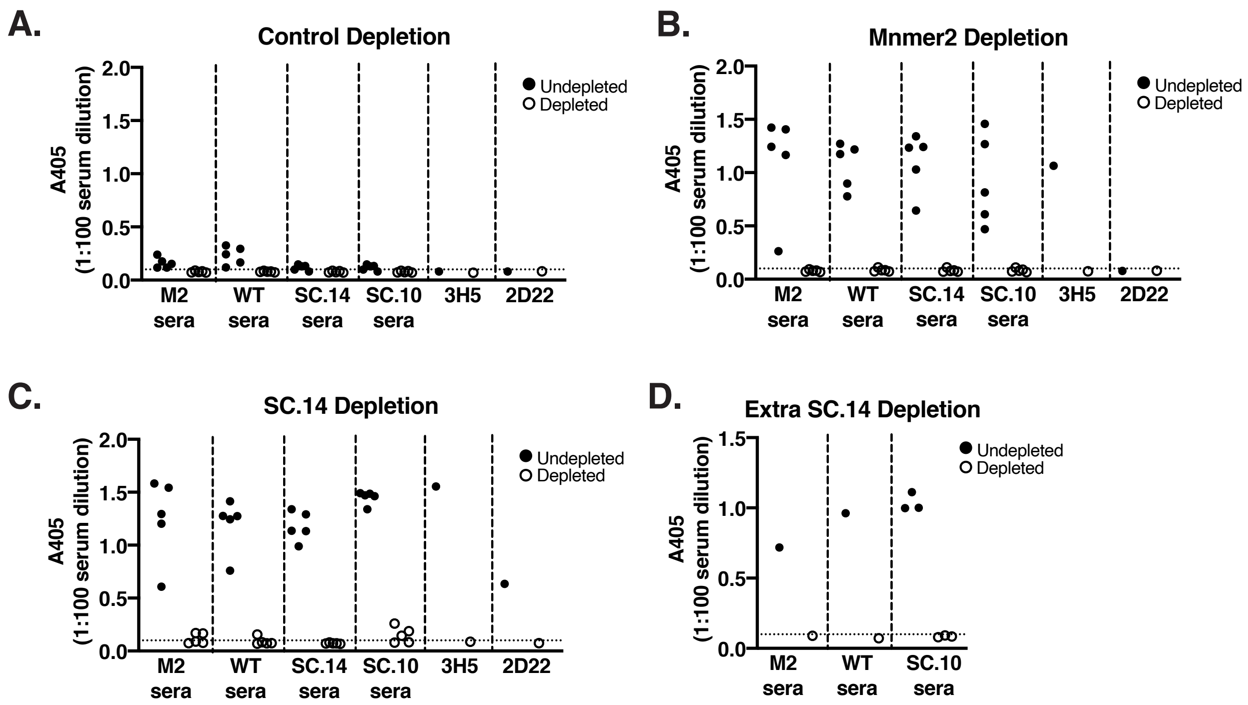

SFig. 7. Validation of complete depletion of Non-adjuvanted DENV2 sE WT, Mnmer2, SC.14 and SC.10 immunized mice sera

Two round depleted mice immunized sera (hollow dot) using His-tagged Spycatcher (A, control depletion), DENV2 sE Mnmer2 (M2) (B) and SC.14 (C-D) were diluted 1:100 and assessed for loss of signal compared to undepleted sera (solid black dot) using a direct ELISA against the homologous antigen used for depletion. D) Observed incomplete depletion of M2, WT and SC.10 sera after two-rounds of depletion using SC.14 warranted a third round of depletion using SC.14. Plotted are the signals of 1:100 diluted depleted third round sera to SC.14 in comparison to the corresponding undepleted sera.

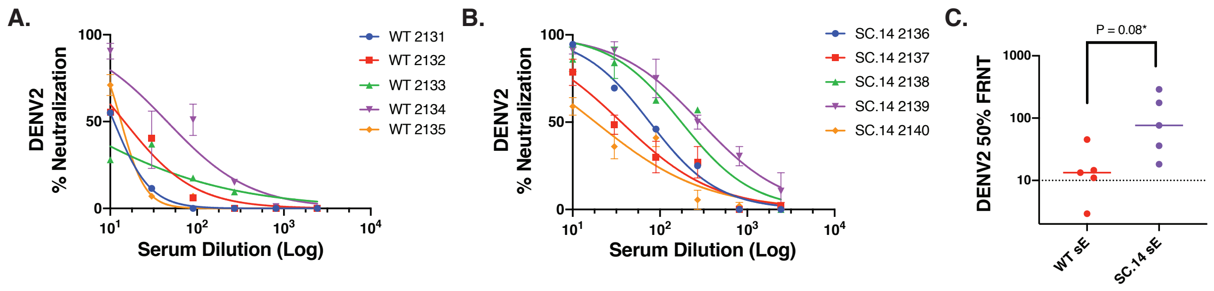

SFig. 8. DENV2 neutralizing antibody levels in mice immunized with WT sE and SC.14 stable dimer variant with Adjuvant. Groups of 5 mice were primed and boosted at three weeks with WT sE (A) or SC.14 (B) using Alhydrogel as an adjuvant. All the mice were bled 6 weeks after the boost for antibody testing. To measure virus neutralizing antibodies, each serum sample was serially diluted and tested in duplicate in a DENV2 focus reduction neutralization assay (A, B). The 50% DENV2 neutralizing antibody titers were greater in mice immunized with the SC.14 sE antigen compared to titers in animals immunized with the WT sE antigen (C). The means of the antibody responses induced by the antigens were compared by unpaired T-test.

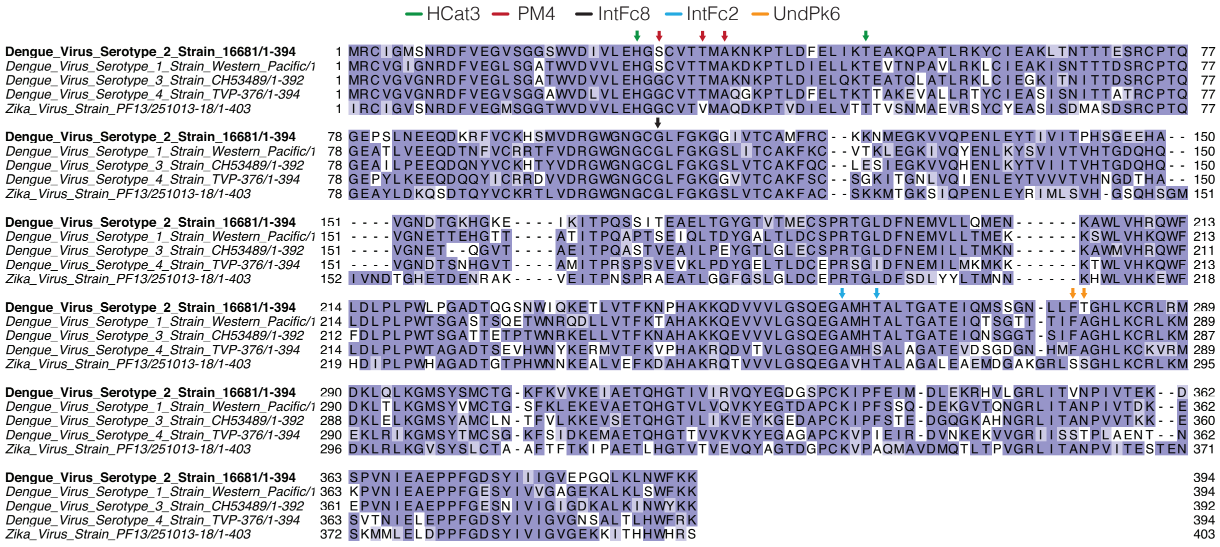

SFig. 9. Multiple sequence alignment for sE from the four DENV serotypes and Zika virus. The strains used for the alignment are indicated in the row titles.

	DENV2 sE Variant Information				NanoDSF Analysis: Melting Point Analysis					NanoDSF Analysis: Dimer Dissociation (T_m^1) Van't Hoff Analysis						Expression Analysis	
	Name	Region	Mutations	# of Mutations	Concentration (μM)	T_m^1 (°C) (Dimer Dissociation)	ΔT_m^1 (°C) (Var - WT)	T_m^2 (°C) (Protein Unfolding)	ΔT_m^2 (°C) (Var - WT)	R^2	Kd (nM) (at 37°C)	ΔH°_{bind} (kcal/mol)	$T\Delta S^\circ_{bind}$ (kcal/mol*K) (at 37°C)	ΔG°_{bind} (kcal/mol)	$\Delta\Delta G^\circ_{bind}$ (kcal/mol) (Var-WT)	Yield (mg/L)	Yield Fold Change (Var/WT)
Reference	WT	-	-	-	8.0	35.9	-	50.3	-								
					4.0	34.2	-	51.0	-	0.994	12958.6 (16000, 10000)	-74.1	-67.1	-6.9	-	0.2	-
					2.0	32.1	-	52.0	-								
Rosetta Design Variants	HCat1 (H1)	DI/DIII Interface	R9V, T32V, H144Y, E368I	4													
	HCat2 (H2)	DI/DIII Interface	R2M, E44M	2	8.0	33.3	-4.1	52.2	2.0							0.3	1.5
	HCat3 (H3)	DI	H27P, T48I	2	4.0	34.0	-0.2	55.1	4.1							1.0	4.3
	HCat4 (H4)	Dimer Region 2	R2M, E44L, D154L, K246I	4	8.0	35.9	0.0	53.8	3.6							0.2	0.9
	HCat5 (H5)	Dimer Region 2	R2M, H27P, E44M, T48I, D154M, H244Q, K246Y	7													
	HCat6 (H6)	Dimer Region 3	K204F, W206F, H261L	3													
	HCat7 (H7)	Dimer Region 3	K204F, V251F, H261F	3													
	HCat8 (H8)	Dimer Region 3	K204F, H261L	2												B.D	B.D
	IntFc1 (I1)	Dimer Region 3	A259V, T262H	2	4.0	38.4	4.2	50.2	-0.8	0.998	2042.7 (2600, 1600)	-93.9	-85.8	-8.1	-1.1	0.2	0.9
	IntFc2 (I2)	Dimer Region 3	A259W, T262R	2	4.0	45.6	11.4	52.9	1.8	0.943	22.7 (800, 0.6) §	-121.0	-110.2	-10.8	-4.6	0.9	3.8
	IntFc3 (I3)	Dimer Region 3	A259V, T262R, A263W	3	4.0	43.4	9.2	51.6	0.6	0.958	191.0 (3800, 10) §	-96.2	-86.7	-9.5	-2.6	0.3	1.3
	IntFc4 (I4)	Dimer Region 3	A259W, T262Y, A263L	3													
	IntFc5 (I5)	Dimer Region 3	T262R, A263W	2	4.0	40.0	5.8	51.3	0.3							0.3	1.1
	IntFc6 (I6)	Dimer Region 2	N8L, S29Y, H244F	3												N.D	N.D
	IntFc7 (I7)	Dimer Region 3	T262R	1	4.0	38.8	4.7	51.3	0.3	0.974	2162.7 (5200, 900)	-68.9	-60.8	-8.0	-1.1	0.5	1.9
	IntFc8 (I8)	Dimer Region 1	G106D	1	4.0	42.9	8.7	51.8	0.8	0.948	219.3 (2500, 20) §	-103.5	-94.1	-9.4	-2.5	4.6	19.1
	PM1 (P1)	Dimer Region 1	M6L, Q316M	2	4.0	28.5	-5.6	49.9	-1.1							0.1	0.3
	PM2 (P2)	DI/DIII Interface & DI/DII Hinge	Q131I, Y299L	2												0.1	0.5
	PM3 (P3)	DI	A35M, M289W	2	4.0	36.5	2.4	47.6	-3.4							0.1	0.4
	PM4 (P4)	DI	S29K, T33V, A35M	3	4.0	34.5	0.3	62.4	11.4	0.989	11506.7 (16000, 8300)	-72.5	-65.5	-7.0	-0.1	2.5	10.5
	PM4.1	DI	A35M	1	4.0	35.0	0.8	52.5	1.5	0.995	9350.8 (14000, 6300)	-79.8	-72.7	-7.1	-0.2	1.3	5.3
	PM4.2	DI	T33V, A35M	2	4.0	34.5	0.4	57.9	6.9	0.997	10845.3 (14000, 8400)	-78.5	-71.4	-7.0	-0.1	1.8	7.5
	PM4.3	DI	S29K	1	4.0	34.9	0.8	55.6	4.6	0.998	9567.7 (12000, 7600)	-81.0	-73.8	-7.1	-0.2	0.8	3.4
	PM5 (P5)	DII	H209D, G266W	2	4.0	36.9	2.7	50.9	-0.1							0.1	0.4
	PM6 (P6)	DIII	D375L, N390Q	2													
	PM7 (P7)	DIII	G330A	1													
	PM8 (P8)	DIII	Y377V, K393R	2													
	UndPk1 (U1)	DI/DII Hinge	I270V, T280A	2													
	UndPk2 (U2)	DI/DII Hinge	L198W	1													
	UndPk3 (U3)	DI/DII Hinge	L191Y, H209D	2													
	UndPk4 (U4)	DI/DIII Interface	E13F, G14A, M34L	3	4.0	37.3	3.1	50.1	-0.9							0.1	0.5
	UndPk5 (U5)	DI/DIII Interface	T359Y	1	4.0	36.2	2.0	51.0	-0.1							0.2	1.0
	UndPk6 (U6)	DI/DII Hinge	F279W, T280P	2	4.0	42.9	8.7	56.9	5.9	0.998	238.2 (360, 160)	-94.0	-84.6	-9.4	-2.5	5.4	22.5
	UndPk6.1	DI/DII Hinge	T280P	1	4.0	40.5	6.4	53.6	2.6	0.989	590 (2300, 150) §	-103.1	-94.3	-8.8	-1.9	1.5	6.3
	UndPk7 (U7)	DI/DII Hinge	T48I, L277M	2	4.0	31.0	-1.1	52.8	0.8							1.0	4.3
	UndPk8 (U8)	Dimer Region 3	Q256Y, G258A, A259W, M260L	4	4.0	31.2	-4.7	53.9	3.6							0.2	0.9
	SHP1 (S1)	DI & DIII	V15E, F373D, F392R	3	4.0	35.1	0.9	51.9	0.9							0.5	2.0
	SHP2 (S2)	DI & DIII	V15E, W20H, F373D, F392R	4													
	Mnmer1 (M1)	Dimer Region 3	S255E	1													
	Mnmer2 (M2)	Dimer Region 3	G258E	1	4.0	N/A	N/A	51.2	0.2	N/A	N/A	N/A	N/A	N/A	N/A	0.2	0.7
Disulfide	Cm1[a]	Dimer Region 3	A259C	1	4.0	N/A	N/A	51.0	0.0	N/A	N/A	N/A	N/A	N/A	N/A	<0.05†	-
	Cm2[b]	Dimer Region 1	L107C, A313C	2												<0.05†	-
Rosetta Design Stable Combination (SC) Variants	SC.1	DR 1 & 3, DI	I2-I8-P4	6	4.0	52.4	18.2	62.5	11.5	0.990	<0.1	-150.3	-135.6	-14.7	-7.8	12.5	51.8
	SC.2	DR 1 & 3, DI	I2-I8-P4-H3	8	4.0	52.1	17.9	65.2	14.2	0.996	<0.1	-149.0	-134.5	-14.5	-7.6	13.5	56.0
	SC.3	DR 1 & 3, DI/DIII, DI/DII, DII	I2-I8-U4-U5-U6-P5	11	4.0	53.8	19.7	59.0	8.0	0.993	<0.1	-323.1	-298.8	-24.3	-17.4	7.4	30.8
	SC.4	DR 1 & 3, DI/DIII, DI/DII, DI, DII	I2-I8-U4-U5-U6-P4-P5-H3	13	4.0	56.7	22.5	65.5	14.4	0.959	<0.1	-208.8	-188.8	-20.0	-13.1	12.2	50.7
	SC.5	DR 1 & 3, DI/DIII, DI/DII	I2-I8-U4-U6	8	4.0	54.0	19.8	59.4	8.4	0.998	<0.1	-299.7	-276.5	-23.2	-16.3	7.5	31.1
	SC.6	DR 1 & 3, DI/DIII, DI	I2-I8-U5-P4	7	4.0	52.3	18.2	63.7	12.7	0.995	0.2 (2, 0.02) §	-133.2	-119.3	-13.9	-7.0	11.7	48.7
	SC.7	DR 1 & 3, DI/DIII, DI/DII	I2-I8-U5-U6	6	4.0	54.2	20.1	59.3	8.3	0.986	<0.1	-416.2	-386.6	-29.6	-22.7	5.8	24.2
	SC.8	DR 1 & 3, DI/DIII, DI/DII, DI	I2-I8-U5-U6-P4	9	4.0	55.6	21.5	66.6	15.6	0.998	<0.1	-156.6	-140.1	-16.5	-9.6	12.8	53.3
	SC.9	DR 1 & 3, DI/DIII, DI/DII, DI, DIII	I2-I8-U5-U6-P4-S1	12	4.0	56.1	22.0	69.3	18.2	0.995	<0.1	-159.0	-142.1	-16.9	-9.9	16.9	70.3
	SC.10	DR 1 & 3, DI/DII	I2-I8-U6	5	4.0	53.9	19.8	59.3	8.3	0.979	<0.1	-428.9	-399.0	-29.9	-23.0	14.3	59.3
	SC.11	DR 1 & 3, DI/DII, DI	I2-I8-U6-H3	7	4.0	56.5	22.3	61.4	10.4	0.815	<0.1	-1060.9	-990.4	-70.5	-63.6	10.8	44.7
	SC.12	DR 1 & 3, DI/DII, DI	I2-I8-U6-P4	8	4.0	55.3	21.2	65.9	14.9	0.995	<0.1	-139.9	-124.5	-15.4	-8.5	10.5	43.6
	SC.13	DR 3, DI/DIII, DI/DII, DI	I2-U5-U6-P4	8	4.0	49.3	15.1	66.4	15.4	0.999	5.4 (10, 3)	-107.2	-95.5	-11.7	-4.8	18.4	76.5
	SC.14	DR 3, DI/DII	I2-U6	4	4.0	50.2	16.1	57.8	6.8	0.941	refer to SC.17 §	-125.9	-113.3	-12.6	-5.7	11.4	47.3
	SC.15	DR 1, DI/DII	I8-U6	3	4.0	50.6	16.4	57.2	6.2	0.989	<0.1	-307.4	-286.9	-20.5	-13.5	13.4	55.7
	SC.16	DR 3, DI/DII, DI	I2-U6-H3	6	4.0	53.8	19.7	60.1	9.1	0.978	<0.1	-1004.0	-944.6	-59.4	-52.5	10.1	41.8
	SC.17	DR 3, DI/DII, DI	I2-U6-P4	7	4.0	48.7	14.5	66.4	15.4	0.998	11.8 (30, 5)	-99.5	-88.2	-11.2	-4.3	15.1	62.8
	SC.18	DR 1 & 3, DI/DII, DI/DIII, DI	I2-I8-U4-U6-P4	11	4.0	54.2	20.1	64.5	13.5	0.990	<0.1	-119.8	-105.9	-13.9	-7.0	12.1	50.3
	SC.19	DR 1, DI/DII, DI/DIII, DI	I8-U5-U6-P4	7	4.0	49.8	15.6	66.6	15.6	0.997	1.8 (6, 0.5)	-120.7	-108.3	-12.4	-5.5	14.9	62.0
	SC.20	DI/DIII, DI	P4-U4	6	4.0	33.4	-0.8	59.5	8.5	0.998	16137.4 (19000, 14000)	-70.6	-63.8	-6.8	0.1	4.5	18.9
	SC.21*	DI/DIII, DI/DII	U4-U5-U6	6	4.0	43.1	8.9	56.7	5.6	0.997	242.4 (400, 150)	-91.3	-81.9	-9.4	-2.5	5.1	21.3
	SC.22*	DI/DIII, DI/DII, DII	U4-U5-U6-P5	8	4.0	43.1	8.9	55.2	4.2	0.988	46 (240, 9) §	-146.2	-135.8	-10.4	-3.5	3.0	12.4
	SC.23	DR 1 & 3	I2-I8	3	4.0	50.1	15.9	55.1	4.1	0.990	<0.1	-490.9	-463.5	-27.4	-20.5	9.5	39.4
	SC.24	DR 3, DI	M2-H3	3	4.0	N/A	N/A	55.4	4.4	N/A	N/A	N/A	N/A	N/A	N/A	0.9	3.7
	SC.25	DR 3, DI	M2-P4	4	4.0	N/A	N/A	62.6	11.6	N/A	N/A	N/A	N/A	N/A	N/A	1.8	7.4
	SC.26	DR 1 & 3, DI/DII	I3-I8-U6	6	4.0	53.1	18.9	58.7	7.7	0.999	<0.1	-320.5	-297.1	-23.4	-16.5	17.2	71.4
	SC.27	DR 1 & 3, DI/DII, DI	I3-I8-U6-P4	9	4.0	53.6	19.5	65.9	14.9	0.999	<0.1	-131.0	-116.7	-14.3	-7.4	15.6	64.8
	SC.28	DR1, DI	P4-Cm2	5	2.0	N/A	N/A	63.4	11.4	N/A	N/A	N/A	N/A	N/A	N/A	0.8	3.2

Key:
N/A: Not applicable; no melting transition observed and no Van't Hoff fit obtained
gray: not tested
green arrow: > WT
red arrow: < WT
yellow box: ≈ WT
*Melting points obtained from 350nm fluorescence data
§Difficult to obtain accurate measurement due to partial overlap of T_m^1 and T_m^2 transitions
† Yield below limit of detection
K_d values within parenthesis indictate 95% confidence limits
DR 1: Dimer Region 1, FL Dimer Interface
DR 2: Dimer Region 2, DI and ij loop Dimer interface
DR 3: Dimer Region 3, αB Helix Central Dimer Interface
Reference[a]: (Rouvinski et. al 2017, Slon Campos et. al., 2017)
Reference[b]: (Rouvinski et. al 2017)

STable. 1. Master Data Table of DENV2 sE variant, expression and stability data

DENV2 sE SC.10 Crystallography
Data collection and refinement statistics

**Data Collection**	
Wavelength (Å)	1.000
Resolution range	86.58 - 3.42
Higher Resolution shell (Å)	3.69 - 3.42
Space group	P 6 2 2
Unit cell	
a, b, c (Å)	125.8 125.8 142.637
α, β, γ (˚)	90 90 120
Total reflections	152827
Unique reflections	9507 (1901)
Multiplicity	16.1 (16.2)
Completeness (%)	99.8 (99.6)
Mean I/sigma(I)	9.2 (2.2)
Wilson B-factor	92.9
R-merge	0.624 (4.789)
R-meas	0.646 (4.900)
R-pim	0.162 (1.264)
CC1/2	0.982 (0.693)
CC*	1.22 (1.58)
**Structure Determination**	
MR Search Model	1OAN*
**Refinement Statistics**	
R-work (%)	31.8 (44.4)
R-free	33.7(55.2)
No. Protein atoms	3005 / 3019
No. Ligand atoms	14 / 3019
Protein residues	383
Root mean square bond lengths (Å)	0.002
Root mean square bond angles (˚)	0.5
Ramachandran	
Favored (%)	84.7
Allowed (%)	13.98
Outliers (%)	1.32
Average B-factor (Å^2)	99.06
Wilson B-factor (Å^2)	92.9
MolProbity	
Clashscore (percentile)	5.80 (575)
Score (percentile)	2.40

Higher resolution statistics are shown in parentheses. The MolProbity clashscore is the lowest in comparison of POLYGON (Urzhumtseva et al., 2009) selected representative structures (in parenthesis) of similar resolution.

*Successful molecular replacement (MR) using Phaser (McCoy et al., 2007) required input of two independent models, made by segmenting PDB 1OAN Chain A into separate models containing DI-DII (Chain A, res 1-296) and DIII (Chain A, res 296-394).

STable. 2. DENV2 sE SC.10 Crystal Structure Data Processing and Refinement Statistics
