# Supplementary material for: Designed, highly expressing, thermostable dengue virus 2 envelope protein dimers elicit quaternary epitope antibodies
Source: Sci Adv. 2021 Oct 15;7(42):eabg4084. doi: 10.1126/sciadv.abg4084 (PMC8519570; doi:10.1126/sciadv.abg4084)
Supplement: Supplementary file 2 — Data files S1 to S4 [file sciadv.abg4084_data_files_s1_to_s4.zip › sciadv.abg4084_data_file_s4.pdf]

Data File S4. DENV2 (D2) Soluble Envelope (sE) Protein Amino Acid Sequences

Key:
Human Serum Albumin Signal Sequence
Dengue 2 Soluble E gene (DENV2 sE)
cMyc Epitope Tag
MHC Iα Cytoplasmic and Transmembrane Domains
Glycine-Serine Linker
His_{8x} Tag

Rosetta Design Variant Mammalian Display Constructs (Dsp)

pD2sE_Dsp (WT)

DNA
ATGAAGTGGGTAACCTTTATTTCCCTTCTTTTTCTCTTTAGCTCGGCTTATTCCATGA
GGTGTATCGGCATGTCCAACAGGGACTTTGTGGAGGGAGTGAGCGGCGGCAGCT
GGGTGGACATTGTGCTGGAGCATGGAAGCTGCGTGACCACGATGGCGAAAAACAA
GCCCACCCTGGACTTCGAGCTCATCAAGACAGAGGCTAAACAGCCCGCCACCCTC
AGGAAGTACTGCATCGAGGCCAAGCTGACCAACACAACAACCGAGTCCAGATGCC
CTACACAGGGCGAACCCAGCCTCAACGAAGAGCAGGACAAGAGGTTCGTGTGCAA
ACACAGCATGGTGGACAGGGGCTGGGGCAATGGATGCGGACTCTTCGGAAAAGG
CGGCATCGTGACCTGCGCCATGTTCAGGTGTAAAAAGAACATGGAAGGCAAGGTG
GTGCAGCCCGAAAATCTGGAGTATACCATCGTGATTACCCCCCACAGCGGAGAGG
AGCACGCCGTGGGCAATGACACCGGCAAGCACGGCAAAGAGATTAAGATCACCC
CCCAGTCCTCCATTACCGAAGCTGAACTGACAGGCTACGGCACCGTGACAATGGA
GTGTAGCCCCAGGACCGGACTGGATTTCAACGAGATGGTCCTGCTACAGATGGAG
AACAAGGCCTGGCTCGTGCACAGGCAATGGTTTCTGGATCTGCCTCTGCCTTGGC
TGCCTGGCGCCGATACACAGGGCTCCAACTGGATACAGAAAGAGACCCTCGTCAC
CTTCAAGAATCCCCATGCTAAGAAGCAGGACGTGGTGGTGCTGGGCAGCCAAGAA
GGCGCCATGCACACAGCCCTGACCGGAGCTACCGAGATCCAGATGAGCTCCGGC
AACCTGCTGTTCACCGGCCATCTGAAATGTAGGCTGAGGATGGATAAGCTGCAAC
TCAAAGGCATGTCCTACTCCATGTGCACCGGAAAGTTCAAGGTGGTGAAAGAAATC
GCCGAAACACAGCACGGCACCATCGTGATCAGGGTGCAGTATGAGGGCGACGGC
TCCCCCTGTAAGATCCCCTTCGAAATCATGGACCTGGAAAAGAGGCACGTGCTGG
GCAGGCTCATCACCGTGAACCCCATTGTCACAGAGAAAGACTCCCCCGTGAACAT
CGAGGCCGAGCCTCCCTTTGGCGACTCCTACATCATCATTGGCGTGGAGCCCGGA
CAGCTCAAGCTGAACTGGTTCAAGAAGTCTAGAGGCAGCAGCGGCGGCAGCGAA
CAAAAACTCATCTCAGAAGAGGATCTGGGAAGTGGAGGCGGGTCCTCTGTAGGAA
TCATTGCGGGACTTGTTCTGCTCGGGGCCGTAATAACTGGAGCGGTCGTGGCGGC
TGTGATGTGGCGACGGAAAAGCAGTGATCGGAAGGGTGGTTCATATACCCAAGCC
GCTTCTAGCGATAGCGCACAAGGATCAGATGTTTCTCTTACCGCATGTAAAGTA

Protein
MKWVTFISLLFLFSSAYSMRCIGMSNRDFVEGVSGGSWVDIVLEHGSCVTTMAKNKPT
LDFELIKTEAKQPATLRKYCIEAKLTNTTTESRCPTQGEPSLNEEQDKRFVCKHSMVDR
GWGNGCGLFGKGGIVTCAMFRCKKNMEGKVVQPENLEYTIVITPHSGEEHAVGNDTG
KHGKEIKITPQSSITEAELTGYGTVTMECSPRTGLDFNEMVLLQMENKAWLVHRQWFL
DLPLPWLPGADTQGSNWIQKETLVTFKNPHAKKQDVVVLGSQEGAMHTALTGATEIQ
MSSGNLLFTGHLKCRLRMDKLQLKGMSYSMCTGKFKVVKEIAETQHGTIVIRVQYEGD
GSPCKIPFEIMDLEKRHVLGRLITVNPIVTEKDSPVNIEAEPPFGDSYIIIGVEPGQLKLN
WFKKSRGSSGGSEQKLISEEDLGSGGGSSVGIIAGLVLLGAVITGAVVAAVMWRRKSS
DRKGGSYTQAASSDSAQGSDVSLTACKV

pD2sE_Dsp_HCat1

DNA
ATGAAGTGGGTAACCTTTATTTCCCTTCTTTTTCTCTTTAGCTCGGCTTATTCCATGA
GGTGTATCGGCATGTCCAACGTGGACTTTGTGGAGGGAGTGAGCGGCGGCAGCT
GGGTGGACATTGTGCTGGAGCATGGAAGCTGCGTGGTGACGATGGCGAAAAACA
AGCCCACCCTGGACTTCGAGCTCATCAAGACAGAGGCTAAACAGCCCGCCACCCT
CAGGAAGTACTGCATCGAGGCCAAGCTGACCAACACAACAACCGAGTCCAGATGC
CCTACACAGGGCGAACCCAGCCTCAACGAAGAGCAGGACAAGAGGTTCGTGTGC
AAACACAGCATGGTGGACAGGGGCTGGGGCAATGGATGCGGACTCTTCGGAAAA
GGCGGCATCGTGACCTGCGCCATGTTCAGGTGTAAAAAGAACATGGAAGGCAAGG
TGGTGCAGCCCGAAAATCTGGAGTATACCATCGTGATTACCCCCTACAGCGGAGA
GGAGCACGCCGTGGGCAATGACACCGGCAAGCACGGCAAAGAGATTAAGATCAC
CCCCCAGTCCTCCATTACCGAAGCTGAACTGACAGGCTACGGCACCGTGACAATG
GAGTGTAGCCCCAGGACCGGACTGGATTTCAACGAGATGGTCCTGCTACAGATGG
AGAACAAGGCCTGGCTCGTGCACAGGCAATGGTTTCTGGATCTGCCTCTGCCTTG
GCTGCCTGGCGCCGATACACAGGGCTCCAACTGGATACAGAAAGAGACCCTCGTC
ACCTTCAAGAATCCCCATGCTAAGAAGCAGGACGTGGTGGTGCTGGGCAGCCAAG
AAGGCGCCATGCACACAGCCCTGACCGGAGCTACCGAGATCCAGATGAGCTCCG
GCAACCTGCTGTTCACCGGCCATCTGAAATGTAGGCTGAGGATGGATAAGCTGCA
ACTCAAAGGCATGTCCTACTCCATGTGCACCGGAAAGTTCAAGGTGGTGAAAGAAA
TCGCCGAAACACAGCACGGCACCATCGTGATCAGGGTGCAGTATGAGGGCGACG
GCTCCCCCTGTAAGATCCCCTTCGAAATCATGGACCTGGAAAAGAGGCACGTGCT
GGGCAGGCTCATCACCGTGAACCCCATTGTCACAGAGAAAGACTCCCCCGTGAAC
ATCATTGCCGAGCCTCCCTTTGGCGACTCCTACATCATCATTGGCGTGGAGCCCG
GACAGCTCAAGCTGAACTGGTTCAAGAAGTCTAGAGGCAGCAGCGGCGGCAGCG
AACAAAAACTCATCTCAGAAGAGGATCTGGGAAGTGGAGGCGGGTCCTCTGTAGG
AATCATTGCGGGACTTGTTCTGCTCGGGGCCGTAATAACTGGAGCGGTCGTGGCG
GCTGTGATGTGGCGACGGAAAAGCAGTGATCGGAAGGGTGGTTCATATACCCAAG
CCGCTTCTAGCGATAGCGCACAAGGATCAGATGTTTCTCTTACCGCATGTAAAGTA

Protein
MKWVTFISLLFLFSSAYSMRCIGMSNVDFVEGVSGGSWVDIVLEHGSCVVTMAKNKPT
LDFELIKTEAKQPATLRKYCIEAKLTNTTTESRCPTQGEPSLNEEQDKRFVCKHSMVDR
GWGNGCGLFGKGGIVTCAMFRCKKNMEGKVVQPENLEYTIVITPYSGEEHAVGNDTG
KHGKEIKITPQSSITEAELTGYGTVTMECSPRTGLDFNEMVLLQMENKAWLVHRQWFL
DLPLPWLPGADTQGSNWIQKETLVTFKNPHAKKQDVVVLGSQEGAMHTALTGATEIQ
MSSGNLLFTGHLKCRLRMDKLQLKGMSYSMCTGKFKVVKEIAETQHGTIVIRVQYEGD
GSPCKIPFEIMDLEKRHVLGRLITVNPIVTEKDSPVNIIAEPPFGDSYIIIGVEPGQLKLNW
FKKSRGSSGGSEQKLISEEDLGSGGGSSVGIIAGLVLLGAVITGAVVAAVMWRRKSSD
RKGGSYTQAASSDSAQGSDVSLTACKV

pD2sE_Dsp_HCat2

DNA
ATGAAGTGGGTAACCTTTATTTCCCTTCTTTTTCTCTTTAGCTCGGCTTATTCCATGA
TGTGTATCGGCATGTCCAACAGGGACTTTGTGGAGGGAGTGAGCGGCGGCAGCT
GGGTGGACATTGTGCTGGAGCATGGAAGCTGCGTGACCACGATGGCGAAAAACAA
GCCCACCCTGGACTTCATGCTCATCAAGACAGAGGCTAAACAGCCCGCCACCCTC
AGGAAGTACTGCATCGAGGCCAAGCTGACCAACACAACAACCGAGTCCAGATGCC
CTACACAGGGCGAACCCAGCCTCAACGAAGAGCAGGACAAGAGGTTCGTGTGCAA
ACACAGCATGGTGGACAGGGGCTGGGGCAATGGATGCGGACTCTTCGGAAAAGG
CGGCATCGTGACCTGCGCCATGTTCAGGTGTAAAAAGAACATGGAAGGCAAGGTG
GTGCAGCCCGAAAATCTGGAGTATACCATCGTGATTACCCCCCACAGCGGAGAGG
AGCACGCCGTGGGCAATGACACCGGCAAGCACGGCAAAGAGATTAAGATCACCC
CCCAGTCCTCCATTACCGAAGCTGAACTGACAGGCTACGGCACCGTGACAATGGA
GTGTAGCCCCAGGACCGGACTGGATTTCAACGAGATGGTCCTGCTACAGATGGAG
AACAAGGCCTGGCTCGTGCACAGGCAATGGTTTCTGGATCTGCCTCTGCCTTGGC
TGCCTGGCGCCGATACACAGGGCTCCAACTGGATACAGAAAGAGACCCTCGTCAC
CTTCAAGAATCCCCATGCTAAGAAGCAGGACGTGGTGGTGCTGGGCAGCCAAGAA
GGCGCCATGCACACAGCCCTGACCGGAGCTACCGAGATCCAGATGAGCTCCGGC
AACCTGCTGTTCACCGGCCATCTGAAATGTAGGCTGAGGATGGATAAGCTGCAAC
TCAAAGGCATGTCCTACTCCATGTGCACCGGAAAGTTCAAGGTGGTGAAAGAAATC
GCCGAAACACAGCACGGCACCATCGTGATCAGGGTGCAGTATGAGGGCGACGGC
TCCCCCTGTAAGATCCCCTTCGAAATCATGGACCTGGAAAAGAGGCACGTGCTGG
GCAGGCTCATCACCGTGAACCCCATTGTCACAGAGAAAGACTCCCCCGTGAACAT
CGAGGCCGAGCCTCCCTTTGGCGACTCCTACATCATCATTGGCGTGGAGCCCGGA
CAGCTCAAGCTGAACTGGTTCAAGAAGTCTAGAGGCAGCAGCGGCGGCAGCGAA
CAAAAACTCATCTCAGAAGAGGATCTGGGAAGTGGAGGCGGGTCCTCTGTAGGAA
TCATTGCGGGACTTGTTCTGCTCGGGGCCGTAATAACTGGAGCGGTCGTGGCGGC
TGTGATGTGGCGACGGAAAAGCAGTGATCGGAAGGGTGGTTCATATACCCAAGCC
GCTTCTAGCGATAGCGCACAAGGATCAGATGTTTCTCTTACCGCATGTAAAGTA

Protein
MKWVTFISLLFLFSSAYSMMCIGMSNRDFVEGVSGGSWVDIVLEHGSCVTTMAKNKP
TLDFMLIKTEAKQPATLRKYCIEAKLTNTTTESRCPTQGEPSLNEEQDKRFVCKHSMVD
RGWGNGCGLFGKGGIVTCAMFRCKKNMEGKVVQPENLEYTIVITPHSGEEHAVGNDT
GKHGKEIKITPQSSITEAELTGYGTVTMECSPRTGLDFNEMVLLQMENKAWLVHRQWF
LDLPLPWLPGADTQGSNWIQKETLVTFKNPHAKKQDVVVLGSQEGAMHTALTGATEIQ
MSSGNLLFTGHLKCRLRMDKLQLKGMSYSMCTGKFKVVKEIAETQHGTIVIRVQYEGD
GSPCKIPFEIMDLEKRHVLGRLITVNPIVTEKDSPVNIEAEPPFGDSYIIIGVEPGQLKLN
WFKKSRGSSGGSEQKLISEEDLGSGGGSSVGIIAGLVLLGAVITGAVVAAVMWRRKSS
DRKGGSYTQAASSDSAQGSDVSLTACKV

pD2sE_Dsp_HCat3

DNA
ATGAAGTGGGTAACCTTTATTTCCCTTCTTTTTCTCTTTAGCTCGGCTTATTCCATGA
GGTGTATCGGCATGTCCAACAGGGACTTTGTGGAGGGAGTGAGCGGCGGCAGCT
GGGTGGACATTGTGCTGGAGCCCGGAAGCTGCGTGACCACGATGGCGAAAAACA
AGCCCACCCTGGACTTCGAGCTCATCAAGATCGAGGCTAAACAGCCCGCCACCCT
CAGGAAGTACTGCATCGAGGCCAAGCTGACCAACACAACAACCGAGTCCAGATGC
CCTACACAGGGCGAACCCAGCCTCAACGAAGAGCAGGACAAGAGGTTCGTGTGC
AAACACAGCATGGTGGACAGGGGCTGGGGCAATGGATGCGGACTCTTCGGAAAA
GGCGGCATCGTGACCTGCGCCATGTTCAGGTGTAAAAAGAACATGGAAGGCAAGG
TGGTGCAGCCCGAAAATCTGGAGTATACCATCGTGATTACCCCCCACAGCGGAGA
GGAGCACGCCGTGGGCAATGACACCGGCAAGCACGGCAAAGAGATTAAGATCAC
CCCCCAGTCCTCCATTACCGAAGCTGAACTGACAGGCTACGGCACCGTGACAATG
GAGTGTAGCCCCAGGACCGGACTGGATTTCAACGAGATGGTCCTGCTACAGATGG
AGAACAAGGCCTGGCTCGTGCACAGGCAATGGTTTCTGGATCTGCCTCTGCCTTG
GCTGCCTGGCGCCGATACACAGGGCTCCAACTGGATACAGAAAGAGACCCTCGTC
ACCTTCAAGAATCCCCATGCTAAGAAGCAGGACGTGGTGGTGCTGGGCAGCCAAG
AAGGCGCCATGCACACAGCCCTGACCGGAGCTACCGAGATCCAGATGAGCTCCG
GCAACCTGCTGTTCACCGGCCATCTGAAATGTAGGCTGAGGATGGATAAGCTGCA
ACTCAAAGGCATGTCCTACTCCATGTGCACCGGAAAGTTCAAGGTGGTGAAAGAAA
TCGCCGAAACACAGCACGGCACCATCGTGATCAGGGTGCAGTATGAGGGCGACG
GCTCCCCCTGTAAGATCCCCTTCGAAATCATGGACCTGGAAAAGAGGCACGTGCT
GGGCAGGCTCATCACCGTGAACCCCATTGTCACAGAGAAAGACTCCCCCGTGAAC
ATCGAGGCCGAGCCTCCCTTTGGCGACTCCTACATCATCATTGGCGTGGAGCCCG
GACAGCTCAAGCTGAACTGGTTCAAGAAGTCTAGAGGCAGCAGCGGCGGCAGCG
AACAAAAACTCATCTCAGAAGAGGATCTGGGAAGTGGAGGCGGGTCCTCTGTAGG
AATCATTGCGGGACTTGTTCTGCTCGGGGCCGTAATAACTGGAGCGGTCGTGGCG
GCTGTGATGTGGCGACGGAAAAGCAGTGATCGGAAGGGTGGTTCATATACCCAAG
CCGCTTCTAGCGATAGCGCACAAGGATCAGATGTTTCTCTTACCGCATGTAAAGTA

Protein
MKWVTFISLLFLFSSAYSMRCIGMSNRDFVEGVSGGSWVDIVLEPGSCVTTMAKNKPT
LDFELIKIEAKQPATLRKYCIEAKLTNTTTESRCPTQGEPSLNEEQDKRFVCKHSMVDR
GWGNGCGLFGKGGIVTCAMFRCKKNMEGKVVQPENLEYTIVITPHSGEEHAVGNDTG
KHGKEIKITPQSSITEAELTGYGTVTMECSPRTGLDFNEMVLLQMENKAWLVHRQWFL
DLPLPWLPGADTQGSNWIQKETLVTFKNPHAKKQDVVVLGSQEGAMHTALTGATEIQ
MSSGNLLFTGHLKCRLRMDKLQLKGMSYSMCTGKFKVVKEIAETQHGTIVIRVQYEGD
GSPCKIPFEIMDLEKRHVLGRLITVNPIVTEKDSPVNIEAEPPFGDSYIIIGVEPGQLKLN
WFKKSRGSSGGSEQKLISEEDLGSGGGSSVGIIAGLVLLGAVITGAVVAAVMWRRKSS
DRKGGSYTQAASSDSAQGSDVSLTACKV

pD2sE_Dsp_HCat4

DNA
ATGAAGTGGGTAACCTTTATTTCCCTTCTTTTTCTCTTTAGCTCGGCTTATTCCATGA
TGTGTATCGGCATGTCCAACAGGGACTTTGTGGAGGGAGTGAGCGGCGGCAGCT
GGGTGGACATTGTGCTGGAGCATGGAAGCTGCGTGACCACGATGGCGAAAAACAA
GCCCACCCTGGACTTCCTGCTCATCAAGACAGAGGCTAAACAGCCCGCCACCCTC
AGGAAGTACTGCATCGAGGCCAAGCTGACCAACACAACAACCGAGTCCAGATGCC
CTACACAGGGCGAACCCAGCCTCAACGAAGAGCAGGACAAGAGGTTCGTGTGCAA
ACACAGCATGGTGGACAGGGGCTGGGGCAATGGATGCGGACTCTTCGGAAAAGG
CGGCATCGTGACCTGCGCCATGTTCAGGTGTAAAAAGAACATGGAAGGCAAGGTG
GTGCAGCCCGAAAATCTGGAGTATACCATCGTGATTACCCCCCACAGCGGAGAGG
AGCACGCCGTGGGCAATCTGACCGGCAAGCACGGCAAAGAGATTAAGATCACCCC
CCAGTCCTCCATTACCGAAGCTGAACTGACAGGCTACGGCACCGTGACAATGGAG
TGTAGCCCCAGGACCGGACTGGATTTCAACGAGATGGTCCTGCTACAGATGGAGA
ACAAGGCCTGGCTCGTGCACAGGCAATGGTTTCTGGATCTGCCTCTGCCTTGGCT
GCCTGGCGCCGATACACAGGGCTCCAACTGGATACAGAAAGAGACCCTCGTCACC
TTCAAGAATCCCCATGCTATCAAGCAGGACGTGGTGGTGCTGGGCAGCCAAGAAG
GCGCCATGCACACAGCCCTGACCGGAGCTACCGAGATCCAGATGAGCTCCGGCA
ACCTGCTGTTCACCGGCCATCTGAAATGTAGGCTGAGGATGGATAAGCTGCAACT
CAAAGGCATGTCCTACTCCATGTGCACCGGAAAGTTCAAGGTGGTGAAAGAAATC
GCCGAAACACAGCACGGCACCATCGTGATCAGGGTGCAGTATGAGGGCGACGGC
TCCCCCTGTAAGATCCCCTTCGAAATCATGGACCTGGAAAAGAGGCACGTGCTGG
GCAGGCTCATCACCGTGAACCCCATTGTCACAGAGAAAGACTCCCCCGTGAACAT
CGAGGCCGAGCCTCCCTTTGGCGACTCCTACATCATCATTGGCGTGGAGCCCGGA
CAGCTCAAGCTGAACTGGTTCAAGAAGTCTAGAGGCAGCAGCGGCGGCAGCGAA
CAAAAACTCATCTCAGAAGAGGATCTGGGAAGTGGAGGCGGGTCCTCTGTAGGAA
TCATTGCGGGACTTGTTCTGCTCGGGGCCGTAATAACTGGAGCGGTCGTGGCGGC
TGTGATGTGGCGACGGAAAAGCAGTGATCGGAAGGGTGGTTCATATACCCAAGCC
GCTTCTAGCGATAGCGCACAAGGATCAGATGTTTCTCTTACCGCATGTAAAGTA

Protein
MKWVTFISLLFLFSSAYSMMCIGMSNRDFVEGVSGGSWVDIVLEHGSCVTTMAKNKP
TLDFLLIKTEAKQPATLRKYCIEAKLTNTTTESRCPTQGEPSLNEEQDKRFVCKHSMVD
RGWGNGCGLFGKGGIVTCAMFRCKKNMEGKVVQPENLEYTIVITPHSGEEHAVGNLT
GKHGKEIKITPQSSITEAELTGYGTVTMECSPRTGLDFNEMVLLQMENKAWLVHRQWF
LDLPLPWLPGADTQGSNWIQKETLVTFKNPHAIKQDVVVLGSQEGAMHTALTGATEIQ
MSSGNLLFTGHLKCRLRMDKLQLKGMSYSMCTGKFKVVKEIAETQHGTIVIRVQYEGD
GSPCKIPFEIMDLEKRHVLGRLITVNPIVTEKDSPVNIEAEPPFGDSYIIIGVEPGQLKLN
WFKKSRGSSGGSEQKLISEEDLGSGGGSSVGIIAGLVLLGAVITGAVVAAVMWRRKSS
DRKGGSYTQAASSDSAQGSDVSLTACKV

pD2sE_Dsp_HCat5

DNA
ATGAAGTGGGTAACCTTTATTTCCCTTCTTTTTCTCTTTAGCTCGGCTTATTCCATGA
TGTGTATCGGCATGTCCAACAGGGACTTTGTGGAGGGAGTGAGCGGCGGCAGCT
GGGTGGACATTGTGCTGGAGCCCGGAAGCTGCGTGACCACGATGGCGAAAAACA
AGCCCACCCTGGACTTCATGCTCATCAAGATCGAGGCTAAACAGCCCGCCACCCT
CAGGAAGTACTGCATCGAGGCCAAGCTGACCAACACAACAACCGAGTCCAGATGC
CCTACACAGGGCGAACCCAGCCTCAACGAAGAGCAGGACAAGAGGTTCGTGTGC
AAACACAGCATGGTGGACAGGGGCTGGGGCAATGGATGCGGACTCTTCGGAAAA
GGCGGCATCGTGACCTGCGCCATGTTCAGGTGTAAAAAGAACATGGAAGGCAAGG
TGGTGCAGCCCGAAAATCTGGAGTATACCATCGTGATTACCCCCCACAGCGGAGA
GGAGCACGCCGTGGGCAATATGACCGGCAAGCACGGCAAAGAGATTAAGATCAC
CCCCCAGTCCTCCATTACCGAAGCTGAACTGACAGGCTACGGCACCGTGACAATG
GAGTGTAGCCCCAGGACCGGACTGGATTTCAACGAGATGGTCCTGCTACAGATGG
AGAACAAGGCCTGGCTCGTGCACAGGCAATGGTTTCTGGATCTGCCTCTGCCTTG
GCTGCCTGGCGCCGATACACAGGGCTCCAACTGGATACAGAAAGAGACCCTCGTC
ACCTTCAAGAATCCCCAGGCTTACAAGCAGGACGTGGTGGTGCTGGGCAGCCAAG
AAGGCGCCATGCACACAGCCCTGACCGGAGCTACCGAGATCCAGATGAGCTCCG
GCAACCTGCTGTTCACCGGCCATCTGAAATGTAGGCTGAGGATGGATAAGCTGCA
ACTCAAAGGCATGTCCTACTCCATGTGCACCGGAAAGTTCAAGGTGGTGAAAGAAA
TCGCCGAAACACAGCACGGCACCATCGTGATCAGGGTGCAGTATGAGGGCGACG
GCTCCCCCTGTAAGATCCCCTTCGAAATCATGGACCTGGAAAAGAGGCACGTGCT
GGGCAGGCTCATCACCGTGAACCCCATTGTCACAGAGAAAGACTCCCCCGTGAAC
ATCGAGGCCGAGCCTCCCTTTGGCGACTCCTACATCATCATTGGCGTGGAGCCCG
GACAGCTCAAGCTGAACTGGTTCAAGAAGTCTAGAGGCAGCAGCGGCGGCAGCG
AACAAAAACTCATCTCAGAAGAGGATCTGGGAAGTGGAGGCGGGTCCTCTGTAGG
AATCATTGCGGGACTTGTTCTGCTCGGGGCCGTAATAACTGGAGCGGTCGTGGCG
GCTGTGATGTGGCGACGGAAAAGCAGTGATCGGAAGGGTGGTTCATATACCCAAG
CCGCTTCTAGCGATAGCGCACAAGGATCAGATGTTTCTCTTACCGCATGTAAAGTA

Protein
MKWVTFISLLFLFSSAYSMMCIGMSNRDFVEGVSGGSWVDIVLEPGSCVTTMAKNKPT
LDFMLIKIEAKQPATLRKYCIEAKLTNTTTESRCPTQGEPSLNEEQDKRFVCKHSMVDR
GWGNGCGLFGKGGIVTCAMFRCKKNMEGKVVQPENLEYTIVITPHSGEEHAVGNMTG
KHGKEIKITPQSSITEAELTGYGTVTMECSPRTGLDFNEMVLLQMENKAWLVHRQWFL
DLPLPWLPGADTQGSNWIQKETLVTFKNPQAYKQDVVVLGSQEGAMHTALTGATEIQ
MSSGNLLFTGHLKCRLRMDKLQLKGMSYSMCTGKFKVVKEIAETQHGTIVIRVQYEGD
GSPCKIPFEIMDLEKRHVLGRLITVNPIVTEKDSPVNIEAEPPFGDSYIIIGVEPGQLKLN
WFKKSRGSSGGSEQKLISEEDLGSGGGSSVGIIAGLVLLGAVITGAVVAAVMWRRKSS
DRKGGSYTQAASSDSAQGSDVSLTACKV

pD2sE_Dsp_HCat6

DNA
ATGAAGTGGGTAACCTTTATTTCCCTTCTTTTTCTCTTTAGCTCGGCTTATTCCATGA
GGTGTATCGGCATGTCCAACAGGGACTTTGTGGAGGGAGTGAGCGGCGGCAGCT
GGGTGGACATTGTGCTGGAGCATGGAAGCTGCGTGACCACGATGGCGAAAAACAA
GCCCACCCTGGACTTCGAGCTCATCAAGACAGAGGCTAAACAGCCCGCCACCCTC
AGGAAGTACTGCATCGAGGCCAAGCTGACCAACACAACAACCGAGTCCAGATGCC
CTACACAGGGCGAACCCAGCCTCAACGAAGAGCAGGACAAGAGGTTCGTGTGCAA
ACACAGCATGGTGGACAGGGGCTGGGGCAATGGATGCGGACTCTTCGGAAAAGG
CGGCATCGTGACCTGCGCCATGTTCAGGTGTAAAAAGAACATGGAAGGCAAGGTG
GTGCAGCCCGAAAATCTGGAGTATACCATCGTGATTACCCCCCACAGCGGAGAGG
AGCACGCCGTGGGCAATGACACCGGCAAGCACGGCAAAGAGATTAAGATCACCC
CCCAGTCCTCCATTACCGAAGCTGAACTGACAGGCTACGGCACCGTGACAATGGA
GTGTAGCCCCAGGACCGGACTGGATTTCAACGAGATGGTCCTGCTACAGATGGAG
AACTTCGCCTTTCTCGTGCACAGGCAATGGTTTCTGGATCTGCCTCTGCCTTGGCT
GCCTGGCGCCGATACACAGGGCTCCAACTGGATACAGAAAGAGACCCTCGTCACC
TTCAAGAATCCCCATGCTAAGAAGCAGGACGTGGTGGTGCTGGGCAGCCAAGAAG
GCGCCATGCTGACAGCCCTGACCGGAGCTACCGAGATCCAGATGAGCTCCGGCA
ACCTGCTGTTCACCGGCCATCTGAAATGTAGGCTGAGGATGGATAAGCTGCAACT
CAAAGGCATGTCCTACTCCATGTGCACCGGAAAGTTCAAGGTGGTGAAAGAAATC
GCCGAAACACAGCACGGCACCATCGTGATCAGGGTGCAGTATGAGGGCGACGGC
TCCCCCTGTAAGATCCCCTTCGAAATCATGGACCTGGAAAAGAGGCACGTGCTGG
GCAGGCTCATCACCGTGAACCCCATTGTCACAGAGAAAGACTCCCCCGTGAACAT
CGAGGCCGAGCCTCCCTTTGGCGACTCCTACATCATCATTGGCGTGGAGCCCGGA
CAGCTCAAGCTGAACTGGTTCAAGAAGTCTAGAGGCAGCAGCGGCGGCAGCGAA
CAAAAACTCATCTCAGAAGAGGATCTGGGAAGTGGAGGCGGGTCCTCTGTAGGAA
TCATTGCGGGACTTGTTCTGCTCGGGGCCGTAATAACTGGAGCGGTCGTGGCGGC
TGTGATGTGGCGACGGAAAAGCAGTGATCGGAAGGGTGGTTCATATACCCAAGCC
GCTTCTAGCGATAGCGCACAAGGATCAGATGTTTCTCTTACCGCATGTAAAGTA

Protein
MKWVTFISLLFLFSSAYSMRCIGMSNRDFVEGVSGGSWVDIVLEHGSCVTTMAKNKPT
LDFELIKTEAKQPATLRKYCIEAKLTNTTTESRCPTQGEPSLNEEQDKRFVCKHSMVDR
GWGNGCGLFGKGGIVTCAMFRCKKNMEGKVVQPENLEYTIVITPHSGEEHAVGNDTG
KHGKEIKITPQSSITEAELTGYGTVTMECSPRTGLDFNEMVLLQMENFAFLVHRQWFL
DLPLPWLPGADTQGSNWIQKETLVTFKNPHAKKQDVVVLGSQEGAMLTALTGATEIQ
MSSGNLLFTGHLKCRLRMDKLQLKGMSYSMCTGKFKVVKEIAETQHGTIVIRVQYEGD
GSPCKIPFEIMDLEKRHVLGRLITVNPIVTEKDSPVNIEAEPPFGDSYIIIGVEPGQLKLN
WFKKSRGSSGGSEQKLISEEDLGSGGGSSVGIIAGLVLLGAVITGAVVAAVMWRRKSS
DRKGGSYTQAASSDSAQGSDVSLTACKV

pD2sE_Dsp_HCat7

DNA
ATGAAGTGGGTAACCTTTATTTCCCTTCTTTTTCTCTTTAGCTCGGCTTATTCCATGA
GGTGTATCGGCATGTCCAACAGGGACTTTGTGGAGGGAGTGAGCGGCGGCAGCT
GGGTGGACATTGTGCTGGAGCATGGAAGCTGCGTGACCACGATGGCGAAAAACAA
GCCCACCCTGGACTTCGAGCTCATCAAGACAGAGGCTAAACAGCCCGCCACCCTC
AGGAAGTACTGCATCGAGGCCAAGCTGACCAACACAACAACCGAGTCCAGATGCC
CTACACAGGGCGAACCCAGCCTCAACGAAGAGCAGGACAAGAGGTTCGTGTGCAA
ACACAGCATGGTGGACAGGGGCTGGGGCAATGGATGCGGACTCTTCGGAAAAGG
CGGCATCGTGACCTGCGCCATGTTCAGGTGTAAAAAGAACATGGAAGGCAAGGTG
GTGCAGCCCGAAAATCTGGAGTATACCATCGTGATTACCCCCCACAGCGGAGAGG
AGCACGCCGTGGGCAATGACACCGGCAAGCACGGCAAAGAGATTAAGATCACCC
CCCAGTCCTCCATTACCGAAGCTGAACTGACAGGCTACGGCACCGTGACAATGGA
GTGTAGCCCCAGGACCGGACTGGATTTCAACGAGATGGTCCTGCTACAGATGGAG
AACTTCGCCTGGCTCGTGCACAGGCAATGGTTTCTGGATCTGCCTCTGCCTTGGCT
GCCTGGCGCCGATACACAGGGCTCCAACTGGATACAGAAAGAGACCCTCGTCACC
TTCAAGAATCCCCATGCTAAGAAGCAGGACGTGTTCGTGCTGGGCAGCCAAGAAG
GCGCCATGTTCACAGCCCTGACCGGAGCTACCGAGATCCAGATGAGCTCCGGCAA
CCTGCTGTTCACCGGCCATCTGAAATGTAGGCTGAGGATGGATAAGCTGCAACTC
AAAGGCATGTCCTACTCCATGTGCACCGGAAAGTTCAAGGTGGTGAAAGAAATCG
CCGAAACACAGCACGGCACCATCGTGATCAGGGTGCAGTATGAGGGCGACGGCT
CCCCCTGTAAGATCCCCTTCGAAATCATGGACCTGGAAAAGAGGCACGTGCTGGG
CAGGCTCATCACCGTGAACCCCATTGTCACAGAGAAAGACTCCCCCGTGAACATC
GAGGCCGAGCCTCCCTTTGGCGACTCCTACATCATCATTGGCGTGGAGCCCGGAC
AGCTCAAGCTGAACTGGTTCAAGAAGTCTAGAGGCAGCAGCGGCGGCAGCGAAC
AAAAACTCATCTCAGAAGAGGATCTGGGAAGTGGAGGCGGGTCCTCTGTAGGAAT
CATTGCGGGACTTGTTCTGCTCGGGGCCGTAATAACTGGAGCGGTCGTGGCGGCT
GTGATGTGGCGACGGAAAAGCAGTGATCGGAAGGGTGGTTCATATACCCAAGCCG
CTTCTAGCGATAGCGCACAAGGATCAGATGTTTCTCTTACCGCATGTAAAGTA

Protein
MKWVTFISLLFLFSSAYSMRCIGMSNRDFVEGVSGGSWVDIVLEHGSCVTTMAKNKPT
LDFELIKTEAKQPATLRKYCIEAKLTNTTTESRCPTQGEPSLNEEQDKRFVCKHSMVDR
GWGNGCGLFGKGGIVTCAMFRCKKNMEGKVVQPENLEYTIVITPHSGEEHAVGNDTG
KHGKEIKITPQSSITEAELTGYGTVTMECSPRTGLDFNEMVLLQMENFAWLVHRQWFL
DLPLPWLPGADTQGSNWIQKETLVTFKNPHAKKQDVFVLGSQEGAMFTALTGATEIQ
MSSGNLLFTGHLKCRLRMDKLQLKGMSYSMCTGKFKVVKEIAETQHGTIVIRVQYEGD
GSPCKIPFEIMDLEKRHVLGRLITVNPIVTEKDSPVNIEAEPPFGDSYIIIGVEPGQLKLN
WFKKSRGSSGGSEQKLISEEDLGSGGGSSVGIIAGLVLLGAVITGAVVAAVMWRRKSS
DRKGGSYTQAASSDSAQGSDVSLTACKV

pD2sE_Dsp_HCat8

DNA
ATGAAGTGGGTAACCTTTATTTCCCTTCTTTTTCTCTTTAGCTCGGCTTATTCCATGA
GGTGTATCGGCATGTCCAACAGGGACTTTGTGGAGGGAGTGAGCGGCGGCAGCT
GGGTGGACATTGTGCTGGAGCATGGAAGCTGCGTGACCACGATGGCGAAAAACAA
GCCCACCCTGGACTTCGAGCTCATCAAGACAGAGGCTAAACAGCCCGCCACCCTC
AGGAAGTACTGCATCGAGGCCAAGCTGACCAACACAACAACCGAGTCCAGATGCC
CTACACAGGGCGAACCCAGCCTCAACGAAGAGCAGGACAAGAGGTTCGTGTGCAA
ACACAGCATGGTGGACAGGGGCTGGGGCAATGGATGCGGACTCTTCGGAAAAGG
CGGCATCGTGACCTGCGCCATGTTCAGGTGTAAAAAGAACATGGAAGGCAAGGTG
GTGCAGCCCGAAAATCTGGAGTATACCATCGTGATTACCCCCCACAGCGGAGAGG
AGCACGCCGTGGGCAATGACACCGGCAAGCACGGCAAAGAGATTAAGATCACCC
CCCAGTCCTCCATTACCGAAGCTGAACTGACAGGCTACGGCACCGTGACAATGGA
GTGTAGCCCCAGGACCGGACTGGATTTCAACGAGATGGTCCTGCTACAGATGGAG
AACTTCGCCTGGCTCGTGCACAGGCAATGGTTTCTGGATCTGCCTCTGCCTTGGCT
GCCTGGCGCCGATACACAGGGCTCCAACTGGATACAGAAAGAGACCCTCGTCACC
TTCAAGAATCCCCATGCTAAGAAGCAGGACGTGGTGGTGCTGGGCAGCCAAGAAG
GCGCCATGCTGACAGCCCTGACCGGAGCTACCGAGATCCAGATGAGCTCCGGCA
ACCTGCTGTTCACCGGCCATCTGAAATGTAGGCTGAGGATGGATAAGCTGCAACT
CAAAGGCATGTCCTACTCCATGTGCACCGGAAAGTTCAAGGTGGTGAAAGAAATC
GCCGAAACACAGCACGGCACCATCGTGATCAGGGTGCAGTATGAGGGCGACGGC
TCCCCCTGTAAGATCCCCTTCGAAATCATGGACCTGGAAAAGAGGCACGTGCTGG
GCAGGCTCATCACCGTGAACCCCATTGTCACAGAGAAAGACTCCCCCGTGAACAT
CGAGGCCGAGCCTCCCTTTGGCGACTCCTACATCATCATTGGCGTGGAGCCCGGA
CAGCTCAAGCTGAACTGGTTCAAGAAGTCTAGAGGCAGCAGCGGCGGCAGCGAA
CAAAAACTCATCTCAGAAGAGGATCTGGGAAGTGGAGGCGGGTCCTCTGTAGGAA
TCATTGCGGGACTTGTTCTGCTCGGGGCCGTAATAACTGGAGCGGTCGTGGCGGC
TGTGATGTGGCGACGGAAAAGCAGTGATCGGAAGGGTGGTTCATATACCCAAGCC
GCTTCTAGCGATAGCGCACAAGGATCAGATGTTTCTCTTACCGCATGTAAAGTA

Protein
MKWVTFISLLFLFSSAYSMRCIGMSNRDFVEGVSGGSWVDIVLEHGSCVTTMAKNKPT
LDFELIKTEAKQPATLRKYCIEAKLTNTTTESRCPTQGEPSLNEEQDKRFVCKHSMVDR
GWGNGCGLFGKGGIVTCAMFRCKKNMEGKVVQPENLEYTIVITPHSGEEHAVGNDTG
KHGKEIKITPQSSITEAELTGYGTVTMECSPRTGLDFNEMVLLQMENFAWLVHRQWFL
DLPLPWLPGADTQGSNWIQKETLVTFKNPHAKKQDVVVLGSQEGAMLTALTGATEIQ
MSSGNLLFTGHLKCRLRMDKLQLKGMSYSMCTGKFKVVKEIAETQHGTIVIRVQYEGD
GSPCKIPFEIMDLEKRHVLGRLITVNPIVTEKDSPVNIEAEPPFGDSYIIIGVEPGQLKLN
WFKKSRGSSGGSEQKLISEEDLGSGGGSSVGIIAGLVLLGAVITGAVVAAVMWRRKSS
DRKGGSYTQAASSDSAQGSDVSLTACKV

pD2sE_Dsp_IntFc1

DNA
ATGAAGTGGGTAACCTTTATTTCCCTTCTTTTTCTCTTTAGCTCGGCTTATTCCATGA
GGTGTATCGGCATGTCCAACAGGGACTTTGTGGAGGGAGTGAGCGGCGGCAGCT
GGGTGGACATTGTGCTGGAGCATGGAAGCTGCGTGACCACGATGGCGAAAAACAA
GCCCACCCTGGACTTCGAGCTCATCAAGACAGAGGCTAAACAGCCCGCCACCCTC
AGGAAGTACTGCATCGAGGCCAAGCTGACCAACACAACAACCGAGTCCAGATGCC
CTACACAGGGCGAACCCAGCCTCAACGAAGAGCAGGACAAGAGGTTCGTGTGCAA
ACACAGCATGGTGGACAGGGGCTGGGGCAATGGATGCGGACTCTTCGGAAAAGG
CGGCATCGTGACCTGCGCCATGTTCAGGTGTAAAAAGAACATGGAAGGCAAGGTG
GTGCAGCCCGAAAATCTGGAGTATACCATCGTGATTACCCCCCACAGCGGAGAGG
AGCACGCCGTGGGCAATGACACCGGCAAGCACGGCAAAGAGATTAAGATCACCC
CCCAGTCCTCCATTACCGAAGCTGAACTGACAGGCTACGGCACCGTGACAATGGA
GTGTAGCCCCAGGACCGGACTGGATTTCAACGAGATGGTCCTGCTACAGATGGAG
AACAAGGCCTGGCTCGTGCACAGGCAATGGTTTCTGGATCTGCCTCTGCCTTGGC
TGCCTGGCGCCGATACACAGGGCTCCAACTGGATACAGAAAGAGACCCTCGTCAC
CTTCAAGAATCCCCATGCTAAGAAGCAGGACGTGGTGGTGCTGGGCAGCCAAGAA
GGCGTGATGCACCACGCCCTGACCGGAGCTACCGAGATCCAGATGAGCTCCGGC
AACCTGCTGTTCACCGGCCATCTGAAATGTAGGCTGAGGATGGATAAGCTGCAAC
TCAAAGGCATGTCCTACTCCATGTGCACCGGAAAGTTCAAGGTGGTGAAAGAAATC
GCCGAAACACAGCACGGCACCATCGTGATCAGGGTGCAGTATGAGGGCGACGGC
TCCCCCTGTAAGATCCCCTTCGAAATCATGGACCTGGAAAAGAGGCACGTGCTGG
GCAGGCTCATCACCGTGAACCCCATTGTCACAGAGAAAGACTCCCCCGTGAACAT
CGAGGCCGAGCCTCCCTTTGGCGACTCCTACATCATCATTGGCGTGGAGCCCGGA
CAGCTCAAGCTGAACTGGTTCAAGAAGTCTAGAGGCAGCAGCGGCGGCAGCGAA
CAAAAACTCATCTCAGAAGAGGATCTGGGAAGTGGAGGCGGGTCCTCTGTAGGAA
TCATTGCGGGACTTGTTCTGCTCGGGGCCGTAATAACTGGAGCGGTCGTGGCGGC
TGTGATGTGGCGACGGAAAAGCAGTGATCGGAAGGGTGGTTCATATACCCAAGCC
GCTTCTAGCGATAGCGCACAAGGATCAGATGTTTCTCTTACCGCATGTAAAGTA

Protein
MKWVTFISLLFLFSSAYSMRCIGMSNRDFVEGVSGGSWVDIVLEHGSCVTTMAKNKPT
LDFELIKTEAKQPATLRKYCIEAKLTNTTTESRCPTQGEPSLNEEQDKRFVCKHSMVDR
GWGNGCGLFGKGGIVTCAMFRCKKNMEGKVVQPENLEYTIVITPHSGEEHAVGNDTG
KHGKEIKITPQSSITEAELTGYGTVTMECSPRTGLDFNEMVLLQMENKAWLVHRQWFL
DLPLPWLPGADTQGSNWIQKETLVTFKNPHAKKQDVVVLGSQEGVMHHALTGATEIQ
MSSGNLLFTGHLKCRLRMDKLQLKGMSYSMCTGKFKVVKEIAETQHGTIVIRVQYEGD
GSPCKIPFEIMDLEKRHVLGRLITVNPIVTEKDSPVNIEAEPPFGDSYIIIGVEPGQLKLN
WFKKSRGSSGGSEQKLISEEDLGSGGGSSVGIIAGLVLLGAVITGAVVAAVMWRRKSS
DRKGGSYTQAASSDSAQGSDVSLTACKV

pD2sE_Dsp_IntFc2

DNA
ATGAAGTGGGTAACCTTTATTTCCCTTCTTTTTCTCTTTAGCTCGGCTTATTCCATGA
GGTGTATCGGCATGTCCAACAGGGACTTTGTGGAGGGAGTGAGCGGCGGCAGCT
GGGTGGACATTGTGCTGGAGCATGGAAGCTGCGTGACCACGATGGCGAAAAACAA
GCCCACCCTGGACTTCGAGCTCATCAAGACAGAGGCTAAACAGCCCGCCACCCTC
AGGAAGTACTGCATCGAGGCCAAGCTGACCAACACAACAACCGAGTCCAGATGCC
CTACACAGGGCGAACCCAGCCTCAACGAAGAGCAGGACAAGAGGTTCGTGTGCAA
ACACAGCATGGTGGACAGGGGCTGGGGCAATGGATGCGGACTCTTCGGAAAAGG
CGGCATCGTGACCTGCGCCATGTTCAGGTGTAAAAAGAACATGGAAGGCAAGGTG
GTGCAGCCCGAAAATCTGGAGTATACCATCGTGATTACCCCCCACAGCGGAGAGG
AGCACGCCGTGGGCAATGACACCGGCAAGCACGGCAAAGAGATTAAGATCACCC
CCCAGTCCTCCATTACCGAAGCTGAACTGACAGGCTACGGCACCGTGACAATGGA
GTGTAGCCCCAGGACCGGACTGGATTTCAACGAGATGGTCCTGCTACAGATGGAG
AACAAGGCCTGGCTCGTGCACAGGCAATGGTTTCTGGATCTGCCTCTGCCTTGGC
TGCCTGGCGCCGATACACAGGGCTCCAACTGGATACAGAAAGAGACCCTCGTCAC
CTTCAAGAATCCCCATGCTAAGAAGCAGGACGTGGTGGTGCTGGGCAGCCAAGAA
GGCTGGATGCACCGGGCCCTGACCGGAGCTACCGAGATCCAGATGAGCTCCGGC
AACCTGCTGTTCACCGGCCATCTGAAATGTAGGCTGAGGATGGATAAGCTGCAAC
TCAAAGGCATGTCCTACTCCATGTGCACCGGAAAGTTCAAGGTGGTGAAAGAAATC
GCCGAAACACAGCACGGCACCATCGTGATCAGGGTGCAGTATGAGGGCGACGGC
TCCCCCTGTAAGATCCCCTTCGAAATCATGGACCTGGAAAAGAGGCACGTGCTGG
GCAGGCTCATCACCGTGAACCCCATTGTCACAGAGAAAGACTCCCCCGTGAACAT
CGAGGCCGAGCCTCCCTTTGGCGACTCCTACATCATCATTGGCGTGGAGCCCGGA
CAGCTCAAGCTGAACTGGTTCAAGAAGTCTAGAGGCAGCAGCGGCGGCAGCGAA
CAAAAACTCATCTCAGAAGAGGATCTGGGAAGTGGAGGCGGGTCCTCTGTAGGAA
TCATTGCGGGACTTGTTCTGCTCGGGGCCGTAATAACTGGAGCGGTCGTGGCGGC
TGTGATGTGGCGACGGAAAAGCAGTGATCGGAAGGGTGGTTCATATACCCAAGCC
GCTTCTAGCGATAGCGCACAAGGATCAGATGTTTCTCTTACCGCATGTAAAGTA

Protein
MKWVTFISLLFLFSSAYSMRCIGMSNRDFVEGVSGGSWVDIVLEHGSCVTTMAKNKPT
LDFELIKTEAKQPATLRKYCIEAKLTNTTTESRCPTQGEPSLNEEQDKRFVCKHSMVDR
GWGNGCGLFGKGGIVTCAMFRCKKNMEGKVVQPENLEYTIVITPHSGEEHAVGNDTG
KHGKEIKITPQSSITEAELTGYGTVTMECSPRTGLDFNEMVLLQMENKAWLVHRQWFL
DLPLPWLPGADTQGSNWIQKETLVTFKNPHAKKQDVVVLGSQEGWMHRALTGATEIQ
MSSGNLLFTGHLKCRLRMDKLQLKGMSYSMCTGKFKVVKEIAETQHGTIVIRVQYEGD
GSPCKIPFEIMDLEKRHVLGRLITVNPIVTEKDSPVNIEAEPPFGDSYIIIGVEPGQLKLN
WFKKSRGSSGGSEQKLISEEDLGSGGGSSVGIIAGLVLLGAVITGAVVAAVMWRRKSS
DRKGGSYTQAASSDSAQGSDVSLTACKV

pD2sE_Dsp_IntFc3

DNA
ATGAAGTGGGTAACCTTTATTTCCCTTCTTTTTCTCTTTAGCTCGGCTTATTCCATGA
GGTGTATCGGCATGTCCAACAGGGACTTTGTGGAGGGAGTGAGCGGCGGCAGCT
GGGTGGACATTGTGCTGGAGCATGGAAGCTGCGTGACCACGATGGCGAAAAACAA
GCCCACCCTGGACTTCGAGCTCATCAAGACAGAGGCTAAACAGCCCGCCACCCTC
AGGAAGTACTGCATCGAGGCCAAGCTGACCAACACAACAACCGAGTCCAGATGCC
CTACACAGGGCGAACCCAGCCTCAACGAAGAGCAGGACAAGAGGTTCGTGTGCAA
ACACAGCATGGTGGACAGGGGCTGGGGCAATGGATGCGGACTCTTCGGAAAAGG
CGGCATCGTGACCTGCGCCATGTTCAGGTGTAAAAAGAACATGGAAGGCAAGGTG
GTGCAGCCCGAAAATCTGGAGTATACCATCGTGATTACCCCCCACAGCGGAGAGG
AGCACGCCGTGGGCAATGACACCGGCAAGCACGGCAAAGAGATTAAGATCACCC
CCCAGTCCTCCATTACCGAAGCTGAACTGACAGGCTACGGCACCGTGACAATGGA
GTGTAGCCCCAGGACCGGACTGGATTTCAACGAGATGGTCCTGCTACAGATGGAG
AACAAGGCCTGGCTCGTGCACAGGCAATGGTTTCTGGATCTGCCTCTGCCTTGGC
TGCCTGGCGCCGATACACAGGGCTCCAACTGGATACAGAAAGAGACCCTCGTCAC
CTTCAAGAATCCCCATGCTAAGAAGCAGGACGTGGTGGTGCTGGGCAGCCAAGAA
GGCGTGATGCACCGGTGGCTGACCGGAGCTACCGAGATCCAGATGAGCTCCGGC
AACCTGCTGTTCACCGGCCATCTGAAATGTAGGCTGAGGATGGATAAGCTGCAAC
TCAAAGGCATGTCCTACTCCATGTGCACCGGAAAGTTCAAGGTGGTGAAAGAAATC
GCCGAAACACAGCACGGCACCATCGTGATCAGGGTGCAGTATGAGGGCGACGGC
TCCCCCTGTAAGATCCCCTTCGAAATCATGGACCTGGAAAAGAGGCACGTGCTGG
GCAGGCTCATCACCGTGAACCCCATTGTCACAGAGAAAGACTCCCCCGTGAACAT
CGAGGCCGAGCCTCCCTTTGGCGACTCCTACATCATCATTGGCGTGGAGCCCGGA
CAGCTCAAGCTGAACTGGTTCAAGAAGTCTAGAGGCAGCAGCGGCGGCAGCGAA
CAAAAACTCATCTCAGAAGAGGATCTGGGAAGTGGAGGCGGGTCCTCTGTAGGAA
TCATTGCGGGACTTGTTCTGCTCGGGGCCGTAATAACTGGAGCGGTCGTGGCGGC
TGTGATGTGGCGACGGAAAAGCAGTGATCGGAAGGGTGGTTCATATACCCAAGCC
GCTTCTAGCGATAGCGCACAAGGATCAGATGTTTCTCTTACCGCATGTAAAGTA

Protein
MKWVTFISLLFLFSSAYSMRCIGMSNRDFVEGVSGGSWVDIVLEHGSCVTTMAKNKPT
LDFELIKTEAKQPATLRKYCIEAKLTNTTTESRCPTQGEPSLNEEQDKRFVCKHSMVDR
GWGNGCGLFGKGGIVTCAMFRCKKNMEGKVVQPENLEYTIVITPHSGEEHAVGNDTG
KHGKEIKITPQSSITEAELTGYGTVTMECSPRTGLDFNEMVLLQMENKAWLVHRQWFL
DLPLPWLPGADTQGSNWIQKETLVTFKNPHAKKQDVVVLGSQEGVMHRWLTGATEIQ
MSSGNLLFTGHLKCRLRMDKLQLKGMSYSMCTGKFKVVKEIAETQHGTIVIRVQYEGD
GSPCKIPFEIMDLEKRHVLGRLITVNPIVTEKDSPVNIEAEPPFGDSYIIIGVEPGQLKLN
WFKKSRGSSGGSEQKLISEEDLGSGGGSSVGIIAGLVLLGAVITGAVVAAVMWRRKSS
DRKGGSYTQAASSDSAQGSDVSLTACKV

pD2sE_Dsp_IntFc4

DNA
ATGAAGTGGGTAACCTTTATTTCCCTTCTTTTTCTCTTTAGCTCGGCTTATTCCATGA
GGTGTATCGGCATGTCCAACAGGGACTTTGTGGAGGGAGTGAGCGGCGGCAGCT
GGGTGGACATTGTGCTGGAGCATGGAAGCTGCGTGACCACGATGGCGAAAAACAA
GCCCACCCTGGACTTCGAGCTCATCAAGACAGAGGCTAAACAGCCCGCCACCCTC
AGGAAGTACTGCATCGAGGCCAAGCTGACCAACACAACAACCGAGTCCAGATGCC
CTACACAGGGCGAACCCAGCCTCAACGAAGAGCAGGACAAGAGGTTCGTGTGCAA
ACACAGCATGGTGGACAGGGGCTGGGGCAATGGATGCGGACTCTTCGGAAAAGG
CGGCATCGTGACCTGCGCCATGTTCAGGTGTAAAAAGAACATGGAAGGCAAGGTG
GTGCAGCCCGAAAATCTGGAGTATACCATCGTGATTACCCCCCACAGCGGAGAGG
AGCACGCCGTGGGCAATGACACCGGCAAGCACGGCAAAGAGATTAAGATCACCC
CCCAGTCCTCCATTACCGAAGCTGAACTGACAGGCTACGGCACCGTGACAATGGA
GTGTAGCCCCAGGACCGGACTGGATTTCAACGAGATGGTCCTGCTACAGATGGAG
AACAAGGCCTGGCTCGTGCACAGGCAATGGTTTCTGGATCTGCCTCTGCCTTGGC
TGCCTGGCGCCGATACACAGGGCTCCAACTGGATACAGAAAGAGACCCTCGTCAC
CTTCAAGAATCCCCATGCTAAGAAGCAGGACGTGGTGGTGCTGGGCAGCCAAGAA
GGCTGGATGCACTACCTCCTGACCGGAGCTACCGAGATCCAGATGAGCTCCGGCA
ACCTGCTGTTCACCGGCCATCTGAAATGTAGGCTGAGGATGGATAAGCTGCAACT
CAAAGGCATGTCCTACTCCATGTGCACCGGAAAGTTCAAGGTGGTGAAAGAAATC
GCCGAAACACAGCACGGCACCATCGTGATCAGGGTGCAGTATGAGGGCGACGGC
TCCCCCTGTAAGATCCCCTTCGAAATCATGGACCTGGAAAAGAGGCACGTGCTGG
GCAGGCTCATCACCGTGAACCCCATTGTCACAGAGAAAGACTCCCCCGTGAACAT
CGAGGCCGAGCCTCCCTTTGGCGACTCCTACATCATCATTGGCGTGGAGCCCGGA
CAGCTCAAGCTGAACTGGTTCAAGAAGTCTAGAGGCAGCAGCGGCGGCAGCGAA
CAAAAACTCATCTCAGAAGAGGATCTGGGAAGTGGAGGCGGGTCCTCTGTAGGAA
TCATTGCGGGACTTGTTCTGCTCGGGGCCGTAATAACTGGAGCGGTCGTGGCGGC
TGTGATGTGGCGACGGAAAAGCAGTGATCGGAAGGGTGGTTCATATACCCAAGCC
GCTTCTAGCGATAGCGCACAAGGATCAGATGTTTCTCTTACCGCATGTAAAGTA

Protein
MKWVTFISLLFLFSSAYSMRCIGMSNRDFVEGVSGGSWVDIVLEHGSCVTTMAKNKPT
LDFELIKTEAKQPATLRKYCIEAKLTNTTTESRCPTQGEPSLNEEQDKRFVCKHSMVDR
GWGNGCGLFGKGGIVTCAMFRCKKNMEGKVVQPENLEYTIVITPHSGEEHAVGNDTG
KHGKEIKITPQSSITEAELTGYGTVTMECSPRTGLDFNEMVLLQMENKAWLVHRQWFL
DLPLPWLPGADTQGSNWIQKETLVTFKNPHAKKQDVVVLGSQEGWMHYLLTGATEIQ
MSSGNLLFTGHLKCRLRMDKLQLKGMSYSMCTGKFKVVKEIAETQHGTIVIRVQYEGD
GSPCKIPFEIMDLEKRHVLGRLITVNPIVTEKDSPVNIEAEPPFGDSYIIIGVEPGQLKLN
WFKKSRGSSGGSEQKLISEEDLGSGGGSSVGIIAGLVLLGAVITGAVVAAVMWRRKSS
DRKGGSYTQAASSDSAQGSDVSLTACKV

pD2sE_Dsp_IntFc5

DNA
ATGAAGTGGGTAACCTTTATTTCCCTTCTTTTTCTCTTTAGCTCGGCTTATTCCATGA
GGTGTATCGGCATGTCCAACAGGGACTTTGTGGAGGGAGTGAGCGGCGGCAGCT
GGGTGGACATTGTGCTGGAGCATGGAAGCTGCGTGACCACGATGGCGAAAAACAA
GCCCACCCTGGACTTCGAGCTCATCAAGACAGAGGCTAAACAGCCCGCCACCCTC
AGGAAGTACTGCATCGAGGCCAAGCTGACCAACACAACAACCGAGTCCAGATGCC
CTACACAGGGCGAACCCAGCCTCAACGAAGAGCAGGACAAGAGGTTCGTGTGCAA
ACACAGCATGGTGGACAGGGGCTGGGGCAATGGATGCGGACTCTTCGGAAAAGG
CGGCATCGTGACCTGCGCCATGTTCAGGTGTAAAAAGAACATGGAAGGCAAGGTG
GTGCAGCCCGAAAATCTGGAGTATACCATCGTGATTACCCCCCACAGCGGAGAGG
AGCACGCCGTGGGCAATGACACCGGCAAGCACGGCAAAGAGATTAAGATCACCC
CCCAGTCCTCCATTACCGAAGCTGAACTGACAGGCTACGGCACCGTGACAATGGA
GTGTAGCCCCAGGACCGGACTGGATTTCAACGAGATGGTCCTGCTACAGATGGAG
AACAAGGCCTGGCTCGTGCACAGGCAATGGTTTCTGGATCTGCCTCTGCCTTGGC
TGCCTGGCGCCGATACACAGGGCTCCAACTGGATACAGAAAGAGACCCTCGTCAC
CTTCAAGAATCCCCATGCTAAGAAGCAGGACGTGGTGGTGCTGGGCAGCCAAGAA
GGCGCCATGCACCGGTGGCTGACCGGAGCTACCGAGATCCAGATGAGCTCCGGC
AACCTGCTGTTCACCGGCCATCTGAAATGTAGGCTGAGGATGGATAAGCTGCAAC
TCAAAGGCATGTCCTACTCCATGTGCACCGGAAAGTTCAAGGTGGTGAAAGAAATC
GCCGAAACACAGCACGGCACCATCGTGATCAGGGTGCAGTATGAGGGCGACGGC
TCCCCCTGTAAGATCCCCTTCGAAATCATGGACCTGGAAAAGAGGCACGTGCTGG
GCAGGCTCATCACCGTGAACCCCATTGTCACAGAGAAAGACTCCCCCGTGAACAT
CGAGGCCGAGCCTCCCTTTGGCGACTCCTACATCATCATTGGCGTGGAGCCCGGA
CAGCTCAAGCTGAACTGGTTCAAGAAGTCTAGAGGCAGCAGCGGCGGCAGCGAA
CAAAAACTCATCTCAGAAGAGGATCTGGGAAGTGGAGGCGGGTCCTCTGTAGGAA
TCATTGCGGGACTTGTTCTGCTCGGGGCCGTAATAACTGGAGCGGTCGTGGCGGC
TGTGATGTGGCGACGGAAAAGCAGTGATCGGAAGGGTGGTTCATATACCCAAGCC
GCTTCTAGCGATAGCGCACAAGGATCAGATGTTTCTCTTACCGCATGTAAAGTA

Protein
MKWVTFISLLFLFSSAYSMRCIGMSNRDFVEGVSGGSWVDIVLEHGSCVTTMAKNKPT
LDFELIKTEAKQPATLRKYCIEAKLTNTTTESRCPTQGEPSLNEEQDKRFVCKHSMVDR
GWGNGCGLFGKGGIVTCAMFRCKKNMEGKVVQPENLEYTIVITPHSGEEHAVGNDTG
KHGKEIKITPQSSITEAELTGYGTVTMECSPRTGLDFNEMVLLQMENKAWLVHRQWFL
DLPLPWLPGADTQGSNWIQKETLVTFKNPHAKKQDVVVLGSQEGAMHRWLTGATEIQ
MSSGNLLFTGHLKCRLRMDKLQLKGMSYSMCTGKFKVVKEIAETQHGTIVIRVQYEGD
GSPCKIPFEIMDLEKRHVLGRLITVNPIVTEKDSPVNIEAEPPFGDSYIIIGVEPGQLKLN
WFKKSRGSSGGSEQKLISEEDLGSGGGSSVGIIAGLVLLGAVITGAVVAAVMWRRKSS
DRKGGSYTQAASSDSAQGSDVSLTACKV

pD2sE_Dsp_IntFc6

DNA
ATGAAGTGGGTAACCTTTATTTCCCTTCTTTTTCTCTTTAGCTCGGCTTATTCCATGA
GGTGTATCGGCATGTCCCTGAGGGACTTTGTGGAGGGAGTGAGCGGCGGCAGCT
GGGTGGACATTGTGCTGGAGCATGGATACTGCGTGACCACGATGGCGAAAAACAA
GCCCACCCTGGACTTCGAGCTCATCAAGACAGAGGCTAAACAGCCCGCCACCCTC
AGGAAGTACTGCATCGAGGCCAAGCTGACCAACACAACAACCGAGTCCAGATGCC
CTACACAGGGCGAACCCAGCCTCAACGAAGAGCAGGACAAGAGGTTCGTGTGCAA
ACACAGCATGGTGGACAGGGGCTGGGGCAATGGATGCGGACTCTTCGGAAAAGG
CGGCATCGTGACCTGCGCCATGTTCAGGTGTAAAAAGAACATGGAAGGCAAGGTG
GTGCAGCCCGAAAATCTGGAGTATACCATCGTGATTACCCCCCACAGCGGAGAGG
AGCACGCCGTGGGCAATGACACCGGCAAGCACGGCAAAGAGATTAAGATCACCC
CCCAGTCCTCCATTACCGAAGCTGAACTGACAGGCTACGGCACCGTGACAATGGA
GTGTAGCCCCAGGACCGGACTGGATTTCAACGAGATGGTCCTGCTACAGATGGAG
AACAAGGCCTGGCTCGTGCACAGGCAATGGTTTCTGGATCTGCCTCTGCCTTGGC
TGCCTGGCGCCGATACACAGGGCTCCAACTGGATACAGAAAGAGACCCTCGTCAC
CTTCAAGAATCCCTTCGCTAAGAAGCAGGACGTGGTGGTGCTGGGCAGCCAAGAA
GGCGCCATGCACACAGCCCTGACCGGAGCTACCGAGATCCAGATGAGCTCCGGC
AACCTGCTGTTCACCGGCCATCTGAAATGTAGGCTGAGGATGGATAAGCTGCAAC
TCAAAGGCATGTCCTACTCCATGTGCACCGGAAAGTTCAAGGTGGTGAAAGAAATC
GCCGAAACACAGCACGGCACCATCGTGATCAGGGTGCAGTATGAGGGCGACGGC
TCCCCCTGTAAGATCCCCTTCGAAATCATGGACCTGGAAAAGAGGCACGTGCTGG
GCAGGCTCATCACCGTGAACCCCATTGTCACAGAGAAAGACTCCCCCGTGAACAT
CGAGGCCGAGCCTCCCTTTGGCGACTCCTACATCATCATTGGCGTGGAGCCCGGA
CAGCTCAAGCTGAACTGGTTCAAGAAGTCTAGAGGCAGCAGCGGCGGCAGCGAA
CAAAAACTCATCTCAGAAGAGGATCTGGGAAGTGGAGGCGGGTCCTCTGTAGGAA
TCATTGCGGGACTTGTTCTGCTCGGGGCCGTAATAACTGGAGCGGTCGTGGCGGC
TGTGATGTGGCGACGGAAAAGCAGTGATCGGAAGGGTGGTTCATATACCCAAGCC
GCTTCTAGCGATAGCGCACAAGGATCAGATGTTTCTCTTACCGCATGTAAAGTA

Protein
MKWVTFISLLFLFSSAYSMRCIGMSLRDFVEGVSGGSWVDIVLEHGYCVTTMAKNKPT
LDFELIKTEAKQPATLRKYCIEAKLTNTTTESRCPTQGEPSLNEEQDKRFVCKHSMVDR
GWGNGCGLFGKGGIVTCAMFRCKKNMEGKVVQPENLEYTIVITPHSGEEHAVGNDTG
KHGKEIKITPQSSITEAELTGYGTVTMECSPRTGLDFNEMVLLQMENKAWLVHRQWFL
DLPLPWLPGADTQGSNWIQKETLVTFKNPFAKKQDVVVLGSQEGAMHTALTGATEIQ
MSSGNLLFTGHLKCRLRMDKLQLKGMSYSMCTGKFKVVKEIAETQHGTIVIRVQYEGD
GSPCKIPFEIMDLEKRHVLGRLITVNPIVTEKDSPVNIEAEPPFGDSYIIIGVEPGQLKLN
WFKKSRGSSGGSEQKLISEEDLGSGGGSSVGIIAGLVLLGAVITGAVVAAVMWRRKSS
DRKGGSYTQAASSDSAQGSDVSLTACKV

pD2sE_Dsp_IntFc7

DNA
ATGAAGTGGGTAACCTTTATTTCCCTTCTTTTTCTCTTTAGCTCGGCTTATTCCATGA
GGTGTATCGGCATGTCCAACAGGGACTTTGTGGAGGGAGTGAGCGGCGGCAGCT
GGGTGGACATTGTGCTGGAGCATGGAAGCTGCGTGACCACGATGGCGAAAAACAA
GCCCACCCTGGACTTCGAGCTCATCAAGACAGAGGCTAAACAGCCCGCCACCCTC
AGGAAGTACTGCATCGAGGCCAAGCTGACCAACACAACAACCGAGTCCAGATGCC
CTACACAGGGCGAACCCAGCCTCAACGAAGAGCAGGACAAGAGGTTCGTGTGCAA
ACACAGCATGGTGGACAGGGGCTGGGGCAATGGATGCGGACTCTTCGGAAAAGG
CGGCATCGTGACCTGCGCCATGTTCAGGTGTAAAAAGAACATGGAAGGCAAGGTG
GTGCAGCCCGAAAATCTGGAGTATACCATCGTGATTACCCCCCACAGCGGAGAGG
AGCACGCCGTGGGCAATGACACCGGCAAGCACGGCAAAGAGATTAAGATCACCC
CCCAGTCCTCCATTACCGAAGCTGAACTGACAGGCTACGGCACCGTGACAATGGA
GTGTAGCCCCAGGACCGGACTGGATTTCAACGAGATGGTCCTGCTACAGATGGAG
AACAAGGCCTGGCTCGTGCACAGGCAATGGTTTCTGGATCTGCCTCTGCCTTGGC
TGCCTGGCGCCGATACACAGGGCTCCAACTGGATACAGAAAGAGACCCTCGTCAC
CTTCAAGAATCCCCATGCTAAGAAGCAGGACGTGGTGGTGCTGGGCAGCCAAGAA
GGCGCCATGCACCGGGCCCTGACCGGAGCTACCGAGATCCAGATGAGCTCCGGC
AACCTGCTGTTCACCGGCCATCTGAAATGTAGGCTGAGGATGGATAAGCTGCAAC
TCAAAGGCATGTCCTACTCCATGTGCACCGGAAAGTTCAAGGTGGTGAAAGAAATC
GCCGAAACACAGCACGGCACCATCGTGATCAGGGTGCAGTATGAGGGCGACGGC
TCCCCCTGTAAGATCCCCTTCGAAATCATGGACCTGGAAAAGAGGCACGTGCTGG
GCAGGCTCATCACCGTGAACCCCATTGTCACAGAGAAAGACTCCCCCGTGAACAT
CGAGGCCGAGCCTCCCTTTGGCGACTCCTACATCATCATTGGCGTGGAGCCCGGA
CAGCTCAAGCTGAACTGGTTCAAGAAGTCTAGAGGCAGCAGCGGCGGCAGCGAA
CAAAAACTCATCTCAGAAGAGGATCTGGGAAGTGGAGGCGGGTCCTCTGTAGGAA
TCATTGCGGGACTTGTTCTGCTCGGGGCCGTAATAACTGGAGCGGTCGTGGCGGC
TGTGATGTGGCGACGGAAAAGCAGTGATCGGAAGGGTGGTTCATATACCCAAGCC
GCTTCTAGCGATAGCGCACAAGGATCAGATGTTTCTCTTACCGCATGTAAAGTA

Protein
MKWVTFISLLFLFSSAYSMRCIGMSNRDFVEGVSGGSWVDIVLEHGSCVTTMAKNKPT
LDFELIKTEAKQPATLRKYCIEAKLTNTTTESRCPTQGEPSLNEEQDKRFVCKHSMVDR
GWGNGCGLFGKGGIVTCAMFRCKKNMEGKVVQPENLEYTIVITPHSGEEHAVGNDTG
KHGKEIKITPQSSITEAELTGYGTVTMECSPRTGLDFNEMVLLQMENKAWLVHRQWFL
DLPLPWLPGADTQGSNWIQKETLVTFKNPHAKKQDVVVLGSQEGAMHRALTGATEIQ
MSSGNLLFTGHLKCRLRMDKLQLKGMSYSMCTGKFKVVKEIAETQHGTIVIRVQYEGD
GSPCKIPFEIMDLEKRHVLGRLITVNPIVTEKDSPVNIEAEPPFGDSYIIIGVEPGQLKLN
WFKKSRGSSGGSEQKLISEEDLGSGGGSSVGIIAGLVLLGAVITGAVVAAVMWRRKSS
DRKGGSYTQAASSDSAQGSDVSLTACKV

pD2sE_Dsp_IntFc8

DNA
ATGAAGTGGGTAACCTTTATTTCCCTTCTTTTTCTCTTTAGCTCGGCTTATTCCATGA
GGTGTATCGGCATGTCCAACAGGGACTTTGTGGAGGGAGTGAGCGGCGGCAGCT
GGGTGGACATTGTGCTGGAGCATGGAAGCTGCGTGACCACGATGGCGAAAAACAA
GCCCACCCTGGACTTCGAGCTCATCAAGACAGAGGCTAAACAGCCCGCCACCCTC
AGGAAGTACTGCATCGAGGCCAAGCTGACCAACACAACAACCGAGTCCAGATGCC
CTACACAGGGCGAACCCAGCCTCAACGAAGAGCAGGACAAGAGGTTCGTGTGCAA
ACACAGCATGGTGGACAGGGGCTGGGGCAATGGATGCGACCTCTTCGGAAAAGG
CGGCATCGTGACCTGCGCCATGTTCAGGTGTAAAAAGAACATGGAAGGCAAGGTG
GTGCAGCCCGAAAATCTGGAGTATACCATCGTGATTACCCCCCACAGCGGAGAGG
AGCACGCCGTGGGCAATGACACCGGCAAGCACGGCAAAGAGATTAAGATCACCC
CCCAGTCCTCCATTACCGAAGCTGAACTGACAGGCTACGGCACCGTGACAATGGA
GTGTAGCCCCAGGACCGGACTGGATTTCAACGAGATGGTCCTGCTACAGATGGAG
AACAAGGCCTGGCTCGTGCACAGGCAATGGTTTCTGGATCTGCCTCTGCCTTGGC
TGCCTGGCGCCGATACACAGGGCTCCAACTGGATACAGAAAGAGACCCTCGTCAC
CTTCAAGAATCCCCATGCTAAGAAGCAGGACGTGGTGGTGCTGGGCAGCCAAGAA
GGCGCCATGCACACAGCCCTGACCGGAGCTACCGAGATCCAGATGAGCTCCGGC
AACCTGCTGTTCACCGGCCATCTGAAATGTAGGCTGAGGATGGATAAGCTGCAAC
TCAAAGGCATGTCCTACTCCATGTGCACCGGAAAGTTCAAGGTGGTGAAAGAAATC
GCCGAAACACAGCACGGCACCATCGTGATCAGGGTGCAGTATGAGGGCGACGGC
TCCCCCTGTAAGATCCCCTTCGAAATCATGGACCTGGAAAAGAGGCACGTGCTGG
GCAGGCTCATCACCGTGAACCCCATTGTCACAGAGAAAGACTCCCCCGTGAACAT
CGAGGCCGAGCCTCCCTTTGGCGACTCCTACATCATCATTGGCGTGGAGCCCGGA
CAGCTCAAGCTGAACTGGTTCAAGAAGTCTAGAGGCAGCAGCGGCGGCAGCGAA
CAAAAACTCATCTCAGAAGAGGATCTGGGAAGTGGAGGCGGGTCCTCTGTAGGAA
TCATTGCGGGACTTGTTCTGCTCGGGGCCGTAATAACTGGAGCGGTCGTGGCGGC
TGTGATGTGGCGACGGAAAAGCAGTGATCGGAAGGGTGGTTCATATACCCAAGCC
GCTTCTAGCGATAGCGCACAAGGATCAGATGTTTCTCTTACCGCATGTAAAGTA

Protein
MKWVTFISLLFLFSSAYSMRCIGMSNRDFVEGVSGGSWVDIVLEHGSCVTTMAKNKPT
LDFELIKTEAKQPATLRKYCIEAKLTNTTTESRCPTQGEPSLNEEQDKRFVCKHSMVDR
GWGNGCDLFGKGGIVTCAMFRCKKNMEGKVVQPENLEYTIVITPHSGEEHAVGNDTG
KHGKEIKITPQSSITEAELTGYGTVTMECSPRTGLDFNEMVLLQMENKAWLVHRQWFL
DLPLPWLPGADTQGSNWIQKETLVTFKNPHAKKQDVVVLGSQEGAMHTALTGATEIQ
MSSGNLLFTGHLKCRLRMDKLQLKGMSYSMCTGKFKVVKEIAETQHGTIVIRVQYEGD
GSPCKIPFEIMDLEKRHVLGRLITVNPIVTEKDSPVNIEAEPPFGDSYIIIGVEPGQLKLN
WFKKSRGSSGGSEQKLISEEDLGSGGGSSVGIIAGLVLLGAVITGAVVAAVMWRRKSS
DRKGGSYTQAASSDSAQGSDVSLTACKV

pD2sE_Dsp_PM1

DNA
ATGAAGTGGGTAACCTTTATTTCCCTTCTTTTTCTCTTTAGCTCGGCTTATTCCATGA
GGTGTATCGGCCTGTCCAACAGGGACTTTGTGGAGGGAGTGAGCGGCGGCAGCT
GGGTGGACATTGTGCTGGAGCATGGAAGCTGCGTGACCACGATGGCGAAAAACAA
GCCCACCCTGGACTTCGAGCTCATCAAGACAGAGGCTAAACAGCCCGCCACCCTC
AGGAAGTACTGCATCGAGGCCAAGCTGACCAACACAACAACCGAGTCCAGATGCC
CTACACAGGGCGAACCCAGCCTCAACGAAGAGCAGGACAAGAGGTTCGTGTGCAA
ACACAGCATGGTGGACAGGGGCTGGGGCAATGGATGCGGACTCTTCGGAAAAGG
CGGCATCGTGACCTGCGCCATGTTCAGGTGTAAAAAGAACATGGAAGGCAAGGTG
GTGCAGCCCGAAAATCTGGAGTATACCATCGTGATTACCCCCCACAGCGGAGAGG
AGCACGCCGTGGGCAATGACACCGGCAAGCACGGCAAAGAGATTAAGATCACCC
CCCAGTCCTCCATTACCGAAGCTGAACTGACAGGCTACGGCACCGTGACAATGGA
GTGTAGCCCCAGGACCGGACTGGATTTCAACGAGATGGTCCTGCTACAGATGGAG
AACAAGGCCTGGCTCGTGCACAGGCAATGGTTTCTGGATCTGCCTCTGCCTTGGC
TGCCTGGCGCCGATACACAGGGCTCCAACTGGATACAGAAAGAGACCCTCGTCAC
CTTCAAGAATCCCCATGCTAAGAAGCAGGACGTGGTGGTGCTGGGCAGCCAAGAA
GGCGCCATGCACACAGCCCTGACCGGAGCTACCGAGATCCAGATGAGCTCCGGC
AACCTGCTGTTCACCGGCCATCTGAAATGTAGGCTGAGGATGGATAAGCTGCAAC
TCAAAGGCATGTCCTACTCCATGTGCACCGGAAAGTTCAAGGTGGTGAAAGAAATC
GCCGAAACAATGCACGGCACCATCGTGATCAGGGTGCAGTATGAGGGCGACGGC
TCCCCCTGTAAGATCCCCTTCGAAATCATGGACCTGGAAAAGAGGCACGTGCTGG
GCAGGCTCATCACCGTGAACCCCATTGTCACAGAGAAAGACTCCCCCGTGAACAT
CGAGGCCGAGCCTCCCTTTGGCGACTCCTACATCATCATTGGCGTGGAGCCCGGA
CAGCTCAAGCTGAACTGGTTCAAGAAGTCTAGAGGCAGCAGCGGCGGCAGCGAA
CAAAAACTCATCTCAGAAGAGGATCTGGGAAGTGGAGGCGGGTCCTCTGTAGGAA
TCATTGCGGGACTTGTTCTGCTCGGGGCCGTAATAACTGGAGCGGTCGTGGCGGC
TGTGATGTGGCGACGGAAAAGCAGTGATCGGAAGGGTGGTTCATATACCCAAGCC
GCTTCTAGCGATAGCGCACAAGGATCAGATGTTTCTCTTACCGCATGTAAAGTA

Protein
MKWVTFISLLFLFSSAYSMRCIGLSNRDFVEGVSGGSWVDIVLEHGSCVTTMAKNKPT
LDFELIKTEAKQPATLRKYCIEAKLTNTTTESRCPTQGEPSLNEEQDKRFVCKHSMVDR
GWGNGCGLFGKGGIVTCAMFRCKKNMEGKVVQPENLEYTIVITPHSGEEHAVGNDTG
KHGKEIKITPQSSITEAELTGYGTVTMECSPRTGLDFNEMVLLQMENKAWLVHRQWFL
DLPLPWLPGADTQGSNWIQKETLVTFKNPHAKKQDVVVLGSQEGAMHTALTGATEIQ
MSSGNLLFTGHLKCRLRMDKLQLKGMSYSMCTGKFKVVKEIAETMHGTIVIRVQYEGD
GSPCKIPFEIMDLEKRHVLGRLITVNPIVTEKDSPVNIEAEPPFGDSYIIIGVEPGQLKLN
WFKKSRGSSGGSEQKLISEEDLGSGGGSSVGIIAGLVLLGAVITGAVVAAVMWRRKSS
DRKGGSYTQAASSDSAQGSDVSLTACKV

pD2sE_Dsp_PM2

DNA

ATGAAGTGGGTAACCTTTATTTCCCTTCTTTTTCTCTTTAGCTCGGCTTATTCCATGA
GGTGTATCGGCATGTCCAACAGGGACTTTGTGGAGGGAGTGAGCGGCGGCAGCT
GGGTGGACATTGTGCTGGAGCATGGAAGCTGCGTGACCACGATGGCGAAAAACAA
GCCCACCCTGGACTTCGAGCTCATCAAGACAGAGGCTAAACAGCCCGCCACCCTC
AGGAAGTACTGCATCGAGGCCAAGCTGACCAACACAACAACCGAGTCCAGATGCC
CTACACAGGGCGAACCCAGCCTCAACGAAGAGCAGGACAAGAGGTTCGTGTGCAA
ACACAGCATGGTGGACAGGGGCTGGGGCAATGGATGCGGACTCTTCGGAAAAGG
CGGCATCGTGACCTGCGCCATGTTCAGGTGTAAAAAGAACATGGAAGGCAAGGTG
GTGATCCCCGAAAATCTGGAGTATACCATCGTGATTACCCCCCACAGCGGAGAGG
AGCACGCCGTGGGCAATGACACCGGCAAGCACGGCAAAGAGATTAAGATCACCC
CCCAGTCCTCCATTACCGAAGCTGAACTGACAGGCTACGGCACCGTGACAATGGA
GTGTAGCCCCAGGACCGGACTGGATTTCAACGAGATGGTCCTGCTACAGATGGAG
AACAAGGCCTGGCTCGTGCACAGGCAATGGTTTCTGGATCTGCCTCTGCCTTGGC
TGCCTGGCGCCGATACACAGGGCTCCAACTGGATACAGAAAGAGACCCTCGTCAC
CTTCAAGAATCCCCATGCTAAGAAGCAGGACGTGGTGGTGCTGGGCAGCCAAGAA
GGCGCCATGCACACAGCCCTGACCGGAGCTACCGAGATCCAGATGAGCTCCGGC
AACCTGCTGTTCACCGGCCATCTGAAATGTAGGCTGAGGATGGATAAGCTGCAAC
TCAAAGGCATGTCCCTGTCCATGTGCACCGGAAAGTTCAAGGTGGTGAAAGAAAT
CGCCGAAACACAGCACGGCACCATCGTGATCAGGGTGCAGTATGAGGGCGACGG
CTCCCCCTGTAAGATCCCCTTCGAAATCATGGACCTGGAAAAGAGGCACGTGCTG
GGCAGGCTCATCACCGTGAACCCCATTGTCACAGAGAAAGACTCCCCCGTGAACA
TCGAGGCCGAGCCTCCCTTTGGCGACTCCTACATCATCATTGGCGTGGAGCCCGG
ACAGCTCAAGCTGAACTGGTTCAAGAAGTCTAGAGGCAGCAGCGGCGGCAGCGA
ACAAAAACTCATCTCAGAAGAGGATCTGGGAAGTGGAGGCGGGTCCTCTGTAGGA
ATCATTGCGGGACTTGTTCTGCTCGGGGCCGTAATAACTGGAGCGGTCGTGGCGG
CTGTGATGTGGCGACGGAAAAGCAGTGATCGGAAGGGTGGTTCATATACCCAAGC
CGCTTCTAGCGATAGCGCACAAGGATCAGATGTTTCTCTTACCGCATGTAAAGTA

Protein

MKWVTFISLLFLFSSAYSMRCIGMSNRDFVEGVSGGSWVDIVLEHGSCVTTMAKNKPT
LDFELIKTEAKQPATLRKYCIEAKLTNTTTESRCPTQGEPSLNEEQDKRFVCKHSMVDR
GWGNGCGLFGKGGIVTCAMFRCKKNMEGKVVIPENLEYTIVITPHSGEEHAVGNDTG
KHGKEIKITPQSSITEAELTGYGTVTMECSPRTGLDFNEMVLLQMENKAWLVHRQWFL
DLPLPWLPGADTQGSNWIQKETLVTFKNPHAKKQDVVVLGSQEGAMHTALTGATEIQ
MSSGNLLFTGHLKCRLRMDKLQLKGMSLSMCTGKFKVVKEIAETQHGTIVIRVQYEGD
GSPCKIPFEIMDLEKRHVLGRLITVNPIVTEKDSPVNIEAEPPFGDSYIIIGVEPGQLKLN
WFKKSRGSSGGSEQKLISEEDLGSGGGSSVGIIAGLVLLGAVITGAVVAAVMWRRKSS
DRKGGSYTQAASSDSAQGSDVSLTACKV

pD2sE_Dsp_PM3

DNA
ATGAAGTGGGTAACCTTTATTTCCCTTCTTTTTCTCTTTAGCTCGGCTTATTCCATGA
GGTGTATCGGCATGTCCAACAGGGACTTTGTGGAGGGAGTGAGCGGCGGCAGCT
GGGTGGACATTGTGCTGGAGCATGGAAGCTGCGTGACCACGATGATGAAAAACAA
GCCCACCCTGGACTTCGAGCTCATCAAGACAGAGGCTAAACAGCCCGCCACCCTC
AGGAAGTACTGCATCGAGGCCAAGCTGACCAACACAACAACCGAGTCCAGATGCC
CTACACAGGGCGAACCCAGCCTCAACGAAGAGCAGGACAAGAGGTTCGTGTGCAA
ACACAGCATGGTGGACAGGGGCTGGGGCAATGGATGCGGACTCTTCGGAAAAGG
CGGCATCGTGACCTGCGCCATGTTCAGGTGTAAAAAGAACATGGAAGGCAAGGTG
GTGCAGCCCGAAAATCTGGAGTATACCATCGTGATTACCCCCCACAGCGGAGAGG
AGCACGCCGTGGGCAATGACACCGGCAAGCACGGCAAAGAGATTAAGATCACCC
CCCAGTCCTCCATTACCGAAGCTGAACTGACAGGCTACGGCACCGTGACAATGGA
GTGTAGCCCCAGGACCGGACTGGATTTCAACGAGATGGTCCTGCTACAGATGGAG
AACAAGGCCTGGCTCGTGCACAGGCAATGGTTTCTGGATCTGCCTCTGCCTTGGC
TGCCTGGCGCCGATACACAGGGCTCCAACTGGATACAGAAAGAGACCCTCGTCAC
CTTCAAGAATCCCCATGCTAAGAAGCAGGACGTGGTGGTGCTGGGCAGCCAAGAA
GGCGCCATGCACACAGCCCTGACCGGAGCTACCGAGATCCAGATGAGCTCCGGC
AACCTGCTGTTCACCGGCCATCTGAAATGTAGGCTGAGGTGGGATAAGCTGCAAC
TCAAAGGCATGTCCTACTCCATGTGCACCGGAAAGTTCAAGGTGGTGAAAGAAATC
GCCGAAACACAGCACGGCACCATCGTGATCAGGGTGCAGTATGAGGGCGACGGC
TCCCCCTGTAAGATCCCCTTCGAAATCATGGACCTGGAAAAGAGGCACGTGCTGG
GCAGGCTCATCACCGTGAACCCCATTGTCACAGAGAAAGACTCCCCCGTGAACAT
CGAGGCCGAGCCTCCCTTTGGCGACTCCTACATCATCATTGGCGTGGAGCCCGGA
CAGCTCAAGCTGAACTGGTTCAAGAAGTCTAGAGGCAGCAGCGGCGGCAGCGAA
CAAAAACTCATCTCAGAAGAGGATCTGGGAAGTGGAGGCGGGTCCTCTGTAGGAA
TCATTGCGGGACTTGTTCTGCTCGGGGCCGTAATAACTGGAGCGGTCGTGGCGGC
TGTGATGTGGCGACGGAAAAGCAGTGATCGGAAGGGTGGTTCATATACCCAAGCC
GCTTCTAGCGATAGCGCACAAGGATCAGATGTTTCTCTTACCGCATGTAAAGTA

Protein
MKWVTFISLLFLFSSAYSMRCIGMSNRDFVEGVSGGSWVDIVLEHGSCVTTMMKNKP
TLDFELIKTEAKQPATLRKYCIEAKLTNTTTESRCPTQGEPSLNEEQDKRFVCKHSMVD
RGWGNGCGLFGKGGIVTCAMFRCKKNMEGKVVQPENLEYTIVITPHSGEEHAVGNDT
GKHGKEIKITPQSSITEAELTGYGTVTMECSPRTGLDFNEMVLLQMENKAWLVHRQWF
LDLPLPWLPGADTQGSNWIQKETLVTFKNPHAKKQDVVVLGSQEGAMHTALTGATEIQ
MSSGNLLFTGHLKCRLRWDKLQLKGMSYSMCTGKFKVVKEIAETQHGTIVIRVQYEGD
GSPCKIPFEIMDLEKRHVLGRLITVNPIVTEKDSPVNIEAEPPFGDSYIIIGVEPGQLKLN
WFKKSRGSSGGSEQKLISEEDLGSGGGSSVGIIAGLVLLGAVITGAVVAAVMWRRKSS
DRKGGSYTQAASSDSAQGSDVSLTACKV

pD2sE_Dsp_PM4

DNA
ATGAAGTGGGTAACCTTTATTTCCCTTCTTTTTCTCTTTAGCTCGGCTTATTCCATGA
GGTGTATCGGCATGTCCAACAGGGACTTTGTGGAGGGAGTGAGCGGCGGCAGCT
GGGTGGACATTGTGCTGGAGCATGGAAAGTGCGTGACCGTGATGATGAAAAACAA
GCCCACCCTGGACTTCGAGCTCATCAAGACAGAGGCTAAACAGCCCGCCACCCTC
AGGAAGTACTGCATCGAGGCCAAGCTGACCAACACAACAACCGAGTCCAGATGCC
CTACACAGGGCGAACCCAGCCTCAACGAAGAGCAGGACAAGAGGTTCGTGTGCAA
ACACAGCATGGTGGACAGGGGCTGGGGCAATGGATGCGGACTCTTCGGAAAAGG
CGGCATCGTGACCTGCGCCATGTTCAGGTGTAAAAAGAACATGGAAGGCAAGGTG
GTGCAGCCCGAAAATCTGGAGTATACCATCGTGATTACCCCCCACAGCGGAGAGG
AGCACGCCGTGGGCAATGACACCGGCAAGCACGGCAAAGAGATTAAGATCACCC
CCCAGTCCTCCATTACCGAAGCTGAACTGACAGGCTACGGCACCGTGACAATGGA
GTGTAGCCCCAGGACCGGACTGGATTTCAACGAGATGGTCCTGCTACAGATGGAG
AACAAGGCCTGGCTCGTGCACAGGCAATGGTTTCTGGATCTGCCTCTGCCTTGGC
TGCCTGGCGCCGATACACAGGGCTCCAACTGGATACAGAAAGAGACCCTCGTCAC
CTTCAAGAATCCCCATGCTAAGAAGCAGGACGTGGTGGTGCTGGGCAGCCAAGAA
GGCGCCATGCACACAGCCCTGACCGGAGCTACCGAGATCCAGATGAGCTCCGGC
AACCTGCTGTTCACCGGCCATCTGAAATGTAGGCTGAGGATGGATAAGCTGCAAC
TCAAAGGCATGTCCTACTCCATGTGCACCGGAAAGTTCAAGGTGGTGAAAGAAATC
GCCGAAACACAGCACGGCACCATCGTGATCAGGGTGCAGTATGAGGGCGACGGC
TCCCCCTGTAAGATCCCCTTCGAAATCATGGACCTGGAAAAGAGGCACGTGCTGG
GCAGGCTCATCACCGTGAACCCCATTGTCACAGAGAAAGACTCCCCCGTGAACAT
CGAGGCCGAGCCTCCCTTTGGCGACTCCTACATCATCATTGGCGTGGAGCCCGGA
CAGCTCAAGCTGAACTGGTTCAAGAAGTCTAGAGGCAGCAGCGGCGGCAGCGAA
CAAAAACTCATCTCAGAAGAGGATCTGGGAAGTGGAGGCGGGTCCTCTGTAGGAA
TCATTGCGGGACTTGTTCTGCTCGGGGCCGTAATAACTGGAGCGGTCGTGGCGGC
TGTGATGTGGCGACGGAAAAGCAGTGATCGGAAGGGTGGTTCATATACCCAAGCC
GCTTCTAGCGATAGCGCACAAGGATCAGATGTTTCTCTTACCGCATGTAAAGTA

Protein
MKWVTFISLLFLFSSAYSMRCIGMSNRDFVEGVSGGSWVDIVLEHGKCVTVMMKNKP
TLDFELIKTEAKQPATLRKYCIEAKLTNTTTESRCPTQGEPSLNEEQDKRFVCKHSMVD
RGWGNGCGLFGKGGIVTCAMFRCKKNMEGKVVQPENLEYTIVITPHSGEEHAVGNDT
GKHGKEIKITPQSSITEAELTGYGTVTMECSPRTGLDFNEMVLLQMENKAWLVHRQWF
LDLPLPWLPGADTQGSNWIQKETLVTFKNPHAKKQDVVVLGSQEGAMHTALTGATEIQ
MSSGNLLFTGHLKCRLRMDKLQLKGMSYSMCTGKFKVVKEIAETQHGTIVIRVQYEGD
GSPCKIPFEIMDLEKRHVLGRLITVNPIVTEKDSPVNIEAEPPFGDSYIIIGVEPGQLKLN
WFKKSRGSSGGSEQKLISEEDLGSGGGSSVGIIAGLVLLGAVITGAVVAAVMWRRKSS
DRKGGSYTQAASSDSAQGSDVSLTACKV

pD2sE_Dsp_PM5

DNA
ATGAAGTGGGTAACCTTTATTTCCCTTCTTTTTCTCTTTAGCTCGGCTTATTCCATGA
GGTGTATCGGCATGTCCAACAGGGACTTTGTGGAGGGAGTGAGCGGCGGCAGCT
GGGTGGACATTGTGCTGGAGCATGGAAGCTGCGTGACCACGATGGCGAAAAACAA
GCCCACCCTGGACTTCGAGCTCATCAAGACAGAGGCTAAACAGCCCGCCACCCTC
AGGAAGTACTGCATCGAGGCCAAGCTGACCAACACAACAACCGAGTCCAGATGCC
CTACACAGGGCGAACCCAGCCTCAACGAAGAGCAGGACAAGAGGTTCGTGTGCAA
ACACAGCATGGTGGACAGGGGCTGGGGCAATGGATGCGGACTCTTCGGAAAAGG
CGGCATCGTGACCTGCGCCATGTTCAGGTGTAAAAAGAACATGGAAGGCAAGGTG
GTGCAGCCCGAAAATCTGGAGTATACCATCGTGATTACCCCCCACAGCGGAGAGG
AGCACGCCGTGGGCAATGACACCGGCAAGCACGGCAAAGAGATTAAGATCACCC
CCCAGTCCTCCATTACCGAAGCTGAACTGACAGGCTACGGCACCGTGACAATGGA
GTGTAGCCCCAGGACCGGACTGGATTTCAACGAGATGGTCCTGCTACAGATGGAG
AACAAGGCCTGGCTCGTGGACAGGCAATGGTTTCTGGATCTGCCTCTGCCTTGGC
TGCCTGGCGCCGATACACAGGGCTCCAACTGGATACAGAAAGAGACCCTCGTCAC
CTTCAAGAATCCCCATGCTAAGAAGCAGGACGTGGTGGTGCTGGGCAGCCAAGAA
GGCGCCATGCACACAGCCCTGACCTGGGCTACCGAGATCCAGATGAGCTCCGGC
AACCTGCTGTTCACCGGCCATCTGAAATGTAGGCTGAGGATGGATAAGCTGCAAC
TCAAAGGCATGTCCTACTCCATGTGCACCGGAAAGTTCAAGGTGGTGAAAGAAATC
GCCGAAACACAGCACGGCACCATCGTGATCAGGGTGCAGTATGAGGGCGACGGC
TCCCCCTGTAAGATCCCCTTCGAAATCATGGACCTGGAAAAGAGGCACGTGCTGG
GCAGGCTCATCACCGTGAACCCCATTGTCACAGAGAAAGACTCCCCCGTGAACAT
CGAGGCCGAGCCTCCCTTTGGCGACTCCTACATCATCATTGGCGTGGAGCCCGGA
CAGCTCAAGCTGAACTGGTTCAAGAAGTCTAGAGGCAGCAGCGGCGGCAGCGAA
CAAAAACTCATCTCAGAAGAGGATCTGGGAAGTGGAGGCGGGTCCTCTGTAGGAA
TCATTGCGGGACTTGTTCTGCTCGGGGCCGTAATAACTGGAGCGGTCGTGGCGGC
TGTGATGTGGCGACGGAAAAGCAGTGATCGGAAGGGTGGTTCATATACCCAAGCC
GCTTCTAGCGATAGCGCACAAGGATCAGATGTTTCTCTTACCGCATGTAAAGTA

Protein
MKWVTFISLLFLFSSAYSMRCIGMSNRDFVEGVSGGSWVDIVLEHGSCVTTMAKNKPT
LDFELIKTEAKQPATLRKYCIEAKLTNTTTESRCPTQGEPSLNEEQDKRFVCKHSMVDR
GWGNGCGLFGKGGIVTCAMFRCKKNMEGKVVQPENLEYTIVITPHSGEEHAVGNDTG
KHGKEIKITPQSSITEAELTGYGTVTMECSPRTGLDFNEMVLLQMENKAWLVDRQWFL
DLPLPWLPGADTQGSNWIQKETLVTFKNPHAKKQDVVVLGSQEGAMHTALTWATEIQ
MSSGNLLFTGHLKCRLRMDKLQLKGMSYSMCTGKFKVVKEIAETQHGTIVIRVQYEGD
GSPCKIPFEIMDLEKRHVLGRLITVNPIVTEKDSPVNIEAEPPFGDSYIIIGVEPGQLKLN
WFKKSRGSSGGSEQKLISEEDLGSGGGSSVGIIAGLVLLGAVITGAVVAAVMWRRKSS
DRKGGSYTQAASSDSAQGSDVSLTACKV

pD2sE_Dsp_PM6

DNA
ATGAAGTGGGTAACCTTTATTTCCCTTCTTTTTCTCTTTAGCTCGGCTTATTCCATGA
GGTGTATCGGCATGTCCAACAGGGACTTTGTGGAGGGAGTGAGCGGCGGCAGCT
GGGTGGACATTGTGCTGGAGCATGGAAGCTGCGTGACCACGATGGCGAAAAACAA
GCCCACCCTGGACTTCGAGCTCATCAAGACAGAGGCTAAACAGCCCGCCACCCTC
AGGAAGTACTGCATCGAGGCCAAGCTGACCAACACAACAACCGAGTCCAGATGCC
CTACACAGGGCGAACCCAGCCTCAACGAAGAGCAGGACAAGAGGTTCGTGTGCAA
ACACAGCATGGTGGACAGGGGCTGGGGCAATGGATGCGGACTCTTCGGAAAAGG
CGGCATCGTGACCTGCGCCATGTTCAGGTGTAAAAAGAACATGGAAGGCAAGGTG
GTGCAGCCCGAAAATCTGGAGTATACCATCGTGATTACCCCCCACAGCGGAGAGG
AGCACGCCGTGGGCAATGACACCGGCAAGCACGGCAAAGAGATTAAGATCACCC
CCCAGTCCTCCATTACCGAAGCTGAACTGACAGGCTACGGCACCGTGACAATGGA
GTGTAGCCCCAGGACCGGACTGGATTTCAACGAGATGGTCCTGCTACAGATGGAG
AACAAGGCCTGGCTCGTGCACAGGCAATGGTTTCTGGATCTGCCTCTGCCTTGGC
TGCCTGGCGCCGATACACAGGGCTCCAACTGGATACAGAAAGAGACCCTCGTCAC
CTTCAAGAATCCCCATGCTAAGAAGCAGGACGTGGTGGTGCTGGGCAGCCAAGAA
GGCGCCATGCACACAGCCCTGACCGGAGCTACCGAGATCCAGATGAGCTCCGGC
AACCTGCTGTTCACCGGCCATCTGAAATGTAGGCTGAGGATGGATAAGCTGCAAC
TCAAAGGCATGTCCTACTCCATGTGCACCGGAAAGTTCAAGGTGGTGAAAGAAATC
GCCGAAACACAGCACGGCACCATCGTGATCAGGGTGCAGTATGAGGGCGACGGC
TCCCCCTGTAAGATCCCCTTCGAAATCATGGACCTGGAAAAGAGGCACGTGCTGG
GCAGGCTCATCACCGTGAACCCCATTGTCACAGAGAAAGACTCCCCCGTGAACAT
CGAGGCCGAGCCTCCCTTTGGCCTGTCCTACATCATCATTGGCGTGGAGCCCGGA
CAGCTCAAGCTGCAGTGGTTCAAGAAGTCTAGAGGCAGCAGCGGCGGCAGCGAA
CAAAAACTCATCTCAGAAGAGGATCTGGGAAGTGGAGGCGGGTCCTCTGTAGGAA
TCATTGCGGGACTTGTTCTGCTCGGGGCCGTAATAACTGGAGCGGTCGTGGCGGC
TGTGATGTGGCGACGGAAAAGCAGTGATCGGAAGGGTGGTTCATATACCCAAGCC
GCTTCTAGCGATAGCGCACAAGGATCAGATGTTTCTCTTACCGCATGTAAAGTA

Protein
MKWVTFISLLFLFSSAYSMRCIGMSNRDFVEGVSGGSWVDIVLEHGSCVTTMAKNKPT
LDFELIKTEAKQPATLRKYCIEAKLTNTTTESRCPTQGEPSLNEEQDKRFVCKHSMVDR
GWGNGCGLFGKGGIVTCAMFRCKKNMEGKVVQPENLEYTIVITPHSGEEHAVGNDTG
KHGKEIKITPQSSITEAELTGYGTVTMECSPRTGLDFNEMVLLQMENKAWLVHRQWFL
DLPLPWLPGADTQGSNWIQKETLVTFKNPHAKKQDVVVLGSQEGAMHTALTGATEIQ
MSSGNLLFTGHLKCRLRMDKLQLKGMSYSMCTGKFKVVKEIAETQHGTIVIRVQYEGD
GSPCKIPFEIMDLEKRHVLGRLITVNPIVTEKDSPVNIEAEPPFGLSYIIIGVEPGQLKLQ
WFKKSRGSSGGSEQKLISEEDLGSGGGSSVGIIAGLVLLGAVITGAVVAAVMWRRKSS
DRKGGSYTQAASSDSAQGSDVSLTACKV

pD2sE_Dsp_PM7

DNA
ATGAAGTGGGTAACCTTTATTTCCCTTCTTTTTCTCTTTAGCTCGGCTTATTCCATGA
GGTGTATCGGCATGTCCAACAGGGACTTTGTGGAGGGAGTGAGCGGCGGCAGCT
GGGTGGACATTGTGCTGGAGCATGGAAGCTGCGTGACCACGATGGCGAAAAACAA
GCCCACCCTGGACTTCGAGCTCATCAAGACAGAGGCTAAACAGCCCGCCACCCTC
AGGAAGTACTGCATCGAGGCCAAGCTGACCAACACAACAACCGAGTCCAGATGCC
CTACACAGGGCGAACCCAGCCTCAACGAAGAGCAGGACAAGAGGTTCGTGTGCAA
ACACAGCATGGTGGACAGGGGCTGGGGCAATGGATGCGGACTCTTCGGAAAAGG
CGGCATCGTGACCTGCGCCATGTTCAGGTGTAAAAAGAACATGGAAGGCAAGGTG
GTGCAGCCCGAAAATCTGGAGTATACCATCGTGATTACCCCCCACAGCGGAGAGG
AGCACGCCGTGGGCAATGACACCGGCAAGCACGGCAAAGAGATTAAGATCACCC
CCCAGTCCTCCATTACCGAAGCTGAACTGACAGGCTACGGCACCGTGACAATGGA
GTGTAGCCCCAGGACCGGACTGGATTTCAACGAGATGGTCCTGCTACAGATGGAG
AACAAGGCCTGGCTCGTGCACAGGCAATGGTTTCTGGATCTGCCTCTGCCTTGGC
TGCCTGGCGCCGATACACAGGGCTCCAACTGGATACAGAAAGAGACCCTCGTCAC
CTTCAAGAATCCCCATGCTAAGAAGCAGGACGTGGTGGTGCTGGGCAGCCAAGAA
GGCGCCATGCACACAGCCCTGACCGGAGCTACCGAGATCCAGATGAGCTCCGGC
AACCTGCTGTTCACCGGCCATCTGAAATGTAGGCTGAGGATGGATAAGCTGCAAC
TCAAAGGCATGTCCTACTCCATGTGCACCGGAAAGTTCAAGGTGGTGAAAGAAATC
GCCGAAACACAGCACGGCACCATCGTGATCAGGGTGCAGTATGAGGGCGACGCC
TCCCCCTGTAAGATCCCCTTCGAAATCATGGACCTGGAAAAGAGGCACGTGCTGG
GCAGGCTCATCACCGTGAACCCCATTGTCACAGAGAAAGACTCCCCCGTGAACAT
CGAGGCCGAGCCTCCCTTTGGCGACTCCTACATCATCATTGGCGTGGAGCCCGGA
CAGCTCAAGCTGAACTGGTTCAAGAAGTCTAGAGGCAGCAGCGGCGGCAGCGAA
CAAAAACTCATCTCAGAAGAGGATCTGGGAAGTGGAGGCGGGTCCTCTGTAGGAA
TCATTGCGGGACTTGTTCTGCTCGGGGCCGTAATAACTGGAGCGGTCGTGGCGGC
TGTGATGTGGCGACGGAAAAGCAGTGATCGGAAGGGTGGTTCATATACCCAAGCC
GCTTCTAGCGATAGCGCACAAGGATCAGATGTTTCTCTTACCGCATGTAAAGTA

Protein
MKWVTFISLLFLFSSAYSMRCIGMSNRDFVEGVSGGSWVDIVLEHGSCVTTMAKNKPT
LDFELIKTEAKQPATLRKYCIEAKLTNTTTESRCPTQGEPSLNEEQDKRFVCKHSMVDR
GWGNGCGLFGKGGIVTCAMFRCKKNMEGKVVQPENLEYTIVITPHSGEEHAVGNDTG
KHGKEIKITPQSSITEAELTGYGTVTMECSPRTGLDFNEMVLLQMENKAWLVHRQWFL
DLPLPWLPGADTQGSNWIQKETLVTFKNPHAKKQDVVVLGSQEGAMHTALTGATEIQ
MSSGNLLFTGHLKCRLRMDKLQLKGMSYSMCTGKFKVVKEIAETQHGTIVIRVQYEGD
ASPCKIPFEIMDLEKRHVLGRLITVNPIVTEKDSPVNIEAEPPFGDSYIIIGVEPGQLKLN
WFKKSRGSSGGSEQKLISEEDLGSGGGSSVGIIAGLVLLGAVITGAVVAAVMWRRKSS
DRKGGSYTQAASSDSAQGSDVSLTACKV

pD2sE_Dsp_PM8

DNA
ATGAAGTGGGTAACCTTTATTTCCCTTCTTTTTCTCTTTAGCTCGGCTTATTCCATGA
GGTGTATCGGCATGTCCAACAGGGACTTTGTGGAGGGAGTGAGCGGCGGCAGCT
GGGTGGACATTGTGCTGGAGCATGGAAGCTGCGTGACCACGATGGCGAAAAACAA
GCCCACCCTGGACTTCGAGCTCATCAAGACAGAGGCTAAACAGCCCGCCACCCTC
AGGAAGTACTGCATCGAGGCCAAGCTGACCAACACAACAACCGAGTCCAGATGCC
CTACACAGGGCGAACCCAGCCTCAACGAAGAGCAGGACAAGAGGTTCGTGTGCAA
ACACAGCATGGTGGACAGGGGCTGGGGCAATGGATGCGGACTCTTCGGAAAAGG
CGGCATCGTGACCTGCGCCATGTTCAGGTGTAAAAAGAACATGGAAGGCAAGGTG
GTGCAGCCCGAAAATCTGGAGTATACCATCGTGATTACCCCCCACAGCGGAGAGG
AGCACGCCGTGGGCAATGACACCGGCAAGCACGGCAAAGAGATTAAGATCACCC
CCCAGTCCTCCATTACCGAAGCTGAACTGACAGGCTACGGCACCGTGACAATGGA
GTGTAGCCCCAGGACCGGACTGGATTTCAACGAGATGGTCCTGCTACAGATGGAG
AACAAGGCCTGGCTCGTGCACAGGCAATGGTTTCTGGATCTGCCTCTGCCTTGGC
TGCCTGGCGCCGATACACAGGGCTCCAACTGGATACAGAAAGAGACCCTCGTCAC
CTTCAAGAATCCCCATGCTAAGAAGCAGGACGTGGTGGTGCTGGGCAGCCAAGAA
GGCGCCATGCACACAGCCCTGACCGGAGCTACCGAGATCCAGATGAGCTCCGGC
AACCTGCTGTTCACCGGCCATCTGAAATGTAGGCTGAGGATGGATAAGCTGCAAC
TCAAAGGCATGTCCTACTCCATGTGCACCGGAAAGTTCAAGGTGGTGAAAGAAATC
GCCGAAACACAGCACGGCACCATCGTGATCAGGGTGCAGTATGAGGGCGACGGC
TCCCCCTGTAAGATCCCCTTCGAAATCATGGACCTGGAAAAGAGGCACGTGCTGG
GCAGGCTCATCACCGTGAACCCCATTGTCACAGAGAAAGACTCCCCCGTGAACAT
CGAGGCCGAGCCTCCCTTTGGCGACTCCGTGATCATCATTGGCGTGGAGCCCGG
ACAGCTCAAGCTGAACTGGTTCCGGAAGTCTAGAGGCAGCAGCGGCGGCAGCGA
ACAAAAACTCATCTCAGAAGAGGATCTGGGAAGTGGAGGCGGGTCCTCTGTAGGA
ATCATTGCGGGACTTGTTCTGCTCGGGGCCGTAATAACTGGAGCGGTCGTGGCGG
CTGTGATGTGGCGACGGAAAAGCAGTGATCGGAAGGGTGGTTCATATACCCAAGC
CGCTTCTAGCGATAGCGCACAAGGATCAGATGTTTCTCTTACCGCATGTAAAGTA

Protein
MKWVTFISLLFLFSSAYSMRCIGMSNRDFVEGVSGGSWVDIVLEHGSCVTTMAKNKPT
LDFELIKTEAKQPATLRKYCIEAKLTNTTTESRCPTQGEPSLNEEQDKRFVCKHSMVDR
GWGNGCGLFGKGGIVTCAMFRCKKNMEGKVVQPENLEYTIVITPHSGEEHAVGNDTG
KHGKEIKITPQSSITEAELTGYGTVTMECSPRTGLDFNEMVLLQMENKAWLVHRQWFL
DLPLPWLPGADTQGSNWIQKETLVTFKNPHAKKQDVVVLGSQEGAMHTALTGATEIQ
MSSGNLLFTGHLKCRLRMDKLQLKGMSYSMCTGKFKVVKEIAETQHGTIVIRVQYEGD
GSPCKIPFEIMDLEKRHVLGRLITVNPIVTEKDSPVNIEAEPPFGDSVIIIGVEPGQLKLN
WFRKSRGSSGGSEQKLISEEDLGSGGGSSVGIIAGLVLLGAVITGAVVAAVMWRRKSS
DRKGGSYTQAASSDSAQGSDVSLTACKV

pD2sE_Dsp_UndPk1

DNA
ATGAAGTGGGTAACCTTTATTTCCCTTCTTTTTCTCTTTAGCTCGGCTTATTCCATGA
GGTGTATCGGCATGTCCAACAGGGACTTTGTGGAGGGAGTGAGCGGCGGCAGCT
GGGTGGACATTGTGCTGGAGCATGGAAGCTGCGTGACCACGATGGCGAAAAACAA
GCCCACCCTGGACTTCGAGCTCATCAAGACAGAGGCTAAACAGCCCGCCACCCTC
AGGAAGTACTGCATCGAGGCCAAGCTGACCAACACAACAACCGAGTCCAGATGCC
CTACACAGGGCGAACCCAGCCTCAACGAAGAGCAGGACAAGAGGTTCGTGTGCAA
ACACAGCATGGTGGACAGGGGCTGGGGCAATGGATGCGGACTCTTCGGAAAAGG
CGGCATCGTGACCTGCGCCATGTTCAGGTGTAAAAAGAACATGGAAGGCAAGGTG
GTGCAGCCCGAAAATCTGGAGTATACCATCGTGATTACCCCCCACAGCGGAGAGG
AGCACGCCGTGGGCAATGACACCGGCAAGCACGGCAAAGAGATTAAGATCACCC
CCCAGTCCTCCATTACCGAAGCTGAACTGACAGGCTACGGCACCGTGACAATGGA
GTGTAGCCCCAGGACCGGACTGGATTTCAACGAGATGGTCCTGCTACAGATGGAG
AACAAGGCCTGGCTCGTGCACAGGCAATGGTTTCTGGATCTGCCTCTGCCTTGGC
TGCCTGGCGCCGATACACAGGGCTCCAACTGGATACAGAAAGAGACCCTCGTCAC
CTTCAAGAATCCCCATGCTAAGAAGCAGGACGTGGTGGTGCTGGGCAGCCAAGAA
GGCGCCATGCACACAGCCCTGACCGGAGCTACCGAGGTGCAGATGAGCTCCGGC
AACCTGCTGTTCGCCGGCCATCTGAAATGTAGGCTGAGGATGGATAAGCTGCAAC
TCAAAGGCATGTCCTACTCCATGTGCACCGGAAAGTTCAAGGTGGTGAAAGAAATC
GCCGAAACACAGCACGGCACCATCGTGATCAGGGTGCAGTATGAGGGCGACGGC
TCCCCCTGTAAGATCCCCTTCGAAATCATGGACCTGGAAAAGAGGCACGTGCTGG
GCAGGCTCATCACCGTGAACCCCATTGTCACAGAGAAAGACTCCCCCGTGAACAT
CGAGGCCGAGCCTCCCTTTGGCGACTCCTACATCATCATTGGCGTGGAGCCCGGA
CAGCTCAAGCTGAACTGGTTCAAGAAGTCTAGAGGCAGCAGCGGCGGCAGCGAA
CAAAAACTCATCTCAGAAGAGGATCTGGGAAGTGGAGGCGGGTCCTCTGTAGGAA
TCATTGCGGGACTTGTTCTGCTCGGGGCCGTAATAACTGGAGCGGTCGTGGCGGC
TGTGATGTGGCGACGGAAAAGCAGTGATCGGAAGGGTGGTTCATATACCCAAGCC
GCTTCTAGCGATAGCGCACAAGGATCAGATGTTTCTCTTACCGCATGTAAAGTA

Protein
MKWVTFISLLFLFSSAYSMRCIGMSNRDFVEGVSGGSWVDIVLEHGSCVTTMAKNKPT
LDFELIKTEAKQPATLRKYCIEAKLTNTTTESRCPTQGEPSLNEEQDKRFVCKHSMVDR
GWGNGCGLFGKGGIVTCAMFRCKKNMEGKVVQPENLEYTIVITPHSGEEHAVGNDTG
KHGKEIKITPQSSITEAELTGYGTVTMECSPRTGLDFNEMVLLQMENKAWLVHRQWFL
DLPLPWLPGADTQGSNWIQKETLVTFKNPHAKKQDVVVLGSQEGAMHTALTGATEVQ
MSSGNLLFAGHLKCRLRMDKLQLKGMSYSMCTGKFKVVKEIAETQHGTIVIRVQYEGD
GSPCKIPFEIMDLEKRHVLGRLITVNPIVTEKDSPVNIEAEPPFGDSYIIIGVEPGQLKLN
WFKKSRGSSGGSEQKLISEEDLGSGGGSSVGIIAGLVLLGAVITGAVVAAVMWRRKSS
DRKGGSYTQAASSDSAQGSDVSLTACKV

pD2sE_Dsp_UndPk2

DNA
ATGAAGTGGGTAACCTTTATTTCCCTTCTTTTTCTCTTTAGCTCGGCTTATTCCATGA
GGTGTATCGGCATGTCCAACAGGGACTTTGTGGAGGGAGTGAGCGGCGGCAGCT
GGGTGGACATTGTGCTGGAGCATGGAAGCTGCGTGACCACGATGGCGAAAAACAA
GCCCACCCTGGACTTCGAGCTCATCAAGACAGAGGCTAAACAGCCCGCCACCCTC
AGGAAGTACTGCATCGAGGCCAAGCTGACCAACACAACAACCGAGTCCAGATGCC
CTACACAGGGCGAACCCAGCCTCAACGAAGAGCAGGACAAGAGGTTCGTGTGCAA
ACACAGCATGGTGGACAGGGGCTGGGGCAATGGATGCGGACTCTTCGGAAAAGG
CGGCATCGTGACCTGCGCCATGTTCAGGTGTAAAAAGAACATGGAAGGCAAGGTG
GTGCAGCCCGAAAATCTGGAGTATACCATCGTGATTACCCCCCACAGCGGAGAGG
AGCACGCCGTGGGCAATGACACCGGCAAGCACGGCAAAGAGATTAAGATCACCC
CCCAGTCCTCCATTACCGAAGCTGAACTGACAGGCTACGGCACCGTGACAATGGA
GTGTAGCCCCAGGACCGGACTGGATTTCAACGAGATGGTCTGGCTACAGATGGAG
AACAAGGCCTGGCTCGTGCACAGGCAATGGTTTCTGGATCTGCCTCTGCCTTGGC
TGCCTGGCGCCGATACACAGGGCTCCAACTGGATACAGAAAGAGACCCTCGTCAC
CTTCAAGAATCCCCATGCTAAGAAGCAGGACGTGGTGGTGCTGGGCAGCCAAGAA
GGCGCCATGCACACAGCCCTGACCGGAGCTACCGAGATCCAGATGAGCTCCGGC
AACCTGCTGTTCACCGGCCATCTGAAATGTAGGCTGAGGATGGATAAGCTGCAAC
TCAAAGGCATGTCCTACTCCATGTGCACCGGAAAGTTCAAGGTGGTGAAAGAAATC
GCCGAAACACAGCACGGCACCATCGTGATCAGGGTGCAGTATGAGGGCGACGGC
TCCCCCTGTAAGATCCCCTTCGAAATCATGGACCTGGAAAAGAGGCACGTGCTGG
GCAGGCTCATCACCGTGAACCCCATTGTCACAGAGAAAGACTCCCCCGTGAACAT
CGAGGCCGAGCCTCCCTTTGGCGACTCCTACATCATCATTGGCGTGGAGCCCGGA
CAGCTCAAGCTGAACTGGTTCAAGAAGTCTAGAGGCAGCAGCGGCGGCAGCGAA
CAAAAACTCATCTCAGAAGAGGATCTGGGAAGTGGAGGCGGGTCCTCTGTAGGAA
TCATTGCGGGACTTGTTCTGCTCGGGGCCGTAATAACTGGAGCGGTCGTGGCGGC
TGTGATGTGGCGACGGAAAAGCAGTGATCGGAAGGGTGGTTCATATACCCAAGCC
GCTTCTAGCGATAGCGCACAAGGATCAGATGTTTCTCTTACCGCATGTAAAGTA

Protein
MKWVTFISLLFLFSSAYSMRCIGMSNRDFVEGVSGGSWVDIVLEHGSCVTTMAKNKPT
LDFELIKTEAKQPATLRKYCIEAKLTNTTTESRCPTQGEPSLNEEQDKRFVCKHSMVDR
GWGNGCGLFGKGGIVTCAMFRCKKNMEGKVVQPENLEYTIVITPHSGEEHAVGNDTG
KHGKEIKITPQSSITEAELTGYGTVTMECSPRTGLDFNEMVWLQMENKAWLVHRQWF
LDLPLPWLPGADTQGSNWIQKETLVTFKNPHAKKQDVVVLGSQEGAMHTALTGATEIQ
MSSGNLLFTGHLKCRLRMDKLQLKGMSYSMCTGKFKVVKEIAETQHGTIVIRVQYEGD
GSPCKIPFEIMDLEKRHVLGRLITVNPIVTEKDSPVNIEAEPPFGDSYIIIGVEPGQLKLN
WFKKSRGSSGGSEQKLISEEDLGSGGGSSVGIIAGLVLLGAVITGAVVAAVMWRRKSS
DRKGGSYTQAASSDSAQGSDVSLTACKV

pD2sE_Dsp_UndPk3

DNA
ATGAAGTGGGTAACCTTTATTTCCCTTCTTTTTCTCTTTAGCTCGGCTTATTCCATGA
GGTGTATCGGCATGTCCAACAGGGACTTTGTGGAGGGAGTGAGCGGCGGCAGCT
GGGTGGACATTGTGCTGGAGCATGGAAGCTGCGTGACCACGATGGCGAAAAACAA
GCCCACCCTGGACTTCGAGCTCATCAAGACAGAGGCTAAACAGCCCGCCACCCTC
AGGAAGTACTGCATCGAGGCCAAGCTGACCAACACAACAACCGAGTCCAGATGCC
CTACACAGGGCGAACCCAGCCTCAACGAAGAGCAGGACAAGAGGTTCGTGTGCAA
ACACAGCATGGTGGACAGGGGCTGGGGCAATGGATGCGGACTCTTCGGAAAAGG
CGGCATCGTGACCTGCGCCATGTTCAGGTGTAAAAAGAACATGGAAGGCAAGGTG
GTGCAGCCCGAAAATCTGGAGTATACCATCGTGATTACCCCCCACAGCGGAGAGG
AGCACGCCGTGGGCAATGACACCGGCAAGCACGGCAAAGAGATTAAGATCACCC
CCCAGTCCTCCATTACCGAAGCTGAACTGACAGGCTACGGCACCGTGACAATGGA
GTGTAGCCCCAGGACCGGATACGATTTCAACGAGATGGTCCTGCTACAGATGGAG
AACAAGGCCTGGCTCGTGGACAGGCAATGGTTTCTGGATCTGCCTCTGCCTTGGC
TGCCTGGCGCCGATACACAGGGCTCCAACTGGATACAGAAAGAGACCCTCGTCAC
CTTCAAGAATCCCCATGCTAAGAAGCAGGACGTGGTGGTGCTGGGCAGCCAAGAA
GGCGCCATGCACACAGCCCTGACCGGAGCTACCGAGATCCAGATGAGCTCCGGC
AACCTGCTGTTCACCGGCCATCTGAAATGTAGGCTGAGGATGGATAAGCTGCAAC
TCAAAGGCATGTCCTACTCCATGTGCACCGGAAAGTTCAAGGTGGTGAAAGAAATC
GCCGAAACACAGCACGGCACCATCGTGATCAGGGTGCAGTATGAGGGCGACGGC
TCCCCCTGTAAGATCCCCTTCGAAATCATGGACCTGGAAAAGAGGCACGTGCTGG
GCAGGCTCATCACCGTGAACCCCATTGTCACAGAGAAAGACTCCCCCGTGAACAT
CGAGGCCGAGCCTCCCTTTGGCGACTCCTACATCATCATTGGCGTGGAGCCCGGA
CAGCTCAAGCTGAACTGGTTCAAGAAGTCTAGAGGCAGCAGCGGCGGCAGCGAA
CAAAAACTCATCTCAGAAGAGGATCTGGGAAGTGGAGGCGGGTCCTCTGTAGGAA
TCATTGCGGGACTTGTTCTGCTCGGGGCCGTAATAACTGGAGCGGTCGTGGCGGC
TGTGATGTGGCGACGGAAAAGCAGTGATCGGAAGGGTGGTTCATATACCCAAGCC
GCTTCTAGCGATAGCGCACAAGGATCAGATGTTTCTCTTACCGCATGTAAAGTA

Protein
MKWVTFISLLFLFSSAYSMRCIGMSNRDFVEGVSGGSWVDIVLEHGSCVTTMAKNKPT
LDFELIKTEAKQPATLRKYCIEAKLTNTTTESRCPTQGEPSLNEEQDKRFVCKHSMVDR
GWGNGCGLFGKGGIVTCAMFRCKKNMEGKVVQPENLEYTIVITPHSGEEHAVGNDTG
KHGKEIKITPQSSITEAELTGYGTVTMECSPRTGYDFNEMVLLQMENKAWLVDRQWFL
DLPLPWLPGADTQGSNWIQKETLVTFKNPHAKKQDVVVLGSQEGAMHTALTGATEIQ
MSSGNLLFTGHLKCRLRMDKLQLKGMSYSMCTGKFKVVKEIAETQHGTIVIRVQYEGD
GSPCKIPFEIMDLEKRHVLGRLITVNPIVTEKDSPVNIEAEPPFGDSYIIIGVEPGQLKLN
WFKKSRGSSGGSEQKLISEEDLGSGGGSSVGIIAGLVLLGAVITGAVVAAVMWRRKSS
DRKGGSYTQAASSDSAQGSDVSLTACKV

pD2sE_Dsp_UndPk4

DNA
ATGAAGTGGGTAACCTTTATTTCCCTTCTTTTTCTCTTTAGCTCGGCTTATTCCATGA
GGTGTATCGGCATGTCCAACAGGGACTTTGTGTTCGCCGTGAGCGGCGGCAGCTG
GGTGGACATTGTGCTGGAGCATGGAAGCTGCGTGACCACGCTGGCGAAAAACAA
GCCCACCCTGGACTTCGAGCTCATCAAGACAGAGGCTAAACAGCCCGCCACCCTC
AGGAAGTACTGCATCGAGGCCAAGCTGACCAACACAACAACCGAGTCCAGATGCC
CTACACAGGGCGAACCCAGCCTCAACGAAGAGCAGGACAAGAGGTTCGTGTGCAA
ACACAGCATGGTGGACAGGGGCTGGGGCAATGGATGCGGACTCTTCGGAAAAGG
CGGCATCGTGACCTGCGCCATGTTCAGGTGTAAAAAGAACATGGAAGGCAAGGTG
GTGCAGCCCGAAAATCTGGAGTATACCATCGTGATTACCCCCCACAGCGGAGAGG
AGCACGCCGTGGGCAATGACACCGGCAAGCACGGCAAAGAGATTAAGATCACCC
CCCAGTCCTCCATTACCGAAGCTGAACTGACAGGCTACGGCACCGTGACAATGGA
GTGTAGCCCCAGGACCGGACTGGATTTCAACGAGATGGTCCTGCTACAGATGGAG
AACAAGGCCTGGCTCGTGCACAGGCAATGGTTTCTGGATCTGCCTCTGCCTTGGC
TGCCTGGCGCCGATACACAGGGCTCCAACTGGATACAGAAAGAGACCCTCGTCAC
CTTCAAGAATCCCCATGCTAAGAAGCAGGACGTGGTGGTGCTGGGCAGCCAAGAA
GGCGCCATGCACACAGCCCTGACCGGAGCTACCGAGATCCAGATGAGCTCCGGC
AACCTGCTGTTCACCGGCCATCTGAAATGTAGGCTGAGGATGGATAAGCTGCAAC
TCAAAGGCATGTCCTACTCCATGTGCACCGGAAAGTTCAAGGTGGTGAAAGAAATC
GCCGAAACACAGCACGGCACCATCGTGATCAGGGTGCAGTATGAGGGCGACGGC
TCCCCCTGTAAGATCCCCTTCGAAATCATGGACCTGGAAAAGAGGCACGTGCTGG
GCAGGCTCATCACCGTGAACCCCATTGTCACAGAGAAAGACTCCCCCGTGAACAT
CGAGGCCGAGCCTCCCTTTGGCGACTCCTACATCATCATTGGCGTGGAGCCCGGA
CAGCTCAAGCTGAACTGGTTCAAGAAGTCTAGAGGCAGCAGCGGCGGCAGCGAA
CAAAAACTCATCTCAGAAGAGGATCTGGGAAGTGGAGGCGGGTCCTCTGTAGGAA
TCATTGCGGGACTTGTTCTGCTCGGGGCCGTAATAACTGGAGCGGTCGTGGCGGC
TGTGATGTGGCGACGGAAAAGCAGTGATCGGAAGGGTGGTTCATATACCCAAGCC
GCTTCTAGCGATAGCGCACAAGGATCAGATGTTTCTCTTACCGCATGTAAAGTA

Protein
MKWVTFISLLFLFSSAYSMRCIGMSNRDFVFAVSGGSWVDIVLEHGSCVTTLAKNKPT
LDFELIKTEAKQPATLRKYCIEAKLTNTTTESRCPTQGEPSLNEEQDKRFVCKHSMVDR
GWGNGCGLFGKGGIVTCAMFRCKKNMEGKVVQPENLEYTIVITPHSGEEHAVGNDTG
KHGKEIKITPQSSITEAELTGYGTVTMECSPRTGLDFNEMVLLQMENKAWLVHRQWFL
DLPLPWLPGADTQGSNWIQKETLVTFKNPHAKKQDVVVLGSQEGAMHTALTGATEIQ
MSSGNLLFTGHLKCRLRMDKLQLKGMSYSMCTGKFKVVKEIAETQHGTIVIRVQYEGD
GSPCKIPFEIMDLEKRHVLGRLITVNPIVTEKDSPVNIEAEPPFGDSYIIIGVEPGQLKLN
WFKKSRGSSGGSEQKLISEEDLGSGGGSSVGIIAGLVLLGAVITGAVVAAVMWRRKSS
DRKGGSYTQAASSDSAQGSDVSLTACKV

pD2sE_Dsp_UndPk5

DNA
ATGAAGTGGGTAACCTTTATTTCCCTTCTTTTTCTCTTTAGCTCGGCTTATTCCATGA
GGTGTATCGGCATGTCCAACAGGGACTTTGTGGAGGGAGTGAGCGGCGGCAGCT
GGGTGGACATTGTGCTGGAGCATGGAAGCTGCGTGACCACGATGGCGAAAAACAA
GCCCACCCTGGACTTCGAGCTCATCAAGACAGAGGCTAAACAGCCCGCCACCCTC
AGGAAGTACTGCATCGAGGCCAAGCTGACCAACACAACAACCGAGTCCAGATGCC
CTACACAGGGCGAACCCAGCCTCAACGAAGAGCAGGACAAGAGGTTCGTGTGCAA
ACACAGCATGGTGGACAGGGGCTGGGGCAATGGATGCGGACTCTTCGGAAAAGG
CGGCATCGTGACCTGCGCCATGTTCAGGTGTAAAAAGAACATGGAAGGCAAGGTG
GTGCAGCCCGAAAATCTGGAGTATACCATCGTGATTACCCCCCACAGCGGAGAGG
AGCACGCCGTGGGCAATGACACCGGCAAGCACGGCAAAGAGATTAAGATCACCC
CCCAGTCCTCCATTACCGAAGCTGAACTGACAGGCTACGGCACCGTGACAATGGA
GTGTAGCCCCAGGACCGGACTGGATTTCAACGAGATGGTCCTGCTACAGATGGAG
AACAAGGCCTGGCTCGTGCACAGGCAATGGTTTCTGGATCTGCCTCTGCCTTGGC
TGCCTGGCGCCGATACACAGGGCTCCAACTGGATACAGAAAGAGACCCTCGTCAC
CTTCAAGAATCCCCATGCTAAGAAGCAGGACGTGGTGGTGCTGGGCAGCCAAGAA
GGCGCCATGCACACAGCCCTGACCGGAGCTACCGAGATCCAGATGAGCTCCGGC
AACCTGCTGTTCACCGGCCATCTGAAATGTAGGCTGAGGATGGATAAGCTGCAAC
TCAAAGGCATGTCCTACTCCATGTGCACCGGAAAGTTCAAGGTGGTGAAAGAAATC
GCCGAAACACAGCACGGCACCATCGTGATCAGGGTGCAGTATGAGGGCGACGGC
TCCCCCTGTAAGATCCCCTTCGAAATCATGGACCTGGAAAAGAGGCACGTGCTGG
GCAGGCTCATCACCGTGAACCCCATTGTCTACGAGAAAGACTCCCCCGTGAACAT
CGAGGCCGAGCCTCCCTTTGGCGACTCCTACATCATCATTGGCGTGGAGCCCGGA
CAGCTCAAGCTGAACTGGTTCAAGAAGTCTAGAGGCAGCAGCGGCGGCAGCGAA
CAAAAACTCATCTCAGAAGAGGATCTGGGAAGTGGAGGCGGGTCCTCTGTAGGAA
TCATTGCGGGACTTGTTCTGCTCGGGGCCGTAATAACTGGAGCGGTCGTGGCGGC
TGTGATGTGGCGACGGAAAAGCAGTGATCGGAAGGGTGGTTCATATACCCAAGCC
GCTTCTAGCGATAGCGCACAAGGATCAGATGTTTCTCTTACCGCATGTAAAGTA

Protein
MKWVTFISLLFLFSSAYSMRCIGMSNRDFVEGVSGGSWVDIVLEHGSCVTTMAKNKPT
LDFELIKTEAKQPATLRKYCIEAKLTNTTTESRCPTQGEPSLNEEQDKRFVCKHSMVDR
GWGNGCGLFGKGGIVTCAMFRCKKNMEGKVVQPENLEYTIVITPHSGEEHAVGNDTG
KHGKEIKITPQSSITEAELTGYGTVTMECSPRTGLDFNEMVLLQMENKAWLVHRQWFL
DLPLPWLPGADTQGSNWIQKETLVTFKNPHAKKQDVVVLGSQEGAMHTALTGATEIQ
MSSGNLLFTGHLKCRLRMDKLQLKGMSYSMCTGKFKVVKEIAETQHGTIVIRVQYEGD
GSPCKIPFEIMDLEKRHVLGRLITVNPIVYEKDSPVNIEAEPPFGDSYIIIGVEPGQLKLN
WFKKSRGSSGGSEQKLISEEDLGSGGGSSVGIIAGLVLLGAVITGAVVAAVMWRRKSS
DRKGGSYTQAASSDSAQGSDVSLTACKV

pD2sE_Dsp_UndPk6

DNA
ATGAAGTGGGTAACCTTTATTTCCCTTCTTTTTCTCTTTAGCTCGGCTTATTCCATGA
GGTGTATCGGCATGTCCAACAGGGACTTTGTGGAGGGAGTGAGCGGCGGCAGCT
GGGTGGACATTGTGCTGGAGCATGGAAGCTGCGTGACCACGATGGCGAAAAACAA
GCCCACCCTGGACTTCGAGCTCATCAAGACAGAGGCTAAACAGCCCGCCACCCTC
AGGAAGTACTGCATCGAGGCCAAGCTGACCAACACAACAACCGAGTCCAGATGCC
CTACACAGGGCGAACCCAGCCTCAACGAAGAGCAGGACAAGAGGTTCGTGTGCAA
ACACAGCATGGTGGACAGGGGCTGGGGCAATGGATGCGGACTCTTCGGAAAAGG
CGGCATCGTGACCTGCGCCATGTTCAGGTGTAAAAAGAACATGGAAGGCAAGGTG
GTGCAGCCCGAAAATCTGGAGTATACCATCGTGATTACCCCCCACAGCGGAGAGG
AGCACGCCGTGGGCAATGACACCGGCAAGCACGGCAAAGAGATTAAGATCACCC
CCCAGTCCTCCATTACCGAAGCTGAACTGACAGGCTACGGCACCGTGACAATGGA
GTGTAGCCCCAGGACCGGACTGGATTTCAACGAGATGGTCCTGCTACAGATGGAG
AACAAGGCCTGGCTCGTGCACAGGCAATGGTTTCTGGATCTGCCTCTGCCTTGGC
TGCCTGGCGCCGATACACAGGGCTCCAACTGGATACAGAAAGAGACCCTCGTCAC
CTTCAAGAATCCCCATGCTAAGAAGCAGGACGTGGTGGTGCTGGGCAGCCAAGAA
GGCGCCATGCACACAGCCCTGACCGGAGCTACCGAGATCCAGATGAGCTCCGGC
AACCTGCTGTGGCCCGGCCATCTGAAATGTAGGCTGAGGATGGATAAGCTGCAAC
TCAAAGGCATGTCCTACTCCATGTGCACCGGAAAGTTCAAGGTGGTGAAAGAAATC
GCCGAAACACAGCACGGCACCATCGTGATCAGGGTGCAGTATGAGGGCGACGGC
TCCCCCTGTAAGATCCCCTTCGAAATCATGGACCTGGAAAAGAGGCACGTGCTGG
GCAGGCTCATCACCGTGAACCCCATTGTCACAGAGAAAGACTCCCCCGTGAACAT
CGAGGCCGAGCCTCCCTTTGGCGACTCCTACATCATCATTGGCGTGGAGCCCGGA
CAGCTCAAGCTGAACTGGTTCAAGAAGTCTAGAGGCAGCAGCGGCGGCAGCGAA
CAAAAACTCATCTCAGAAGAGGATCTGGGAAGTGGAGGCGGGTCCTCTGTAGGAA
TCATTGCGGGACTTGTTCTGCTCGGGGCCGTAATAACTGGAGCGGTCGTGGCGGC
TGTGATGTGGCGACGGAAAAGCAGTGATCGGAAGGGTGGTTCATATACCCAAGCC
GCTTCTAGCGATAGCGCACAAGGATCAGATGTTTCTCTTACCGCATGTAAAGTA

Protein
MKWVTFISLLFLFSSAYSMRCIGMSNRDFVEGVSGGSWVDIVLEHGSCVTTMAKNKPT
LDFELIKTEAKQPATLRKYCIEAKLTNTTTESRCPTQGEPSLNEEQDKRFVCKHSMVDR
GWGNGCGLFGKGGIVTCAMFRCKKNMEGKVVQPENLEYTIVITPHSGEEHAVGNDTG
KHGKEIKITPQSSITEAELTGYGTVTMECSPRTGLDFNEMVLLQMENKAWLVHRQWFL
DLPLPWLPGADTQGSNWIQKETLVTFKNPHAKKQDVVVLGSQEGAMHTALTGATEIQ
MSSGNLLWPGHLKCRLRMDKLQLKGMSYSMCTGKFKVVKEIAETQHGTIVIRVQYEG
DGSPCKIPFEIMDLEKRHVLGRLITVNPIVTEKDSPVNIEAEPPFGDSYIIIGVEPGQLKLN
WFKKSRGSSGGSEQKLISEEDLGSGGGSSVGIIAGLVLLGAVITGAVVAAVMWRRKSS
DRKGGSYTQAASSDSAQGSDVSLTACKV

pD2sE_Dsp_UndPk7

DNA
ATGAAGTGGGTAACCTTTATTTCCCTTCTTTTTCTCTTTAGCTCGGCTTATTCCATGA
GGTGTATCGGCATGTCCAACAGGGACTTTGTGGAGGGAGTGAGCGGCGGCAGCT
GGGTGGACATTGTGCTGGAGCATGGAAGCTGCGTGACCACGATGGCGAAAAACAA
GCCCACCCTGGACTTCGAGCTCATCAAGATCGAGGCTAAACAGCCCGCCACCCTC
AGGAAGTACTGCATCGAGGCCAAGCTGACCAACACAACAACCGAGTCCAGATGCC
CTACACAGGGCGAACCCAGCCTCAACGAAGAGCAGGACAAGAGGTTCGTGTGCAA
ACACAGCATGGTGGACAGGGGCTGGGGCAATGGATGCGGACTCTTCGGAAAAGG
CGGCATCGTGACCTGCGCCATGTTCAGGTGTAAAAAGAACATGGAAGGCAAGGTG
GTGCAGCCCGAAAATCTGGAGTATACCATCGTGATTACCCCCCACAGCGGAGAGG
AGCACGCCGTGGGCAATGACACCGGCAAGCACGGCAAAGAGATTAAGATCACCC
CCCAGTCCTCCATTACCGAAGCTGAACTGACAGGCTACGGCACCGTGACAATGGA
GTGTAGCCCCAGGACCGGACTGGATTTCAACGAGATGGTCCTGCTACAGATGGAG
AACAAGGCCTGGCTCGTGCACAGGCAATGGTTTCTGGATCTGCCTCTGCCTTGGC
TGCCTGGCGCCGATACACAGGGCTCCAACTGGATACAGAAAGAGACCCTCGTCAC
CTTCAAGAATCCCCATGCTAAGAAGCAGGACGTGGTGGTGCTGGGCAGCCAAGAA
GGCGCCATGCACACAGCCCTGACCGGAGCTACCGAGATCCAGATGAGCTCCGGC
AACATGCTGTTCACCGGCCATCTGAAATGTAGGCTGAGGATGGATAAGCTGCAACT
CAAAGGCATGTCCTACTCCATGTGCACCGGAAAGTTCAAGGTGGTGAAAGAAATC
GCCGAAACACAGCACGGCACCATCGTGATCAGGGTGCAGTATGAGGGCGACGGC
TCCCCCTGTAAGATCCCCTTCGAAATCATGGACCTGGAAAAGAGGCACGTGCTGG
GCAGGCTCATCACCGTGAACCCCATTGTCACAGAGAAAGACTCCCCCGTGAACAT
CGAGGCCGAGCCTCCCTTTGGCGACTCCTACATCATCATTGGCGTGGAGCCCGGA
CAGCTCAAGCTGAACTGGTTCAAGAAGTCTAGAGGCAGCAGCGGCGGCAGCGAA
CAAAAACTCATCTCAGAAGAGGATCTGGGAAGTGGAGGCGGGTCCTCTGTAGGAA
TCATTGCGGGACTTGTTCTGCTCGGGGCCGTAATAACTGGAGCGGTCGTGGCGGC
TGTGATGTGGCGACGGAAAAGCAGTGATCGGAAGGGTGGTTCATATACCCAAGCC
GCTTCTAGCGATAGCGCACAAGGATCAGATGTTTCTCTTACCGCATGTAAAGTA

Protein
MKWVTFISLLFLFSSAYSMRCIGMSNRDFVEGVSGGSWVDIVLEHGSCVTTMAKNKPT
LDFELIKIEAKQPATLRKYCIEAKLTNTTTESRCPTQGEPSLNEEQDKRFVCKHSMVDR
GWGNGCGLFGKGGIVTCAMFRCKKNMEGKVVQPENLEYTIVITPHSGEEHAVGNDTG
KHGKEIKITPQSSITEAELTGYGTVTMECSPRTGLDFNEMVLLQMENKAWLVHRQWFL
DLPLPWLPGADTQGSNWIQKETLVTFKNPHAKKQDVVVLGSQEGAMHTALTGATEIQ
MSSGNMLFTGHLKCRLRMDKLQLKGMSYSMCTGKFKVVKEIAETQHGTIVIRVQYEG
DGSPCKIPFEIMDLEKRHVLGRLITVNPIVTEKDSPVNIEAEPPFGDSYIIIGVEPGQLKLN
WFKKSRGSSGGSEQKLISEEDLGSGGGSSVGIIAGLVLLGAVITGAVVAAVMWRRKSS
DRKGGSYTQAASSDSAQGSDVSLTACKV

pD2sE_Dsp_UndPk8

DNA
ATGAAGTGGGTAACCTTTATTTCCCTTCTTTTTCTCTTTAGCTCGGCTTATTCCATGA
GGTGTATCGGCATGTCCAACAGGGACTTTGTGGAGGGAGTGAGCGGCGGCAGCT
GGGTGGACATTGTGCTGGAGCATGGAAGCTGCGTGACCACGATGGCGAAAAACAA
GCCCACCCTGGACTTCGAGCTCATCAAGACAGAGGCTAAACAGCCCGCCACCCTC
AGGAAGTACTGCATCGAGGCCAAGCTGACCAACACAACAACCGAGTCCAGATGCC
CTACACAGGGCGAACCCAGCCTCAACGAAGAGCAGGACAAGAGGTTCGTGTGCAA
ACACAGCATGGTGGACAGGGGCTGGGGCAATGGATGCGGACTCTTCGGAAAAGG
CGGCATCGTGACCTGCGCCATGTTCAGGTGTAAAAAGAACATGGAAGGCAAGGTG
GTGCAGCCCGAAAATCTGGAGTATACCATCGTGATTACCCCCCACAGCGGAGAGG
AGCACGCCGTGGGCAATGACACCGGCAAGCACGGCAAAGAGATTAAGATCACCC
CCCAGTCCTCCATTACCGAAGCTGAACTGACAGGCTACGGCACCGTGACAATGGA
GTGTAGCCCCAGGACCGGACTGGATTTCAACGAGATGGTCCTGCTACAGATGGAG
AACAAGGCCTGGCTCGTGCACAGGCAATGGTTTCTGGATCTGCCTCTGCCTTGGC
TGCCTGGCGCCGATACACAGGGCTCCAACTGGATACAGAAAGAGACCCTCGTCAC
CTTCAAGAATCCCCATGCTAAGAAGCAGGACGTGGTGGTGCTGGGCAGCTACGAA
GCCTGGCTGCACACAGCCCTGACCGGAGCTACCGAGATCCAGATGAGCTCCGGC
AACCTGCTGTTCACCGGCCATCTGAAATGTAGGCTGAGGATGGATAAGCTGCAAC
TCAAAGGCATGTCCTACTCCATGTGCACCGGAAAGTTCAAGGTGGTGAAAGAAATC
GCCGAAACACAGCACGGCACCATCGTGATCAGGGTGCAGTATGAGGGCGACGGC
TCCCCCTGTAAGATCCCCTTCGAAATCATGGACCTGGAAAAGAGGCACGTGCTGG
GCAGGCTCATCACCGTGAACCCCATTGTCACAGAGAAAGACTCCCCCGTGAACAT
CGAGGCCGAGCCTCCCTTTGGCGACTCCTACATCATCATTGGCGTGGAGCCCGGA
CAGCTCAAGCTGAACTGGTTCAAGAAGTCTAGAGGCAGCAGCGGCGGCAGCGAA
CAAAAACTCATCTCAGAAGAGGATCTGGGAAGTGGAGGCGGGTCCTCTGTAGGAA
TCATTGCGGGACTTGTTCTGCTCGGGGCCGTAATAACTGGAGCGGTCGTGGCGGC
TGTGATGTGGCGACGGAAAAGCAGTGATCGGAAGGGTGGTTCATATACCCAAGCC
GCTTCTAGCGATAGCGCACAAGGATCAGATGTTTCTCTTACCGCATGTAAAGTA

Protein
MKWVTFISLLFLFSSAYSMRCIGMSNRDFVEGVSGGSWVDIVLEHGSCVTTMAKNKPT
LDFELIKTEAKQPATLRKYCIEAKLTNTTTESRCPTQGEPSLNEEQDKRFVCKHSMVDR
GWGNGCGLFGKGGIVTCAMFRCKKNMEGKVVQPENLEYTIVITPHSGEEHAVGNDTG
KHGKEIKITPQSSITEAELTGYGTVTMECSPRTGLDFNEMVLLQMENKAWLVHRQWFL
DLPLPWLPGADTQGSNWIQKETLVTFKNPHAKKQDVVVLGSYEAWLHTALTGATEIQ
MSSGNLLFTGHLKCRLRMDKLQLKGMSYSMCTGKFKVVKEIAETQHGTIVIRVQYEGD
GSPCKIPFEIMDLEKRHVLGRLITVNPIVTEKDSPVNIEAEPPFGDSYIIIGVEPGQLKLN
WFKKSRGSSGGSEQKLISEEDLGSGGGSSVGIIAGLVLLGAVITGAVVAAVMWRRKSS
DRKGGSYTQAASSDSAQGSDVSLTACKV

pD2sE_Dsp_SHP1

DNA
ATGAAGTGGGTAACCTTTATTTCCCTTCTTTTTCTCTTTAGCTCGGCTTATTCCATGA
GGTGTATCGGCATGTCCAACAGGGACTTTGTGGAGGGAGAGAGCGGCGGCAGCT
GGGTGGACATTGTGCTGGAGCATGGAAGCTGCGTGACCACGATGGCGAAAAACAA
GCCCACCCTGGACTTCGAGCTCATCAAGACAGAGGCTAAACAGCCCGCCACCCTC
AGGAAGTACTGCATCGAGGCCAAGCTGACCAACACAACAACCGAGTCCAGATGCC
CTACACAGGGCGAACCCAGCCTCAACGAAGAGCAGGACAAGAGGTTCGTGTGCAA
ACACAGCATGGTGGACAGGGGCTGGGGCAATGGATGCGGACTCTTCGGAAAAGG
CGGCATCGTGACCTGCGCCATGTTCAGGTGTAAAAAGAACATGGAAGGCAAGGTG
GTGCAGCCCGAAAATCTGGAGTATACCATCGTGATTACCCCCCACAGCGGAGAGG
AGCACGCCGTGGGCAATGACACCGGCAAGCACGGCAAAGAGATTAAGATCACCC
CCCAGTCCTCCATTACCGAAGCTGAACTGACAGGCTACGGCACCGTGACAATGGA
GTGTAGCCCCAGGACCGGACTGGATTTCAACGAGATGGTCCTGCTACAGATGGAG
AACAAGGCCTGGCTCGTGCACAGGCAATGGTTTCTGGATCTGCCTCTGCCTTGGC
TGCCTGGCGCCGATACACAGGGCTCCAACTGGATACAGAAAGAGACCCTCGTCAC
CTTCAAGAATCCCCATGCTAAGAAGCAGGACGTGGTGGTGCTGGGCAGCCAAGAA
GGCGCCATGCACACAGCCCTGACCGGAGCTACCGAGATCCAGATGAGCTCCGGC
AACCTGCTGTTCACCGGCCATCTGAAATGTAGGCTGAGGATGGATAAGCTGCAAC
TCAAAGGCATGTCCTACTCCATGTGCACCGGAAAGTTCAAGGTGGTGAAAGAAATC
GCCGAAACACAGCACGGCACCATCGTGATCAGGGTGCAGTATGAGGGCGACGGC
TCCCCCTGTAAGATCCCCTTCGAAATCATGGACCTGGAAAAGAGGCACGTGCTGG
GCAGGCTCATCACCGTGAACCCCATTGTCACAGAGAAAGACTCCCCCGTGAACAT
CGAGGCCGAGCCTCCCGACGGCGACTCCTACATCATCATTGGCGTGGAGCCCGG
ACAGCTCAAGCTGAACTGGCGGAAGAAGTCTAGAGGCAGCAGCGGCGGCAGCGA
ACAAAAACTCATCTCAGAAGAGGATCTGGGAAGTGGAGGCGGGTCCTCTGTAGGA
ATCATTGCGGGACTTGTTCTGCTCGGGGCCGTAATAACTGGAGCGGTCGTGGCGG
CTGTGATGTGGCGACGGAAAAGCAGTGATCGGAAGGGTGGTTCATATACCCAAGC
CGCTTCTAGCGATAGCGCACAAGGATCAGATGTTTCTCTTACCGCATGTAAAGTA

Protein
MKWVTFISLLFLFSSAYSMRCIGMSNRDFVEGESGGSWVDIVLEHGSCVTTMAKNKPT
LDFELIKTEAKQPATLRKYCIEAKLTNTTTESRCPTQGEPSLNEEQDKRFVCKHSMVDR
GWGNGCGLFGKGGIVTCAMFRCKKNMEGKVVQPENLEYTIVITPHSGEEHAVGNDTG
KHGKEIKITPQSSITEAELTGYGTVTMECSPRTGLDFNEMVLLQMENKAWLVHRQWFL
DLPLPWLPGADTQGSNWIQKETLVTFKNPHAKKQDVVVLGSQEGAMHTALTGATEIQ
MSSGNLLFTGHLKCRLRMDKLQLKGMSYSMCTGKFKVVKEIAETQHGTIVIRVQYEGD
GSPCKIPFEIMDLEKRHVLGRLITVNPIVTEKDSPVNIEAEPPDGDSYIIIGVEPGQLKLN
WRKKSRGSSGGSEQKLISEEDLGSGGGSSVGIIAGLVLLGAVITGAVVAAVMWRRKSS
DRKGGSYTQAASSDSAQGSDVSLTACKV

pD2sE_Dsp_SHP2

DNA
ATGAAGTGGGTAACCTTTATTTCCCTTCTTTTTCTCTTTAGCTCGGCTTATTCCATGA
GGTGTATCGGCATGTCCAACAGGGACTTTGTGGAGGGAGAGAGCGGCGGCAGCC
ACGTGGACATTGTGCTGGAGCATGGAAGCTGCGTGACCACGATGGCGAAAAACAA
GCCCACCCTGGACTTCGAGCTCATCAAGACAGAGGCTAAACAGCCCGCCACCCTC
AGGAAGTACTGCATCGAGGCCAAGCTGACCAACACAACAACCGAGTCCAGATGCC
CTACACAGGGCGAACCCAGCCTCAACGAAGAGCAGGACAAGAGGTTCGTGTGCAA
ACACAGCATGGTGGACAGGGGCTGGGGCAATGGATGCGGACTCTTCGGAAAAGG
CGGCATCGTGACCTGCGCCATGTTCAGGTGTAAAAAGAACATGGAAGGCAAGGTG
GTGCAGCCCGAAAATCTGGAGTATACCATCGTGATTACCCCCCACAGCGGAGAGG
AGCACGCCGTGGGCAATGACACCGGCAAGCACGGCAAAGAGATTAAGATCACCC
CCCAGTCCTCCATTACCGAAGCTGAACTGACAGGCTACGGCACCGTGACAATGGA
GTGTAGCCCCAGGACCGGACTGGATTTCAACGAGATGGTCCTGCTACAGATGGAG
AACAAGGCCTGGCTCGTGCACAGGCAATGGTTTCTGGATCTGCCTCTGCCTTGGC
TGCCTGGCGCCGATACACAGGGCTCCAACTGGATACAGAAAGAGACCCTCGTCAC
CTTCAAGAATCCCCATGCTAAGAAGCAGGACGTGGTGGTGCTGGGCAGCCAAGAA
GGCGCCATGCACACAGCCCTGACCGGAGCTACCGAGATCCAGATGAGCTCCGGC
AACCTGCTGTTCACCGGCCATCTGAAATGTAGGCTGAGGATGGATAAGCTGCAAC
TCAAAGGCATGTCCTACTCCATGTGCACCGGAAAGTTCAAGGTGGTGAAAGAAATC
GCCGAAACACAGCACGGCACCATCGTGATCAGGGTGCAGTATGAGGGCGACGGC
TCCCCCTGTAAGATCCCCTTCGAAATCATGGACCTGGAAAAGAGGCACGTGCTGG
GCAGGCTCATCACCGTGAACCCCATTGTCACAGAGAAAGACTCCCCCGTGAACAT
CGAGGCCGAGCCTCCCGACGGCGACTCCTACATCATCATTGGCGTGGAGCCCGG
ACAGCTCAAGCTGAACTGGCGGAAGAAGTCTAGAGGCAGCAGCGGCGGCAGCGA
ACAAAAACTCATCTCAGAAGAGGATCTGGGAAGTGGAGGCGGGTCCTCTGTAGGA
ATCATTGCGGGACTTGTTCTGCTCGGGGCCGTAATAACTGGAGCGGTCGTGGCGG
CTGTGATGTGGCGACGGAAAAGCAGTGATCGGAAGGGTGGTTCATATACCCAAGC
CGCTTCTAGCGATAGCGCACAAGGATCAGATGTTTCTCTTACCGCATGTAAAGTA

Protein
MKWVTFISLLFLFSSAYSMRCIGMSNRDFVEGESGGSHVDIVLEHGSCVTTMAKNKPT
LDFELIKTEAKQPATLRKYCIEAKLTNTTTESRCPTQGEPSLNEEQDKRFVCKHSMVDR
GWGNGCGLFGKGGIVTCAMFRCKKNMEGKVVQPENLEYTIVITPHSGEEHAVGNDTG
KHGKEIKITPQSSITEAELTGYGTVTMECSPRTGLDFNEMVLLQMENKAWLVHRQWFL
DLPLPWLPGADTQGSNWIQKETLVTFKNPHAKKQDVVVLGSQEGAMHTALTGATEIQ
MSSGNLLFTGHLKCRLRMDKLQLKGMSYSMCTGKFKVVKEIAETQHGTIVIRVQYEGD
GSPCKIPFEIMDLEKRHVLGRLITVNPIVTEKDSPVNIEAEPPDGDSYIIIGVEPGQLKLN
WRKKSRGSSGGSEQKLISEEDLGSGGGSSVGIIAGLVLLGAVITGAVVAAVMWRRKSS
DRKGGSYTQAASSDSAQGSDVSLTACKV

pD2sE_Dsp_Mnmer1

DNA
ATGAAGTGGGTAACCTTTATTTCCCTTCTTTTTCTCTTTAGCTCGGCTTATTCCATGA
GGTGTATCGGCATGTCCAACAGGGACTTTGTGGAGGGAGTGAGCGGCGGCAGCT
GGGTGGACATTGTGCTGGAGCATGGAAGCTGCGTGACCACGATGGCGAAAAACAA
GCCCACCCTGGACTTCGAGCTCATCAAGACAGAGGCTAAACAGCCCGCCACCCTC
AGGAAGTACTGCATCGAGGCCAAGCTGACCAACACAACAACCGAGTCCAGATGCC
CTACACAGGGCGAACCCAGCCTCAACGAAGAGCAGGACAAGAGGTTCGTGTGCAA
ACACAGCATGGTGGACAGGGGCTGGGGCAATGGATGCGGACTCTTCGGAAAAGG
CGGCATCGTGACCTGCGCCATGTTCAGGTGTAAAAAGAACATGGAAGGCAAGGTG
GTGCAGCCCGAAAATCTGGAGTATACCATCGTGATTACCCCCCACAGCGGAGAGG
AGCACGCCGTGGGCAATGACACCGGCAAGCACGGCAAAGAGATTAAGATCACCC
CCCAGTCCTCCATTACCGAAGCTGAACTGACAGGCTACGGCACCGTGACAATGGA
GTGTAGCCCCAGGACCGGACTGGATTTCAACGAGATGGTCCTGCTACAGATGGAG
AACAAGGCCTGGCTCGTGCACAGGCAATGGTTTCTGGATCTGCCTCTGCCTTGGC
TGCCTGGCGCCGATACACAGGGCTCCAACTGGATACAGAAAGAGACCCTCGTCAC
CTTCAAGAATCCCCATGCTAAGAAGCAGGACGTGGTGGTGCTGGGCGAACAAGAA
GGCGCCATGCACACAGCCCTGACCGGAGCTACCGAGATCCAGATGAGCTCCGGC
AACCTGCTGTTCACCGGCCATCTGAAATGTAGGCTGAGGATGGATAAGCTGCAAC
TCAAAGGCATGTCCTACTCCATGTGCACCGGAAAGTTCAAGGTGGTGAAAGAAATC
GCCGAAACACAGCACGGCACCATCGTGATCAGGGTGCAGTATGAGGGCGACGGC
TCCCCCTGTAAGATCCCCTTCGAAATCATGGACCTGGAAAAGAGGCACGTGCTGG
GCAGGCTCATCACCGTGAACCCCATTGTCACAGAGAAAGACTCCCCCGTGAACAT
CGAGGCCGAGCCTCCCTTTGGCGACTCCTACATCATCATTGGCGTGGAGCCCGGA
CAGCTCAAGCTGAACTGGTTCAAGAAGTCTAGAGGCAGCAGCGGCGGCAGCGAA
CAAAAACTCATCTCAGAAGAGGATCTGGGAAGTGGAGGCGGGTCCTCTGTAGGAA
TCATTGCGGGACTTGTTCTGCTCGGGGCCGTAATAACTGGAGCGGTCGTGGCGGC
TGTGATGTGGCGACGGAAAAGCAGTGATCGGAAGGGTGGTTCATATACCCAAGCC
GCTTCTAGCGATAGCGCACAAGGATCAGATGTTTCTCTTACCGCATGTAAAGTA

Protein
MKWVTFISLLFLFSSAYSMRCIGMSNRDFVEGVSGGSWVDIVLEHGSCVTTMAKNKPT
LDFELIKTEAKQPATLRKYCIEAKLTNTTTESRCPTQGEPSLNEEQDKRFVCKHSMVDR
GWGNGCGLFGKGGIVTCAMFRCKKNMEGKVVQPENLEYTIVITPHSGEEHAVGNDTG
KHGKEIKITPQSSITEAELTGYGTVTMECSPRTGLDFNEMVLLQMENKAWLVHRQWFL
DLPLPWLPGADTQGSNWIQKETLVTFKNPHAKKQDVVVLGEQEGAMHTALTGATEIQ
MSSGNLLFTGHLKCRLRMDKLQLKGMSYSMCTGKFKVVKEIAETQHGTIVIRVQYEGD
GSPCKIPFEIMDLEKRHVLGRLITVNPIVTEKDSPVNIEAEPPFGDSYIIIGVEPGQLKLN
WFKKSRGSSGGSEQKLISEEDLGSGGGSSVGIIAGLVLLGAVITGAVVAAVMWRRKSS
DRKGGSYTQAASSDSAQGSDVSLTACKV

pD2sE_Dsp_Mnmer2

DNA

ATGAAGTGGGTAACCTTTATTTCCCTTCTTTTTCTCTTTAGCTCGGCTTATTCCATGA
GGTGTATCGGCATGTCCAACAGGGACTTTGTGGAGGGAGTGAGCGGCGGCAGCT
GGGTGGACATTGTGCTGGAGCATGGAAGCTGCGTGACCACGATGGCGAAAAACAA
GCCCACCCTGGACTTCGAGCTCATCAAGACAGAGGCTAAACAGCCCGCCACCCTC
AGGAAGTACTGCATCGAGGCCAAGCTGACCAACACAACAACCGAGTCCAGATGCC
CTACACAGGGCGAACCCAGCCTCAACGAAGAGCAGGACAAGAGGTTCGTGTGCAA
ACACAGCATGGTGGACAGGGGCTGGGGCAATGGATGCGGACTCTTCGGAAAAGG
CGGCATCGTGACCTGCGCCATGTTCAGGTGTAAAAAGAACATGGAAGGCAAGGTG
GTGCAGCCCGAAAATCTGGAGTATACCATCGTGATTACCCCCCACAGCGGAGAGG
AGCACGCCGTGGGCAATGACACCGGCAAGCACGGCAAAGAGATTAAGATCACCC
CCCAGTCCTCCATTACCGAAGCTGAACTGACAGGCTACGGCACCGTGACAATGGA
GTGTAGCCCCAGGACCGGACTGGATTTCAACGAGATGGTCCTGCTACAGATGGAG
AACAAGGCCTGGCTCGTGCACAGGCAATGGTTTCTGGATCTGCCTCTGCCTTGGC
TGCCTGGCGCCGATACACAGGGCTCCAACTGGATACAGAAAGAGACCCTCGTCAC
CTTCAAGAATCCCCATGCTAAGAAGCAGGACGTGGTGGTGCTGGGCAGCCAAGAA
GAGGCCATGCACACAGCCCTGACCGGAGCTACCGAGATCCAGATGAGCTCCGGC
AACCTGCTGTTCACCGGCCATCTGAAATGTAGGCTGAGGATGGATAAGCTGCAAC
TCAAAGGCATGTCCTACTCCATGTGCACCGGAAAGTTCAAGGTGGTGAAAGAAATC
GCCGAAACACAGCACGGCACCATCGTGATCAGGGTGCAGTATGAGGGCGACGGC
TCCCCCTGTAAGATCCCCTTCGAAATCATGGACCTGGAAAAGAGGCACGTGCTGG
GCAGGCTCATCACCGTGAACCCCATTGTCACAGAGAAAGACTCCCCCGTGAACAT
CGAGGCCGAGCCTCCCTTTGGCGACTCCTACATCATCATTGGCGTGGAGCCCGGA
CAGCTCAAGCTGAACTGGTTCAAGAAGTCTAGAGGCAGCAGCGGCGGCAGCGAA
CAAAAACTCATCTCAGAAGAGGATCTGGGAAGTGGAGGCGGGTCCTCTGTAGGAA
TCATTGCGGGACTTGTTCTGCTCGGGGCCGTAATAACTGGAGCGGTCGTGGCGGC
TGTGATGTGGCGACGGAAAAGCAGTGATCGGAAGGGTGGTTCATATACCCAAGCC
GCTTCTAGCGATAGCGCACAAGGATCAGATGTTTCTCTTACCGCATGTAAAGTA

Protein

MKWVTFISLLFLFSSAYSMRCIGMSNRDFVEGVSGGSWVDIVLEHGSCVTTMAKNKPT
LDFELIKTEAKQPATLRKYCIEAKLTNTTTESRCPTQGEPSLNEEQDKRFVCKHSMVDR
GWGNGCGLFGKGGIVTCAMFRCKKNMEGKVVQPENLEYTIVITPHSGEEHAVGNDTG
KHGKEIKITPQSSITEAELTGYGTVTMECSPRTGLDFNEMVLLQMENKAWLVHRQWFL
DLPLPWLPGADTQGSNWIQKETLVTFKNPHAKKQDVVVLGSQEEAMHTALTGATEIQ
MSSGNLLFTGHLKCRLRMDKLQLKGMSYSMCTGKFKVVKEIAETQHGTIVIRVQYEGD
GSPCKIPFEIMDLEKRHVLGRLITVNPIVTEKDSPVNIEAEPPFGDSYIIIGVEPGQLKLN
WFKKSRGSSGGSEQKLISEEDLGSGGGSSVGIIAGLVLLGAVITGAVVAAVMWRRKSS
DRKGGSYTQAASSDSAQGSDVSLTACKV

pD2sE_Dsp_Cm1 - Previously published disulfide (A259C)
Reference: (Rouvinski et. al 2017, Slon Campos et. al., 2017)

DNA
ATGAAGTGGGTAACCTTTATTTCCCTTCTTTTTCTCTTTAGCTCGGCTTATTCCATGA
GGTGTATCGGCATGTCCAACAGGGACTTTGTGGAGGGAGTGAGCGGCGGCAGCT
GGGTGGACATTGTGCTGGAGCATGGAAGCTGCGTGACCACGATGGCGAAAAACAA
GCCCACCCTGGACTTCGAGCTCATCAAGACAGAGGCTAAACAGCCCGCCACCCTC
AGGAAGTACTGCATCGAGGCCAAGCTGACCAACACAACAACCGAGTCCAGATGCC
CTACACAGGGCGAACCCAGCCTCAACGAAGAGCAGGACAAGAGGTTCGTGTGCAA
ACACAGCATGGTGGACAGGGGCTGGGGCAATGGATGCGGACTCTTCGGAAAAGG
CGGCATCGTGACCTGCGCCATGTTCAGGTGTAAAAAGAACATGGAAGGCAAGGTG
GTGCAGCCCGAAAATCTGGAGTATACCATCGTGATTACCCCCCACAGCGGAGAGG
AGCACGCCGTGGGCAATGACACCGGCAAGCACGGCAAAGAGATTAAGATCACCC
CCCAGTCCTCCATTACCGAAGCTGAACTGACAGGCTACGGCACCGTGACAATGGA
GTGTAGCCCCAGGACCGGACTGGATTTCAACGAGATGGTCCTGCTACAGATGGAG
AACAAGGCCTGGCTCGTGCACAGGCAATGGTTTCTGGATCTGCCTCTGCCTTGGC
TGCCTGGCGCCGATACACAGGGCTCCAACTGGATACAGAAAGAGACCCTCGTCAC
CTTCAAGAATCCCCATGCTAAGAAGCAGGACGTGGTGGTGCTGGGCAGCCAAGAA
GGCTGTATGCACACAGCCCTGACCGGAGCTACCGAGATCCAGATGAGCTCCGGC
AACCTGCTGTTCACCGGCCATCTGAAATGTAGGCTGAGGATGGATAAGCTGCAAC
TCAAAGGCATGTCCTACTCCATGTGCACCGGAAAGTTCAAGGTGGTGAAAGAAATC
GCCGAAACACAGCACGGCACCATCGTGATCAGGGTGCAGTATGAGGGCGACGGC
TCCCCCTGTAAGATCCCCTTCGAAATCATGGACCTGGAAAAGAGGCACGTGCTGG
GCAGGCTCATCACCGTGAACCCCATTGTCACAGAGAAAGACTCCCCCGTGAACAT
CGAGGCCGAGCCTCCCTTTGGCGACTCCTACATCATCATTGGCGTGGAGCCCGGA
CAGCTCAAGCTGAACTGGTTCAAGAAGTCTAGAGGCAGCAGCGGCGGCAGCGAA
CAAAAACTCATCTCAGAAGAGGATCTGGGAAGTGGAGGCGGGTCCTCTGTAGGAA
TCATTGCGGGACTTGTTCTGCTCGGGGCCGTAATAACTGGAGCGGTCGTGGCGGC
TGTGATGTGGCGACGGAAAAGCAGTGATCGGAAGGGTGGTTCATATACCCAAGCC
GCTTCTAGCGATAGCGCACAAGGATCAGATGTTTCTCTTACCGCATGTAAAGTA

Protein
MKWVTFISLLFLFSSAYSMRCIGMSNRDFVEGVSGGSWVDIVLEHGSCVTTMAKNKPT
LDFELIKTEAKQPATLRKYCIEAKLTNTTTESRCPTQGEPSLNEEQDKRFVCKHSMVDR
GWGNGCGLFGKGGIVTCAMFRCKKNMEGKVVQPENLEYTIVITPHSGEEHAVGNDTG
KHGKEIKITPQSSITEAELTGYGTVTMECSPRTGLDFNEMVLLQMENKAWLVHRQWFL
DLPLPWLPGADTQGSNWIQKETLVTFKNPHAKKQDVVVLGSQEGCMHTALTGATEIQ
MSSGNLLFTGHLKCRLRMDKLQLKGMSYSMCTGKFKVVKEIAETQHGTIVIRVQYEGD
GSPCKIPFEIMDLEKRHVLGRLITVNPIVTEKDSPVNIEAEPPFGDSYIIIGVEPGQLKLN
WFKKSRGSSGGSEQKLISEEDLGSGGGSSVGIIAGLVLLGAVITGAVVAAVMWRRKSS
DRKGGSYTQAASSDSAQGSDVSLTACKV

pD2sE_Dsp_Cm2 - Previously published disulfide (L107C/A313C)
Reference: (Rouvinski et. al 2017)

DNA
ATGAAGTGGGTAACCTTTATTTCCCTTCTTTTTCTCTTTAGCTCGGCTTATTCCATGA
GGTGTATCGGCATGTCCAACAGGGACTTTGTGGAGGGAGTGAGCGGCGGCAGCT
GGGTGGACATTGTGCTGGAGCATGGAAGCTGCGTGACCACGATGGCGAAAAACAA
GCCCACCCTGGACTTCGAGCTCATCAAGACAGAGGCTAAACAGCCCGCCACCCTC
AGGAAGTACTGCATCGAGGCCAAGCTGACCAACACAACAACCGAGTCCAGATGCC
CTACACAGGGCGAACCCAGCCTCAACGAAGAGCAGGACAAGAGGTTCGTGTGCAA
ACACAGCATGGTGGACAGGGGCTGGGGCAATGGATGCGGATGTTTCGGAAAAGG
CGGCATCGTGACCTGCGCCATGTTCAGGTGTAAAAAGAACATGGAAGGCAAGGTG
GTGCAGCCCGAAAATCTGGAGTATACCATCGTGATTACCCCCCACAGCGGAGAGG
AGCACGCCGTGGGCAATGACACCGGCAAGCACGGCAAAGAGATTAAGATCACCC
CCCAGTCCTCCATTACCGAAGCTGAACTGACAGGCTACGGCACCGTGACAATGGA
GTGTAGCCCCAGGACCGGACTGGATTTCAACGAGATGGTCCTGCTACAGATGGAG
AACAAGGCCTGGCTCGTGCACAGGCAATGGTTTCTGGATCTGCCTCTGCCTTGGC
TGCCTGGCGCCGATACACAGGGCTCCAACTGGATACAGAAAGAGACCCTCGTCAC
CTTCAAGAATCCCCATGCTAAGAAGCAGGACGTGGTGGTGCTGGGCAGCCAAGAA
GGCGCCATGCACACAGCCCTGACCGGAGCTACCGAGATCCAGATGAGCTCCGGC
AACCTGCTGTTCACCGGCCATCTGAAATGTAGGCTGAGGATGGATAAGCTGCAAC
TCAAAGGCATGTCCTACTCCATGTGCACCGGAAAGTTCAAGGTGGTGAAAGAAATC
TGTGAAACACAGCACGGCACCATCGTGATCAGGGTGCAGTATGAGGGCGACGGC
TCCCCCTGTAAGATCCCCTTCGAAATCATGGACCTGGAAAAGAGGCACGTGCTGG
GCAGGCTCATCACCGTGAACCCCATTGTCACAGAGAAAGACTCCCCCGTGAACAT
CGAGGCCGAGCCTCCCTTTGGCGACTCCTACATCATCATTGGCGTGGAGCCCGGA
CAGCTCAAGCTGAACTGGTTCAAGAAGTCTAGAGGCAGCAGCGGCGGCAGCGAA
CAAAAACTCATCTCAGAAGAGGATCTGGGAAGTGGAGGCGGGTCCTCTGTAGGAA
TCATTGCGGGACTTGTTCTGCTCGGGGCCGTAATAACTGGAGCGGTCGTGGCGGC
TGTGATGTGGCGACGGAAAAGCAGTGATCGGAAGGGTGGTTCATATACCCAAGCC
GCTTCTAGCGATAGCGCACAAGGATCAGATGTTTCTCTTACCGCATGTAAAGTA

Protein
MKWVTFISLLFLFSSAYSMRCIGMSNRDFVEGVSGGSWVDIVLEHGSCVTTMAKNKPT
LDFELIKTEAKQPATLRKYCIEAKLTNTTTESRCPTQGEPSLNEEQDKRFVCKHSMVDR
GWGNGCGCFGKGGIVTCAMFRCKKNMEGKVVQPENLEYTIVITPHSGEEHAVGNDT
GKHGKEIKITPQSSITEAELTGYGTVTMECSPRTGLDFNEMVLLQMENKAWLVHRQWF
LDLPLPWLPGADTQGSNWIQKETLVTFKNPHAKKQDVVVLGSQEGAMHTALTGATEIQ
MSSGNLLFTGHLKCRLRMDKLQLKGMSYSMCTGKFKVVKEICETQHGTIVIRVQYEGD
GSPCKIPFEIMDLEKRHVLGRLITVNPIVTEKDSPVNIEAEPPFGDSYIIIGVEPGQLKLN
WFKKSRGSSGGSEQKLISEEDLGSGGGSSVGIIAGLVLLGAVITGAVVAAVMWRRKSS
DRKGGSYTQAASSDSAQGSDVSLTACKV

Rosetta Design Variant Soluble Expression Vector Constructs (EV8)

pD2sE_EV8 (WT)

DNA
ATGAAGTGGGTAACCTTTATTTCCCTTCTTTTTCTCTTTAGCTCGGCTTATTCCATGA
GGTGTATCGGCATGTCCAACAGGGACTTTGTGGAGGGAGTGAGCGGCGGCAGCT
GGGTGGACATTGTGCTGGAGCATGGAAGCTGCGTGACCACGATGGCGAAAAACAA
GCCCACCCTGGACTTCGAGCTCATCAAGACAGAGGCTAAACAGCCCGCCACCCTC
AGGAAGTACTGCATCGAGGCCAAGCTGACCAACACAACAACCGAGTCCAGATGCC
CTACACAGGGCGAACCCAGCCTCAACGAAGAGCAGGACAAGAGGTTCGTGTGCAA
ACACAGCATGGTGGACAGGGGCTGGGGCAATGGATGCGGACTCTTCGGAAAAGG
CGGCATCGTGACCTGCGCCATGTTCAGGTGTAAAAAGAACATGGAAGGCAAGGTG
GTGCAGCCCGAAAATCTGGAGTATACCATCGTGATTACCCCCCACAGCGGAGAGG
AGCACGCCGTGGGCAATGACACCGGCAAGCACGGCAAAGAGATTAAGATCACCC
CCCAGTCCTCCATTACCGAAGCTGAACTGACAGGCTACGGCACCGTGACAATGGA
GTGTAGCCCCAGGACCGGACTGGATTTCAACGAGATGGTCCTGCTACAGATGGAG
AACAAGGCCTGGCTCGTGCACAGGCAATGGTTTCTGGATCTGCCTCTGCCTTGGC
TGCCTGGCGCCGATACACAGGGCTCCAACTGGATACAGAAAGAGACCCTCGTCAC
CTTCAAGAATCCCCATGCTAAGAAGCAGGACGTGGTGGTGCTGGGCAGCCAAGAA
GGCGCCATGCACACAGCCCTGACCGGAGCTACCGAGATCCAGATGAGCTCCGGC
AACCTGCTGTTCACCGGCCATCTGAAATGTAGGCTGAGGATGGATAAGCTGCAAC
TCAAAGGCATGTCCTACTCCATGTGCACCGGAAAGTTCAAGGTGGTGAAAGAAATC
GCCGAAACACAGCACGGCACCATCGTGATCAGGGTGCAGTATGAGGGCGACGGC
TCCCCCTGTAAGATCCCCTTCGAAATCATGGACCTGGAAAAGAGGCACGTGCTGG
GCAGGCTCATCACCGTGAACCCCATTGTCACAGAGAAAGACTCCCCCGTGAACAT
CGAGGCCGAGCCTCCCTTTGGCGACTCCTACATCATCATTGGCGTGGAGCCCGGA
CAGCTCAAGCTGAACTGGTTCAAGAAGGGCAGCAGCGGCGGCAGCCATCACCAC
CATCATCACCATCAT

Protein
MKWVTFISLLFLFSSAYSMRCIGMSNRDFVEGVSGGSWVDIVLEHGSCVTTMAKNKPT
LDFELIKTEAKQPATLRKYCIEAKLTNTTTESRCPTQGEPSLNEEQDKRFVCKHSMVDR
GWGNGCGLFGKGGIVTCAMFRCKKNMEGKVVQPENLEYTIVITPHSGEEHAVGNDTG
KHGKEIKITPQSSITEAELTGYGTVTMECSPRTGLDFNEMVLLQMENKAWLVHRQWFL
DLPLPWLPGADTQGSNWIQKETLVTFKNPHAKKQDVVVLGSQEGAMHTALTGATEIQ
MSSGNLLFTGHLKCRLRMDKLQLKGMSYSMCTGKFKVVKEIAETQHGTIVIRVQYEGD
GSPCKIPFEIMDLEKRHVLGRLITVNPIVTEKDSPVNIEAEPPFGDSYIIIGVEPGQLKLN
WFKKGSSGGSHHHHHHHH

pD2sE_EV8_HCat2

DNA
ATGAAGTGGGTAACCTTTATTTCCCTTCTTTTTCTCTTTAGCTCGGCTTATTCCATGA
TGTGTATCGGCATGTCCAACAGGGACTTTGTGGAGGGAGTGAGCGGCGGCAGCT
GGGTGGACATTGTGCTGGAGCATGGAAGCTGCGTGACCACGATGGCGAAAAACAA
GCCCACCCTGGACTTCATGCTCATCAAGACAGAGGCTAAACAGCCCGCCACCCTC
AGGAAGTACTGCATCGAGGCCAAGCTGACCAACACAACAACCGAGTCCAGATGCC
CTACACAGGGCGAACCCAGCCTCAACGAAGAGCAGGACAAGAGGTTCGTGTGCAA
ACACAGCATGGTGGACAGGGGCTGGGGCAATGGATGCGGACTCTTCGGAAAAGG
CGGCATCGTGACCTGCGCCATGTTCAGGTGTAAAAAGAACATGGAAGGCAAGGTG
GTGCAGCCCGAAAATCTGGAGTATACCATCGTGATTACCCCCCACAGCGGAGAGG
AGCACGCCGTGGGCAATGACACCGGCAAGCACGGCAAAGAGATTAAGATCACCC
CCCAGTCCTCCATTACCGAAGCTGAACTGACAGGCTACGGCACCGTGACAATGGA
GTGTAGCCCCAGGACCGGACTGGATTTCAACGAGATGGTCCTGCTACAGATGGAG
AACAAGGCCTGGCTCGTGCACAGGCAATGGTTTCTGGATCTGCCTCTGCCTTGGC
TGCCTGGCGCCGATACACAGGGCTCCAACTGGATACAGAAAGAGACCCTCGTCAC
CTTCAAGAATCCCCATGCTAAGAAGCAGGACGTGGTGGTGCTGGGCAGCCAAGAA
GGCGCCATGCACACAGCCCTGACCGGAGCTACCGAGATCCAGATGAGCTCCGGC
AACCTGCTGTTCACCGGCCATCTGAAATGTAGGCTGAGGATGGATAAGCTGCAAC
TCAAAGGCATGTCCTACTCCATGTGCACCGGAAAGTTCAAGGTGGTGAAAGAAATC
GCCGAAACACAGCACGGCACCATCGTGATCAGGGTGCAGTATGAGGGCGACGGC
TCCCCCTGTAAGATCCCCTTCGAAATCATGGACCTGGAAAAGAGGCACGTGCTGG
GCAGGCTCATCACCGTGAACCCCATTGTCACAGAGAAAGACTCCCCCGTGAACAT
CGAGGCCGAGCCTCCCTTTGGCGACTCCTACATCATCATTGGCGTGGAGCCCGGA
CAGCTCAAGCTGAACTGGTTCAAGAAGGGCAGCAGCGGCGGCAGCCATCACCAC
CATCATCACCATCAT

Protein
MKWVTFISLLFLFSSAYSMMCIGMSNRDFVEGVSGGSWVDIVLEHGSCVTTMAKNKP
TLDFMLIKTEAKQPATLRKYCIEAKLTNTTTESRCPTQGEPSLNEEQDKRFVCKHSMVD
RGWGNGCGLFGKGGIVTCAMFRCKKNMEGKVVQPENLEYTIVITPHSGEEHAVGNDT
GKHGKEIKITPQSSITEAELTGYGTVTMECSPRTGLDFNEMVLLQMENKAWLVHRQWF
LDLPLPWLPGADTQGSNWIQKETLVTFKNPHAKKQDVVVLGSQEGAMHTALTGATEIQ
MSSGNLLFTGHLKCRLRMDKLQLKGMSYSMCTGKFKVVKEIAETQHGTIVIRVQYEGD
GSPCKIPFEIMDLEKRHVLGRLITVNPIVTEKDSPVNIEAEPPFGDSYIIIGVEPGQLKLN
WFKKGSSGGSHHHHHHHH

pD2sE_EV8_HCat3

DNA
ATGAAGTGGGTAACCTTTATTTCCCTTCTTTTTCTCTTTAGCTCGGCTTATTCCATGA
GGTGTATCGGCATGTCCAACAGGGACTTTGTGGAGGGAGTGAGCGGCGGCAGCT
GGGTGGACATTGTGCTGGAGCCCGGAAGCTGCGTGACCACGATGGCGAAAAACA
AGCCCACCCTGGACTTCGAGCTCATCAAGATCGAGGCTAAACAGCCCGCCACCCT
CAGGAAGTACTGCATCGAGGCCAAGCTGACCAACACAACAACCGAGTCCAGATGC
CCTACACAGGGCGAACCCAGCCTCAACGAAGAGCAGGACAAGAGGTTCGTGTGC
AAACACAGCATGGTGGACAGGGGCTGGGGCAATGGATGCGGACTCTTCGGAAAA
GGCGGCATCGTGACCTGCGCCATGTTCAGGTGTAAAAAGAACATGGAAGGCAAGG
TGGTGCAGCCCGAAAATCTGGAGTATACCATCGTGATTACCCCCCACAGCGGAGA
GGAGCACGCCGTGGGCAATGACACCGGCAAGCACGGCAAAGAGATTAAGATCAC
CCCCCAGTCCTCCATTACCGAAGCTGAACTGACAGGCTACGGCACCGTGACAATG
GAGTGTAGCCCCAGGACCGGACTGGATTTCAACGAGATGGTCCTGCTACAGATGG
AGAACAAGGCCTGGCTCGTGCACAGGCAATGGTTTCTGGATCTGCCTCTGCCTTG
GCTGCCTGGCGCCGATACACAGGGCTCCAACTGGATACAGAAAGAGACCCTCGTC
ACCTTCAAGAATCCCCATGCTAAGAAGCAGGACGTGGTGGTGCTGGGCAGCCAAG
AAGGCGCCATGCACACAGCCCTGACCGGAGCTACCGAGATCCAGATGAGCTCCG
GCAACCTGCTGTTCACCGGCCATCTGAAATGTAGGCTGAGGATGGATAAGCTGCA
ACTCAAAGGCATGTCCTACTCCATGTGCACCGGAAAGTTCAAGGTGGTGAAAGAAA
TCGCCGAAACACAGCACGGCACCATCGTGATCAGGGTGCAGTATGAGGGCGACG
GCTCCCCCTGTAAGATCCCCTTCGAAATCATGGACCTGGAAAAGAGGCACGTGCT
GGGCAGGCTCATCACCGTGAACCCCATTGTCACAGAGAAAGACTCCCCCGTGAAC
ATCGAGGCCGAGCCTCCCTTTGGCGACTCCTACATCATCATTGGCGTGGAGCCCG
GACAGCTCAAGCTGAACTGGTTCAAGAAGGGCAGCAGCGGCGGCAGCCATCACC
ACCATCATCACCATCAT

Protein
MKWVTFISLLFLFSSAYSMRCIGMSNRDFVEGVSGGSWVDIVLEPGSCVTTMAKNKPT
LDFELIKIEAKQPATLRKYCIEAKLTNTTTESRCPTQGEPSLNEEQDKRFVCKHSMVDR
GWGNGCGLFGKGGIVTCAMFRCKKNMEGKVVQPENLEYTIVITPHSGEEHAVGNDTG
KHGKEIKITPQSSITEAELTGYGTVTMECSPRTGLDFNEMVLLQMENKAWLVHRQWFL
DLPLPWLPGADTQGSNWIQKETLVTFKNPHAKKQDVVVLGSQEGAMHTALTGATEIQ
MSSGNLLFTGHLKCRLRMDKLQLKGMSYSMCTGKFKVVKEIAETQHGTIVIRVQYEGD
GSPCKIPFEIMDLEKRHVLGRLITVNPIVTEKDSPVNIEAEPPFGDSYIIIGVEPGQLKLN
WFKKGSSGGSHHHHHHHH

pD2sE_EV8_HCat4

DNA
ATGAAGTGGGTAACCTTTATTTCCCTTCTTTTTCTCTTTAGCTCGGCTTATTCCATGA
TGTGTATCGGCATGTCCAACAGGGACTTTGTGGAGGGAGTGAGCGGCGGCAGCT
GGGTGGACATTGTGCTGGAGCATGGAAGCTGCGTGACCACGATGGCGAAAAACAA
GCCCACCCTGGACTTCCTGCTCATCAAGACAGAGGCTAAACAGCCCGCCACCCTC
AGGAAGTACTGCATCGAGGCCAAGCTGACCAACACAACAACCGAGTCCAGATGCC
CTACACAGGGCGAACCCAGCCTCAACGAAGAGCAGGACAAGAGGTTCGTGTGCAA
ACACAGCATGGTGGACAGGGGCTGGGGCAATGGATGCGGACTCTTCGGAAAAGG
CGGCATCGTGACCTGCGCCATGTTCAGGTGTAAAAAGAACATGGAAGGCAAGGTG
GTGCAGCCCGAAAATCTGGAGTATACCATCGTGATTACCCCCCACAGCGGAGAGG
AGCACGCCGTGGGCAATCTGACCGGCAAGCACGGCAAAGAGATTAAGATCACCCC
CCAGTCCTCCATTACCGAAGCTGAACTGACAGGCTACGGCACCGTGACAATGGAG
TGTAGCCCCAGGACCGGACTGGATTTCAACGAGATGGTCCTGCTACAGATGGAGA
ACAAGGCCTGGCTCGTGCACAGGCAATGGTTTCTGGATCTGCCTCTGCCTTGGCT
GCCTGGCGCCGATACACAGGGCTCCAACTGGATACAGAAAGAGACCCTCGTCACC
TTCAAGAATCCCCATGCTATCAAGCAGGACGTGGTGGTGCTGGGCAGCCAAGAAG
GCGCCATGCACACAGCCCTGACCGGAGCTACCGAGATCCAGATGAGCTCCGGCA
ACCTGCTGTTCACCGGCCATCTGAAATGTAGGCTGAGGATGGATAAGCTGCAACT
CAAAGGCATGTCCTACTCCATGTGCACCGGAAAGTTCAAGGTGGTGAAAGAAATC
GCCGAAACACAGCACGGCACCATCGTGATCAGGGTGCAGTATGAGGGCGACGGC
TCCCCCTGTAAGATCCCCTTCGAAATCATGGACCTGGAAAAGAGGCACGTGCTGG
GCAGGCTCATCACCGTGAACCCCATTGTCACAGAGAAAGACTCCCCCGTGAACAT
CGAGGCCGAGCCTCCCTTTGGCGACTCCTACATCATCATTGGCGTGGAGCCCGGA
CAGCTCAAGCTGAACTGGTTCAAGAAGGGCAGCAGCGGCGGCAGCCATCACCAC
CATCATCACCATCAT

Protein
MKWVTFISLLFLFSSAYSMMCIGMSNRDFVEGVSGGSWVDIVLEHGSCVTTMAKNKP
TLDFLLIKTEAKQPATLRKYCIEAKLTNTTTESRCPTQGEPSLNEEQDKRFVCKHSMVD
RGWGNGCGLFGKGGIVTCAMFRCKKNMEGKVVQPENLEYTIVITPHSGEEHAVGNLT
GKHGKEIKITPQSSITEAELTGYGTVTMECSPRTGLDFNEMVLLQMENKAWLVHRQWF
LDLPLPWLPGADTQGSNWIQKETLVTFKNPHAIKQDVVVLGSQEGAMHTALTGATEIQ
MSSGNLLFTGHLKCRLRMDKLQLKGMSYSMCTGKFKVVKEIAETQHGTIVIRVQYEGD
GSPCKIPFEIMDLEKRHVLGRLITVNPIVTEKDSPVNIEAEPPFGDSYIIIGVEPGQLKLN
WFKKGSSGGSHHHHHHHH

pD2sE_EV8_HCat8

DNA

ATGAAGTGGGTAACCTTTATTTCCCTTCTTTTTCTCTTTAGCTCGGCTTATTCCATGA
GGTGTATCGGCATGTCCAACAGGGACTTTGTGGAGGGAGTGAGCGGCGGCAGCT
GGGTGGACATTGTGCTGGAGCATGGAAGCTGCGTGACCACGATGGCGAAAAACAA
GCCCACCCTGGACTTCGAGCTCATCAAGACAGAGGCTAAACAGCCCGCCACCCTC
AGGAAGTACTGCATCGAGGCCAAGCTGACCAACACAACAACCGAGTCCAGATGCC
CTACACAGGGCGAACCCAGCCTCAACGAAGAGCAGGACAAGAGGTTCGTGTGCAA
ACACAGCATGGTGGACAGGGGCTGGGGCAATGGATGCGGACTCTTCGGAAAAGG
CGGCATCGTGACCTGCGCCATGTTCAGGTGTAAAAAGAACATGGAAGGCAAGGTG
GTGCAGCCCGAAAATCTGGAGTATACCATCGTGATTACCCCCCACAGCGGAGAGG
AGCACGCCGTGGGCAATGACACCGGCAAGCACGGCAAAGAGATTAAGATCACCC
CCCAGTCCTCCATTACCGAAGCTGAACTGACAGGCTACGGCACCGTGACAATGGA
GTGTAGCCCCAGGACCGGACTGGATTTCAACGAGATGGTCCTGCTACAGATGGAG
AACTTCGCCTGGCTCGTGCACAGGCAATGGTTTCTGGATCTGCCTCTGCCTTGGCT
GCCTGGCGCCGATACACAGGGCTCCAACTGGATACAGAAAGAGACCCTCGTCACC
TTCAAGAATCCCCATGCTAAGAAGCAGGACGTGGTGGTGCTGGGCAGCCAAGAAG
GCGCCATGCTGACAGCCCTGACCGGAGCTACCGAGATCCAGATGAGCTCCGGCA
ACCTGCTGTTCACCGGCCATCTGAAATGTAGGCTGAGGATGGATAAGCTGCAACT
CAAAGGCATGTCCTACTCCATGTGCACCGGAAAGTTCAAGGTGGTGAAAGAAATC
GCCGAAACACAGCACGGCACCATCGTGATCAGGGTGCAGTATGAGGGCGACGGC
TCCCCCTGTAAGATCCCCTTCGAAATCATGGACCTGGAAAAGAGGCACGTGCTGG
GCAGGCTCATCACCGTGAACCCCATTGTCACAGAGAAAGACTCCCCCGTGAACAT
CGAGGCCGAGCCTCCCTTTGGCGACTCCTACATCATCATTGGCGTGGAGCCCGGA
CAGCTCAAGCTGAACTGGTTCAAGAAGGGCAGCAGCGGCGGCAGCCATCACCAC
CATCATCACCATCAT

Protein

MKWVTFISLLFLFSSAYSMRCIGMSNRDFVEGVSGGSWVDIVLEHGSCVTTMAKNKPT
LDFELIKTEAKQPATLRKYCIEAKLTNTTTESRCPTQGEPSLNEEQDKRFVCKHSMVDR
GWGNGCGLFGKGGIVTCAMFRCKKNMEGKVVQPENLEYTIVITPHSGEEHAVGNDTG
KHGKEIKITPQSSITEAELTGYGTVTMECSPRTGLDFNEMVLLQMENFAWLVHRQWFL
DLPLPWLPGADTQGSNWIQKETLVTFKNPHAKKQDVVVLGSQEGAMLTALTGATEIQ
MSSGNLLFTGHLKCRLRMDKLQLKGMSYSMCTGKFKVVKEIAETQHGTIVIRVQYEGD
GSPCKIPFEIMDLEKRHVLGRLITVNPIVTEKDSPVNIEAEPPFGDSYIIIGVEPGQLKLN
WFKKGSSGGSHHHHHHHH

pD2sE_EV8_IntFc1

DNA
ATGAAGTGGGTAACCTTTATTTCCCTTCTTTTTCTCTTTAGCTCGGCTTATTCCATGA
GGTGTATCGGCATGTCCAACAGGGACTTTGTGGAGGGAGTGAGCGGCGGCAGCT
GGGTGGACATTGTGCTGGAGCATGGAAGCTGCGTGACCACGATGGCGAAAAACAA
GCCCACCCTGGACTTCGAGCTCATCAAGACAGAGGCTAAACAGCCCGCCACCCTC
AGGAAGTACTGCATCGAGGCCAAGCTGACCAACACAACAACCGAGTCCAGATGCC
CTACACAGGGCGAACCCAGCCTCAACGAAGAGCAGGACAAGAGGTTCGTGTGCAA
ACACAGCATGGTGGACAGGGGCTGGGGCAATGGATGCGGACTCTTCGGAAAAGG
CGGCATCGTGACCTGCGCCATGTTCAGGTGTAAAAAGAACATGGAAGGCAAGGTG
GTGCAGCCCGAAAATCTGGAGTATACCATCGTGATTACCCCCCACAGCGGAGAGG
AGCACGCCGTGGGCAATGACACCGGCAAGCACGGCAAAGAGATTAAGATCACCC
CCCAGTCCTCCATTACCGAAGCTGAACTGACAGGCTACGGCACCGTGACAATGGA
GTGTAGCCCCAGGACCGGACTGGATTTCAACGAGATGGTCCTGCTACAGATGGAG
AACAAGGCCTGGCTCGTGCACAGGCAATGGTTTCTGGATCTGCCTCTGCCTTGGC
TGCCTGGCGCCGATACACAGGGCTCCAACTGGATACAGAAAGAGACCCTCGTCAC
CTTCAAGAATCCCCATGCTAAGAAGCAGGACGTGGTGGTGCTGGGCAGCCAAGAA
GGCGTGATGCACCACGCCCTGACCGGAGCTACCGAGATCCAGATGAGCTCCGGC
AACCTGCTGTTCACCGGCCATCTGAAATGTAGGCTGAGGATGGATAAGCTGCAAC
TCAAAGGCATGTCCTACTCCATGTGCACCGGAAAGTTCAAGGTGGTGAAAGAAATC
GCCGAAACACAGCACGGCACCATCGTGATCAGGGTGCAGTATGAGGGCGACGGC
TCCCCCTGTAAGATCCCCTTCGAAATCATGGACCTGGAAAAGAGGCACGTGCTGG
GCAGGCTCATCACCGTGAACCCCATTGTCACAGAGAAAGACTCCCCCGTGAACAT
CGAGGCCGAGCCTCCCTTTGGCGACTCCTACATCATCATTGGCGTGGAGCCCGGA
CAGCTCAAGCTGAACTGGTTCAAGAAGGGCAGCAGCGGCGGCAGCCATCACCAC
CATCATCACCATCAT

Protein
MKWVTFISLLFLFSSAYSMRCIGMSNRDFVEGVSGGSWVDIVLEHGSCVTTMAKNKPT
LDFELIKTEAKQPATLRKYCIEAKLTNTTTESRCPTQGEPSLNEEQDKRFVCKHSMVDR
GWGNGCGLFGKGGIVTCAMFRCKKNMEGKVVQPENLEYTIVITPHSGEEHAVGNDTG
KHGKEIKITPQSSITEAELTGYGTVTMECSPRTGLDFNEMVLLQMENKAWLVHRQWFL
DLPLPWLPGADTQGSNWIQKETLVTFKNPHAKKQDVVVLGSQEGVMHHALTGATEIQ
MSSGNLLFTGHLKCRLRMDKLQLKGMSYSMCTGKFKVVKEIAETQHGTIVIRVQYEGD
GSPCKIPFEIMDLEKRHVLGRLITVNPIVTEKDSPVNIEAEPPFGDSYIIIGVEPGQLKLN
WFKKGSSGGSHHHHHHHH

pD2sE_EV8_IntFc2

DNA
ATGAAGTGGGTAACCTTTATTTCCCTTCTTTTTCTCTTTAGCTCGGCTTATTCCATGA
GGTGTATCGGCATGTCCAACAGGGACTTTGTGGAGGGAGTGAGCGGCGGCAGCT
GGGTGGACATTGTGCTGGAGCATGGAAGCTGCGTGACCACGATGGCGAAAAACAA
GCCCACCCTGGACTTCGAGCTCATCAAGACAGAGGCTAAACAGCCCGCCACCCTC
AGGAAGTACTGCATCGAGGCCAAGCTGACCAACACAACAACCGAGTCCAGATGCC
CTACACAGGGCGAACCCAGCCTCAACGAAGAGCAGGACAAGAGGTTCGTGTGCAA
ACACAGCATGGTGGACAGGGGCTGGGGCAATGGATGCGGACTCTTCGGAAAAGG
CGGCATCGTGACCTGCGCCATGTTCAGGTGTAAAAAGAACATGGAAGGCAAGGTG
GTGCAGCCCGAAAATCTGGAGTATACCATCGTGATTACCCCCCACAGCGGAGAGG
AGCACGCCGTGGGCAATGACACCGGCAAGCACGGCAAAGAGATTAAGATCACCC
CCCAGTCCTCCATTACCGAAGCTGAACTGACAGGCTACGGCACCGTGACAATGGA
GTGTAGCCCCAGGACCGGACTGGATTTCAACGAGATGGTCCTGCTACAGATGGAG
AACAAGGCCTGGCTCGTGCACAGGCAATGGTTTCTGGATCTGCCTCTGCCTTGGC
TGCCTGGCGCCGATACACAGGGCTCCAACTGGATACAGAAAGAGACCCTCGTCAC
CTTCAAGAATCCCCATGCTAAGAAGCAGGACGTGGTGGTGCTGGGCAGCCAAGAA
GGCTGGATGCACCGGGCCCTGACCGGAGCTACCGAGATCCAGATGAGCTCCGGC
AACCTGCTGTTCACCGGCCATCTGAAATGTAGGCTGAGGATGGATAAGCTGCAAC
TCAAAGGCATGTCCTACTCCATGTGCACCGGAAAGTTCAAGGTGGTGAAAGAAATC
GCCGAAACACAGCACGGCACCATCGTGATCAGGGTGCAGTATGAGGGCGACGGC
TCCCCCTGTAAGATCCCCTTCGAAATCATGGACCTGGAAAAGAGGCACGTGCTGG
GCAGGCTCATCACCGTGAACCCCATTGTCACAGAGAAAGACTCCCCCGTGAACAT
CGAGGCCGAGCCTCCCTTTGGCGACTCCTACATCATCATTGGCGTGGAGCCCGGA
CAGCTCAAGCTGAACTGGTTCAAGAAGGGCAGCAGCGGCGGCAGCCATCACCAC
CATCATCACCATCAT

Protein
MKWVTFISLLFLFSSAYSMRCIGMSNRDFVEGVSGGSWVDIVLEHGSCVTTMAKNKPT
LDFELIKTEAKQPATLRKYCIEAKLTNTTTESRCPTQGEPSLNEEQDKRFVCKHSMVDR
GWGNGCGLFGKGGIVTCAMFRCKKNMEGKVVQPENLEYTIVITPHSGEEHAVGNDTG
KHGKEIKITPQSSITEAELTGYGTVTMECSPRTGLDFNEMVLLQMENKAWLVHRQWFL
DLPLPWLPGADTQGSNWIQKETLVTFKNPHAKKQDVVVLGSQEGWMHRALTGATEIQ
MSSGNLLFTGHLKCRLRMDKLQLKGMSYSMCTGKFKVVKEIAETQHGTIVIRVQYEGD
GSPCKIPFEIMDLEKRHVLGRLITVNPIVTEKDSPVNIEAEPPFGDSYIIIGVEPGQLKLN
WFKKGSSGGSHHHHHHHH

pD2sE_EV8_IntFc3

DNA
ATGAAGTGGGTAACCTTTATTTCCCTTCTTTTTCTCTTTAGCTCGGCTTATTCCATGA
GGTGTATCGGCATGTCCAACAGGGACTTTGTGGAGGGAGTGAGCGGCGGCAGCT
GGGTGGACATTGTGCTGGAGCATGGAAGCTGCGTGACCACGATGGCGAAAAACAA
GCCCACCCTGGACTTCGAGCTCATCAAGACAGAGGCTAAACAGCCCGCCACCCTC
AGGAAGTACTGCATCGAGGCCAAGCTGACCAACACAACAACCGAGTCCAGATGCC
CTACACAGGGCGAACCCAGCCTCAACGAAGAGCAGGACAAGAGGTTCGTGTGCAA
ACACAGCATGGTGGACAGGGGCTGGGGCAATGGATGCGGACTCTTCGGAAAAGG
CGGCATCGTGACCTGCGCCATGTTCAGGTGTAAAAAGAACATGGAAGGCAAGGTG
GTGCAGCCCGAAAATCTGGAGTATACCATCGTGATTACCCCCCACAGCGGAGAGG
AGCACGCCGTGGGCAATGACACCGGCAAGCACGGCAAAGAGATTAAGATCACCC
CCCAGTCCTCCATTACCGAAGCTGAACTGACAGGCTACGGCACCGTGACAATGGA
GTGTAGCCCCAGGACCGGACTGGATTTCAACGAGATGGTCCTGCTACAGATGGAG
AACAAGGCCTGGCTCGTGCACAGGCAATGGTTTCTGGATCTGCCTCTGCCTTGGC
TGCCTGGCGCCGATACACAGGGCTCCAACTGGATACAGAAAGAGACCCTCGTCAC
CTTCAAGAATCCCCATGCTAAGAAGCAGGACGTGGTGGTGCTGGGCAGCCAAGAA
GGCGTGATGCACCGGTGGCTGACCGGAGCTACCGAGATCCAGATGAGCTCCGGC
AACCTGCTGTTCACCGGCCATCTGAAATGTAGGCTGAGGATGGATAAGCTGCAAC
TCAAAGGCATGTCCTACTCCATGTGCACCGGAAAGTTCAAGGTGGTGAAAGAAATC
GCCGAAACACAGCACGGCACCATCGTGATCAGGGTGCAGTATGAGGGCGACGGC
TCCCCCTGTAAGATCCCCTTCGAAATCATGGACCTGGAAAAGAGGCACGTGCTGG
GCAGGCTCATCACCGTGAACCCCATTGTCACAGAGAAAGACTCCCCCGTGAACAT
CGAGGCCGAGCCTCCCTTTGGCGACTCCTACATCATCATTGGCGTGGAGCCCGGA
CAGCTCAAGCTGAACTGGTTCAAGAAGGGCAGCAGCGGCGGCAGCCATCACCAC
CATCATCACCATCAT

Protein
MKWVTFISLLFLFSSAYSMRCIGMSNRDFVEGVSGGSWVDIVLEHGSCVTTMAKNKPT
LDFELIKTEAKQPATLRKYCIEAKLTNTTTESRCPTQGEPSLNEEQDKRFVCKHSMVDR
GWGNGCGLFGKGGIVTCAMFRCKKNMEGKVVQPENLEYTIVITPHSGEEHAVGNDTG
KHGKEIKITPQSSITEAELTGYGTVTMECSPRTGLDFNEMVLLQMENKAWLVHRQWFL
DLPLPWLPGADTQGSNWIQKETLVTFKNPHAKKQDVVVLGSQEGVMHRWLTGATEIQ
MSSGNLLFTGHLKCRLRMDKLQLKGMSYSMCTGKFKVVKEIAETQHGTIVIRVQYEGD
GSPCKIPFEIMDLEKRHVLGRLITVNPIVTEKDSPVNIEAEPPFGDSYIIIGVEPGQLKLN
WFKKGSSGGSHHHHHHHH

pD2sE_EV8_IntFc5

DNA

ATGAAGTGGGTAACCTTTATTTCCCTTCTTTTTCTCTTTAGCTCGGCTTATTCCATGA
GGTGTATCGGCATGTCCAACAGGGACTTTGTGGAGGGAGTGAGCGGCGGCAGCT
GGGTGGACATTGTGCTGGAGCATGGAAGCTGCGTGACCACGATGGCGAAAAACAA
GCCCACCCTGGACTTCGAGCTCATCAAGACAGAGGCTAAACAGCCCGCCACCCTC
AGGAAGTACTGCATCGAGGCCAAGCTGACCAACACAACAACCGAGTCCAGATGCC
CTACACAGGGCGAACCCAGCCTCAACGAAGAGCAGGACAAGAGGTTCGTGTGCAA
ACACAGCATGGTGGACAGGGGCTGGGGCAATGGATGCGGACTCTTCGGAAAAGG
CGGCATCGTGACCTGCGCCATGTTCAGGTGTAAAAAGAACATGGAAGGCAAGGTG
GTGCAGCCCGAAAATCTGGAGTATACCATCGTGATTACCCCCCACAGCGGAGAGG
AGCACGCCGTGGGCAATGACACCGGCAAGCACGGCAAAGAGATTAAGATCACCC
CCCAGTCCTCCATTACCGAAGCTGAACTGACAGGCTACGGCACCGTGACAATGGA
GTGTAGCCCCAGGACCGGACTGGATTTCAACGAGATGGTCCTGCTACAGATGGAG
AACAAGGCCTGGCTCGTGCACAGGCAATGGTTTCTGGATCTGCCTCTGCCTTGGC
TGCCTGGCGCCGATACACAGGGCTCCAACTGGATACAGAAAGAGACCCTCGTCAC
CTTCAAGAATCCCCATGCTAAGAAGCAGGACGTGGTGGTGCTGGGCAGCCAAGAA
GGCGCCATGCACCGGTGGCTGACCGGAGCTACCGAGATCCAGATGAGCTCCGGC
AACCTGCTGTTCACCGGCCATCTGAAATGTAGGCTGAGGATGGATAAGCTGCAAC
TCAAAGGCATGTCCTACTCCATGTGCACCGGAAAGTTCAAGGTGGTGAAAGAAATC
GCCGAAACACAGCACGGCACCATCGTGATCAGGGTGCAGTATGAGGGCGACGGC
TCCCCCTGTAAGATCCCCTTCGAAATCATGGACCTGGAAAAGAGGCACGTGCTGG
GCAGGCTCATCACCGTGAACCCCATTGTCACAGAGAAAGACTCCCCCGTGAACAT
CGAGGCCGAGCCTCCCTTTGGCGACTCCTACATCATCATTGGCGTGGAGCCCGGA
CAGCTCAAGCTGAACTGGTTCAAGAAGGGCAGCAGCGGCGGCAGCCATCACCAC
CATCATCACCATCAT

Protein

MKWVTFISLLFLFSSAYSMRCIGMSNRDFVEGVSGGSWVDIVLEHGSCVTTMAKNKPT
LDFELIKTEAKQPATLRKYCIEAKLTNTTTESRCPTQGEPSLNEEQDKRFVCKHSMVDR
GWGNGCGLFGKGGIVTCAMFRCKKNMEGKVVQPENLEYTIVITPHSGEEHAVGNDTG
KHGKEIKITPQSSITEAELTGYGTVTMECSPRTGLDFNEMVLLQMENKAWLVHRQWFL
DLPLPWLPGADTQGSNWIQKETLVTFKNPHAKKQDVVVLGSQEGAMHRWLTGATEIQ
MSSGNLLFTGHLKCRLRMDKLQLKGMSYSMCTGKFKVVKEIAETQHGTIVIRVQYEGD
GSPCKIPFEIMDLEKRHVLGRLITVNPIVTEKDSPVNIEAEPPFGDSYIIIGVEPGQLKLN
WFKKGSSGGSHHHHHHHH

pD2sE_EV8_IntFc6

DNA
ATGAAGTGGGTAACCTTTATTTCCCTTCTTTTTCTCTTTAGCTCGGCTTATTCCATGA
GGTGTATCGGCATGTCCCTGAGGGACTTTGTGGAGGGAGTGAGCGGCGGCAGCT
GGGTGGACATTGTGCTGGAGCATGGATACTGCGTGACCACGATGGCGAAAAACAA
GCCCACCCTGGACTTCGAGCTCATCAAGACAGAGGCTAAACAGCCCGCCACCCTC
AGGAAGTACTGCATCGAGGCCAAGCTGACCAACACAACAACCGAGTCCAGATGCC
CTACACAGGGCGAACCCAGCCTCAACGAAGAGCAGGACAAGAGGTTCGTGTGCAA
ACACAGCATGGTGGACAGGGGCTGGGGCAATGGATGCGGACTCTTCGGAAAAGG
CGGCATCGTGACCTGCGCCATGTTCAGGTGTAAAAAGAACATGGAAGGCAAGGTG
GTGCAGCCCGAAAATCTGGAGTATACCATCGTGATTACCCCCCACAGCGGAGAGG
AGCACGCCGTGGGCAATGACACCGGCAAGCACGGCAAAGAGATTAAGATCACCC
CCCAGTCCTCCATTACCGAAGCTGAACTGACAGGCTACGGCACCGTGACAATGGA
GTGTAGCCCCAGGACCGGACTGGATTTCAACGAGATGGTCCTGCTACAGATGGAG
AACAAGGCCTGGCTCGTGCACAGGCAATGGTTTCTGGATCTGCCTCTGCCTTGGC
TGCCTGGCGCCGATACACAGGGCTCCAACTGGATACAGAAAGAGACCCTCGTCAC
CTTCAAGAATCCCTTCGCTAAGAAGCAGGACGTGGTGGTGCTGGGCAGCCAAGAA
GGCGCCATGCACACAGCCCTGACCGGAGCTACCGAGATCCAGATGAGCTCCGGC
AACCTGCTGTTCACCGGCCATCTGAAATGTAGGCTGAGGATGGATAAGCTGCAAC
TCAAAGGCATGTCCTACTCCATGTGCACCGGAAAGTTCAAGGTGGTGAAAGAAATC
GCCGAAACACAGCACGGCACCATCGTGATCAGGGTGCAGTATGAGGGCGACGGC
TCCCCCTGTAAGATCCCCTTCGAAATCATGGACCTGGAAAAGAGGCACGTGCTGG
GCAGGCTCATCACCGTGAACCCCATTGTCACAGAGAAAGACTCCCCCGTGAACAT
CGAGGCCGAGCCTCCCTTTGGCGACTCCTACATCATCATTGGCGTGGAGCCCGGA
CAGCTCAAGCTGAACTGGTTCAAGAAGGGCAGCAGCGGCGGCAGCCATCACCAC
CATCATCACCATCAT

Protein
MKWVTFISLLFLFSSAYSMRCIGMSLRDFVEGVSGGSWVDIVLEHGYCVTTMAKNKPT
LDFELIKTEAKQPATLRKYCIEAKLTNTTTESRCPTQGEPSLNEEQDKRFVCKHSMVDR
GWGNGCGLFGKGGIVTCAMFRCKKNMEGKVVQPENLEYTIVITPHSGEEHAVGNDTG
KHGKEIKITPQSSITEAELTGYGTVTMECSPRTGLDFNEMVLLQMENKAWLVHRQWFL
DLPLPWLPGADTQGSNWIQKETLVTFKNPFAKKQDVVVLGSQEGAMHTALTGATEIQ
MSSGNLLFTGHLKCRLRMDKLQLKGMSYSMCTGKFKVVKEIAETQHGTIVIRVQYEGD
GSPCKIPFEIMDLEKRHVLGRLITVNPIVTEKDSPVNIEAEPPFGDSYIIIGVEPGQLKLN
WFKKGSSGGSHHHHHHHH

pD2sE_EV8_IntFc7

DNA
ATGAAGTGGGTAACCTTTATTTCCCTTCTTTTTCTCTTTAGCTCGGCTTATTCCATGA
GGTGTATCGGCATGTCCAACAGGGACTTTGTGGAGGGAGTGAGCGGCGGCAGCT
GGGTGGACATTGTGCTGGAGCATGGAAGCTGCGTGACCACGATGGCGAAAAACAA
GCCCACCCTGGACTTCGAGCTCATCAAGACAGAGGCTAAACAGCCCGCCACCCTC
AGGAAGTACTGCATCGAGGCCAAGCTGACCAACACAACAACCGAGTCCAGATGCC
CTACACAGGGCGAACCCAGCCTCAACGAAGAGCAGGACAAGAGGTTCGTGTGCAA
ACACAGCATGGTGGACAGGGGCTGGGGCAATGGATGCGGACTCTTCGGAAAAGG
CGGCATCGTGACCTGCGCCATGTTCAGGTGTAAAAAGAACATGGAAGGCAAGGTG
GTGCAGCCCGAAAATCTGGAGTATACCATCGTGATTACCCCCCACAGCGGAGAGG
AGCACGCCGTGGGCAATGACACCGGCAAGCACGGCAAAGAGATTAAGATCACCC
CCCAGTCCTCCATTACCGAAGCTGAACTGACAGGCTACGGCACCGTGACAATGGA
GTGTAGCCCCAGGACCGGACTGGATTTCAACGAGATGGTCCTGCTACAGATGGAG
AACAAGGCCTGGCTCGTGCACAGGCAATGGTTTCTGGATCTGCCTCTGCCTTGGC
TGCCTGGCGCCGATACACAGGGCTCCAACTGGATACAGAAAGAGACCCTCGTCAC
CTTCAAGAATCCCCATGCTAAGAAGCAGGACGTGGTGGTGCTGGGCAGCCAAGAA
GGCGCCATGCACCGGGCCCTGACCGGAGCTACCGAGATCCAGATGAGCTCCGGC
AACCTGCTGTTCACCGGCCATCTGAAATGTAGGCTGAGGATGGATAAGCTGCAAC
TCAAAGGCATGTCCTACTCCATGTGCACCGGAAAGTTCAAGGTGGTGAAAGAAATC
GCCGAAACACAGCACGGCACCATCGTGATCAGGGTGCAGTATGAGGGCGACGGC
TCCCCCTGTAAGATCCCCTTCGAAATCATGGACCTGGAAAAGAGGCACGTGCTGG
GCAGGCTCATCACCGTGAACCCCATTGTCACAGAGAAAGACTCCCCCGTGAACAT
CGAGGCCGAGCCTCCCTTTGGCGACTCCTACATCATCATTGGCGTGGAGCCCGGA
CAGCTCAAGCTGAACTGGTTCAAGAAGGGCAGCAGCGGCGGCAGCCATCACCAC
CATCATCACCATCAT

Protein
MKWVTFISLLFLFSSAYSMRCIGMSNRDFVEGVSGGSWVDIVLEHGSCVTTMAKNKPT
LDFELIKTEAKQPATLRKYCIEAKLTNTTTESRCPTQGEPSLNEEQDKRFVCKHSMVDR
GWGNGCGLFGKGGIVTCAMFRCKKNMEGKVVQPENLEYTIVITPHSGEEHAVGNDTG
KHGKEIKITPQSSITEAELTGYGTVTMECSPRTGLDFNEMVLLQMENKAWLVHRQWFL
DLPLPWLPGADTQGSNWIQKETLVTFKNPHAKKQDVVVLGSQEGAMHRALTGATEIQ
MSSGNLLFTGHLKCRLRMDKLQLKGMSYSMCTGKFKVVKEIAETQHGTIVIRVQYEGD
GSPCKIPFEIMDLEKRHVLGRLITVNPIVTEKDSPVNIEAEPPFGDSYIIIGVEPGQLKLN
WFKKGSSGGSHHHHHHHH

pD2sE_EV8_IntFc8

DNA
ATGAAGTGGGTAACCTTTATTTCCCTTCTTTTTCTCTTTAGCTCGGCTTATTCCATGA
GGTGTATCGGCATGTCCAACAGGGACTTTGTGGAGGGAGTGAGCGGCGGCAGCT
GGGTGGACATTGTGCTGGAGCATGGAAGCTGCGTGACCACGATGGCGAAAAACAA
GCCCACCCTGGACTTCGAGCTCATCAAGACAGAGGCTAAACAGCCCGCCACCCTC
AGGAAGTACTGCATCGAGGCCAAGCTGACCAACACAACAACCGAGTCCAGATGCC
CTACACAGGGCGAACCCAGCCTCAACGAAGAGCAGGACAAGAGGTTCGTGTGCAA
ACACAGCATGGTGGACAGGGGCTGGGGCAATGGATGCGACCTCTTCGGAAAAGG
CGGCATCGTGACCTGCGCCATGTTCAGGTGTAAAAAGAACATGGAAGGCAAGGTG
GTGCAGCCCGAAAATCTGGAGTATACCATCGTGATTACCCCCCACAGCGGAGAGG
AGCACGCCGTGGGCAATGACACCGGCAAGCACGGCAAAGAGATTAAGATCACCC
CCCAGTCCTCCATTACCGAAGCTGAACTGACAGGCTACGGCACCGTGACAATGGA
GTGTAGCCCCAGGACCGGACTGGATTTCAACGAGATGGTCCTGCTACAGATGGAG
AACAAGGCCTGGCTCGTGCACAGGCAATGGTTTCTGGATCTGCCTCTGCCTTGGC
TGCCTGGCGCCGATACACAGGGCTCCAACTGGATACAGAAAGAGACCCTCGTCAC
CTTCAAGAATCCCCATGCTAAGAAGCAGGACGTGGTGGTGCTGGGCAGCCAAGAA
GGCGCCATGCACACAGCCCTGACCGGAGCTACCGAGATCCAGATGAGCTCCGGC
AACCTGCTGTTCACCGGCCATCTGAAATGTAGGCTGAGGATGGATAAGCTGCAAC
TCAAAGGCATGTCCTACTCCATGTGCACCGGAAAGTTCAAGGTGGTGAAAGAAATC
GCCGAAACACAGCACGGCACCATCGTGATCAGGGTGCAGTATGAGGGCGACGGC
TCCCCCTGTAAGATCCCCTTCGAAATCATGGACCTGGAAAAGAGGCACGTGCTGG
GCAGGCTCATCACCGTGAACCCCATTGTCACAGAGAAAGACTCCCCCGTGAACAT
CGAGGCCGAGCCTCCCTTTGGCGACTCCTACATCATCATTGGCGTGGAGCCCGGA
CAGCTCAAGCTGAACTGGTTCAAGAAGGGCAGCAGCGGCGGCAGCCATCACCAC
CATCATCACCATCAT

Protein
MKWVTFISLLFLFSSAYSMRCIGMSNRDFVEGVSGGSWVDIVLEHGSCVTTMAKNKPT
LDFELIKTEAKQPATLRKYCIEAKLTNTTTESRCPTQGEPSLNEEQDKRFVCKHSMVDR
GWGNGCDLFGKGGIVTCAMFRCKKNMEGKVVQPENLEYTIVITPHSGEEHAVGNDTG
KHGKEIKITPQSSITEAELTGYGTVTMECSPRTGLDFNEMVLLQMENKAWLVHRQWFL
DLPLPWLPGADTQGSNWIQKETLVTFKNPHAKKQDVVVLGSQEGAMHTALTGATEIQ
MSSGNLLFTGHLKCRLRMDKLQLKGMSYSMCTGKFKVVKEIAETQHGTIVIRVQYEGD
GSPCKIPFEIMDLEKRHVLGRLITVNPIVTEKDSPVNIEAEPPFGDSYIIIGVEPGQLKLN
WFKKGSSGGSHHHHHHHH

pD2sE_EV8_Mnmer2

DNA
ATGAAGTGGGTAACCTTTATTTCCCTTCTTTTTCTCTTTAGCTCGGCTTATTCCATGA
GGTGTATCGGCATGTCCAACAGGGACTTTGTGGAGGGAGTGAGCGGCGGCAGCT
GGGTGGACATTGTGCTGGAGCATGGAAGCTGCGTGACCACGATGGCGAAAAACAA
GCCCACCCTGGACTTCGAGCTCATCAAGACAGAGGCTAAACAGCCCGCCACCCTC
AGGAAGTACTGCATCGAGGCCAAGCTGACCAACACAACAACCGAGTCCAGATGCC
CTACACAGGGCGAACCCAGCCTCAACGAAGAGCAGGACAAGAGGTTCGTGTGCAA
ACACAGCATGGTGGACAGGGGCTGGGGCAATGGATGCGGACTCTTCGGAAAAGG
CGGCATCGTGACCTGCGCCATGTTCAGGTGTAAAAAGAACATGGAAGGCAAGGTG
GTGCAGCCCGAAAATCTGGAGTATACCATCGTGATTACCCCCCACAGCGGAGAGG
AGCACGCCGTGGGCAATGACACCGGCAAGCACGGCAAAGAGATTAAGATCACCC
CCCAGTCCTCCATTACCGAAGCTGAACTGACAGGCTACGGCACCGTGACAATGGA
GTGTAGCCCCAGGACCGGACTGGATTTCAACGAGATGGTCCTGCTACAGATGGAG
AACAAGGCCTGGCTCGTGCACAGGCAATGGTTTCTGGATCTGCCTCTGCCTTGGC
TGCCTGGCGCCGATACACAGGGCTCCAACTGGATACAGAAAGAGACCCTCGTCAC
CTTCAAGAATCCCCATGCTAAGAAGCAGGACGTGGTGGTGCTGGGCAGCCAAGAA
GAGGCCATGCACACAGCCCTGACCGGAGCTACCGAGATCCAGATGAGCTCCGGC
AACCTGCTGTTCACCGGCCATCTGAAATGTAGGCTGAGGATGGATAAGCTGCAAC
TCAAAGGCATGTCCTACTCCATGTGCACCGGAAAGTTCAAGGTGGTGAAAGAAATC
GCCGAAACACAGCACGGCACCATCGTGATCAGGGTGCAGTATGAGGGCGACGGC
TCCCCCTGTAAGATCCCCTTCGAAATCATGGACCTGGAAAAGAGGCACGTGCTGG
GCAGGCTCATCACCGTGAACCCCATTGTCACAGAGAAAGACTCCCCCGTGAACAT
CGAGGCCGAGCCTCCCTTTGGCGACTCCTACATCATCATTGGCGTGGAGCCCGGA
CAGCTCAAGCTGAACTGGTTCAAGAAGGGCAGCAGCGGCGGCAGCCATCACCAC
CATCATCACCATCAT

Protein
MKWVTFISLLFLFSSAYSMRCIGMSNRDFVEGVSGGSWVDIVLEHGSCVTTMAKNKPT
LDFELIKTEAKQPATLRKYCIEAKLTNTTTESRCPTQGEPSLNEEQDKRFVCKHSMVDR
GWGNGCGLFGKGGIVTCAMFRCKKNMEGKVVQPENLEYTIVITPHSGEEHAVGNDTG
KHGKEIKITPQSSITEAELTGYGTVTMECSPRTGLDFNEMVLLQMENKAWLVHRQWFL
DLPLPWLPGADTQGSNWIQKETLVTFKNPHAKKQDVVVLGSQEEAMHTALTGATEIQ
MSSGNLLFTGHLKCRLRMDKLQLKGMSYSMCTGKFKVVKEIAETQHGTIVIRVQYEGD
GSPCKIPFEIMDLEKRHVLGRLITVNPIVTEKDSPVNIEAEPPFGDSYIIIGVEPGQLKLN
WFKKGSSGGSHHHHHHHH

pD2sE_EV8_PM1

DNA
ATGAAGTGGGTAACCTTTATTTCCCTTCTTTTTCTCTTTAGCTCGGCTTATTCCATGA
GGTGTATCGGCCTGTCCAACAGGGACTTTGTGGAGGGAGTGAGCGGCGGCAGCT
GGGTGGACATTGTGCTGGAGCATGGAAGCTGCGTGACCACGATGGCGAAAAACAA
GCCCACCCTGGACTTCGAGCTCATCAAGACAGAGGCTAAACAGCCCGCCACCCTC
AGGAAGTACTGCATCGAGGCCAAGCTGACCAACACAACAACCGAGTCCAGATGCC
CTACACAGGGCGAACCCAGCCTCAACGAAGAGCAGGACAAGAGGTTCGTGTGCAA
ACACAGCATGGTGGACAGGGGCTGGGGCAATGGATGCGGACTCTTCGGAAAAGG
CGGCATCGTGACCTGCGCCATGTTCAGGTGTAAAAAGAACATGGAAGGCAAGGTG
GTGCAGCCCGAAAATCTGGAGTATACCATCGTGATTACCCCCCACAGCGGAGAGG
AGCACGCCGTGGGCAATGACACCGGCAAGCACGGCAAAGAGATTAAGATCACCC
CCCAGTCCTCCATTACCGAAGCTGAACTGACAGGCTACGGCACCGTGACAATGGA
GTGTAGCCCCAGGACCGGACTGGATTTCAACGAGATGGTCCTGCTACAGATGGAG
AACAAGGCCTGGCTCGTGCACAGGCAATGGTTTCTGGATCTGCCTCTGCCTTGGC
TGCCTGGCGCCGATACACAGGGCTCCAACTGGATACAGAAAGAGACCCTCGTCAC
CTTCAAGAATCCCCATGCTAAGAAGCAGGACGTGGTGGTGCTGGGCAGCCAAGAA
GGCGCCATGCACACAGCCCTGACCGGAGCTACCGAGATCCAGATGAGCTCCGGC
AACCTGCTGTTCACCGGCCATCTGAAATGTAGGCTGAGGATGGATAAGCTGCAAC
TCAAAGGCATGTCCTACTCCATGTGCACCGGAAAGTTCAAGGTGGTGAAAGAAATC
GCCGAAACAATGCACGGCACCATCGTGATCAGGGTGCAGTATGAGGGCGACGGC
TCCCCCTGTAAGATCCCCTTCGAAATCATGGACCTGGAAAAGAGGCACGTGCTGG
GCAGGCTCATCACCGTGAACCCCATTGTCACAGAGAAAGACTCCCCCGTGAACAT
CGAGGCCGAGCCTCCCTTTGGCGACTCCTACATCATCATTGGCGTGGAGCCCGGA
CAGCTCAAGCTGAACTGGTTCAAGAAGGGCAGCAGCGGCGGCAGCCATCACCAC
CATCATCACCATCAT

Protein
MKWVTFISLLFLFSSAYSMRCIGLSNRDFVEGVSGGSWVDIVLEHGSCVTTMAKNKPT
LDFELIKTEAKQPATLRKYCIEAKLTNTTTESRCPTQGEPSLNEEQDKRFVCKHSMVDR
GWGNGCGLFGKGGIVTCAMFRCKKNMEGKVVQPENLEYTIVITPHSGEEHAVGNDTG
KHGKEIKITPQSSITEAELTGYGTVTMECSPRTGLDFNEMVLLQMENKAWLVHRQWFL
DLPLPWLPGADTQGSNWIQKETLVTFKNPHAKKQDVVVLGSQEGAMHTALTGATEIQ
MSSGNLLFTGHLKCRLRMDKLQLKGMSYSMCTGKFKVVKEIAETMHGTIVIRVQYEGD
GSPCKIPFEIMDLEKRHVLGRLITVNPIVTEKDSPVNIEAEPPFGDSYIIIGVEPGQLKLN
WFKKGSSGGSHHHHHHHH

pD2sE_EV8_PM2

DNA
ATGAAGTGGGTAACCTTTATTTCCCTTCTTTTTCTCTTTAGCTCGGCTTATTCCATGA
GGTGTATCGGCATGTCCAACAGGGACTTTGTGGAGGGAGTGAGCGGCGGCAGCT
GGGTGGACATTGTGCTGGAGCATGGAAGCTGCGTGACCACGATGGCGAAAAACAA
GCCCACCCTGGACTTCGAGCTCATCAAGACAGAGGCTAAACAGCCCGCCACCCTC
AGGAAGTACTGCATCGAGGCCAAGCTGACCAACACAACAACCGAGTCCAGATGCC
CTACACAGGGCGAACCCAGCCTCAACGAAGAGCAGGACAAGAGGTTCGTGTGCAA
ACACAGCATGGTGGACAGGGGCTGGGGCAATGGATGCGGACTCTTCGGAAAAGG
CGGCATCGTGACCTGCGCCATGTTCAGGTGTAAAAAGAACATGGAAGGCAAGGTG
GTGATCCCCGAAAATCTGGAGTATACCATCGTGATTACCCCCCACAGCGGAGAGG
AGCACGCCGTGGGCAATGACACCGGCAAGCACGGCAAAGAGATTAAGATCACCC
CCCAGTCCTCCATTACCGAAGCTGAACTGACAGGCTACGGCACCGTGACAATGGA
GTGTAGCCCCAGGACCGGACTGGATTTCAACGAGATGGTCCTGCTACAGATGGAG
AACAAGGCCTGGCTCGTGCACAGGCAATGGTTTCTGGATCTGCCTCTGCCTTGGC
TGCCTGGCGCCGATACACAGGGCTCCAACTGGATACAGAAAGAGACCCTCGTCAC
CTTCAAGAATCCCCATGCTAAGAAGCAGGACGTGGTGGTGCTGGGCAGCCAAGAA
GGCGCCATGCACACAGCCCTGACCGGAGCTACCGAGATCCAGATGAGCTCCGGC
AACCTGCTGTTCACCGGCCATCTGAAATGTAGGCTGAGGATGGATAAGCTGCAAC
TCAAAGGCATGTCCCTGTCCATGTGCACCGGAAAGTTCAAGGTGGTGAAAGAAAT
CGCCGAAACACAGCACGGCACCATCGTGATCAGGGTGCAGTATGAGGGCGACGG
CTCCCCCTGTAAGATCCCCTTCGAAATCATGGACCTGGAAAAGAGGCACGTGCTG
GGCAGGCTCATCACCGTGAACCCCATTGTCACAGAGAAAGACTCCCCCGTGAACA
TCGAGGCCGAGCCTCCCTTTGGCGACTCCTACATCATCATTGGCGTGGAGCCCGG
ACAGCTCAAGCTGAACTGGTTCAAGAAGGGCAGCAGCGGCGGCAGCCATCACCA
CCATCATCACCATCAT

Protein
MKWVTFISLLFLFSSAYSMRCIGMSNRDFVEGVSGGSWVDIVLEHGSCVTTMAKNKPT
LDFELIKTEAKQPATLRKYCIEAKLTNTTTESRCPTQGEPSLNEEQDKRFVCKHSMVDR
GWGNGCGLFGKGGIVTCAMFRCKKNMEGKVVIPENLEYTIVITPHSGEEHAVGNDTG
KHGKEIKITPQSSITEAELTGYGTVTMECSPRTGLDFNEMVLLQMENKAWLVHRQWFL
DLPLPWLPGADTQGSNWIQKETLVTFKNPHAKKQDVVVLGSQEGAMHTALTGATEIQ
MSSGNLLFTGHLKCRLRMDKLQLKGMSLSMCTGKFKVVKEIAETQHGTIVIRVQYEGD
GSPCKIPFEIMDLEKRHVLGRLITVNPIVTEKDSPVNIEAEPPFGDSYIIIGVEPGQLKLN
WFKKGSSGGSHHHHHHHH

pD2sE_EV8_PM3

DNA
ATGAAGTGGGTAACCTTTATTTCCCTTCTTTTTCTCTTTAGCTCGGCTTATTCCATGA
GGTGTATCGGCATGTCCAACAGGGACTTTGTGGAGGGAGTGAGCGGCGGCAGCT
GGGTGGACATTGTGCTGGAGCATGGAAGCTGCGTGACCACGATGATGAAAAACAA
GCCCACCCTGGACTTCGAGCTCATCAAGACAGAGGCTAAACAGCCCGCCACCCTC
AGGAAGTACTGCATCGAGGCCAAGCTGACCAACACAACAACCGAGTCCAGATGCC
CTACACAGGGCGAACCCAGCCTCAACGAAGAGCAGGACAAGAGGTTCGTGTGCAA
ACACAGCATGGTGGACAGGGGCTGGGGCAATGGATGCGGACTCTTCGGAAAAGG
CGGCATCGTGACCTGCGCCATGTTCAGGTGTAAAAAGAACATGGAAGGCAAGGTG
GTGCAGCCCGAAAATCTGGAGTATACCATCGTGATTACCCCCCACAGCGGAGAGG
AGCACGCCGTGGGCAATGACACCGGCAAGCACGGCAAAGAGATTAAGATCACCC
CCCAGTCCTCCATTACCGAAGCTGAACTGACAGGCTACGGCACCGTGACAATGGA
GTGTAGCCCCAGGACCGGACTGGATTTCAACGAGATGGTCCTGCTACAGATGGAG
AACAAGGCCTGGCTCGTGCACAGGCAATGGTTTCTGGATCTGCCTCTGCCTTGGC
TGCCTGGCGCCGATACACAGGGCTCCAACTGGATACAGAAAGAGACCCTCGTCAC
CTTCAAGAATCCCCATGCTAAGAAGCAGGACGTGGTGGTGCTGGGCAGCCAAGAA
GGCGCCATGCACACAGCCCTGACCGGAGCTACCGAGATCCAGATGAGCTCCGGC
AACCTGCTGTTCACCGGCCATCTGAAATGTAGGCTGAGGTGGGATAAGCTGCAAC
TCAAAGGCATGTCCTACTCCATGTGCACCGGAAAGTTCAAGGTGGTGAAAGAAATC
GCCGAAACACAGCACGGCACCATCGTGATCAGGGTGCAGTATGAGGGCGACGGC
TCCCCCTGTAAGATCCCCTTCGAAATCATGGACCTGGAAAAGAGGCACGTGCTGG
GCAGGCTCATCACCGTGAACCCCATTGTCACAGAGAAAGACTCCCCCGTGAACAT
CGAGGCCGAGCCTCCCTTTGGCGACTCCTACATCATCATTGGCGTGGAGCCCGGA
CAGCTCAAGCTGAACTGGTTCAAGAAGGGCAGCAGCGGCGGCAGCCATCACCAC
CATCATCACCATCAT

Protein
MKWVTFISLLFLFSSAYSMRCIGMSNRDFVEGVSGGSWVDIVLEHGSCVTTMMKNKP
TLDFELIKTEAKQPATLRKYCIEAKLTNTTTESRCPTQGEPSLNEEQDKRFVCKHSMVD
RGWGNGCGLFGKGGIVTCAMFRCKKNMEGKVVQPENLEYTIVITPHSGEEHAVGNDT
GKHGKEIKITPQSSITEAELTGYGTVTMECSPRTGLDFNEMVLLQMENKAWLVHRQWF
LDLPLPWLPGADTQGSNWIQKETLVTFKNPHAKKQDVVVLGSQEGAMHTALTGATEIQ
MSSGNLLFTGHLKCRLRWDKLQLKGMSYSMCTGKFKVVKEIAETQHGTIVIRVQYEGD
GSPCKIPFEIMDLEKRHVLGRLITVNPIVTEKDSPVNIEAEPPFGDSYIIIGVEPGQLKLN
WFKKGSSGGSHHHHHHHH

pD2sE_EV8_PM4

DNA
ATGAAGTGGGTAACCTTTATTTCCCTTCTTTTTCTCTTTAGCTCGGCTTATTCCATGA
GGTGTATCGGCATGTCCAACAGGGACTTTGTGGAGGGAGTGAGCGGCGGCAGCT
GGGTGGACATTGTGCTGGAGCATGGAAAGTGCGTGACCGTGATGATGAAAAACAA
GCCCACCCTGGACTTCGAGCTCATCAAGACAGAGGCTAAACAGCCCGCCACCCTC
AGGAAGTACTGCATCGAGGCCAAGCTGACCAACACAACAACCGAGTCCAGATGCC
CTACACAGGGCGAACCCAGCCTCAACGAAGAGCAGGACAAGAGGTTCGTGTGCAA
ACACAGCATGGTGGACAGGGGCTGGGGCAATGGATGCGGACTCTTCGGAAAAGG
CGGCATCGTGACCTGCGCCATGTTCAGGTGTAAAAAGAACATGGAAGGCAAGGTG
GTGCAGCCCGAAAATCTGGAGTATACCATCGTGATTACCCCCCACAGCGGAGAGG
AGCACGCCGTGGGCAATGACACCGGCAAGCACGGCAAAGAGATTAAGATCACCC
CCCAGTCCTCCATTACCGAAGCTGAACTGACAGGCTACGGCACCGTGACAATGGA
GTGTAGCCCCAGGACCGGACTGGATTTCAACGAGATGGTCCTGCTACAGATGGAG
AACAAGGCCTGGCTCGTGCACAGGCAATGGTTTCTGGATCTGCCTCTGCCTTGGC
TGCCTGGCGCCGATACACAGGGCTCCAACTGGATACAGAAAGAGACCCTCGTCAC
CTTCAAGAATCCCCATGCTAAGAAGCAGGACGTGGTGGTGCTGGGCAGCCAAGAA
GGCGCCATGCACACAGCCCTGACCGGAGCTACCGAGATCCAGATGAGCTCCGGC
AACCTGCTGTTCACCGGCCATCTGAAATGTAGGCTGAGGATGGATAAGCTGCAAC
TCAAAGGCATGTCCTACTCCATGTGCACCGGAAAGTTCAAGGTGGTGAAAGAAATC
GCCGAAACACAGCACGGCACCATCGTGATCAGGGTGCAGTATGAGGGCGACGGC
TCCCCCTGTAAGATCCCCTTCGAAATCATGGACCTGGAAAAGAGGCACGTGCTGG
GCAGGCTCATCACCGTGAACCCCATTGTCACAGAGAAAGACTCCCCCGTGAACAT
CGAGGCCGAGCCTCCCTTTGGCGACTCCTACATCATCATTGGCGTGGAGCCCGGA
CAGCTCAAGCTGAACTGGTTCAAGAAGGGCAGCAGCGGCGGCAGCCATCACCAC
CATCATCACCATCAT

Protein
MKWVTFISLLFLFSSAYSMRCIGMSNRDFVEGVSGGSWVDIVLEHGKCVTVMMKNKP
TLDFELIKTEAKQPATLRKYCIEAKLTNTTTESRCPTQGEPSLNEEQDKRFVCKHSMVD
RGWGNGCGLFGKGGIVTCAMFRCKKNMEGKVVQPENLEYTIVITPHSGEEHAVGNDT
GKHGKEIKITPQSSITEAELTGYGTVTMECSPRTGLDFNEMVLLQMENKAWLVHRQWF
LDLPLPWLPGADTQGSNWIQKETLVTFKNPHAKKQDVVVLGSQEGAMHTALTGATEIQ
MSSGNLLFTGHLKCRLRMDKLQLKGMSYSMCTGKFKVVKEIAETQHGTIVIRVQYEGD
GSPCKIPFEIMDLEKRHVLGRLITVNPIVTEKDSPVNIEAEPPFGDSYIIIGVEPGQLKLN
WFKKGSSGGSHHHHHHHH

pD2sE_EV8_PM4.1

DNA
ATGAAGTGGGTAACCTTTATTTCCCTTCTTTTTCTCTTTAGCTCGGCTTATTCCATGA
GGTGTATCGGCATGTCCAACAGGGACTTTGTGGAGGGAGTGAGCGGCGGCAGCT
GGGTGGACATTGTGCTGGAGCATGGAAGCTGCGTGACCACGATGATGAAAAACAA
GCCCACCCTGGACTTCGAGCTCATCAAGACAGAGGCTAAACAGCCCGCCACCCTC
AGGAAGTACTGCATCGAGGCCAAGCTGACCAACACAACAACCGAGTCCAGATGCC
CTACACAGGGCGAACCCAGCCTCAACGAAGAGCAGGACAAGAGGTTCGTGTGCAA
ACACAGCATGGTGGACAGGGGCTGGGGCAATGGATGCGGACTCTTCGGAAAAGG
CGGCATCGTGACCTGCGCCATGTTCAGGTGTAAAAAGAACATGGAAGGCAAGGTG
GTGCAGCCCGAAAATCTGGAGTATACCATCGTGATTACCCCCCACAGCGGAGAGG
AGCACGCCGTGGGCAATGACACCGGCAAGCACGGCAAAGAGATTAAGATCACCC
CCCAGTCCTCCATTACCGAAGCTGAACTGACAGGCTACGGCACCGTGACAATGGA
GTGTAGCCCCAGGACCGGACTGGATTTCAACGAGATGGTCCTGCTACAGATGGAG
AACAAGGCCTGGCTCGTGCACAGGCAATGGTTTCTGGATCTGCCTCTGCCTTGGC
TGCCTGGCGCCGATACACAGGGCTCCAACTGGATACAGAAAGAGACCCTCGTCAC
CTTCAAGAATCCCCATGCTAAGAAGCAGGACGTGGTGGTGCTGGGCAGCCAAGAA
GGCGCCATGCACACAGCCCTGACCGGAGCTACCGAGATCCAGATGAGCTCCGGC
AACCTGCTGTTCACCGGCCATCTGAAATGTAGGCTGAGGATGGATAAGCTGCAAC
TCAAAGGCATGTCCTACTCCATGTGCACCGGAAAGTTCAAGGTGGTGAAAGAAATC
GCCGAAACACAGCACGGCACCATCGTGATCAGGGTGCAGTATGAGGGCGACGGC
TCCCCCTGTAAGATCCCCTTCGAAATCATGGACCTGGAAAAGAGGCACGTGCTGG
GCAGGCTCATCACCGTGAACCCCATTGTCACAGAGAAAGACTCCCCCGTGAACAT
CGAGGCCGAGCCTCCCTTTGGCGACTCCTACATCATCATTGGCGTGGAGCCCGGA
CAGCTCAAGCTGAACTGGTTCAAGAAGGGCAGCAGCGGCGGCAGCCATCACCAC
CATCATCACCATCAT

Protein
MKWVTFISLLFLFSSAYSMRCIGMSNRDFVEGVSGGSWVDIVLEHGSCVTTMMKNKP
TLDFELIKTEAKQPATLRKYCIEAKLTNTTTESRCPTQGEPSLNEEQDKRFVCKHSMVD
RGWGNGCGLFGKGGIVTCAMFRCKKNMEGKVVQPENLEYTIVITPHSGEEHAVGNDT
GKHGKEIKITPQSSITEAELTGYGTVTMECSPRTGLDFNEMVLLQMENKAWLVHRQWF
LDLPLPWLPGADTQGSNWIQKETLVTFKNPHAKKQDVVVLGSQEGAMHTALTGATEIQ
MSSGNLLFTGHLKCRLRMDKLQLKGMSYSMCTGKFKVVKEIAETQHGTIVIRVQYEGD
GSPCKIPFEIMDLEKRHVLGRLITVNPIVTEKDSPVNIEAEPPFGDSYIIIGVEPGQLKLN
WFKKGSSGGSHHHHHHHH

pD2sE_EV8_PM4.2

DNA
ATGAAGTGGGTAACCTTTATTTCCCTTCTTTTTCTCTTTAGCTCGGCTTATTCCATGA
GGTGTATCGGCATGTCCAACAGGGACTTTGTGGAGGGAGTGAGCGGCGGCAGCT
GGGTGGACATTGTGCTGGAGCATGGAAGCTGCGTGACCGTGATGATGAAAAACAA
GCCCACCCTGGACTTCGAGCTCATCAAGACAGAGGCTAAACAGCCCGCCACCCTC
AGGAAGTACTGCATCGAGGCCAAGCTGACCAACACAACAACCGAGTCCAGATGCC
CTACACAGGGCGAACCCAGCCTCAACGAAGAGCAGGACAAGAGGTTCGTGTGCAA
ACACAGCATGGTGGACAGGGGCTGGGGCAATGGATGCGGACTCTTCGGAAAAGG
CGGCATCGTGACCTGCGCCATGTTCAGGTGTAAAAAGAACATGGAAGGCAAGGTG
GTGCAGCCCGAAAATCTGGAGTATACCATCGTGATTACCCCCCACAGCGGAGAGG
AGCACGCCGTGGGCAATGACACCGGCAAGCACGGCAAAGAGATTAAGATCACCC
CCCAGTCCTCCATTACCGAAGCTGAACTGACAGGCTACGGCACCGTGACAATGGA
GTGTAGCCCCAGGACCGGACTGGATTTCAACGAGATGGTCCTGCTACAGATGGAG
AACAAGGCCTGGCTCGTGCACAGGCAATGGTTTCTGGATCTGCCTCTGCCTTGGC
TGCCTGGCGCCGATACACAGGGCTCCAACTGGATACAGAAAGAGACCCTCGTCAC
CTTCAAGAATCCCCATGCTAAGAAGCAGGACGTGGTGGTGCTGGGCAGCCAAGAA
GGCGCCATGCACACAGCCCTGACCGGAGCTACCGAGATCCAGATGAGCTCCGGC
AACCTGCTGTTCACCGGCCATCTGAAATGTAGGCTGAGGATGGATAAGCTGCAAC
TCAAAGGCATGTCCTACTCCATGTGCACCGGAAAGTTCAAGGTGGTGAAAGAAATC
GCCGAAACACAGCACGGCACCATCGTGATCAGGGTGCAGTATGAGGGCGACGGC
TCCCCCTGTAAGATCCCCTTCGAAATCATGGACCTGGAAAAGAGGCACGTGCTGG
GCAGGCTCATCACCGTGAACCCCATTGTCACAGAGAAAGACTCCCCCGTGAACAT
CGAGGCCGAGCCTCCCTTTGGCGACTCCTACATCATCATTGGCGTGGAGCCCGGA
CAGCTCAAGCTGAACTGGTTCAAGAAGGGCAGCAGCGGCGGCAGCCATCACCAC
CATCATCACCATCAT

Protein
MKWVTFISLLFLFSSAYSMRCIGMSNRDFVEGVSGGSWVDIVLEHGSCVTVMMKNKP
TLDFELIKTEAKQPATLRKYCIEAKLTNTTTESRCPTQGEPSLNEEQDKRFVCKHSMVD
RGWGNGCGLFGKGGIVTCAMFRCKKNMEGKVVQPENLEYTIVITPHSGEEHAVGNDT
GKHGKEIKITPQSSITEAELTGYGTVTMECSPRTGLDFNEMVLLQMENKAWLVHRQWF
LDLPLPWLPGADTQGSNWIQKETLVTFKNPHAKKQDVVVLGSQEGAMHTALTGATEIQ
MSSGNLLFTGHLKCRLRMDKLQLKGMSYSMCTGKFKVVKEIAETQHGTIVIRVQYEGD
GSPCKIPFEIMDLEKRHVLGRLITVNPIVTEKDSPVNIEAEPPFGDSYIIIGVEPGQLKLN
WFKKGSSGGSHHHHHHHH

pD2sE_EV8_PM4.3

DNA
ATGAAGTGGGTAACCTTTATTTCCCTTCTTTTTCTCTTTAGCTCGGCTTATTCCATGA
GGTGTATCGGCATGTCCAACAGGGACTTTGTGGAGGGAGTGAGCGGCGGCAGCT
GGGTGGACATTGTGCTGGAGCATGGAAAGTGCGTGACCACGATGGCGAAAAACAA
GCCCACCCTGGACTTCGAGCTCATCAAGACAGAGGCTAAACAGCCCGCCACCCTC
AGGAAGTACTGCATCGAGGCCAAGCTGACCAACACAACAACCGAGTCCAGATGCC
CTACACAGGGCGAACCCAGCCTCAACGAAGAGCAGGACAAGAGGTTCGTGTGCAA
ACACAGCATGGTGGACAGGGGCTGGGGCAATGGATGCGGACTCTTCGGAAAAGG
CGGCATCGTGACCTGCGCCATGTTCAGGTGTAAAAAGAACATGGAAGGCAAGGTG
GTGCAGCCCGAAAATCTGGAGTATACCATCGTGATTACCCCCCACAGCGGAGAGG
AGCACGCCGTGGGCAATGACACCGGCAAGCACGGCAAAGAGATTAAGATCACCC
CCCAGTCCTCCATTACCGAAGCTGAACTGACAGGCTACGGCACCGTGACAATGGA
GTGTAGCCCCAGGACCGGACTGGATTTCAACGAGATGGTCCTGCTACAGATGGAG
AACAAGGCCTGGCTCGTGCACAGGCAATGGTTTCTGGATCTGCCTCTGCCTTGGC
TGCCTGGCGCCGATACACAGGGCTCCAACTGGATACAGAAAGAGACCCTCGTCAC
CTTCAAGAATCCCCATGCTAAGAAGCAGGACGTGGTGGTGCTGGGCAGCCAAGAA
GGCGCCATGCACACAGCCCTGACCGGAGCTACCGAGATCCAGATGAGCTCCGGC
AACCTGCTGTTCACCGGCCATCTGAAATGTAGGCTGAGGATGGATAAGCTGCAAC
TCAAAGGCATGTCCTACTCCATGTGCACCGGAAAGTTCAAGGTGGTGAAAGAAATC
GCCGAAACACAGCACGGCACCATCGTGATCAGGGTGCAGTATGAGGGCGACGGC
TCCCCCTGTAAGATCCCCTTCGAAATCATGGACCTGGAAAAGAGGCACGTGCTGG
GCAGGCTCATCACCGTGAACCCCATTGTCACAGAGAAAGACTCCCCCGTGAACAT
CGAGGCCGAGCCTCCCTTTGGCGACTCCTACATCATCATTGGCGTGGAGCCCGGA
CAGCTCAAGCTGAACTGGTTCAAGAAGGGCAGCAGCGGCGGCAGCCATCACCAC
CATCATCACCATCAT

Protein
MKWVTFISLLFLFSSAYSMRCIGMSNRDFVEGVSGGSWVDIVLEHGKCVTTMAKNKPT
LDFELIKTEAKQPATLRKYCIEAKLTNTTTESRCPTQGEPSLNEEQDKRFVCKHSMVDR
GWGNGCGLFGKGGIVTCAMFRCKKNMEGKVVQPENLEYTIVITPHSGEEHAVGNDTG
KHGKEIKITPQSSITEAELTGYGTVTMECSPRTGLDFNEMVLLQMENKAWLVHRQWFL
DLPLPWLPGADTQGSNWIQKETLVTFKNPHAKKQDVVVLGSQEGAMHTALTGATEIQ
MSSGNLLFTGHLKCRLRMDKLQLKGMSYSMCTGKFKVVKEIAETQHGTIVIRVQYEGD
GSPCKIPFEIMDLEKRHVLGRLITVNPIVTEKDSPVNIEAEPPFGDSYIIIGVEPGQLKLN
WFKKGSSGGSHHHHHHHH

pD2sE_EV8_PM5

DNA
ATGAAGTGGGTAACCTTTATTTCCCTTCTTTTTCTCTTTAGCTCGGCTTATTCCATGA
GGTGTATCGGCATGTCCAACAGGGACTTTGTGGAGGGAGTGAGCGGCGGCAGCT
GGGTGGACATTGTGCTGGAGCATGGAAGCTGCGTGACCACGATGGCGAAAAACAA
GCCCACCCTGGACTTCGAGCTCATCAAGACAGAGGCTAAACAGCCCGCCACCCTC
AGGAAGTACTGCATCGAGGCCAAGCTGACCAACACAACAACCGAGTCCAGATGCC
CTACACAGGGCGAACCCAGCCTCAACGAAGAGCAGGACAAGAGGTTCGTGTGCAA
ACACAGCATGGTGGACAGGGGCTGGGGCAATGGATGCGGACTCTTCGGAAAAGG
CGGCATCGTGACCTGCGCCATGTTCAGGTGTAAAAAGAACATGGAAGGCAAGGTG
GTGCAGCCCGAAAATCTGGAGTATACCATCGTGATTACCCCCCACAGCGGAGAGG
AGCACGCCGTGGGCAATGACACCGGCAAGCACGGCAAAGAGATTAAGATCACCC
CCCAGTCCTCCATTACCGAAGCTGAACTGACAGGCTACGGCACCGTGACAATGGA
GTGTAGCCCCAGGACCGGACTGGATTTCAACGAGATGGTCCTGCTACAGATGGAG
AACAAGGCCTGGCTCGTGGACAGGCAATGGTTTCTGGATCTGCCTCTGCCTTGGC
TGCCTGGCGCCGATACACAGGGCTCCAACTGGATACAGAAAGAGACCCTCGTCAC
CTTCAAGAATCCCCATGCTAAGAAGCAGGACGTGGTGGTGCTGGGCAGCCAAGAA
GGCGCCATGCACACAGCCCTGACCTGGGCTACCGAGATCCAGATGAGCTCCGGC
AACCTGCTGTTCACCGGCCATCTGAAATGTAGGCTGAGGATGGATAAGCTGCAAC
TCAAAGGCATGTCCTACTCCATGTGCACCGGAAAGTTCAAGGTGGTGAAAGAAATC
GCCGAAACACAGCACGGCACCATCGTGATCAGGGTGCAGTATGAGGGCGACGGC
TCCCCCTGTAAGATCCCCTTCGAAATCATGGACCTGGAAAAGAGGCACGTGCTGG
GCAGGCTCATCACCGTGAACCCCATTGTCACAGAGAAAGACTCCCCCGTGAACAT
CGAGGCCGAGCCTCCCTTTGGCGACTCCTACATCATCATTGGCGTGGAGCCCGGA
CAGCTCAAGCTGAACTGGTTCAAGAAGGGCAGCAGCGGCGGCAGCCATCACCAC
CATCATCACCATCAT

Protein
MKWVTFISLLFLFSSAYSMRCIGMSNRDFVEGVSGGSWVDIVLEHGSCVTTMAKNKPT
LDFELIKTEAKQPATLRKYCIEAKLTNTTTESRCPTQGEPSLNEEQDKRFVCKHSMVDR
GWGNGCGLFGKGGIVTCAMFRCKKNMEGKVVQPENLEYTIVITPHSGEEHAVGNDTG
KHGKEIKITPQSSITEAELTGYGTVTMECSPRTGLDFNEMVLLQMENKAWLVDRQWFL
DLPLPWLPGADTQGSNWIQKETLVTFKNPHAKKQDVVVLGSQEGAMHTALTWATEIQ
MSSGNLLFTGHLKCRLRMDKLQLKGMSYSMCTGKFKVVKEIAETQHGTIVIRVQYEGD
GSPCKIPFEIMDLEKRHVLGRLITVNPIVTEKDSPVNIEAEPPFGDSYIIIGVEPGQLKLN
WFKKGSSGGSHHHHHHHH

pD2sE_EV8_SHP1

DNA
ATGAAGTGGGTAACCTTTATTTCCCTTCTTTTTCTCTTTAGCTCGGCTTATTCCATGA
GGTGTATCGGCATGTCCAACAGGGACTTTGTGGAGGGAGAGAGCGGCGGCAGCT
GGGTGGACATTGTGCTGGAGCATGGAAGCTGCGTGACCACGATGGCGAAAAACAA
GCCCACCCTGGACTTCGAGCTCATCAAGACAGAGGCTAAACAGCCCGCCACCCTC
AGGAAGTACTGCATCGAGGCCAAGCTGACCAACACAACAACCGAGTCCAGATGCC
CTACACAGGGCGAACCCAGCCTCAACGAAGAGCAGGACAAGAGGTTCGTGTGCAA
ACACAGCATGGTGGACAGGGGCTGGGGCAATGGATGCGGACTCTTCGGAAAAGG
CGGCATCGTGACCTGCGCCATGTTCAGGTGTAAAAAGAACATGGAAGGCAAGGTG
GTGCAGCCCGAAAATCTGGAGTATACCATCGTGATTACCCCCCACAGCGGAGAGG
AGCACGCCGTGGGCAATGACACCGGCAAGCACGGCAAAGAGATTAAGATCACCC
CCCAGTCCTCCATTACCGAAGCTGAACTGACAGGCTACGGCACCGTGACAATGGA
GTGTAGCCCCAGGACCGGACTGGATTTCAACGAGATGGTCCTGCTACAGATGGAG
AACAAGGCCTGGCTCGTGCACAGGCAATGGTTTCTGGATCTGCCTCTGCCTTGGC
TGCCTGGCGCCGATACACAGGGCTCCAACTGGATACAGAAAGAGACCCTCGTCAC
CTTCAAGAATCCCCATGCTAAGAAGCAGGACGTGGTGGTGCTGGGCAGCCAAGAA
GGCGCCATGCACACAGCCCTGACCGGAGCTACCGAGATCCAGATGAGCTCCGGC
AACCTGCTGTTCACCGGCCATCTGAAATGTAGGCTGAGGATGGATAAGCTGCAAC
TCAAAGGCATGTCCTACTCCATGTGCACCGGAAAGTTCAAGGTGGTGAAAGAAATC
GCCGAAACACAGCACGGCACCATCGTGATCAGGGTGCAGTATGAGGGCGACGGC
TCCCCCTGTAAGATCCCCTTCGAAATCATGGACCTGGAAAAGAGGCACGTGCTGG
GCAGGCTCATCACCGTGAACCCCATTGTCACAGAGAAAGACTCCCCCGTGAACAT
CGAGGCCGAGCCTCCCGACGGCGACTCCTACATCATCATTGGCGTGGAGCCCGG
ACAGCTCAAGCTGAACTGGAGAAAGAAGGGCAGCAGCGGCGGCAGCCATCACCA
CCATCATCACCATCAT

Protein
MKWVTFISLLFLFSSAYSMRCIGMSNRDFVEGESGGSWVDIVLEHGSCVTTMAKNKPT
LDFELIKTEAKQPATLRKYCIEAKLTNTTTESRCPTQGEPSLNEEQDKRFVCKHSMVDR
GWGNGCGLFGKGGIVTCAMFRCKKNMEGKVVQPENLEYTIVITPHSGEEHAVGNDTG
KHGKEIKITPQSSITEAELTGYGTVTMECSPRTGLDFNEMVLLQMENKAWLVHRQWFL
DLPLPWLPGADTQGSNWIQKETLVTFKNPHAKKQDVVVLGSQEGAMHTALTGATEIQ
MSSGNLLFTGHLKCRLRMDKLQLKGMSYSMCTGKFKVVKEIAETQHGTIVIRVQYEGD
GSPCKIPFEIMDLEKRHVLGRLITVNPIVTEKDSPVNIEAEPPDGDSYIIIGVEPGQLKLN
WRKKGSSGGSHHHHHHHH

pD2sE_EV8_UndPk4

DNA
ATGAAGTGGGTAACCTTTATTTCCCTTCTTTTTCTCTTTAGCTCGGCTTATTCCATGA
GGTGTATCGGCATGTCCAACAGGGACTTTGTGTTCGCCGTGAGCGGCGGCAGCTG
GGTGGACATTGTGCTGGAGCATGGAAGCTGCGTGACCACGCTGGCGAAAAACAA
GCCCACCCTGGACTTCGAGCTCATCAAGACAGAGGCTAAACAGCCCGCCACCCTC
AGGAAGTACTGCATCGAGGCCAAGCTGACCAACACAACAACCGAGTCCAGATGCC
CTACACAGGGCGAACCCAGCCTCAACGAAGAGCAGGACAAGAGGTTCGTGTGCAA
ACACAGCATGGTGGACAGGGGCTGGGGCAATGGATGCGGACTCTTCGGAAAAGG
CGGCATCGTGACCTGCGCCATGTTCAGGTGTAAAAAGAACATGGAAGGCAAGGTG
GTGCAGCCCGAAAATCTGGAGTATACCATCGTGATTACCCCCCACAGCGGAGAGG
AGCACGCCGTGGGCAATGACACCGGCAAGCACGGCAAAGAGATTAAGATCACCC
CCCAGTCCTCCATTACCGAAGCTGAACTGACAGGCTACGGCACCGTGACAATGGA
GTGTAGCCCCAGGACCGGACTGGATTTCAACGAGATGGTCCTGCTACAGATGGAG
AACAAGGCCTGGCTCGTGCACAGGCAATGGTTTCTGGATCTGCCTCTGCCTTGGC
TGCCTGGCGCCGATACACAGGGCTCCAACTGGATACAGAAAGAGACCCTCGTCAC
CTTCAAGAATCCCCATGCTAAGAAGCAGGACGTGGTGGTGCTGGGCAGCCAAGAA
GGCGCCATGCACACAGCCCTGACCGGAGCTACCGAGATCCAGATGAGCTCCGGC
AACCTGCTGTTCACCGGCCATCTGAAATGTAGGCTGAGGATGGATAAGCTGCAAC
TCAAAGGCATGTCCTACTCCATGTGCACCGGAAAGTTCAAGGTGGTGAAAGAAATC
GCCGAAACACAGCACGGCACCATCGTGATCAGGGTGCAGTATGAGGGCGACGGC
TCCCCCTGTAAGATCCCCTTCGAAATCATGGACCTGGAAAAGAGGCACGTGCTGG
GCAGGCTCATCACCGTGAACCCCATTGTCACAGAGAAAGACTCCCCCGTGAACAT
CGAGGCCGAGCCTCCCTTTGGCGACTCCTACATCATCATTGGCGTGGAGCCCGGA
CAGCTCAAGCTGAACTGGTTCAAGAAGGGCAGCAGCGGCGGCAGCCATCACCAC
CATCATCACCATCAT

Protein
MKWVTFISLLFLFSSAYSMRCIGMSNRDFVFAVSGGSWVDIVLEHGSCVTTLAKNKPT
LDFELIKTEAKQPATLRKYCIEAKLTNTTTESRCPTQGEPSLNEEQDKRFVCKHSMVDR
GWGNGCGLFGKGGIVTCAMFRCKKNMEGKVVQPENLEYTIVITPHSGEEHAVGNDTG
KHGKEIKITPQSSITEAELTGYGTVTMECSPRTGLDFNEMVLLQMENKAWLVHRQWFL
DLPLPWLPGADTQGSNWIQKETLVTFKNPHAKKQDVVVLGSQEGAMHTALTGATEIQ
MSSGNLLFTGHLKCRLRMDKLQLKGMSYSMCTGKFKVVKEIAETQHGTIVIRVQYEGD
GSPCKIPFEIMDLEKRHVLGRLITVNPIVTEKDSPVNIEAEPPFGDSYIIIGVEPGQLKLN
WFKKGSSGGSHHHHHHHH

pD2sE_EV8_UndPk5

DNA
ATGAAGTGGGTAACCTTTATTTCCCTTCTTTTTCTCTTTAGCTCGGCTTATTCCATGA
GGTGTATCGGCATGTCCAACAGGGACTTTGTGGAGGGAGTGAGCGGCGGCAGCT
GGGTGGACATTGTGCTGGAGCATGGAAGCTGCGTGACCACGATGGCGAAAAACAA
GCCCACCCTGGACTTCGAGCTCATCAAGACAGAGGCTAAACAGCCCGCCACCCTC
AGGAAGTACTGCATCGAGGCCAAGCTGACCAACACAACAACCGAGTCCAGATGCC
CTACACAGGGCGAACCCAGCCTCAACGAAGAGCAGGACAAGAGGTTCGTGTGCAA
ACACAGCATGGTGGACAGGGGCTGGGGCAATGGATGCGGACTCTTCGGAAAAGG
CGGCATCGTGACCTGCGCCATGTTCAGGTGTAAAAAGAACATGGAAGGCAAGGTG
GTGCAGCCCGAAAATCTGGAGTATACCATCGTGATTACCCCCCACAGCGGAGAGG
AGCACGCCGTGGGCAATGACACCGGCAAGCACGGCAAAGAGATTAAGATCACCC
CCCAGTCCTCCATTACCGAAGCTGAACTGACAGGCTACGGCACCGTGACAATGGA
GTGTAGCCCCAGGACCGGACTGGATTTCAACGAGATGGTCCTGCTACAGATGGAG
AACAAGGCCTGGCTCGTGCACAGGCAATGGTTTCTGGATCTGCCTCTGCCTTGGC
TGCCTGGCGCCGATACACAGGGCTCCAACTGGATACAGAAAGAGACCCTCGTCAC
CTTCAAGAATCCCCATGCTAAGAAGCAGGACGTGGTGGTGCTGGGCAGCCAAGAA
GGCGCCATGCACACAGCCCTGACCGGAGCTACCGAGATCCAGATGAGCTCCGGC
AACCTGCTGTTCACCGGCCATCTGAAATGTAGGCTGAGGATGGATAAGCTGCAAC
TCAAAGGCATGTCCTACTCCATGTGCACCGGAAAGTTCAAGGTGGTGAAAGAAATC
GCCGAAACACAGCACGGCACCATCGTGATCAGGGTGCAGTATGAGGGCGACGGC
TCCCCCTGTAAGATCCCCTTCGAAATCATGGACCTGGAAAAGAGGCACGTGCTGG
GCAGGCTCATCACCGTGAACCCCATTGTCTACGAGAAAGACTCCCCCGTGAACAT
CGAGGCCGAGCCTCCCTTTGGCGACTCCTACATCATCATTGGCGTGGAGCCCGGA
CAGCTCAAGCTGAACTGGTTCAAGAAGGGCAGCAGCGGCGGCAGCCATCACCAC
CATCATCACCATCAT

Protein
MKWVTFISLLFLFSSAYSMRCIGMSNRDFVEGVSGGSWVDIVLEHGSCVTTMAKNKPT
LDFELIKTEAKQPATLRKYCIEAKLTNTTTESRCPTQGEPSLNEEQDKRFVCKHSMVDR
GWGNGCGLFGKGGIVTCAMFRCKKNMEGKVVQPENLEYTIVITPHSGEEHAVGNDTG
KHGKEIKITPQSSITEAELTGYGTVTMECSPRTGLDFNEMVLLQMENKAWLVHRQWFL
DLPLPWLPGADTQGSNWIQKETLVTFKNPHAKKQDVVVLGSQEGAMHTALTGATEIQ
MSSGNLLFTGHLKCRLRMDKLQLKGMSYSMCTGKFKVVKEIAETQHGTIVIRVQYEGD
GSPCKIPFEIMDLEKRHVLGRLITVNPIVYEKDSPVNIEAEPPFGDSYIIIGVEPGQLKLN
WFKKGSSGGSHHHHHHHH

pD2sE_EV8_UndPk6

DNA
ATGAAGTGGGTAACCTTTATTTCCCTTCTTTTTCTCTTTAGCTCGGCTTATTCCATGA
GGTGTATCGGCATGTCCAACAGGGACTTTGTGGAGGGAGTGAGCGGCGGCAGCT
GGGTGGACATTGTGCTGGAGCATGGAAGCTGCGTGACCACGATGGCGAAAAACAA
GCCCACCCTGGACTTCGAGCTCATCAAGACAGAGGCTAAACAGCCCGCCACCCTC
AGGAAGTACTGCATCGAGGCCAAGCTGACCAACACAACAACCGAGTCCAGATGCC
CTACACAGGGCGAACCCAGCCTCAACGAAGAGCAGGACAAGAGGTTCGTGTGCAA
ACACAGCATGGTGGACAGGGGCTGGGGCAATGGATGCGGACTCTTCGGAAAAGG
CGGCATCGTGACCTGCGCCATGTTCAGGTGTAAAAAGAACATGGAAGGCAAGGTG
GTGCAGCCCGAAAATCTGGAGTATACCATCGTGATTACCCCCCACAGCGGAGAGG
AGCACGCCGTGGGCAATGACACCGGCAAGCACGGCAAAGAGATTAAGATCACCC
CCCAGTCCTCCATTACCGAAGCTGAACTGACAGGCTACGGCACCGTGACAATGGA
GTGTAGCCCCAGGACCGGACTGGATTTCAACGAGATGGTCCTGCTACAGATGGAG
AACAAGGCCTGGCTCGTGCACAGGCAATGGTTTCTGGATCTGCCTCTGCCTTGGC
TGCCTGGCGCCGATACACAGGGCTCCAACTGGATACAGAAAGAGACCCTCGTCAC
CTTCAAGAATCCCCATGCTAAGAAGCAGGACGTGGTGGTGCTGGGCAGCCAAGAA
GGCGCCATGCACACAGCCCTGACCGGAGCTACCGAGATCCAGATGAGCTCCGGC
AACCTGCTGTGGCCCGGCCATCTGAAATGTAGGCTGAGGATGGATAAGCTGCAAC
TCAAAGGCATGTCCTACTCCATGTGCACCGGAAAGTTCAAGGTGGTGAAAGAAATC
GCCGAAACACAGCACGGCACCATCGTGATCAGGGTGCAGTATGAGGGCGACGGC
TCCCCCTGTAAGATCCCCTTCGAAATCATGGACCTGGAAAAGAGGCACGTGCTGG
GCAGGCTCATCACCGTGAACCCCATTGTCACAGAGAAAGACTCCCCCGTGAACAT
CGAGGCCGAGCCTCCCTTTGGCGACTCCTACATCATCATTGGCGTGGAGCCCGGA
CAGCTCAAGCTGAACTGGTTCAAGAAGGGCAGCAGCGGCGGCAGCCATCACCAC
CATCATCACCATCAT

Protein
MKWVTFISLLFLFSSAYSMRCIGMSNRDFVEGVSGGSWVDIVLEHGSCVTTMAKNKPT
LDFELIKTEAKQPATLRKYCIEAKLTNTTTESRCPTQGEPSLNEEQDKRFVCKHSMVDR
GWGNGCGLFGKGGIVTCAMFRCKKNMEGKVVQPENLEYTIVITPHSGEEHAVGNDTG
KHGKEIKITPQSSITEAELTGYGTVTMECSPRTGLDFNEMVLLQMENKAWLVHRQWFL
DLPLPWLPGADTQGSNWIQKETLVTFKNPHAKKQDVVVLGSQEGAMHTALTGATEIQ
MSSGNLLWPGHLKCRLRMDKLQLKGMSYSMCTGKFKVVKEIAETQHGTIVIRVQYEG
DGSPCKIPFEIMDLEKRHVLGRLITVNPIVTEKDSPVNIEAEPPFGDSYIIIGVEPGQLKLN
WFKKGSSGGSHHHHHHHH

pD2sE_EV8_UndPk6.1

DNA
ATGAAGTGGGTAACCTTTATTTCCCTTCTTTTTCTCTTTAGCTCGGCTTATTCCATGA
GGTGTATCGGCATGTCCAACAGGGACTTTGTGGAGGGAGTGAGCGGCGGCAGCT
GGGTGGACATTGTGCTGGAGCATGGAAGCTGCGTGACCACGATGGCGAAAAACAA
GCCCACCCTGGACTTCGAGCTCATCAAGACAGAGGCTAAACAGCCCGCCACCCTC
AGGAAGTACTGCATCGAGGCCAAGCTGACCAACACAACAACCGAGTCCAGATGCC
CTACACAGGGCGAACCCAGCCTCAACGAAGAGCAGGACAAGAGGTTCGTGTGCAA
ACACAGCATGGTGGACAGGGGCTGGGGCAATGGATGCGGACTCTTCGGAAAAGG
CGGCATCGTGACCTGCGCCATGTTCAGGTGTAAAAAGAACATGGAAGGCAAGGTG
GTGCAGCCCGAAAATCTGGAGTATACCATCGTGATTACCCCCCACAGCGGAGAGG
AGCACGCCGTGGGCAATGACACCGGCAAGCACGGCAAAGAGATTAAGATCACCC
CCCAGTCCTCCATTACCGAAGCTGAACTGACAGGCTACGGCACCGTGACAATGGA
GTGTAGCCCCAGGACCGGACTGGATTTCAACGAGATGGTCCTGCTACAGATGGAG
AACAAGGCCTGGCTCGTGCACAGGCAATGGTTTCTGGATCTGCCTCTGCCTTGGC
TGCCTGGCGCCGATACACAGGGCTCCAACTGGATACAGAAAGAGACCCTCGTCAC
CTTCAAGAATCCCCATGCTAAGAAGCAGGACGTGGTGGTGCTGGGCAGCCAAGAA
GGCGCCATGCACACAGCCCTGACCGGAGCTACCGAGATCCAGATGAGCTCCGGC
AACCTGCTGTTCCCCGGCCATCTGAAATGTAGGCTGAGGATGGATAAGCTGCAAC
TCAAAGGCATGTCCTACTCCATGTGCACCGGAAAGTTCAAGGTGGTGAAAGAAATC
GCCGAAACACAGCACGGCACCATCGTGATCAGGGTGCAGTATGAGGGCGACGGC
TCCCCCTGTAAGATCCCCTTCGAAATCATGGACCTGGAAAAGAGGCACGTGCTGG
GCAGGCTCATCACCGTGAACCCCATTGTCACAGAGAAAGACTCCCCCGTGAACAT
CGAGGCCGAGCCTCCCTTTGGCGACTCCTACATCATCATTGGCGTGGAGCCCGGA
CAGCTCAAGCTGAACTGGTTCAAGAAGGGCAGCAGCGGCGGCAGCCATCACCAC
CATCATCACCATCAT

Protein
MKWVTFISLLFLFSSAYSMRCIGMSNRDFVEGVSGGSWVDIVLEHGSCVTTMAKNKPT
LDFELIKTEAKQPATLRKYCIEAKLTNTTTESRCPTQGEPSLNEEQDKRFVCKHSMVDR
GWGNGCGLFGKGGIVTCAMFRCKKNMEGKVVQPENLEYTIVITPHSGEEHAVGNDTG
KHGKEIKITPQSSITEAELTGYGTVTMECSPRTGLDFNEMVLLQMENKAWLVHRQWFL
DLPLPWLPGADTQGSNWIQKETLVTFKNPHAKKQDVVVLGSQEGAMHTALTGATEIQ
MSSGNLLFPGHLKCRLRMDKLQLKGMSYSMCTGKFKVVKEIAETQHGTIVIRVQYEGD
GSPCKIPFEIMDLEKRHVLGRLITVNPIVTEKDSPVNIEAEPPFGDSYIIIGVEPGQLKLN
WFKKGSSGGSHHHHHHHH

pD2sE_EV8_UndPk7

DNA
ATGAAGTGGGTAACCTTTATTTCCCTTCTTTTTCTCTTTAGCTCGGCTTATTCCATGA
GGTGTATCGGCATGTCCAACAGGGACTTTGTGGAGGGAGTGAGCGGCGGCAGCT
GGGTGGACATTGTGCTGGAGCATGGAAGCTGCGTGACCACGATGGCGAAAAACAA
GCCCACCCTGGACTTCGAGCTCATCAAGATCGAGGCTAAACAGCCCGCCACCCTC
AGGAAGTACTGCATCGAGGCCAAGCTGACCAACACAACAACCGAGTCCAGATGCC
CTACACAGGGCGAACCCAGCCTCAACGAAGAGCAGGACAAGAGGTTCGTGTGCAA
ACACAGCATGGTGGACAGGGGCTGGGGCAATGGATGCGGACTCTTCGGAAAAGG
CGGCATCGTGACCTGCGCCATGTTCAGGTGTAAAAAGAACATGGAAGGCAAGGTG
GTGCAGCCCGAAAATCTGGAGTATACCATCGTGATTACCCCCCACAGCGGAGAGG
AGCACGCCGTGGGCAATGACACCGGCAAGCACGGCAAAGAGATTAAGATCACCC
CCCAGTCCTCCATTACCGAAGCTGAACTGACAGGCTACGGCACCGTGACAATGGA
GTGTAGCCCCAGGACCGGACTGGATTTCAACGAGATGGTCCTGCTACAGATGGAG
AACAAGGCCTGGCTCGTGCACAGGCAATGGTTTCTGGATCTGCCTCTGCCTTGGC
TGCCTGGCGCCGATACACAGGGCTCCAACTGGATACAGAAAGAGACCCTCGTCAC
CTTCAAGAATCCCCATGCTAAGAAGCAGGACGTGGTGGTGCTGGGCAGCCAAGAA
GGCGCCATGCACACAGCCCTGACCGGAGCTACCGAGATCCAGATGAGCTCCGGC
AACATGCTGTTCACCGGCCATCTGAAATGTAGGCTGAGGATGGATAAGCTGCAACT
CAAAGGCATGTCCTACTCCATGTGCACCGGAAAGTTCAAGGTGGTGAAAGAAATC
GCCGAAACACAGCACGGCACCATCGTGATCAGGGTGCAGTATGAGGGCGACGGC
TCCCCCTGTAAGATCCCCTTCGAAATCATGGACCTGGAAAAGAGGCACGTGCTGG
GCAGGCTCATCACCGTGAACCCCATTGTCACAGAGAAAGACTCCCCCGTGAACAT
CGAGGCCGAGCCTCCCTTTGGCGACTCCTACATCATCATTGGCGTGGAGCCCGGA
CAGCTCAAGCTGAACTGGTTCAAGAAGGGCAGCAGCGGCGGCAGCCATCACCAC
CATCATCACCATCAT

Protein
MKWVTFISLLFLFSSAYSMRCIGMSNRDFVEGVSGGSWVDIVLEHGSCVTTMAKNKPT
LDFELIKIEAKQPATLRKYCIEAKLTNTTTESRCPTQGEPSLNEEQDKRFVCKHSMVDR
GWGNGCGLFGKGGIVTCAMFRCKKNMEGKVVQPENLEYTIVITPHSGEEHAVGNDTG
KHGKEIKITPQSSITEAELTGYGTVTMECSPRTGLDFNEMVLLQMENKAWLVHRQWFL
DLPLPWLPGADTQGSNWIQKETLVTFKNPHAKKQDVVVLGSQEGAMHTALTGATEIQ
MSSGNMLFTGHLKCRLRMDKLQLKGMSYSMCTGKFKVVKEIAETQHGTIVIRVQYEG
DGSPCKIPFEIMDLEKRHVLGRLITVNPIVTEKDSPVNIEAEPPFGDSYIIIGVEPGQLKLN
WFKKGSSGGSHHHHHHHH

pD2sE_EV8_UndPk8

DNA
ATGAAGTGGGTAACCTTTATTTCCCTTCTTTTTCTCTTTAGCTCGGCTTATTCCATGA
GGTGTATCGGCATGTCCAACAGGGACTTTGTGGAGGGAGTGAGCGGCGGCAGCT
GGGTGGACATTGTGCTGGAGCATGGAAGCTGCGTGACCACGATGGCGAAAAACAA
GCCCACCCTGGACTTCGAGCTCATCAAGACAGAGGCTAAACAGCCCGCCACCCTC
AGGAAGTACTGCATCGAGGCCAAGCTGACCAACACAACAACCGAGTCCAGATGCC
CTACACAGGGCGAACCCAGCCTCAACGAAGAGCAGGACAAGAGGTTCGTGTGCAA
ACACAGCATGGTGGACAGGGGCTGGGGCAATGGATGCGGACTCTTCGGAAAAGG
CGGCATCGTGACCTGCGCCATGTTCAGGTGTAAAAAGAACATGGAAGGCAAGGTG
GTGCAGCCCGAAAATCTGGAGTATACCATCGTGATTACCCCCCACAGCGGAGAGG
AGCACGCCGTGGGCAATGACACCGGCAAGCACGGCAAAGAGATTAAGATCACCC
CCCAGTCCTCCATTACCGAAGCTGAACTGACAGGCTACGGCACCGTGACAATGGA
GTGTAGCCCCAGGACCGGACTGGATTTCAACGAGATGGTCCTGCTACAGATGGAG
AACAAGGCCTGGCTCGTGCACAGGCAATGGTTTCTGGATCTGCCTCTGCCTTGGC
TGCCTGGCGCCGATACACAGGGCTCCAACTGGATACAGAAAGAGACCCTCGTCAC
CTTCAAGAATCCCCATGCTAAGAAGCAGGACGTGGTGGTGCTGGGCAGCTACGAA
GCCTGGCTGCACACAGCCCTGACCGGAGCTACCGAGATCCAGATGAGCTCCGGC
AACCTGCTGTTCACCGGCCATCTGAAATGTAGGCTGAGGATGGATAAGCTGCAAC
TCAAAGGCATGTCCTACTCCATGTGCACCGGAAAGTTCAAGGTGGTGAAAGAAATC
GCCGAAACACAGCACGGCACCATCGTGATCAGGGTGCAGTATGAGGGCGACGGC
TCCCCCTGTAAGATCCCCTTCGAAATCATGGACCTGGAAAAGAGGCACGTGCTGG
GCAGGCTCATCACCGTGAACCCCATTGTCACAGAGAAAGACTCCCCCGTGAACAT
CGAGGCCGAGCCTCCCTTTGGCGACTCCTACATCATCATTGGCGTGGAGCCCGGA
CAGCTCAAGCTGAACTGGTTCAAGAAGGGCAGCAGCGGCGGCAGCCATCACCAC
CATCATCACCATCAT

Protein
MKWVTFISLLFLFSSAYSMRCIGMSNRDFVEGVSGGSWVDIVLEHGSCVTTMAKNKPT
LDFELIKTEAKQPATLRKYCIEAKLTNTTTESRCPTQGEPSLNEEQDKRFVCKHSMVDR
GWGNGCGLFGKGGIVTCAMFRCKKNMEGKVVQPENLEYTIVITPHSGEEHAVGNDTG
KHGKEIKITPQSSITEAELTGYGTVTMECSPRTGLDFNEMVLLQMENKAWLVHRQWFL
DLPLPWLPGADTQGSNWIQKETLVTFKNPHAKKQDVVVLGSYEAWLHTALTGATEIQ
MSSGNLLFTGHLKCRLRMDKLQLKGMSYSMCTGKFKVVKEIAETQHGTIVIRVQYEGD
GSPCKIPFEIMDLEKRHVLGRLITVNPIVTEKDSPVNIEAEPPFGDSYIIIGVEPGQLKLN
WFKKGSSGGSHHHHHHHH

pD2sE_EV8_Cm1 - Previously published disulfide (A259C)
Reference: (Rouvinski et. al 2017, Slon Campos et. al., 2017)

DNA
ATGAAGTGGGTAACCTTTATTTCCCTTCTTTTTCTCTTTAGCTCGGCTTATTCCATGA
GGTGTATCGGCATGTCCAACAGGGACTTTGTGGAGGGAGTGAGCGGCGGCAGCT
GGGTGGACATTGTGCTGGAGCATGGAAGCTGCGTGACCACGATGGCGAAAAACAA
GCCCACCCTGGACTTCGAGCTCATCAAGACAGAGGCTAAACAGCCCGCCACCCTC
AGGAAGTACTGCATCGAGGCCAAGCTGACCAACACAACAACCGAGTCCAGATGCC
CTACACAGGGCGAACCCAGCCTCAACGAAGAGCAGGACAAGAGGTTCGTGTGCAA
ACACAGCATGGTGGACAGGGGCTGGGGCAATGGATGCGGACTCTTCGGAAAAGG
CGGCATCGTGACCTGCGCCATGTTCAGGTGTAAAAAGAACATGGAAGGCAAGGTG
GTGCAGCCCGAAAATCTGGAGTATACCATCGTGATTACCCCCCACAGCGGAGAGG
AGCACGCCGTGGGCAATGACACCGGCAAGCACGGCAAAGAGATTAAGATCACCC
CCCAGTCCTCCATTACCGAAGCTGAACTGACAGGCTACGGCACCGTGACAATGGA
GTGTAGCCCCAGGACCGGACTGGATTTCAACGAGATGGTCCTGCTACAGATGGAG
AACAAGGCCTGGCTCGTGCACAGGCAATGGTTTCTGGATCTGCCTCTGCCTTGGC
TGCCTGGCGCCGATACACAGGGCTCCAACTGGATACAGAAAGAGACCCTCGTCAC
CTTCAAGAATCCCCATGCTAAGAAGCAGGACGTGGTGGTGCTGGGCAGCCAAGAA
GGCTGTATGCACACAGCCCTGACCGGAGCTACCGAGATCCAGATGAGCTCCGGC
AACCTGCTGTTCACCGGCCATCTGAAATGTAGGCTGAGGATGGATAAGCTGCAAC
TCAAAGGCATGTCCTACTCCATGTGCACCGGAAAGTTCAAGGTGGTGAAAGAAATC
GCCGAAACACAGCACGGCACCATCGTGATCAGGGTGCAGTATGAGGGCGACGGC
TCCCCCTGTAAGATCCCCTTCGAAATCATGGACCTGGAAAAGAGGCACGTGCTGG
GCAGGCTCATCACCGTGAACCCCATTGTCACAGAGAAAGACTCCCCCGTGAACAT
CGAGGCCGAGCCTCCCTTTGGCGACTCCTACATCATCATTGGCGTGGAGCCCGGA
CAGCTCAAGCTGAACTGGTTCAAGAAGGGCAGCAGCGGCGGCAGCCATCACCAC
CATCATCACCATCAT

Protein
MKWVTFISLLFLFSSAYSMRCIGMSNRDFVEGVSGGSWVDIVLEHGSCVTTMAKNKPT
LDFELIKTEAKQPATLRKYCIEAKLTNTTTESRCPTQGEPSLNEEQDKRFVCKHSMVDR
GWGNGCGLFGKGGIVTCAMFRCKKNMEGKVVQPENLEYTIVITPHSGEEHAVGNDTG
KHGKEIKITPQSSITEAELTGYGTVTMECSPRTGLDFNEMVLLQMENKAWLVHRQWFL
DLPLPWLPGADTQGSNWIQKETLVTFKNPHAKKQDVVVLGSQEGCMHTALTGATEIQ
MSSGNLLFTGHLKCRLRMDKLQLKGMSYSMCTGKFKVVKEIAETQHGTIVIRVQYEGD
GSPCKIPFEIMDLEKRHVLGRLITVNPIVTEKDSPVNIEAEPPFGDSYIIIGVEPGQLKLN
WFKKGSSGGSHHHHHHHH

pD2sE_EV8_Cm2 - Previously published disulfide (L107C/A313C)
Reference: (Rouvinski et. al 2017)

DNA
ATGAAGTGGGTAACCTTTATTTCCCTTCTTTTTCTCTTTAGCTCGGCTTATTCCATGA
GGTGTATCGGCATGTCCAACAGGGACTTTGTGGAGGGAGTGAGCGGCGGCAGCT
GGGTGGACATTGTGCTGGAGCATGGAAGCTGCGTGACCACGATGGCGAAAAACAA
GCCCACCCTGGACTTCGAGCTCATCAAGACAGAGGCTAAACAGCCCGCCACCCTC
AGGAAGTACTGCATCGAGGCCAAGCTGACCAACACAACAACCGAGTCCAGATGCC
CTACACAGGGCGAACCCAGCCTCAACGAAGAGCAGGACAAGAGGTTCGTGTGCAA
ACACAGCATGGTGGACAGGGGCTGGGGCAATGGATGCGGATGTTTCGGAAAAGG
CGGCATCGTGACCTGCGCCATGTTCAGGTGTAAAAAGAACATGGAAGGCAAGGTG
GTGCAGCCCGAAAATCTGGAGTATACCATCGTGATTACCCCCCACAGCGGAGAGG
AGCACGCCGTGGGCAATGACACCGGCAAGCACGGCAAAGAGATTAAGATCACCC
CCCAGTCCTCCATTACCGAAGCTGAACTGACAGGCTACGGCACCGTGACAATGGA
GTGTAGCCCCAGGACCGGACTGGATTTCAACGAGATGGTCCTGCTACAGATGGAG
AACAAGGCCTGGCTCGTGCACAGGCAATGGTTTCTGGATCTGCCTCTGCCTTGGC
TGCCTGGCGCCGATACACAGGGCTCCAACTGGATACAGAAAGAGACCCTCGTCAC
CTTCAAGAATCCCCATGCTAAGAAGCAGGACGTGGTGGTGCTGGGCAGCCAAGAA
GGCGCCATGCACACAGCCCTGACCGGAGCTACCGAGATCCAGATGAGCTCCGGC
AACCTGCTGTTCACCGGCCATCTGAAATGTAGGCTGAGGATGGATAAGCTGCAAC
TCAAAGGCATGTCCTACTCCATGTGCACCGGAAAGTTCAAGGTGGTGAAAGAAATC
TGTGAAACACAGCACGGCACCATCGTGATCAGGGTGCAGTATGAGGGCGACGGC
TCCCCCTGTAAGATCCCCTTCGAAATCATGGACCTGGAAAAGAGGCACGTGCTGG
GCAGGCTCATCACCGTGAACCCCATTGTCACAGAGAAAGACTCCCCCGTGAACAT
CGAGGCCGAGCCTCCCTTTGGCGACTCCTACATCATCATTGGCGTGGAGCCCGGA
CAGCTCAAGCTGAACTGGTTCAAGAAGGGCAGCAGCGGCGGCAGCCATCACCAC
CATCATCACCATCAT

Protein
MKWVTFISLLFLFSSAYSMRCIGMSNRDFVEGVSGGSWVDIVLEHGSCVTTMAKNKPT
LDFELIKTEAKQPATLRKYCIEAKLTNTTTESRCPTQGEPSLNEEQDKRFVCKHSMVDR
GWGNGCGCFGKGGIVTCAMFRCKKNMEGKVVQPENLEYTIVITPHSGEEHAVGNDT
GKHGKEIKITPQSSITEAELTGYGTVTMECSPRTGLDFNEMVLLQMENKAWLVHRQWF
LDLPLPWLPGADTQGSNWIQKETLVTFKNPHAKKQDVVVLGSQEGAMHTALTGATEIQ
MSSGNLLFTGHLKCRLRMDKLQLKGMSYSMCTGKFKVVKEICETQHGTIVIRVQYEGD
GSPCKIPFEIMDLEKRHVLGRLITVNPIVTEKDSPVNIEAEPPFGDSYIIIGVEPGQLKLN
WFKKGSSGGSHHHHHHHH

Rosetta Design Stable Combination (SC) Variants

pD2sE_EV8_SC.1 (I2-I8-P4)

DNA
ATGAAGTGGGTAACCTTTATTTCCCTTCTTTTTCTCTTTAGCTCGGCTTATTCCATGA
GGTGTATCGGCATGTCCAACAGGGACTTTGTGGAGGGAGTGAGCGGCGGCAGCT
GGGTGGACATTGTGCTGGAGCATGGAAAGTGCGTGACCGTGATGATGAAAAACAA
GCCCACCCTGGACTTCGAGCTCATCAAGACAGAGGCTAAACAGCCCGCCACCCTC
AGGAAGTACTGCATCGAGGCCAAGCTGACCAACACAACAACCGAGTCCAGATGCC
CTACACAGGGCGAACCCAGCCTCAACGAAGAGCAGGACAAGAGGTTCGTGTGCAA
ACACAGCATGGTGGACAGGGGCTGGGGCAATGGATGCGACCTCTTCGGAAAAGG
CGGCATCGTGACCTGCGCCATGTTCAGGTGTAAAAAGAACATGGAAGGCAAGGTG
GTGCAGCCCGAAAATCTGGAGTATACCATCGTGATTACCCCCCACAGCGGAGAGG
AGCACGCCGTGGGCAATGACACCGGCAAGCACGGCAAAGAGATTAAGATCACCC
CCCAGTCCTCCATTACCGAAGCTGAACTGACAGGCTACGGCACCGTGACAATGGA
GTGTAGCCCCAGGACCGGACTGGATTTCAACGAGATGGTCCTGCTACAGATGGAG
AACAAGGCCTGGCTCGTGCACAGGCAATGGTTTCTGGATCTGCCTCTGCCTTGGC
TGCCTGGCGCCGATACACAGGGCTCCAACTGGATACAGAAAGAGACCCTCGTCAC
CTTCAAGAATCCCCATGCTAAGAAGCAGGACGTGGTGGTGCTGGGCAGCCAAGAA
GGCTGGATGCACCGGGCCCTGACCGGAGCTACCGAGATCCAGATGAGCTCCGGC
AACCTGCTGTTCACCGGCCATCTGAAATGTAGGCTGAGGATGGATAAGCTGCAAC
TCAAAGGCATGTCCTACTCCATGTGCACCGGAAAGTTCAAGGTGGTGAAAGAAATC
GCCGAAACACAGCACGGCACCATCGTGATCAGGGTGCAGTATGAGGGCGACGGC
TCCCCCTGTAAGATCCCCTTCGAAATCATGGACCTGGAAAAGAGGCACGTGCTGG
GCAGGCTCATCACCGTGAACCCCATTGTCACAGAGAAAGACTCCCCCGTGAACAT
CGAGGCCGAGCCTCCCTTTGGCGACTCCTACATCATCATTGGCGTGGAGCCCGGA
CAGCTCAAGCTGAACTGGTTCAAGAAGGGCAGCAGCGGCGGCAGCCATCACCAC
CATCATCACCATCAT

Protein
MKWVTFISLLFLFSSAYSMRCIGMSNRDFVEGVSGGSWVDIVLEHGKCVTVMMKNKP
TLDFELIKTEAKQPATLRKYCIEAKLTNTTTESRCPTQGEPSLNEEQDKRFVCKHSMVD
RGWGNGCDLFGKGGIVTCAMFRCKKNMEGKVVQPENLEYTIVITPHSGEEHAVGNDT
GKHGKEIKITPQSSITEAELTGYGTVTMECSPRTGLDFNEMVLLQMENKAWLVHRQWF
LDLPLPWLPGADTQGSNWIQKETLVTFKNPHAKKQDVVVLGSQEGWMHRALTGATEI
QMSSGNLLFTGHLKCRLRMDKLQLKGMSYSMCTGKFKVVKEIAETQHGTIVIRVQYEG
DGSPCKIPFEIMDLEKRHVLGRLITVNPIVTEKDSPVNIEAEPPFGDSYIIIGVEPGQLKLN
WFKKGSSGGSHHHHHHHH

pD2sE_EV8_SC.2 (I2-I8-P4-H3)

DNA

ATGAAGTGGGTAACCTTTATTTCCCTTCTTTTTCTCTTTAGCTCGGCTTATTCCATGA
GGTGTATCGGCATGTCCAACAGGGACTTTGTGGAGGGAGTGAGCGGCGGCAGCT
GGGTGGACATTGTGCTGGAGCCCGGAAAGTGCGTGACCGTGATGATGAAAAACAA
GCCCACCCTGGACTTCGAGCTCATCAAGATCGAGGCTAAACAGCCCGCCACCCTC
AGGAAGTACTGCATCGAGGCCAAGCTGACCAACACAACAACCGAGTCCAGATGCC
CTACACAGGGCGAACCCAGCCTCAACGAAGAGCAGGACAAGAGGTTCGTGTGCAA
ACACAGCATGGTGGACAGGGGCTGGGGCAATGGATGCGACCTCTTCGGAAAAGG
CGGCATCGTGACCTGCGCCATGTTCAGGTGTAAAAAGAACATGGAAGGCAAGGTG
GTGCAGCCCGAAAATCTGGAGTATACCATCGTGATTACCCCCCACAGCGGAGAGG
AGCACGCCGTGGGCAATGACACCGGCAAGCACGGCAAAGAGATTAAGATCACCC
CCCAGTCCTCCATTACCGAAGCTGAACTGACAGGCTACGGCACCGTGACAATGGA
GTGTAGCCCCAGGACCGGACTGGATTTCAACGAGATGGTCCTGCTACAGATGGAG
AACAAGGCCTGGCTCGTGCACAGGCAATGGTTTCTGGATCTGCCTCTGCCTTGGC
TGCCTGGCGCCGATACACAGGGCTCCAACTGGATACAGAAAGAGACCCTCGTCAC
CTTCAAGAATCCCCATGCTAAGAAGCAGGACGTGGTGGTGCTGGGCAGCCAAGAA
GGCTGGATGCACCGGGCCCTGACCGGAGCTACCGAGATCCAGATGAGCTCCGGC
AACCTGCTGTTCACCGGCCATCTGAAATGTAGGCTGAGGATGGATAAGCTGCAAC
TCAAAGGCATGTCCTACTCCATGTGCACCGGAAAGTTCAAGGTGGTGAAAGAAATC
GCCGAAACACAGCACGGCACCATCGTGATCAGGGTGCAGTATGAGGGCGACGGC
TCCCCCTGTAAGATCCCCTTCGAAATCATGGACCTGGAAAAGAGGCACGTGCTGG
GCAGGCTCATCACCGTGAACCCCATTGTCACAGAGAAAGACTCCCCCGTGAACAT
CGAGGCCGAGCCTCCCTTTGGCGACTCCTACATCATCATTGGCGTGGAGCCCGGA
CAGCTCAAGCTGAACTGGTTCAAGAAGGGCAGCAGCGGCGGCAGCCATCACCAC
CATCATCACCATCAT

Protein

MKWVTFISLLFLFSSAYSMRCIGMSNRDFVEGVSGGSWVDIVLEPGKCVTVMMKNKP
TLDFELIKIEAKQPATLRKYCIEAKLTNTTTESRCPTQGEPSLNEEQDKRFVCKHSMVD
RGWGNGCDLFGKGGIVTCAMFRCKKNMEGKVVQPENLEYTIVITPHSGEEHAVGNDT
GKHGKEIKITPQSSITEAELTGYGTVTMECSPRTGLDFNEMVLLQMENKAWLVHRQWF
LDLPLPWLPGADTQGSNWIQKETLVTFKNPHAKKQDVVVLGSQEGWMHRALTGATEI
QMSSGNLLFTGHLKCRLRMDKLQLKGMSYSMCTGKFKVVKEIAETQHGTIVIRVQYEG
DGSPCKIPFEIMDLEKRHVLGRLITVNPIVTEKDSPVNIEAEPPFGDSYIIIGVEPGQLKLN
WFKKGSSGGSHHHHHHHH

pD2sE_EV8_SC.3 (I2-I8-U4-U5-U6-P5)

DNA
ATGAAGTGGGTAACCTTTATTTCCCTTCTTTTTCTCTTTAGCTCGGCTTATTCCATGA
GGTGTATCGGCATGTCCAACAGGGACTTTGTGTTCGCCGTGAGCGGCGGCAGCTG
GGTGGACATTGTGCTGGAGCATGGAAGCTGCGTGACCACGCTGGCGAAAAACAA
GCCCACCCTGGACTTCGAGCTCATCAAGACAGAGGCTAAACAGCCCGCCACCCTC
AGGAAGTACTGCATCGAGGCCAAGCTGACCAACACAACAACCGAGTCCAGATGCC
CTACACAGGGCGAACCCAGCCTCAACGAAGAGCAGGACAAGAGGTTCGTGTGCAA
ACACAGCATGGTGGACAGGGGCTGGGGCAATGGATGCGACCTCTTCGGAAAAGG
CGGCATCGTGACCTGCGCCATGTTCAGGTGTAAAAAGAACATGGAAGGCAAGGTG
GTGCAGCCCGAAAATCTGGAGTATACCATCGTGATTACCCCCCACAGCGGAGAGG
AGCACGCCGTGGGCAATGACACCGGCAAGCACGGCAAAGAGATTAAGATCACCC
CCCAGTCCTCCATTACCGAAGCTGAACTGACAGGCTACGGCACCGTGACAATGGA
GTGTAGCCCCAGGACCGGACTGGATTTCAACGAGATGGTCCTGCTACAGATGGAG
AACAAGGCCTGGCTCGTGGACAGGCAATGGTTTCTGGATCTGCCTCTGCCTTGGC
TGCCTGGCGCCGATACACAGGGCTCCAACTGGATACAGAAAGAGACCCTCGTCAC
CTTCAAGAATCCCCATGCTAAGAAGCAGGACGTGGTGGTGCTGGGCAGCCAAGAA
GGCTGGATGCACCGGGCCCTGACCTGGGCTACCGAGATCCAGATGAGCTCCGGC
AACCTGCTGTGGCCCGGCCATCTGAAATGTAGGCTGAGGATGGATAAGCTGCAAC
TCAAAGGCATGTCCTACTCCATGTGCACCGGAAAGTTCAAGGTGGTGAAAGAAATC
GCCGAAACACAGCACGGCACCATCGTGATCAGGGTGCAGTATGAGGGCGACGGC
TCCCCCTGTAAGATCCCCTTCGAAATCATGGACCTGGAAAAGAGGCACGTGCTGG
GCAGGCTCATCACCGTGAACCCCATTGTCTACGAGAAAGACTCCCCCGTGAACAT
CGAGGCCGAGCCTCCCTTTGGCGACTCCTACATCATCATTGGCGTGGAGCCCGGA
CAGCTCAAGCTGAACTGGTTCAAGAAGGGCAGCAGCGGCGGCAGCCATCACCAC
CATCATCACCATCAT

Protein
MKWVTFISLLFLFSSAYSMRCIGMSNRDFVFAVSGGSWVDIVLEHGSCVTTLAKNKPT
LDFELIKTEAKQPATLRKYCIEAKLTNTTTESRCPTQGEPSLNEEQDKRFVCKHSMVDR
GWGNGCDLFGKGGIVTCAMFRCKKNMEGKVVQPENLEYTIVITPHSGEEHAVGNDTG
KHGKEIKITPQSSITEAELTGYGTVTMECSPRTGLDFNEMVLLQMENKAWLVDRQWFL
DLPLPWLPGADTQGSNWIQKETLVTFKNPHAKKQDVVVLGSQEGWMHRALTWATEIQ
MSSGNLLWPGHLKCRLRMDKLQLKGMSYSMCTGKFKVVKEIAETQHGTIVIRVQYEG
DGSPCKIPFEIMDLEKRHVLGRLITVNPIVYEKDSPVNIEAEPPFGDSYIIIGVEPGQLKLN
WFKKGSSGGSHHHHHHHH

pD2sE_EV8_SC.4 (I2-I8-U4-U5-U6-P4-P5-H3)

DNA
ATGAAGTGGGTAACCTTTATTTCCCTTCTTTTTCTCTTTAGCTCGGCTTATTCCATGA
GGTGTATCGGCATGTCCAACAGGGACTTTGTGTTCGCCGTGAGCGGCGGCAGCTG
GGTGGACATTGTGCTGGAGCCCGGAAAGTGCGTGACCGTGCTGATGAAAAACAAG
CCCACCCTGGACTTCGAGCTCATCAAGATCGAGGCTAAACAGCCCGCCACCCTCA
GGAAGTACTGCATCGAGGCCAAGCTGACCAACACAACAACCGAGTCCAGATGCCC
TACACAGGGCGAACCCAGCCTCAACGAAGAGCAGGACAAGAGGTTCGTGTGCAAA
CACAGCATGGTGGACAGGGGCTGGGGCAATGGATGCGACCTCTTCGGAAAAGGC
GGCATCGTGACCTGCGCCATGTTCAGGTGTAAAAAGAACATGGAAGGCAAGGTGG
TGCAGCCCGAAAATCTGGAGTATACCATCGTGATTACCCCCCACAGCGGAGAGGA
GCACGCCGTGGGCAATGACACCGGCAAGCACGGCAAAGAGATTAAGATCACCCC
CCAGTCCTCCATTACCGAAGCTGAACTGACAGGCTACGGCACCGTGACAATGGAG
TGTAGCCCCAGGACCGGACTGGATTTCAACGAGATGGTCCTGCTACAGATGGAGA
ACAAGGCCTGGCTCGTGGACAGGCAATGGTTTCTGGATCTGCCTCTGCCTTGGCT
GCCTGGCGCCGATACACAGGGCTCCAACTGGATACAGAAAGAGACCCTCGTCACC
TTCAAGAATCCCCATGCTAAGAAGCAGGACGTGGTGGTGCTGGGCAGCCAAGAAG
GCTGGATGCACCGGGCCCTGACCTGGGCTACCGAGATCCAGATGAGCTCCGGCA
ACCTGCTGTGGCCCGGCCATCTGAAATGTAGGCTGAGGATGGATAAGCTGCAACT
CAAAGGCATGTCCTACTCCATGTGCACCGGAAAGTTCAAGGTGGTGAAAGAAATC
GCCGAAACACAGCACGGCACCATCGTGATCAGGGTGCAGTATGAGGGCGACGGC
TCCCCCTGTAAGATCCCCTTCGAAATCATGGACCTGGAAAAGAGGCACGTGCTGG
GCAGGCTCATCACCGTGAACCCCATTGTCTACGAGAAAGACTCCCCCGTGAACAT
CGAGGCCGAGCCTCCCTTTGGCGACTCCTACATCATCATTGGCGTGGAGCCCGGA
CAGCTCAAGCTGAACTGGTTCAAGAAGGGCAGCAGCGGCGGCAGCCATCACCAC
CATCATCACCATCAT

Protein
MKWVTFISLLFLFSSAYSMRCIGMSNRDFVFAVSGGSWVDIVLEPGKCVTVLMKNKPT
LDFELIKIEAKQPATLRKYCIEAKLTNTTTESRCPTQGEPSLNEEQDKRFVCKHSMVDR
GWGNGCDLFGKGGIVTCAMFRCKKNMEGKVVQPENLEYTIVITPHSGEEHAVGNDTG
KHGKEIKITPQSSITEAELTGYGTVTMECSPRTGLDFNEMVLLQMENKAWLVDRQWFL
DLPLPWLPGADTQGSNWIQKETLVTFKNPHAKKQDVVVLGSQEGWMHRALTWATEIQ
MSSGNLLWPGHLKCRLRMDKLQLKGMSYSMCTGKFKVVKEIAETQHGTIVIRVQYEG
DGSPCKIPFEIMDLEKRHVLGRLITVNPIVYEKDSPVNIEAEPPFGDSYIIIGVEPGQLKLN
WFKKGSSGGSHHHHHHHH

pD2sE_EV8_SC.5 (I2-I8-U4-U6)

DNA
ATGAAGTGGGTAACCTTTATTTCCCTTCTTTTTCTCTTTAGCTCGGCTTATTCCATGA
GGTGTATCGGCATGTCCAACAGGGACTTTGTGTTCGCCGTGAGCGGCGGCAGCTG
GGTGGACATTGTGCTGGAGCATGGAAGCTGCGTGACCACGCTGGCGAAAAACAA
GCCCACCCTGGACTTCGAGCTCATCAAGACAGAGGCTAAACAGCCCGCCACCCTC
AGGAAGTACTGCATCGAGGCCAAGCTGACCAACACAACAACCGAGTCCAGATGCC
CTACACAGGGCGAACCCAGCCTCAACGAAGAGCAGGACAAGAGGTTCGTGTGCAA
ACACAGCATGGTGGACAGGGGCTGGGGCAATGGATGCGACCTCTTCGGAAAAGG
CGGCATCGTGACCTGCGCCATGTTCAGGTGTAAAAAGAACATGGAAGGCAAGGTG
GTGCAGCCCGAAAATCTGGAGTATACCATCGTGATTACCCCCCACAGCGGAGAGG
AGCACGCCGTGGGCAATGACACCGGCAAGCACGGCAAAGAGATTAAGATCACCC
CCCAGTCCTCCATTACCGAAGCTGAACTGACAGGCTACGGCACCGTGACAATGGA
GTGTAGCCCCAGGACCGGACTGGATTTCAACGAGATGGTCCTGCTACAGATGGAG
AACAAGGCCTGGCTCGTGCACAGGCAATGGTTTCTGGATCTGCCTCTGCCTTGGC
TGCCTGGCGCCGATACACAGGGCTCCAACTGGATACAGAAAGAGACCCTCGTCAC
CTTCAAGAATCCCCATGCTAAGAAGCAGGACGTGGTGGTGCTGGGCAGCCAAGAA
GGCTGGATGCACCGGGCCCTGACCGGAGCTACCGAGATCCAGATGAGCTCCGGC
AACCTGCTGTGGCCCGGCCATCTGAAATGTAGGCTGAGGATGGATAAGCTGCAAC
TCAAAGGCATGTCCTACTCCATGTGCACCGGAAAGTTCAAGGTGGTGAAAGAAATC
GCCGAAACACAGCACGGCACCATCGTGATCAGGGTGCAGTATGAGGGCGACGGC
TCCCCCTGTAAGATCCCCTTCGAAATCATGGACCTGGAAAAGAGGCACGTGCTGG
GCAGGCTCATCACCGTGAACCCCATTGTCACAGAGAAAGACTCCCCCGTGAACAT
CGAGGCCGAGCCTCCCTTTGGCGACTCCTACATCATCATTGGCGTGGAGCCCGGA
CAGCTCAAGCTGAACTGGTTCAAGAAGGGCAGCAGCGGCGGCAGCCATCACCAC
CATCATCACCATCAT

Protein
MKWVTFISLLFLFSSAYSMRCIGMSNRDFVFAVSGGSWVDIVLEHGSCVTTLAKNKPT
LDFELIKTEAKQPATLRKYCIEAKLTNTTTESRCPTQGEPSLNEEQDKRFVCKHSMVDR
GWGNGCDLFGKGGIVTCAMFRCKKNMEGKVVQPENLEYTIVITPHSGEEHAVGNDTG
KHGKEIKITPQSSITEAELTGYGTVTMECSPRTGLDFNEMVLLQMENKAWLVHRQWFL
DLPLPWLPGADTQGSNWIQKETLVTFKNPHAKKQDVVVLGSQEGWMHRALTGATEIQ
MSSGNLLWPGHLKCRLRMDKLQLKGMSYSMCTGKFKVVKEIAETQHGTIVIRVQYEG
DGSPCKIPFEIMDLEKRHVLGRLITVNPIVTEKDSPVNIEAEPPFGDSYIIIGVEPGQLKLN
WFKKGSSGGSHHHHHHHH

pD2sE_EV8_SC.6 (I2-I8-U5-P4)

DNA

ATGAAGTGGGTAACCTTTATTTCCCTTCTTTTTCTCTTTAGCTCGGCTTATTCCATGA
GGTGTATCGGCATGTCCAACAGGGACTTTGTGGAGGGAGTGAGCGGCGGCAGCT
GGGTGGACATTGTGCTGGAGCATGGAAAGTGCGTGACCGTGATGATGAAAAACAA
GCCCACCCTGGACTTCGAGCTCATCAAGACAGAGGCTAAACAGCCCGCCACCCTC
AGGAAGTACTGCATCGAGGCCAAGCTGACCAACACAACAACCGAGTCCAGATGCC
CTACACAGGGCGAACCCAGCCTCAACGAAGAGCAGGACAAGAGGTTCGTGTGCAA
ACACAGCATGGTGGACAGGGGCTGGGGCAATGGATGCGACCTCTTCGGAAAAGG
CGGCATCGTGACCTGCGCCATGTTCAGGTGTAAAAAGAACATGGAAGGCAAGGTG
GTGCAGCCCGAAAATCTGGAGTATACCATCGTGATTACCCCCCACAGCGGAGAGG
AGCACGCCGTGGGCAATGACACCGGCAAGCACGGCAAAGAGATTAAGATCACCC
CCCAGTCCTCCATTACCGAAGCTGAACTGACAGGCTACGGCACCGTGACAATGGA
GTGTAGCCCCAGGACCGGACTGGATTTCAACGAGATGGTCCTGCTACAGATGGAG
AACAAGGCCTGGCTCGTGCACAGGCAATGGTTTCTGGATCTGCCTCTGCCTTGGC
TGCCTGGCGCCGATACACAGGGCTCCAACTGGATACAGAAAGAGACCCTCGTCAC
CTTCAAGAATCCCCATGCTAAGAAGCAGGACGTGGTGGTGCTGGGCAGCCAAGAA
GGCTGGATGCACCGGGCCCTGACCGGAGCTACCGAGATCCAGATGAGCTCCGGC
AACCTGCTGTTCACCGGCCATCTGAAATGTAGGCTGAGGATGGATAAGCTGCAAC
TCAAAGGCATGTCCTACTCCATGTGCACCGGAAAGTTCAAGGTGGTGAAAGAAATC
GCCGAAACACAGCACGGCACCATCGTGATCAGGGTGCAGTATGAGGGCGACGGC
TCCCCCTGTAAGATCCCCTTCGAAATCATGGACCTGGAAAAGAGGCACGTGCTGG
GCAGGCTCATCACCGTGAACCCCATTGTCTACGAGAAAGACTCCCCCGTGAACAT
CGAGGCCGAGCCTCCCTTTGGCGACTCCTACATCATCATTGGCGTGGAGCCCGGA
CAGCTCAAGCTGAACTGGTTCAAGAAGGGCAGCAGCGGCGGCAGCCATCACCAC
CATCATCACCATCAT

Protein

MKWVTFISLLFLFSSAYSMRCIGMSNRDFVEGVSGGSWVDIVLEHGKCVTVMMKNKP
TLDFELIKTEAKQPATLRKYCIEAKLTNTTTESRCPTQGEPSLNEEQDKRFVCKHSMVD
RGWGNGCDLFGKGGIVTCAMFRCKKNMEGKVVQPENLEYTIVITPHSGEEHAVGNDT
GKHGKEIKITPQSSITEAELTGYGTVTMECSPRTGLDFNEMVLLQMENKAWLVHRQWF
LDLPLPWLPGADTQGSNWIQKETLVTFKNPHAKKQDVVVLGSQEGWMHRALTGATEI
QMSSGNLLFTGHLKCRLRMDKLQLKGMSYSMCTGKFKVVKEIAETQHGTIVIRVQYEG
DGSPCKIPFEIMDLEKRHVLGRLITVNPIVYEKDSPVNIEAEPPFGDSYIIIGVEPGQLKLN
WFKKGSSGGSHHHHHHHH

pD2sE_EV8_SC.7 (I2-I8-U5-U6)

DNA
ATGAAGTGGGTAACCTTTATTTCCCTTCTTTTTCTCTTTAGCTCGGCTTATTCCATGA
GGTGTATCGGCATGTCCAACAGGGACTTTGTGGAGGGAGTGAGCGGCGGCAGCT
GGGTGGACATTGTGCTGGAGCATGGAAGCTGCGTGACCACGATGGCGAAAAACAA
GCCCACCCTGGACTTCGAGCTCATCAAGACAGAGGCTAAACAGCCCGCCACCCTC
AGGAAGTACTGCATCGAGGCCAAGCTGACCAACACAACAACCGAGTCCAGATGCC
CTACACAGGGCGAACCCAGCCTCAACGAAGAGCAGGACAAGAGGTTCGTGTGCAA
ACACAGCATGGTGGACAGGGGCTGGGGCAATGGATGCGACCTCTTCGGAAAAGG
CGGCATCGTGACCTGCGCCATGTTCAGGTGTAAAAAGAACATGGAAGGCAAGGTG
GTGCAGCCCGAAAATCTGGAGTATACCATCGTGATTACCCCCCACAGCGGAGAGG
AGCACGCCGTGGGCAATGACACCGGCAAGCACGGCAAAGAGATTAAGATCACCC
CCCAGTCCTCCATTACCGAAGCTGAACTGACAGGCTACGGCACCGTGACAATGGA
GTGTAGCCCCAGGACCGGACTGGATTTCAACGAGATGGTCCTGCTACAGATGGAG
AACAAGGCCTGGCTCGTGCACAGGCAATGGTTTCTGGATCTGCCTCTGCCTTGGC
TGCCTGGCGCCGATACACAGGGCTCCAACTGGATACAGAAAGAGACCCTCGTCAC
CTTCAAGAATCCCCATGCTAAGAAGCAGGACGTGGTGGTGCTGGGCAGCCAAGAA
GGCTGGATGCACCGGGCCCTGACCGGAGCTACCGAGATCCAGATGAGCTCCGGC
AACCTGCTGTGGCCCGGCCATCTGAAATGTAGGCTGAGGATGGATAAGCTGCAAC
TCAAAGGCATGTCCTACTCCATGTGCACCGGAAAGTTCAAGGTGGTGAAAGAAATC
GCCGAAACACAGCACGGCACCATCGTGATCAGGGTGCAGTATGAGGGCGACGGC
TCCCCCTGTAAGATCCCCTTCGAAATCATGGACCTGGAAAAGAGGCACGTGCTGG
GCAGGCTCATCACCGTGAACCCCATTGTCTACGAGAAAGACTCCCCCGTGAACAT
CGAGGCCGAGCCTCCCTTTGGCGACTCCTACATCATCATTGGCGTGGAGCCCGGA
CAGCTCAAGCTGAACTGGTTCAAGAAGGGCAGCAGCGGCGGCAGCCATCACCAC
CATCATCACCATCATTAGTAA

Protein
MKWVTFISLLFLFSSAYSMRCIGMSNRDFVEGVSGGSWVDIVLEHGSCVTTMAKNKPT
LDFELIKTEAKQPATLRKYCIEAKLTNTTTESRCPTQGEPSLNEEQDKRFVCKHSMVDR
GWGNGCDLFGKGGIVTCAMFRCKKNMEGKVVQPENLEYTIVITPHSGEEHAVGNDTG
KHGKEIKITPQSSITEAELTGYGTVTMECSPRTGLDFNEMVLLQMENKAWLVHRQWFL
DLPLPWLPGADTQGSNWIQKETLVTFKNPHAKKQDVVVLGSQEGWMHRALTGATEIQ
MSSGNLLWPGHLKCRLRMDKLQLKGMSYSMCTGKFKVVKEIAETQHGTIVIRVQYEG
DGSPCKIPFEIMDLEKRHVLGRLITVNPIVYEKDSPVNIEAEPPFGDSYIIIGVEPGQLKLN
WFKKGSSGGSHHHHHHHH

pD2sE_EV8_SC.8 (I2-I8-U5-U6-P4)

DNA
ATGAAGTGGGTAACCTTTATTTCCCTTCTTTTTCTCTTTAGCTCGGCTTATTCCATGA
GGTGTATCGGCATGTCCAACAGGGACTTTGTGGAGGGAGTGAGCGGCGGCAGCT
GGGTGGACATTGTGCTGGAGCATGGAAAGTGCGTGACCGTGATGATGAAAAACAA
GCCCACCCTGGACTTCGAGCTCATCAAGACAGAGGCTAAACAGCCCGCCACCCTC
AGGAAGTACTGCATCGAGGCCAAGCTGACCAACACAACAACCGAGTCCAGATGCC
CTACACAGGGCGAACCCAGCCTCAACGAAGAGCAGGACAAGAGGTTCGTGTGCAA
ACACAGCATGGTGGACAGGGGCTGGGGCAATGGATGCGACCTCTTCGGAAAAGG
CGGCATCGTGACCTGCGCCATGTTCAGGTGTAAAAAGAACATGGAAGGCAAGGTG
GTGCAGCCCGAAAATCTGGAGTATACCATCGTGATTACCCCCCACAGCGGAGAGG
AGCACGCCGTGGGCAATGACACCGGCAAGCACGGCAAAGAGATTAAGATCACCC
CCCAGTCCTCCATTACCGAAGCTGAACTGACAGGCTACGGCACCGTGACAATGGA
GTGTAGCCCCAGGACCGGACTGGATTTCAACGAGATGGTCCTGCTACAGATGGAG
AACAAGGCCTGGCTCGTGCACAGGCAATGGTTTCTGGATCTGCCTCTGCCTTGGC
TGCCTGGCGCCGATACACAGGGCTCCAACTGGATACAGAAAGAGACCCTCGTCAC
CTTCAAGAATCCCCATGCTAAGAAGCAGGACGTGGTGGTGCTGGGCAGCCAAGAA
GGCTGGATGCACCGGGCCCTGACCGGAGCTACCGAGATCCAGATGAGCTCCGGC
AACCTGCTGTGGCCCGGCCATCTGAAATGTAGGCTGAGGATGGATAAGCTGCAAC
TCAAAGGCATGTCCTACTCCATGTGCACCGGAAAGTTCAAGGTGGTGAAAGAAATC
GCCGAAACACAGCACGGCACCATCGTGATCAGGGTGCAGTATGAGGGCGACGGC
TCCCCCTGTAAGATCCCCTTCGAAATCATGGACCTGGAAAAGAGGCACGTGCTGG
GCAGGCTCATCACCGTGAACCCCATTGTCTACGAGAAAGACTCCCCCGTGAACAT
CGAGGCCGAGCCTCCCTTTGGCGACTCCTACATCATCATTGGCGTGGAGCCCGGA
CAGCTCAAGCTGAACTGGTTCAAGAAGGGCAGCAGCGGCGGCAGCCATCACCAC
CATCATCACCATCAT

Protein
MKWVTFISLLFLFSSAYSMRCIGMSNRDFVEGVSGGSWVDIVLEHGKCVTVMMKNKP
TLDFELIKTEAKQPATLRKYCIEAKLTNTTTESRCPTQGEPSLNEEQDKRFVCKHSMVD
RGWGNGCDLFGKGGIVTCAMFRCKKNMEGKVVQPENLEYTIVITPHSGEEHAVGNDT
GKHGKEIKITPQSSITEAELTGYGTVTMECSPRTGLDFNEMVLLQMENKAWLVHRQWF
LDLPLPWLPGADTQGSNWIQKETLVTFKNPHAKKQDVVVLGSQEGWMHRALTGATEI
QMSSGNLLWPGHLKCRLRMDKLQLKGMSYSMCTGKFKVVKEIAETQHGTIVIRVQYE
GDGSPCKIPFEIMDLEKRHVLGRLITVNPIVYEKDSPVNIEAEPPFGDSYIIIGVEPGQLK
LNWFKKGSSGGSHHHHHHHH

pD2sE_EV8_SC.9 (I2-I8-U5-U6-P4-S1)

DNA
ATGAAGTGGGTAACCTTTATTTCCCTTCTTTTTCTCTTTAGCTCGGCTTATTCCATGA
GGTGTATCGGCATGTCCAACAGGGACTTTGTGGAGGGAGAGAGCGGCGGCAGCT
GGGTGGACATTGTGCTGGAGCATGGAAAGTGCGTGACCGTGATGATGAAAAACAA
GCCCACCCTGGACTTCGAGCTCATCAAGACAGAGGCTAAACAGCCCGCCACCCTC
AGGAAGTACTGCATCGAGGCCAAGCTGACCAACACAACAACCGAGTCCAGATGCC
CTACACAGGGCGAACCCAGCCTCAACGAAGAGCAGGACAAGAGGTTCGTGTGCAA
ACACAGCATGGTGGACAGGGGCTGGGGCAATGGATGCGACCTCTTCGGAAAAGG
CGGCATCGTGACCTGCGCCATGTTCAGGTGTAAAAAGAACATGGAAGGCAAGGTG
GTGCAGCCCGAAAATCTGGAGTATACCATCGTGATTACCCCCCACAGCGGAGAGG
AGCACGCCGTGGGCAATGACACCGGCAAGCACGGCAAAGAGATTAAGATCACCC
CCCAGTCCTCCATTACCGAAGCTGAACTGACAGGCTACGGCACCGTGACAATGGA
GTGTAGCCCCAGGACCGGACTGGATTTCAACGAGATGGTCCTGCTACAGATGGAG
AACAAGGCCTGGCTCGTGCACAGGCAATGGTTTCTGGATCTGCCTCTGCCTTGGC
TGCCTGGCGCCGATACACAGGGCTCCAACTGGATACAGAAAGAGACCCTCGTCAC
CTTCAAGAATCCCCATGCTAAGAAGCAGGACGTGGTGGTGCTGGGCAGCCAAGAA
GGCTGGATGCACCGGGCCCTGACCGGAGCTACCGAGATCCAGATGAGCTCCGGC
AACCTGCTGTGGCCCGGCCATCTGAAATGTAGGCTGAGGATGGATAAGCTGCAAC
TCAAAGGCATGTCCTACTCCATGTGCACCGGAAAGTTCAAGGTGGTGAAAGAAATC
GCCGAAACACAGCACGGCACCATCGTGATCAGGGTGCAGTATGAGGGCGACGGC
TCCCCCTGTAAGATCCCCTTCGAAATCATGGACCTGGAAAAGAGGCACGTGCTGG
GCAGGCTCATCACCGTGAACCCCATTGTCTACGAGAAAGACTCCCCCGTGAACAT
CGAGGCCGAGCCTCCCGACGGCGACTCCTACATCATCATTGGCGTGGAGCCCGG
ACAGCTCAAGCTGAACTGGAGAAAGAAGGGCAGCAGCGGCGGCAGCCATCACCA
CCATCATCACCATCAT

Protein
MKWVTFISLLFLFSSAYSMRCIGMSNRDFVEGESGGSWVDIVLEHGKCVTVMMKNKP
TLDFELIKTEAKQPATLRKYCIEAKLTNTTTESRCPTQGEPSLNEEQDKRFVCKHSMVD
RGWGNGCDLFGKGGIVTCAMFRCKKNMEGKVVQPENLEYTIVITPHSGEEHAVGNDT
GKHGKEIKITPQSSITEAELTGYGTVTMECSPRTGLDFNEMVLLQMENKAWLVHRQWF
LDLPLPWLPGADTQGSNWIQKETLVTFKNPHAKKQDVVVLGSQEGWMHRALTGATEI
QMSSGNLLWPGHLKCRLRMDKLQLKGMSYSMCTGKFKVVKEIAETQHGTIVIRVQYE
GDGSPCKIPFEIMDLEKRHVLGRLITVNPIVYEKDSPVNIEAEPPDGDSYIIIGVEPGQLK
LNWRKKGSSGGSHHHHHHHH

pD2sE_EV8_SC.10 (I2-I8-U6)

DNA
ATGAAGTGGGTAACCTTTATTTCCCTTCTTTTTCTCTTTAGCTCGGCTTATTCCATGA
GGTGTATCGGCATGTCCAACAGGGACTTTGTGGAGGGAGTGAGCGGCGGCAGCT
GGGTGGACATTGTGCTGGAGCATGGAAGCTGCGTGACCACGATGGCGAAAAACAA
GCCCACCCTGGACTTCGAGCTCATCAAGACAGAGGCTAAACAGCCCGCCACCCTC
AGGAAGTACTGCATCGAGGCCAAGCTGACCAACACAACAACCGAGTCCAGATGCC
CTACACAGGGCGAACCCAGCCTCAACGAAGAGCAGGACAAGAGGTTCGTGTGCAA
ACACAGCATGGTGGACAGGGGCTGGGGCAATGGATGCGACCTCTTCGGAAAAGG
CGGCATCGTGACCTGCGCCATGTTCAGGTGTAAAAAGAACATGGAAGGCAAGGTG
GTGCAGCCCGAAAATCTGGAGTATACCATCGTGATTACCCCCCACAGCGGAGAGG
AGCACGCCGTGGGCAATGACACCGGCAAGCACGGCAAAGAGATTAAGATCACCC
CCCAGTCCTCCATTACCGAAGCTGAACTGACAGGCTACGGCACCGTGACAATGGA
GTGTAGCCCCAGGACCGGACTGGATTTCAACGAGATGGTCCTGCTACAGATGGAG
AACAAGGCCTGGCTCGTGCACAGGCAATGGTTTCTGGATCTGCCTCTGCCTTGGC
TGCCTGGCGCCGATACACAGGGCTCCAACTGGATACAGAAAGAGACCCTCGTCAC
CTTCAAGAATCCCCATGCTAAGAAGCAGGACGTGGTGGTGCTGGGCAGCCAAGAA
GGCTGGATGCACCGGGCCCTGACCGGAGCTACCGAGATCCAGATGAGCTCCGGC
AACCTGCTGTGGCCCGGCCATCTGAAATGTAGGCTGAGGATGGATAAGCTGCAAC
TCAAAGGCATGTCCTACTCCATGTGCACCGGAAAGTTCAAGGTGGTGAAAGAAATC
GCCGAAACACAGCACGGCACCATCGTGATCAGGGTGCAGTATGAGGGCGACGGC
TCCCCCTGTAAGATCCCCTTCGAAATCATGGACCTGGAAAAGAGGCACGTGCTGG
GCAGGCTCATCACCGTGAACCCCATTGTCACAGAGAAAGACTCCCCCGTGAACAT
CGAGGCCGAGCCTCCCTTTGGCGACTCCTACATCATCATTGGCGTGGAGCCCGGA
CAGCTCAAGCTGAACTGGTTCAAGAAGGGCAGCAGCGGCGGCAGCCATCACCAC
CATCATCACCATCAT

Protein
MKWVTFISLLFLFSSAYSMRCIGMSNRDFVEGVSGGSWVDIVLEHGSCVTTMAKNKPT
LDFELIKTEAKQPATLRKYCIEAKLTNTTTESRCPTQGEPSLNEEQDKRFVCKHSMVDR
GWGNGCDLFGKGGIVTCAMFRCKKNMEGKVVQPENLEYTIVITPHSGEEHAVGNDTG
KHGKEIKITPQSSITEAELTGYGTVTMECSPRTGLDFNEMVLLQMENKAWLVHRQWFL
DLPLPWLPGADTQGSNWIQKETLVTFKNPHAKKQDVVVLGSQEGWMHRALTGATEIQ
MSSGNLLWPGHLKCRLRMDKLQLKGMSYSMCTGKFKVVKEIAETQHGTIVIRVQYEG
DGSPCKIPFEIMDLEKRHVLGRLITVNPIVTEKDSPVNIEAEPPFGDSYIIIGVEPGQLKLN
WFKKGSSGGSHHHHHHHH

pD2sE_EV8_SC.11 (I2-I8-U6-H3)

DNA
ATGAAGTGGGTAACCTTTATTTCCCTTCTTTTTCTCTTTAGCTCGGCTTATTCCATGA
GGTGTATCGGCATGTCCAACAGGGACTTTGTGGAGGGAGTGAGCGGCGGCAGCT
GGGTGGACATTGTGCTGGAGCCCGGAAGCTGCGTGACCACGATGGCGAAAAACA
AGCCCACCCTGGACTTCGAGCTCATCAAGATCGAGGCTAAACAGCCCGCCACCCT
CAGGAAGTACTGCATCGAGGCCAAGCTGACCAACACAACAACCGAGTCCAGATGC
CCTACACAGGGCGAACCCAGCCTCAACGAAGAGCAGGACAAGAGGTTCGTGTGC
AAACACAGCATGGTGGACAGGGGCTGGGGCAATGGATGCGACCTCTTCGGAAAA
GGCGGCATCGTGACCTGCGCCATGTTCAGGTGTAAAAAGAACATGGAAGGCAAGG
TGGTGCAGCCCGAAAATCTGGAGTATACCATCGTGATTACCCCCCACAGCGGAGA
GGAGCACGCCGTGGGCAATGACACCGGCAAGCACGGCAAAGAGATTAAGATCAC
CCCCCAGTCCTCCATTACCGAAGCTGAACTGACAGGCTACGGCACCGTGACAATG
GAGTGTAGCCCCAGGACCGGACTGGATTTCAACGAGATGGTCCTGCTACAGATGG
AGAACAAGGCCTGGCTCGTGCACAGGCAATGGTTTCTGGATCTGCCTCTGCCTTG
GCTGCCTGGCGCCGATACACAGGGCTCCAACTGGATACAGAAAGAGACCCTCGTC
ACCTTCAAGAATCCCCATGCTAAGAAGCAGGACGTGGTGGTGCTGGGCAGCCAAG
AAGGCTGGATGCACCGGGCCCTGACCGGAGCTACCGAGATCCAGATGAGCTCCG
GCAACCTGCTGTGGCCCGGCCATCTGAAATGTAGGCTGAGGATGGATAAGCTGCA
ACTCAAAGGCATGTCCTACTCCATGTGCACCGGAAAGTTCAAGGTGGTGAAAGAAA
TCGCCGAAACACAGCACGGCACCATCGTGATCAGGGTGCAGTATGAGGGCGACG
GCTCCCCCTGTAAGATCCCCTTCGAAATCATGGACCTGGAAAAGAGGCACGTGCT
GGGCAGGCTCATCACCGTGAACCCCATTGTCACAGAGAAAGACTCCCCCGTGAAC
ATCGAGGCCGAGCCTCCCTTTGGCGACTCCTACATCATCATTGGCGTGGAGCCCG
GACAGCTCAAGCTGAACTGGTTCAAGAAGGGCAGCAGCGGCGGCAGCCATCACC
ACCATCATCACCATCAT

Protein
MKWVTFISLLFLFSSAYSMRCIGMSNRDFVEGVSGGSWVDIVLEPGSCVTTMAKNKPT
LDFELIKIEAKQPATLRKYCIEAKLTNTTTESRCPTQGEPSLNEEQDKRFVCKHSMVDR
GWGNGCDLFGKGGIVTCAMFRCKKNMEGKVVQPENLEYTIVITPHSGEEHAVGNDTG
KHGKEIKITPQSSITEAELTGYGTVTMECSPRTGLDFNEMVLLQMENKAWLVHRQWFL
DLPLPWLPGADTQGSNWIQKETLVTFKNPHAKKQDVVVLGSQEGWMHRALTGATEIQ
MSSGNLLWPGHLKCRLRMDKLQLKGMSYSMCTGKFKVVKEIAETQHGTIVIRVQYEG
DGSPCKIPFEIMDLEKRHVLGRLITVNPIVTEKDSPVNIEAEPPFGDSYIIIGVEPGQLKLN
WFKKGSSGGSHHHHHHHH

pD2sE_EV8_SC.12 (I2-I8-U6-P4)

DNA
ATGAAGTGGGTAACCTTTATTTCCCTTCTTTTTCTCTTTAGCTCGGCTTATTCCATGA
GGTGTATCGGCATGTCCAACAGGGACTTTGTGGAGGGAGTGAGCGGCGGCAGCT
GGGTGGACATTGTGCTGGAGCATGGAAAGTGCGTGACCGTGATGATGAAAAACAA
GCCCACCCTGGACTTCGAGCTCATCAAGACAGAGGCTAAACAGCCCGCCACCCTC
AGGAAGTACTGCATCGAGGCCAAGCTGACCAACACAACAACCGAGTCCAGATGCC
CTACACAGGGCGAACCCAGCCTCAACGAAGAGCAGGACAAGAGGTTCGTGTGCAA
ACACAGCATGGTGGACAGGGGCTGGGGCAATGGATGCGACCTCTTCGGAAAAGG
CGGCATCGTGACCTGCGCCATGTTCAGGTGTAAAAAGAACATGGAAGGCAAGGTG
GTGCAGCCCGAAAATCTGGAGTATACCATCGTGATTACCCCCCACAGCGGAGAGG
AGCACGCCGTGGGCAATGACACCGGCAAGCACGGCAAAGAGATTAAGATCACCC
CCCAGTCCTCCATTACCGAAGCTGAACTGACAGGCTACGGCACCGTGACAATGGA
GTGTAGCCCCAGGACCGGACTGGATTTCAACGAGATGGTCCTGCTACAGATGGAG
AACAAGGCCTGGCTCGTGCACAGGCAATGGTTTCTGGATCTGCCTCTGCCTTGGC
TGCCTGGCGCCGATACACAGGGCTCCAACTGGATACAGAAAGAGACCCTCGTCAC
CTTCAAGAATCCCCATGCTAAGAAGCAGGACGTGGTGGTGCTGGGCAGCCAAGAA
GGCTGGATGCACCGGGCCCTGACCGGAGCTACCGAGATCCAGATGAGCTCCGGC
AACCTGCTGTGGCCCGGCCATCTGAAATGTAGGCTGAGGATGGATAAGCTGCAAC
TCAAAGGCATGTCCTACTCCATGTGCACCGGAAAGTTCAAGGTGGTGAAAGAAATC
GCCGAAACACAGCACGGCACCATCGTGATCAGGGTGCAGTATGAGGGCGACGGC
TCCCCCTGTAAGATCCCCTTCGAAATCATGGACCTGGAAAAGAGGCACGTGCTGG
GCAGGCTCATCACCGTGAACCCCATTGTCACAGAGAAAGACTCCCCCGTGAACAT
CGAGGCCGAGCCTCCCTTTGGCGACTCCTACATCATCATTGGCGTGGAGCCCGGA
CAGCTCAAGCTGAACTGGTTCAAGAAGGGCAGCAGCGGCGGCAGCCATCACCAC
CATCATCACCATCAT

Protein
MKWVTFISLLFLFSSAYSMRCIGMSNRDFVEGVSGGSWVDIVLEHGKCVTVMMKNKP
TLDFELIKTEAKQPATLRKYCIEAKLTNTTTESRCPTQGEPSLNEEQDKRFVCKHSMVD
RGWGNGCDLFGKGGIVTCAMFRCKKNMEGKVVQPENLEYTIVITPHSGEEHAVGNDT
GKHGKEIKITPQSSITEAELTGYGTVTMECSPRTGLDFNEMVLLQMENKAWLVHRQWF
LDLPLPWLPGADTQGSNWIQKETLVTFKNPHAKKQDVVVLGSQEGWMHRALTGATEI
QMSSGNLLWPGHLKCRLRMDKLQLKGMSYSMCTGKFKVVKEIAETQHGTIVIRVQYE
GDGSPCKIPFEIMDLEKRHVLGRLITVNPIVTEKDSPVNIEAEPPFGDSYIIIGVEPGQLKL
NWFKKGSSGGSHHHHHHHH

pD2sE_EV8_SC.13 (I2-U5-U6-P4)

DNA
ATGAAGTGGGTAACCTTTATTTCCCTTCTTTTTCTCTTTAGCTCGGCTTATTCCATGA
GGTGTATCGGCATGTCCAACAGGGACTTTGTGGAGGGAGTGAGCGGCGGCAGCT
GGGTGGACATTGTGCTGGAGCATGGAAAGTGCGTGACCGTGATGATGAAAAACAA
GCCCACCCTGGACTTCGAGCTCATCAAGACAGAGGCTAAACAGCCCGCCACCCTC
AGGAAGTACTGCATCGAGGCCAAGCTGACCAACACAACAACCGAGTCCAGATGCC
CTACACAGGGCGAACCCAGCCTCAACGAAGAGCAGGACAAGAGGTTCGTGTGCAA
ACACAGCATGGTGGACAGGGGCTGGGGCAATGGATGCGGACTCTTCGGAAAAGG
CGGCATCGTGACCTGCGCCATGTTCAGGTGTAAAAAGAACATGGAAGGCAAGGTG
GTGCAGCCCGAAAATCTGGAGTATACCATCGTGATTACCCCCCACAGCGGAGAGG
AGCACGCCGTGGGCAATGACACCGGCAAGCACGGCAAAGAGATTAAGATCACCC
CCCAGTCCTCCATTACCGAAGCTGAACTGACAGGCTACGGCACCGTGACAATGGA
GTGTAGCCCCAGGACCGGACTGGATTTCAACGAGATGGTCCTGCTACAGATGGAG
AACAAGGCCTGGCTCGTGCACAGGCAATGGTTTCTGGATCTGCCTCTGCCTTGGC
TGCCTGGCGCCGATACACAGGGCTCCAACTGGATACAGAAAGAGACCCTCGTCAC
CTTCAAGAATCCCCATGCTAAGAAGCAGGACGTGGTGGTGCTGGGCAGCCAAGAA
GGCTGGATGCACCGGGCCCTGACCGGAGCTACCGAGATCCAGATGAGCTCCGGC
AACCTGCTGTGGCCCGGCCATCTGAAATGTAGGCTGAGGATGGATAAGCTGCAAC
TCAAAGGCATGTCCTACTCCATGTGCACCGGAAAGTTCAAGGTGGTGAAAGAAATC
GCCGAAACACAGCACGGCACCATCGTGATCAGGGTGCAGTATGAGGGCGACGGC
TCCCCCTGTAAGATCCCCTTCGAAATCATGGACCTGGAAAAGAGGCACGTGCTGG
GCAGGCTCATCACCGTGAACCCCATTGTCTACGAGAAAGACTCCCCCGTGAACAT
CGAGGCCGAGCCTCCCTTTGGCGACTCCTACATCATCATTGGCGTGGAGCCCGGA
CAGCTCAAGCTGAACTGGTTCAAGAAGGGCAGCAGCGGCGGCAGCCATCACCAC
CATCATCACCATCAT

Protein
MKWVTFISLLFLFSSAYSMRCIGMSNRDFVEGVSGGSWVDIVLEHGKCVTVMMKNKP
TLDFELIKTEAKQPATLRKYCIEAKLTNTTTESRCPTQGEPSLNEEQDKRFVCKHSMVD
RGWGNGCGLFGKGGIVTCAMFRCKKNMEGKVVQPENLEYTIVITPHSGEEHAVGNDT
GKHGKEIKITPQSSITEAELTGYGTVTMECSPRTGLDFNEMVLLQMENKAWLVHRQWF
LDLPLPWLPGADTQGSNWIQKETLVTFKNPHAKKQDVVVLGSQEGWMHRALTGATEI
QMSSGNLLWPGHLKCRLRMDKLQLKGMSYSMCTGKFKVVKEIAETQHGTIVIRVQYE
GDGSPCKIPFEIMDLEKRHVLGRLITVNPIVYEKDSPVNIEAEPPFGDSYIIIGVEPGQLK
LNWFKKGSSGGSHHHHHHHH

pD2sE_EV8_SC.14 (I2-U6)

DNA
ATGAAGTGGGTAACCTTTATTTCCCTTCTTTTTCTCTTTAGCTCGGCTTATTCCATGA
GGTGTATCGGCATGTCCAACAGGGACTTTGTGGAGGGAGTGAGCGGCGGCAGCT
GGGTGGACATTGTGCTGGAGCATGGAAGCTGCGTGACCACGATGGCGAAAAACAA
GCCCACCCTGGACTTCGAGCTCATCAAGACAGAGGCTAAACAGCCCGCCACCCTC
AGGAAGTACTGCATCGAGGCCAAGCTGACCAACACAACAACCGAGTCCAGATGCC
CTACACAGGGCGAACCCAGCCTCAACGAAGAGCAGGACAAGAGGTTCGTGTGCAA
ACACAGCATGGTGGACAGGGGCTGGGGCAATGGATGCGGACTCTTCGGAAAAGG
CGGCATCGTGACCTGCGCCATGTTCAGGTGTAAAAAGAACATGGAAGGCAAGGTG
GTGCAGCCCGAAAATCTGGAGTATACCATCGTGATTACCCCCCACAGCGGAGAGG
AGCACGCCGTGGGCAATGACACCGGCAAGCACGGCAAAGAGATTAAGATCACCC
CCCAGTCCTCCATTACCGAAGCTGAACTGACAGGCTACGGCACCGTGACAATGGA
GTGTAGCCCCAGGACCGGACTGGATTTCAACGAGATGGTCCTGCTACAGATGGAG
AACAAGGCCTGGCTCGTGCACAGGCAATGGTTTCTGGATCTGCCTCTGCCTTGGC
TGCCTGGCGCCGATACACAGGGCTCCAACTGGATACAGAAAGAGACCCTCGTCAC
CTTCAAGAATCCCCATGCTAAGAAGCAGGACGTGGTGGTGCTGGGCAGCCAAGAA
GGCTGGATGCACCGGGCCCTGACCGGAGCTACCGAGATCCAGATGAGCTCCGGC
AACCTGCTGTGGCCCGGCCATCTGAAATGTAGGCTGAGGATGGATAAGCTGCAAC
TCAAAGGCATGTCCTACTCCATGTGCACCGGAAAGTTCAAGGTGGTGAAAGAAATC
GCCGAAACACAGCACGGCACCATCGTGATCAGGGTGCAGTATGAGGGCGACGGC
TCCCCCTGTAAGATCCCCTTCGAAATCATGGACCTGGAAAAGAGGCACGTGCTGG
GCAGGCTCATCACCGTGAACCCCATTGTCACAGAGAAAGACTCCCCCGTGAACAT
CGAGGCCGAGCCTCCCTTTGGCGACTCCTACATCATCATTGGCGTGGAGCCCGGA
CAGCTCAAGCTGAACTGGTTCAAGAAGGGCAGCAGCGGCGGCAGCCATCACCAC
CATCATCACCATCAT

Protein
MKWVTFISLLFLFSSAYSMRCIGMSNRDFVEGVSGGSWVDIVLEHGSCVTTMAKNKPT
LDFELIKTEAKQPATLRKYCIEAKLTNTTTESRCPTQGEPSLNEEQDKRFVCKHSMVDR
GWGNGCGLFGKGGIVTCAMFRCKKNMEGKVVQPENLEYTIVITPHSGEEHAVGNDTG
KHGKEIKITPQSSITEAELTGYGTVTMECSPRTGLDFNEMVLLQMENKAWLVHRQWFL
DLPLPWLPGADTQGSNWIQKETLVTFKNPHAKKQDVVVLGSQEGWMHRALTGATEIQ
MSSGNLLWPGHLKCRLRMDKLQLKGMSYSMCTGKFKVVKEIAETQHGTIVIRVQYEG
DGSPCKIPFEIMDLEKRHVLGRLITVNPIVTEKDSPVNIEAEPPFGDSYIIIGVEPGQLKLN
WFKKGSSGGSHHHHHHHH

pD2sE_EV8_SC.15 (I8-U6)

DNA
ATGAAGTGGGTAACCTTTATTTCCCTTCTTTTTCTCTTTAGCTCGGCTTATTCCATGA
GGTGTATCGGCATGTCCAACAGGGACTTTGTGGAGGGAGTGAGCGGCGGCAGCT
GGGTGGACATTGTGCTGGAGCATGGAAGCTGCGTGACCACGATGGCGAAAAACAA
GCCCACCCTGGACTTCGAGCTCATCAAGACAGAGGCTAAACAGCCCGCCACCCTC
AGGAAGTACTGCATCGAGGCCAAGCTGACCAACACAACAACCGAGTCCAGATGCC
CTACACAGGGCGAACCCAGCCTCAACGAAGAGCAGGACAAGAGGTTCGTGTGCAA
ACACAGCATGGTGGACAGGGGCTGGGGCAATGGATGCGACCTCTTCGGAAAAGG
CGGCATCGTGACCTGCGCCATGTTCAGGTGTAAAAAGAACATGGAAGGCAAGGTG
GTGCAGCCCGAAAATCTGGAGTATACCATCGTGATTACCCCCCACAGCGGAGAGG
AGCACGCCGTGGGCAATGACACCGGCAAGCACGGCAAAGAGATTAAGATCACCC
CCCAGTCCTCCATTACCGAAGCTGAACTGACAGGCTACGGCACCGTGACAATGGA
GTGTAGCCCCAGGACCGGACTGGATTTCAACGAGATGGTCCTGCTACAGATGGAG
AACAAGGCCTGGCTCGTGCACAGGCAATGGTTTCTGGATCTGCCTCTGCCTTGGC
TGCCTGGCGCCGATACACAGGGCTCCAACTGGATACAGAAAGAGACCCTCGTCAC
CTTCAAGAATCCCCATGCTAAGAAGCAGGACGTGGTGGTGCTGGGCAGCCAAGAA
GGCGCCATGCACACAGCCCTGACCGGAGCTACCGAGATCCAGATGAGCTCCGGC
AACCTGCTGTGGCCCGGCCATCTGAAATGTAGGCTGAGGATGGATAAGCTGCAAC
TCAAAGGCATGTCCTACTCCATGTGCACCGGAAAGTTCAAGGTGGTGAAAGAAATC
GCCGAAACACAGCACGGCACCATCGTGATCAGGGTGCAGTATGAGGGCGACGGC
TCCCCCTGTAAGATCCCCTTCGAAATCATGGACCTGGAAAAGAGGCACGTGCTGG
GCAGGCTCATCACCGTGAACCCCATTGTCACAGAGAAAGACTCCCCCGTGAACAT
CGAGGCCGAGCCTCCCTTTGGCGACTCCTACATCATCATTGGCGTGGAGCCCGGA
CAGCTCAAGCTGAACTGGTTCAAGAAGGGCAGCAGCGGCGGCAGCCATCACCAC
CATCATCACCATCAT

Protein
MKWVTFISLLFLFSSAYSMRCIGMSNRDFVEGVSGGSWVDIVLEHGSCVTTMAKNKPT
LDFELIKTEAKQPATLRKYCIEAKLTNTTTESRCPTQGEPSLNEEQDKRFVCKHSMVDR
GWGNGCDLFGKGGIVTCAMFRCKKNMEGKVVQPENLEYTIVITPHSGEEHAVGNDTG
KHGKEIKITPQSSITEAELTGYGTVTMECSPRTGLDFNEMVLLQMENKAWLVHRQWFL
DLPLPWLPGADTQGSNWIQKETLVTFKNPHAKKQDVVVLGSQEGAMHTALTGATEIQ
MSSGNLLWPGHLKCRLRMDKLQLKGMSYSMCTGKFKVVKEIAETQHGTIVIRVQYEG
DGSPCKIPFEIMDLEKRHVLGRLITVNPIVTEKDSPVNIEAEPPFGDSYIIIGVEPGQLKLN
WFKKGSSGGSHHHHHHHH

pD2sE_EV8_SC.16 (I2-U6-H3)

DNA
ATGAAGTGGGTAACCTTTATTTCCCTTCTTTTTCTCTTTAGCTCGGCTTATTCCATGA
GGTGTATCGGCATGTCCAACAGGGACTTTGTGGAGGGAGTGAGCGGCGGCAGCT
GGGTGGACATTGTGCTGGAGCCCGGAAGCTGCGTGACCACGATGGCGAAAAACA
AGCCCACCCTGGACTTCGAGCTCATCAAGATCGAGGCTAAACAGCCCGCCACCCT
CAGGAAGTACTGCATCGAGGCCAAGCTGACCAACACAACAACCGAGTCCAGATGC
CCTACACAGGGCGAACCCAGCCTCAACGAAGAGCAGGACAAGAGGTTCGTGTGC
AAACACAGCATGGTGGACAGGGGCTGGGGCAATGGATGCGGACTCTTCGGAAAA
GGCGGCATCGTGACCTGCGCCATGTTCAGGTGTAAAAAGAACATGGAAGGCAAGG
TGGTGCAGCCCGAAAATCTGGAGTATACCATCGTGATTACCCCCCACAGCGGAGA
GGAGCACGCCGTGGGCAATGACACCGGCAAGCACGGCAAAGAGATTAAGATCAC
CCCCCAGTCCTCCATTACCGAAGCTGAACTGACAGGCTACGGCACCGTGACAATG
GAGTGTAGCCCCAGGACCGGACTGGATTTCAACGAGATGGTCCTGCTACAGATGG
AGAACAAGGCCTGGCTCGTGCACAGGCAATGGTTTCTGGATCTGCCTCTGCCTTG
GCTGCCTGGCGCCGATACACAGGGCTCCAACTGGATACAGAAAGAGACCCTCGTC
ACCTTCAAGAATCCCCATGCTAAGAAGCAGGACGTGGTGGTGCTGGGCAGCCAAG
AAGGCTGGATGCACCGGGCCCTGACCGGAGCTACCGAGATCCAGATGAGCTCCG
GCAACCTGCTGTGGCCCGGCCATCTGAAATGTAGGCTGAGGATGGATAAGCTGCA
ACTCAAAGGCATGTCCTACTCCATGTGCACCGGAAAGTTCAAGGTGGTGAAAGAAA
TCGCCGAAACACAGCACGGCACCATCGTGATCAGGGTGCAGTATGAGGGCGACG
GCTCCCCCTGTAAGATCCCCTTCGAAATCATGGACCTGGAAAAGAGGCACGTGCT
GGGCAGGCTCATCACCGTGAACCCCATTGTCACAGAGAAAGACTCCCCCGTGAAC
ATCGAGGCCGAGCCTCCCTTTGGCGACTCCTACATCATCATTGGCGTGGAGCCCG
GACAGCTCAAGCTGAACTGGTTCAAGAAGGGCAGCAGCGGCGGCAGCCATCACC
ACCATCATCACCATCAT

Protein
MKWVTFISLLFLFSSAYSMRCIGMSNRDFVEGVSGGSWVDIVLEPGSCVTTMAKNKPT
LDFELIKIEAKQPATLRKYCIEAKLTNTTTESRCPTQGEPSLNEEQDKRFVCKHSMVDR
GWGNGCGLFGKGGIVTCAMFRCKKNMEGKVVQPENLEYTIVITPHSGEEHAVGNDTG
KHGKEIKITPQSSITEAELTGYGTVTMECSPRTGLDFNEMVLLQMENKAWLVHRQWFL
DLPLPWLPGADTQGSNWIQKETLVTFKNPHAKKQDVVVLGSQEGWMHRALTGATEIQ
MSSGNLLWPGHLKCRLRMDKLQLKGMSYSMCTGKFKVVKEIAETQHGTIVIRVQYEG
DGSPCKIPFEIMDLEKRHVLGRLITVNPIVTEKDSPVNIEAEPPFGDSYIIIGVEPGQLKLN
WFKKGSSGGSHHHHHHHH

pD2sE_EV8_SC.17 (I2-U6-P4)

DNA
ATGAAGTGGGTAACCTTTATTTCCCTTCTTTTTCTCTTTAGCTCGGCTTATTCCATGA
GGTGTATCGGCATGTCCAACAGGGACTTTGTGGAGGGAGTGAGCGGCGGCAGCT
GGGTGGACATTGTGCTGGAGCATGGAAAGTGCGTGACCGTGATGATGAAAAACAA
GCCCACCCTGGACTTCGAGCTCATCAAGACAGAGGCTAAACAGCCCGCCACCCTC
AGGAAGTACTGCATCGAGGCCAAGCTGACCAACACAACAACCGAGTCCAGATGCC
CTACACAGGGCGAACCCAGCCTCAACGAAGAGCAGGACAAGAGGTTCGTGTGCAA
ACACAGCATGGTGGACAGGGGCTGGGGCAATGGATGCGGACTCTTCGGAAAAGG
CGGCATCGTGACCTGCGCCATGTTCAGGTGTAAAAAGAACATGGAAGGCAAGGTG
GTGCAGCCCGAAAATCTGGAGTATACCATCGTGATTACCCCCCACAGCGGAGAGG
AGCACGCCGTGGGCAATGACACCGGCAAGCACGGCAAAGAGATTAAGATCACCC
CCCAGTCCTCCATTACCGAAGCTGAACTGACAGGCTACGGCACCGTGACAATGGA
GTGTAGCCCCAGGACCGGACTGGATTTCAACGAGATGGTCCTGCTACAGATGGAG
AACAAGGCCTGGCTCGTGCACAGGCAATGGTTTCTGGATCTGCCTCTGCCTTGGC
TGCCTGGCGCCGATACACAGGGCTCCAACTGGATACAGAAAGAGACCCTCGTCAC
CTTCAAGAATCCCCATGCTAAGAAGCAGGACGTGGTGGTGCTGGGCAGCCAAGAA
GGCTGGATGCACCGGGCCCTGACCGGAGCTACCGAGATCCAGATGAGCTCCGGC
AACCTGCTGTGGCCCGGCCATCTGAAATGTAGGCTGAGGATGGATAAGCTGCAAC
TCAAAGGCATGTCCTACTCCATGTGCACCGGAAAGTTCAAGGTGGTGAAAGAAATC
GCCGAAACACAGCACGGCACCATCGTGATCAGGGTGCAGTATGAGGGCGACGGC
TCCCCCTGTAAGATCCCCTTCGAAATCATGGACCTGGAAAAGAGGCACGTGCTGG
GCAGGCTCATCACCGTGAACCCCATTGTCACAGAGAAAGACTCCCCCGTGAACAT
CGAGGCCGAGCCTCCCTTTGGCGACTCCTACATCATCATTGGCGTGGAGCCCGGA
CAGCTCAAGCTGAACTGGTTCAAGAAGGGCAGCAGCGGCGGCAGCCATCACCAC
CATCATCACCATCAT

Protein
MKWVTFISLLFLFSSAYSMRCIGMSNRDFVEGVSGGSWVDIVLEHGKCVTVMMKNKP
TLDFELIKTEAKQPATLRKYCIEAKLTNTTTESRCPTQGEPSLNEEQDKRFVCKHSMVD
RGWGNGCGLFGKGGIVTCAMFRCKKNMEGKVVQPENLEYTIVITPHSGEEHAVGNDT
GKHGKEIKITPQSSITEAELTGYGTVTMECSPRTGLDFNEMVLLQMENKAWLVHRQWF
LDLPLPWLPGADTQGSNWIQKETLVTFKNPHAKKQDVVVLGSQEGWMHRALTGATEI
QMSSGNLLWPGHLKCRLRMDKLQLKGMSYSMCTGKFKVVKEIAETQHGTIVIRVQYE
GDGSPCKIPFEIMDLEKRHVLGRLITVNPIVTEKDSPVNIEAEPPFGDSYIIIGVEPGQLKL
NWFKKGSSGGSHHHHHHHH

pD2sE_EV8_SC.18 (I2-I8-U4-U6-P4)

DNA
ATGAAGTGGGTAACCTTTATTTCCCTTCTTTTTCTCTTTAGCTCGGCTTATTCCATGA
GGTGTATCGGCATGTCCAACAGGGACTTTGTGTTCGCCGTGAGCGGCGGCAGCTG
GGTGGACATTGTGCTGGAGCATGGAAAGTGCGTGACCGTGCTGATGAAAAACAAG
CCCACCCTGGACTTCGAGCTCATCAAGACAGAGGCTAAACAGCCCGCCACCCTCA
GGAAGTACTGCATCGAGGCCAAGCTGACCAACACAACAACCGAGTCCAGATGCCC
TACACAGGGCGAACCCAGCCTCAACGAAGAGCAGGACAAGAGGTTCGTGTGCAAA
CACAGCATGGTGGACAGGGGCTGGGGCAATGGATGCGACCTCTTCGGAAAAGGC
GGCATCGTGACCTGCGCCATGTTCAGGTGTAAAAAGAACATGGAAGGCAAGGTGG
TGCAGCCCGAAAATCTGGAGTATACCATCGTGATTACCCCCCACAGCGGAGAGGA
GCACGCCGTGGGCAATGACACCGGCAAGCACGGCAAAGAGATTAAGATCACCCC
CCAGTCCTCCATTACCGAAGCTGAACTGACAGGCTACGGCACCGTGACAATGGAG
TGTAGCCCCAGGACCGGACTGGATTTCAACGAGATGGTCCTGCTACAGATGGAGA
ACAAGGCCTGGCTCGTGCACAGGCAATGGTTTCTGGATCTGCCTCTGCCTTGGCT
GCCTGGCGCCGATACACAGGGCTCCAACTGGATACAGAAAGAGACCCTCGTCACC
TTCAAGAATCCCCATGCTAAGAAGCAGGACGTGGTGGTGCTGGGCAGCCAAGAAG
GCTGGATGCACCGGGCCCTGACCGGAGCTACCGAGATCCAGATGAGCTCCGGCA
ACCTGCTGTGGCCCGGCCATCTGAAATGTAGGCTGAGGATGGATAAGCTGCAACT
CAAAGGCATGTCCTACTCCATGTGCACCGGAAAGTTCAAGGTGGTGAAAGAAATC
GCCGAAACACAGCACGGCACCATCGTGATCAGGGTGCAGTATGAGGGCGACGGC
TCCCCCTGTAAGATCCCCTTCGAAATCATGGACCTGGAAAAGAGGCACGTGCTGG
GCAGGCTCATCACCGTGAACCCCATTGTCACAGAGAAAGACTCCCCCGTGAACAT
CGAGGCCGAGCCTCCCTTTGGCGACTCCTACATCATCATTGGCGTGGAGCCCGGA
CAGCTCAAGCTGAACTGGTTCAAGAAGGGCAGCAGCGGCGGCAGCCATCACCAC
CATCATCACCATCAT

Protein
MKWVTFISLLFLFSSAYSMRCIGMSNRDFVFAVSGGSWVDIVLEHGKCVTVLMKNKPT
LDFELIKTEAKQPATLRKYCIEAKLTNTTTESRCPTQGEPSLNEEQDKRFVCKHSMVDR
GWGNGCDLFGKGGIVTCAMFRCKKNMEGKVVQPENLEYTIVITPHSGEEHAVGNDTG
KHGKEIKITPQSSITEAELTGYGTVTMECSPRTGLDFNEMVLLQMENKAWLVHRQWFL
DLPLPWLPGADTQGSNWIQKETLVTFKNPHAKKQDVVVLGSQEGWMHRALTGATEIQ
MSSGNLLWPGHLKCRLRMDKLQLKGMSYSMCTGKFKVVKEIAETQHGTIVIRVQYEG
DGSPCKIPFEIMDLEKRHVLGRLITVNPIVTEKDSPVNIEAEPPFGDSYIIIGVEPGQLKLN
WFKKGSSGGSHHHHHHHH

pD2sE_EV8_SC.19 (I8-U5-U6-P4)

DNA
ATGAAGTGGGTAACCTTTATTTCCCTTCTTTTTCTCTTTAGCTCGGCTTATTCCATGA
GGTGTATCGGCATGTCCAACAGGGACTTTGTGGAGGGAGTGAGCGGCGGCAGCT
GGGTGGACATTGTGCTGGAGCATGGAAAGTGCGTGACCGTGATGATGAAAAACAA
GCCCACCCTGGACTTCGAGCTCATCAAGACAGAGGCTAAACAGCCCGCCACCCTC
AGGAAGTACTGCATCGAGGCCAAGCTGACCAACACAACAACCGAGTCCAGATGCC
CTACACAGGGCGAACCCAGCCTCAACGAAGAGCAGGACAAGAGGTTCGTGTGCAA
ACACAGCATGGTGGACAGGGGCTGGGGCAATGGATGCGACCTCTTCGGAAAAGG
CGGCATCGTGACCTGCGCCATGTTCAGGTGTAAAAAGAACATGGAAGGCAAGGTG
GTGCAGCCCGAAAATCTGGAGTATACCATCGTGATTACCCCCCACAGCGGAGAGG
AGCACGCCGTGGGCAATGACACCGGCAAGCACGGCAAAGAGATTAAGATCACCC
CCCAGTCCTCCATTACCGAAGCTGAACTGACAGGCTACGGCACCGTGACAATGGA
GTGTAGCCCCAGGACCGGACTGGATTTCAACGAGATGGTCCTGCTACAGATGGAG
AACAAGGCCTGGCTCGTGCACAGGCAATGGTTTCTGGATCTGCCTCTGCCTTGGC
TGCCTGGCGCCGATACACAGGGCTCCAACTGGATACAGAAAGAGACCCTCGTCAC
CTTCAAGAATCCCCATGCTAAGAAGCAGGACGTGGTGGTGCTGGGCAGCCAAGAA
GGCGCCATGCACACAGCCCTGACCGGAGCTACCGAGATCCAGATGAGCTCCGGC
AACCTGCTGTGGCCCGGCCATCTGAAATGTAGGCTGAGGATGGATAAGCTGCAAC
TCAAAGGCATGTCCTACTCCATGTGCACCGGAAAGTTCAAGGTGGTGAAAGAAATC
GCCGAAACACAGCACGGCACCATCGTGATCAGGGTGCAGTATGAGGGCGACGGC
TCCCCCTGTAAGATCCCCTTCGAAATCATGGACCTGGAAAAGAGGCACGTGCTGG
GCAGGCTCATCACCGTGAACCCCATTGTCTACGAGAAAGACTCCCCCGTGAACAT
CGAGGCCGAGCCTCCCTTTGGCGACTCCTACATCATCATTGGCGTGGAGCCCGGA
CAGCTCAAGCTGAACTGGTTCAAGAAGGGCAGCAGCGGCGGCAGCCATCACCAC
CATCATCACCATCAT

Protein
MKWVTFISLLFLFSSAYSMRCIGMSNRDFVEGVSGGSWVDIVLEHGKCVTVMMKNKP
TLDFELIKTEAKQPATLRKYCIEAKLTNTTTESRCPTQGEPSLNEEQDKRFVCKHSMVD
RGWGNGCDLFGKGGIVTCAMFRCKKNMEGKVVQPENLEYTIVITPHSGEEHAVGNDT
GKHGKEIKITPQSSITEAELTGYGTVTMECSPRTGLDFNEMVLLQMENKAWLVHRQWF
LDLPLPWLPGADTQGSNWIQKETLVTFKNPHAKKQDVVVLGSQEGAMHTALTGATEIQ
MSSGNLLWPGHLKCRLRMDKLQLKGMSYSMCTGKFKVVKEIAETQHGTIVIRVQYEG
DGSPCKIPFEIMDLEKRHVLGRLITVNPIVYEKDSPVNIEAEPPFGDSYIIIGVEPGQLKLN
WFKKGSSGGSHHHHHHHH

pD2sE_EV8_SC.20 (P4-U4)

DNA
ATGAAGTGGGTAACCTTTATTTCCCTTCTTTTTCTCTTTAGCTCGGCTTATTCCATGA
GGTGTATCGGCATGTCCAACAGGGACTTTGTGTTCGCCGTGAGCGGCGGCAGCTG
GGTGGACATTGTGCTGGAGCATGGAAAGTGCGTGACCGTGCTGATGAAAAACAAG
CCCACCCTGGACTTCGAGCTCATCAAGACAGAGGCTAAACAGCCCGCCACCCTCA
GGAAGTACTGCATCGAGGCCAAGCTGACCAACACAACAACCGAGTCCAGATGCCC
TACACAGGGCGAACCCAGCCTCAACGAAGAGCAGGACAAGAGGTTCGTGTGCAAA
CACAGCATGGTGGACAGGGGCTGGGGCAATGGATGCGGACTCTTCGGAAAAGGC
GGCATCGTGACCTGCGCCATGTTCAGGTGTAAAAAGAACATGGAAGGCAAGGTGG
TGCAGCCCGAAAATCTGGAGTATACCATCGTGATTACCCCCCACAGCGGAGAGGA
GCACGCCGTGGGCAATGACACCGGCAAGCACGGCAAAGAGATTAAGATCACCCC
CCAGTCCTCCATTACCGAAGCTGAACTGACAGGCTACGGCACCGTGACAATGGAG
TGTAGCCCCAGGACCGGACTGGATTTCAACGAGATGGTCCTGCTACAGATGGAGA
ACAAGGCCTGGCTCGTGCACAGGCAATGGTTTCTGGATCTGCCTCTGCCTTGGCT
GCCTGGCGCCGATACACAGGGCTCCAACTGGATACAGAAAGAGACCCTCGTCACC
TTCAAGAATCCCCATGCTAAGAAGCAGGACGTGGTGGTGCTGGGCAGCCAAGAAG
GCGCCATGCACACAGCCCTGACCGGAGCTACCGAGATCCAGATGAGCTCCGGCA
ACCTGCTGTTCACCGGCCATCTGAAATGTAGGCTGAGGATGGATAAGCTGCAACT
CAAAGGCATGTCCTACTCCATGTGCACCGGAAAGTTCAAGGTGGTGAAAGAAATC
GCCGAAACACAGCACGGCACCATCGTGATCAGGGTGCAGTATGAGGGCGACGGC
TCCCCCTGTAAGATCCCCTTCGAAATCATGGACCTGGAAAAGAGGCACGTGCTGG
GCAGGCTCATCACCGTGAACCCCATTGTCACAGAGAAAGACTCCCCCGTGAACAT
CGAGGCCGAGCCTCCCTTTGGCGACTCCTACATCATCATTGGCGTGGAGCCCGGA
CAGCTCAAGCTGAACTGGTTCAAGAAGGGCAGCAGCGGCGGCAGCCATCACCAC
CATCATCACCATCAT

Protein
MKWVTFISLLFLFSSAYSMRCIGMSNRDFVFAVSGGSWVDIVLEHGKCVTVLMKNKPT
LDFELIKTEAKQPATLRKYCIEAKLTNTTTESRCPTQGEPSLNEEQDKRFVCKHSMVDR
GWGNGCGLFGKGGIVTCAMFRCKKNMEGKVVQPENLEYTIVITPHSGEEHAVGNDTG
KHGKEIKITPQSSITEAELTGYGTVTMECSPRTGLDFNEMVLLQMENKAWLVHRQWFL
DLPLPWLPGADTQGSNWIQKETLVTFKNPHAKKQDVVVLGSQEGAMHTALTGATEIQ
MSSGNLLFTGHLKCRLRMDKLQLKGMSYSMCTGKFKVVKEIAETQHGTIVIRVQYEGD
GSPCKIPFEIMDLEKRHVLGRLITVNPIVTEKDSPVNIEAEPPFGDSYIIIGVEPGQLKLN
WFKKGSSGGSHHHHHHHH

pD2sE_EV8_SC.21 (U4-U5-U6)

DNA
ATGAAGTGGGTAACCTTTATTTCCCTTCTTTTTCTCTTTAGCTCGGCTTATTCCATGA
GGTGTATCGGCATGTCCAACAGGGACTTTGTGTTCGCCGTGAGCGGCGGCAGCTG
GGTGGACATTGTGCTGGAGCATGGAAGCTGCGTGACCACGCTGGCGAAAAACAA
GCCCACCCTGGACTTCGAGCTCATCAAGACAGAGGCTAAACAGCCCGCCACCCTC
AGGAAGTACTGCATCGAGGCCAAGCTGACCAACACAACAACCGAGTCCAGATGCC
CTACACAGGGCGAACCCAGCCTCAACGAAGAGCAGGACAAGAGGTTCGTGTGCAA
ACACAGCATGGTGGACAGGGGCTGGGGCAATGGATGCGGACTCTTCGGAAAAGG
CGGCATCGTGACCTGCGCCATGTTCAGGTGTAAAAAGAACATGGAAGGCAAGGTG
GTGCAGCCCGAAAATCTGGAGTATACCATCGTGATTACCCCCCACAGCGGAGAGG
AGCACGCCGTGGGCAATGACACCGGCAAGCACGGCAAAGAGATTAAGATCACCC
CCCAGTCCTCCATTACCGAAGCTGAACTGACAGGCTACGGCACCGTGACAATGGA
GTGTAGCCCCAGGACCGGACTGGATTTCAACGAGATGGTCCTGCTACAGATGGAG
AACAAGGCCTGGCTCGTGCACAGGCAATGGTTTCTGGATCTGCCTCTGCCTTGGC
TGCCTGGCGCCGATACACAGGGCTCCAACTGGATACAGAAAGAGACCCTCGTCAC
CTTCAAGAATCCCCATGCTAAGAAGCAGGACGTGGTGGTGCTGGGCAGCCAAGAA
GGCGCCATGCACACAGCCCTGACCGGAGCTACCGAGATCCAGATGAGCTCCGGC
AACCTGCTGTGGCCCGGCCATCTGAAATGTAGGCTGAGGATGGATAAGCTGCAAC
TCAAAGGCATGTCCTACTCCATGTGCACCGGAAAGTTCAAGGTGGTGAAAGAAATC
GCCGAAACACAGCACGGCACCATCGTGATCAGGGTGCAGTATGAGGGCGACGGC
TCCCCCTGTAAGATCCCCTTCGAAATCATGGACCTGGAAAAGAGGCACGTGCTGG
GCAGGCTCATCACCGTGAACCCCATTGTCTACGAGAAAGACTCCCCCGTGAACAT
CGAGGCCGAGCCTCCCTTTGGCGACTCCTACATCATCATTGGCGTGGAGCCCGGA
CAGCTCAAGCTGAACTGGTTCAAGAAGGGCAGCAGCGGCGGCAGCCATCACCAC
CATCATCACCATCATTAGTAA

Protein
MKWVTFISLLFLFSSAYSMRCIGMSNRDFVFAVSGGSWVDIVLEHGSCVTTLAKNKPT
LDFELIKTEAKQPATLRKYCIEAKLTNTTTESRCPTQGEPSLNEEQDKRFVCKHSMVDR
GWGNGCGLFGKGGIVTCAMFRCKKNMEGKVVQPENLEYTIVITPHSGEEHAVGNDTG
KHGKEIKITPQSSITEAELTGYGTVTMECSPRTGLDFNEMVLLQMENKAWLVHRQWFL
DLPLPWLPGADTQGSNWIQKETLVTFKNPHAKKQDVVVLGSQEGAMHTALTGATEIQ
MSSGNLLWPGHLKCRLRMDKLQLKGMSYSMCTGKFKVVKEIAETQHGTIVIRVQYEG
DGSPCKIPFEIMDLEKRHVLGRLITVNPIVYEKDSPVNIEAEPPFGDSYIIIGVEPGQLKLN
WFKKGSSGGSHHHHHHHH

pD2sE_EV8_SC.22 (U4-U5-U6-P5)

DNA
ATGAAGTGGGTAACCTTTATTTCCCTTCTTTTTCTCTTTAGCTCGGCTTATTCCATGA
GGTGTATCGGCATGTCCAACAGGGACTTTGTGTTCGCCGTGAGCGGCGGCAGCTG
GGTGGACATTGTGCTGGAGCATGGAAGCTGCGTGACCACGCTGGCGAAAAACAA
GCCCACCCTGGACTTCGAGCTCATCAAGACAGAGGCTAAACAGCCCGCCACCCTC
AGGAAGTACTGCATCGAGGCCAAGCTGACCAACACAACAACCGAGTCCAGATGCC
CTACACAGGGCGAACCCAGCCTCAACGAAGAGCAGGACAAGAGGTTCGTGTGCAA
ACACAGCATGGTGGACAGGGGCTGGGGCAATGGATGCGGACTCTTCGGAAAAGG
CGGCATCGTGACCTGCGCCATGTTCAGGTGTAAAAAGAACATGGAAGGCAAGGTG
GTGCAGCCCGAAAATCTGGAGTATACCATCGTGATTACCCCCCACAGCGGAGAGG
AGCACGCCGTGGGCAATGACACCGGCAAGCACGGCAAAGAGATTAAGATCACCC
CCCAGTCCTCCATTACCGAAGCTGAACTGACAGGCTACGGCACCGTGACAATGGA
GTGTAGCCCCAGGACCGGACTGGATTTCAACGAGATGGTCCTGCTACAGATGGAG
AACAAGGCCTGGCTCGTGGACAGGCAATGGTTTCTGGATCTGCCTCTGCCTTGGC
TGCCTGGCGCCGATACACAGGGCTCCAACTGGATACAGAAAGAGACCCTCGTCAC
CTTCAAGAATCCCCATGCTAAGAAGCAGGACGTGGTGGTGCTGGGCAGCCAAGAA
GGCGCCATGCACACAGCCCTGACCTGGGCTACCGAGATCCAGATGAGCTCCGGC
AACCTGCTGTGGCCCGGCCATCTGAAATGTAGGCTGAGGATGGATAAGCTGCAAC
TCAAAGGCATGTCCTACTCCATGTGCACCGGAAAGTTCAAGGTGGTGAAAGAAATC
GCCGAAACACAGCACGGCACCATCGTGATCAGGGTGCAGTATGAGGGCGACGGC
TCCCCCTGTAAGATCCCCTTCGAAATCATGGACCTGGAAAAGAGGCACGTGCTGG
GCAGGCTCATCACCGTGAACCCCATTGTCTACGAGAAAGACTCCCCCGTGAACAT
CGAGGCCGAGCCTCCCTTTGGCGACTCCTACATCATCATTGGCGTGGAGCCCGGA
CAGCTCAAGCTGAACTGGTTCAAGAAGGGCAGCAGCGGCGGCAGCCATCACCAC
CATCATCACCATCAT

Protein
MKWVTFISLLFLFSSAYSMRCIGMSNRDFVFAVSGGSWVDIVLEHGSCVTTLAKNKPT
LDFELIKTEAKQPATLRKYCIEAKLTNTTTESRCPTQGEPSLNEEQDKRFVCKHSMVDR
GWGNGCGLFGKGGIVTCAMFRCKKNMEGKVVQPENLEYTIVITPHSGEEHAVGNDTG
KHGKEIKITPQSSITEAELTGYGTVTMECSPRTGLDFNEMVLLQMENKAWLVDRQWFL
DLPLPWLPGADTQGSNWIQKETLVTFKNPHAKKQDVVVLGSQEGAMHTALTWATEIQ
MSSGNLLWPGHLKCRLRMDKLQLKGMSYSMCTGKFKVVKEIAETQHGTIVIRVQYEG
DGSPCKIPFEIMDLEKRHVLGRLITVNPIVYEKDSPVNIEAEPPFGDSYIIIGVEPGQLKLN
WFKKGSSGGSHHHHHHHH

pD2sE_EV8_SC.23 (I2-I8)

DNA
ATGAAGTGGGTAACCTTTATTTCCCTTCTTTTTCTCTTTAGCTCGGCTTATTCCATGA
GGTGTATCGGCATGTCCAACAGGGACTTTGTGGAGGGAGTGAGCGGCGGCAGCT
GGGTGGACATTGTGCTGGAGCATGGAAGCTGCGTGACCACGATGGCGAAAAACAA
GCCCACCCTGGACTTCGAGCTCATCAAGACAGAGGCTAAACAGCCCGCCACCCTC
AGGAAGTACTGCATCGAGGCCAAGCTGACCAACACAACAACCGAGTCCAGATGCC
CTACACAGGGCGAACCCAGCCTCAACGAAGAGCAGGACAAGAGGTTCGTGTGCAA
ACACAGCATGGTGGACAGGGGCTGGGGCAATGGATGCGACCTCTTCGGAAAAGG
CGGCATCGTGACCTGCGCCATGTTCAGGTGTAAAAAGAACATGGAAGGCAAGGTG
GTGCAGCCCGAAAATCTGGAGTATACCATCGTGATTACCCCCCACAGCGGAGAGG
AGCACGCCGTGGGCAATGACACCGGCAAGCACGGCAAAGAGATTAAGATCACCC
CCCAGTCCTCCATTACCGAAGCTGAACTGACAGGCTACGGCACCGTGACAATGGA
GTGTAGCCCCAGGACCGGACTGGATTTCAACGAGATGGTCCTGCTACAGATGGAG
AACAAGGCCTGGCTCGTGCACAGGCAATGGTTTCTGGATCTGCCTCTGCCTTGGC
TGCCTGGCGCCGATACACAGGGCTCCAACTGGATACAGAAAGAGACCCTCGTCAC
CTTCAAGAATCCCCATGCTAAGAAGCAGGACGTGGTGGTGCTGGGCAGCCAAGAA
GGCTGGATGCACCGGGCCCTGACCGGAGCTACCGAGATCCAGATGAGCTCCGGC
AACCTGCTGTTCACCGGCCATCTGAAATGTAGGCTGAGGATGGATAAGCTGCAAC
TCAAAGGCATGTCCTACTCCATGTGCACCGGAAAGTTCAAGGTGGTGAAAGAAATC
GCCGAAACACAGCACGGCACCATCGTGATCAGGGTGCAGTATGAGGGCGACGGC
TCCCCCTGTAAGATCCCCTTCGAAATCATGGACCTGGAAAAGAGGCACGTGCTGG
GCAGGCTCATCACCGTGAACCCCATTGTCACAGAGAAAGACTCCCCCGTGAACAT
CGAGGCCGAGCCTCCCTTTGGCGACTCCTACATCATCATTGGCGTGGAGCCCGGA
CAGCTCAAGCTGAACTGGTTCAAGAAGGGCAGCAGCGGCGGCAGCCATCACCAC
CATCATCACCATCAT

Protein
MKWVTFISLLFLFSSAYSMRCIGMSNRDFVEGVSGGSWVDIVLEHGSCVTTMAKNKPT
LDFELIKTEAKQPATLRKYCIEAKLTNTTTESRCPTQGEPSLNEEQDKRFVCKHSMVDR
GWGNGCDLFGKGGIVTCAMFRCKKNMEGKVVQPENLEYTIVITPHSGEEHAVGNDTG
KHGKEIKITPQSSITEAELTGYGTVTMECSPRTGLDFNEMVLLQMENKAWLVHRQWFL
DLPLPWLPGADTQGSNWIQKETLVTFKNPHAKKQDVVVLGSQEGWMHRALTGATEIQ
MSSGNLLFTGHLKCRLRMDKLQLKGMSYSMCTGKFKVVKEIAETQHGTIVIRVQYEGD
GSPCKIPFEIMDLEKRHVLGRLITVNPIVTEKDSPVNIEAEPPFGDSYIIIGVEPGQLKLN
WFKKGSSGGSHHHHHHHH

pD2sE_EV8_SC.24 (M2-H3)

DNA
ATGAAGTGGGTAACCTTTATTTCCCTTCTTTTTCTCTTTAGCTCGGCTTATTCCATGA
GGTGTATCGGCATGTCCAACAGGGACTTTGTGGAGGGAGTGAGCGGCGGCAGCT
GGGTGGACATTGTGCTGGAGCCCGGAAGCTGCGTGACCACGATGGCGAAAAACA
AGCCCACCCTGGACTTCGAGCTCATCAAGATCGAGGCTAAACAGCCCGCCACCCT
CAGGAAGTACTGCATCGAGGCCAAGCTGACCAACACAACAACCGAGTCCAGATGC
CCTACACAGGGCGAACCCAGCCTCAACGAAGAGCAGGACAAGAGGTTCGTGTGC
AAACACAGCATGGTGGACAGGGGCTGGGGCAATGGATGCGGACTCTTCGGAAAA
GGCGGCATCGTGACCTGCGCCATGTTCAGGTGTAAAAAGAACATGGAAGGCAAGG
TGGTGCAGCCCGAAAATCTGGAGTATACCATCGTGATTACCCCCCACAGCGGAGA
GGAGCACGCCGTGGGCAATGACACCGGCAAGCACGGCAAAGAGATTAAGATCAC
CCCCCAGTCCTCCATTACCGAAGCTGAACTGACAGGCTACGGCACCGTGACAATG
GAGTGTAGCCCCAGGACCGGACTGGATTTCAACGAGATGGTCCTGCTACAGATGG
AGAACAAGGCCTGGCTCGTGCACAGGCAATGGTTTCTGGATCTGCCTCTGCCTTG
GCTGCCTGGCGCCGATACACAGGGCTCCAACTGGATACAGAAAGAGACCCTCGTC
ACCTTCAAGAATCCCCATGCTAAGAAGCAGGACGTGGTGGTGCTGGGCAGCCAAG
AAGAGGCCATGCACACAGCCCTGACCGGAGCTACCGAGATCCAGATGAGCTCCG
GCAACCTGCTGTTCACCGGCCATCTGAAATGTAGGCTGAGGATGGATAAGCTGCA
ACTCAAAGGCATGTCCTACTCCATGTGCACCGGAAAGTTCAAGGTGGTGAAAGAAA
TCGCCGAAACACAGCACGGCACCATCGTGATCAGGGTGCAGTATGAGGGCGACG
GCTCCCCCTGTAAGATCCCCTTCGAAATCATGGACCTGGAAAAGAGGCACGTGCT
GGGCAGGCTCATCACCGTGAACCCCATTGTCACAGAGAAAGACTCCCCCGTGAAC
ATCGAGGCCGAGCCTCCCTTTGGCGACTCCTACATCATCATTGGCGTGGAGCCCG
GACAGCTCAAGCTGAACTGGTTCAAGAAGGGCAGCAGCGGCGGCAGCCATCACC
ACCATCATCACCATCAT

Protein
MKWVTFISLLFLFSSAYSMRCIGMSNRDFVEGVSGGSWVDIVLEPGSCVTTMAKNKPT
LDFELIKIEAKQPATLRKYCIEAKLTNTTTESRCPTQGEPSLNEEQDKRFVCKHSMVDR
GWGNGCGLFGKGGIVTCAMFRCKKNMEGKVVQPENLEYTIVITPHSGEEHAVGNDTG
KHGKEIKITPQSSITEAELTGYGTVTMECSPRTGLDFNEMVLLQMENKAWLVHRQWFL
DLPLPWLPGADTQGSNWIQKETLVTFKNPHAKKQDVVVLGSQEEAMHTALTGATEIQ
MSSGNLLFTGHLKCRLRMDKLQLKGMSYSMCTGKFKVVKEIAETQHGTIVIRVQYEGD
GSPCKIPFEIMDLEKRHVLGRLITVNPIVTEKDSPVNIEAEPPFGDSYIIIGVEPGQLKLN
WFKKGSSGGSHHHHHHHH

pD2sE_EV8_SC.25 (M2-P4)

DNA
ATGAAGTGGGTAACCTTTATTTCCCTTCTTTTTCTCTTTAGCTCGGCTTATTCCATGA
GGTGTATCGGCATGTCCAACAGGGACTTTGTGGAGGGAGTGAGCGGCGGCAGCT
GGGTGGACATTGTGCTGGAGCATGGAAAGTGCGTGACCGTGATGATGAAAAACAA
GCCCACCCTGGACTTCGAGCTCATCAAGACAGAGGCTAAACAGCCCGCCACCCTC
AGGAAGTACTGCATCGAGGCCAAGCTGACCAACACAACAACCGAGTCCAGATGCC
CTACACAGGGCGAACCCAGCCTCAACGAAGAGCAGGACAAGAGGTTCGTGTGCAA
ACACAGCATGGTGGACAGGGGCTGGGGCAATGGATGCGGACTCTTCGGAAAAGG
CGGCATCGTGACCTGCGCCATGTTCAGGTGTAAAAAGAACATGGAAGGCAAGGTG
GTGCAGCCCGAAAATCTGGAGTATACCATCGTGATTACCCCCCACAGCGGAGAGG
AGCACGCCGTGGGCAATGACACCGGCAAGCACGGCAAAGAGATTAAGATCACCC
CCCAGTCCTCCATTACCGAAGCTGAACTGACAGGCTACGGCACCGTGACAATGGA
GTGTAGCCCCAGGACCGGACTGGATTTCAACGAGATGGTCCTGCTACAGATGGAG
AACAAGGCCTGGCTCGTGCACAGGCAATGGTTTCTGGATCTGCCTCTGCCTTGGC
TGCCTGGCGCCGATACACAGGGCTCCAACTGGATACAGAAAGAGACCCTCGTCAC
CTTCAAGAATCCCCATGCTAAGAAGCAGGACGTGGTGGTGCTGGGCAGCCAAGAA
GAGGCCATGCACACAGCCCTGACCGGAGCTACCGAGATCCAGATGAGCTCCGGC
AACCTGCTGTTCACCGGCCATCTGAAATGTAGGCTGAGGATGGATAAGCTGCAAC
TCAAAGGCATGTCCTACTCCATGTGCACCGGAAAGTTCAAGGTGGTGAAAGAAATC
GCCGAAACACAGCACGGCACCATCGTGATCAGGGTGCAGTATGAGGGCGACGGC
TCCCCCTGTAAGATCCCCTTCGAAATCATGGACCTGGAAAAGAGGCACGTGCTGG
GCAGGCTCATCACCGTGAACCCCATTGTCACAGAGAAAGACTCCCCCGTGAACAT
CGAGGCCGAGCCTCCCTTTGGCGACTCCTACATCATCATTGGCGTGGAGCCCGGA
CAGCTCAAGCTGAACTGGTTCAAGAAGGGCAGCAGCGGCGGCAGCCATCACCAC
CATCATCACCATCAT

Protein
MKWVTFISLLFLFSSAYSMRCIGMSNRDFVEGVSGGSWVDIVLEHGKCVTVMMKNKP
TLDFELIKTEAKQPATLRKYCIEAKLTNTTTESRCPTQGEPSLNEEQDKRFVCKHSMVD
RGWGNGCGLFGKGGIVTCAMFRCKKNMEGKVVQPENLEYTIVITPHSGEEHAVGNDT
GKHGKEIKITPQSSITEAELTGYGTVTMECSPRTGLDFNEMVLLQMENKAWLVHRQWF
LDLPLPWLPGADTQGSNWIQKETLVTFKNPHAKKQDVVVLGSQEEAMHTALTGATEIQ
MSSGNLLFTGHLKCRLRMDKLQLKGMSYSMCTGKFKVVKEIAETQHGTIVIRVQYEGD
GSPCKIPFEIMDLEKRHVLGRLITVNPIVTEKDSPVNIEAEPPFGDSYIIIGVEPGQLKLN
WFKKGSSGGSHHHHHHHH

pD2sE_EV8_SC.26 (I3-I8-U6)

DNA
ATGAAGTGGGTAACCTTTATTTCCCTTCTTTTTCTCTTTAGCTCGGCTTATTCCATGA
GGTGTATCGGCATGTCCAACAGGGACTTTGTGGAGGGAGTGAGCGGCGGCAGCT
GGGTGGACATTGTGCTGGAGCATGGAAGCTGCGTGACCACGATGGCGAAAAACAA
GCCCACCCTGGACTTCGAGCTCATCAAGACAGAGGCTAAACAGCCCGCCACCCTC
AGGAAGTACTGCATCGAGGCCAAGCTGACCAACACAACAACCGAGTCCAGATGCC
CTACACAGGGCGAACCCAGCCTCAACGAAGAGCAGGACAAGAGGTTCGTGTGCAA
ACACAGCATGGTGGACAGGGGCTGGGGCAATGGATGCGACCTCTTCGGAAAAGG
CGGCATCGTGACCTGCGCCATGTTCAGGTGTAAAAAGAACATGGAAGGCAAGGTG
GTGCAGCCCGAAAATCTGGAGTATACCATCGTGATTACCCCCCACAGCGGAGAGG
AGCACGCCGTGGGCAATGACACCGGCAAGCACGGCAAAGAGATTAAGATCACCC
CCCAGTCCTCCATTACCGAAGCTGAACTGACAGGCTACGGCACCGTGACAATGGA
GTGTAGCCCCAGGACCGGACTGGATTTCAACGAGATGGTCCTGCTACAGATGGAG
AACAAGGCCTGGCTCGTGCACAGGCAATGGTTTCTGGATCTGCCTCTGCCTTGGC
TGCCTGGCGCCGATACACAGGGCTCCAACTGGATACAGAAAGAGACCCTCGTCAC
CTTCAAGAATCCCCATGCTAAGAAGCAGGACGTGGTGGTGCTGGGCAGCCAAGAA
GGCGTGATGCACCGGTGGCTGACCGGAGCTACCGAGATCCAGATGAGCTCCGGC
AACCTGCTGTGGCCCGGCCATCTGAAATGTAGGCTGAGGATGGATAAGCTGCAAC
TCAAAGGCATGTCCTACTCCATGTGCACCGGAAAGTTCAAGGTGGTGAAAGAAATC
GCCGAAACACAGCACGGCACCATCGTGATCAGGGTGCAGTATGAGGGCGACGGC
TCCCCCTGTAAGATCCCCTTCGAAATCATGGACCTGGAAAAGAGGCACGTGCTGG
GCAGGCTCATCACCGTGAACCCCATTGTCACAGAGAAAGACTCCCCCGTGAACAT
CGAGGCCGAGCCTCCCTTTGGCGACTCCTACATCATCATTGGCGTGGAGCCCGGA
CAGCTCAAGCTGAACTGGTTCAAGAAGGGCAGCAGCGGCGGCAGCCATCACCAC
CATCATCACCATCAT

Protein
MKWVTFISLLFLFSSAYSMRCIGMSNRDFVEGVSGGSWVDIVLEHGSCVTTMAKNKPT
LDFELIKTEAKQPATLRKYCIEAKLTNTTTESRCPTQGEPSLNEEQDKRFVCKHSMVDR
GWGNGCDLFGKGGIVTCAMFRCKKNMEGKVVQPENLEYTIVITPHSGEEHAVGNDTG
KHGKEIKITPQSSITEAELTGYGTVTMECSPRTGLDFNEMVLLQMENKAWLVHRQWFL
DLPLPWLPGADTQGSNWIQKETLVTFKNPHAKKQDVVVLGSQEGVMHRWLTGATEIQ
MSSGNLLWPGHLKCRLRMDKLQLKGMSYSMCTGKFKVVKEIAETQHGTIVIRVQYEG
DGSPCKIPFEIMDLEKRHVLGRLITVNPIVTEKDSPVNIEAEPPFGDSYIIIGVEPGQLKLN
WFKKGSSGGSHHHHHHHH

pD2sE_EV8_SC.27 (I3-I8-U6-P4)

DNA
ATGAAGTGGGTAACCTTTATTTCCCTTCTTTTTCTCTTTAGCTCGGCTTATTCCATGA
GGTGTATCGGCATGTCCAACAGGGACTTTGTGGAGGGAGTGAGCGGCGGCAGCT
GGGTGGACATTGTGCTGGAGCATGGAAAGTGCGTGACCGTGATGATGAAAAACAA
GCCCACCCTGGACTTCGAGCTCATCAAGACAGAGGCTAAACAGCCCGCCACCCTC
AGGAAGTACTGCATCGAGGCCAAGCTGACCAACACAACAACCGAGTCCAGATGCC
CTACACAGGGCGAACCCAGCCTCAACGAAGAGCAGGACAAGAGGTTCGTGTGCAA
ACACAGCATGGTGGACAGGGGCTGGGGCAATGGATGCGACCTCTTCGGAAAAGG
CGGCATCGTGACCTGCGCCATGTTCAGGTGTAAAAAGAACATGGAAGGCAAGGTG
GTGCAGCCCGAAAATCTGGAGTATACCATCGTGATTACCCCCCACAGCGGAGAGG
AGCACGCCGTGGGCAATGACACCGGCAAGCACGGCAAAGAGATTAAGATCACCC
CCCAGTCCTCCATTACCGAAGCTGAACTGACAGGCTACGGCACCGTGACAATGGA
GTGTAGCCCCAGGACCGGACTGGATTTCAACGAGATGGTCCTGCTACAGATGGAG
AACAAGGCCTGGCTCGTGCACAGGCAATGGTTTCTGGATCTGCCTCTGCCTTGGC
TGCCTGGCGCCGATACACAGGGCTCCAACTGGATACAGAAAGAGACCCTCGTCAC
CTTCAAGAATCCCCATGCTAAGAAGCAGGACGTGGTGGTGCTGGGCAGCCAAGAA
GGCGTGATGCACCGGTGGCTGACCGGAGCTACCGAGATCCAGATGAGCTCCGGC
AACCTGCTGTGGCCCGGCCATCTGAAATGTAGGCTGAGGATGGATAAGCTGCAAC
TCAAAGGCATGTCCTACTCCATGTGCACCGGAAAGTTCAAGGTGGTGAAAGAAATC
GCCGAAACACAGCACGGCACCATCGTGATCAGGGTGCAGTATGAGGGCGACGGC
TCCCCCTGTAAGATCCCCTTCGAAATCATGGACCTGGAAAAGAGGCACGTGCTGG
GCAGGCTCATCACCGTGAACCCCATTGTCACAGAGAAAGACTCCCCCGTGAACAT
CGAGGCCGAGCCTCCCTTTGGCGACTCCTACATCATCATTGGCGTGGAGCCCGGA
CAGCTCAAGCTGAACTGGTTCAAGAAGGGCAGCAGCGGCGGCAGCCATCACCAC
CATCATCACCATCAT

Protein
MKWVTFISLLFLFSSAYSMRCIGMSNRDFVEGVSGGSWVDIVLEHGKCVTVMMKNKP
TLDFELIKTEAKQPATLRKYCIEAKLTNTTTESRCPTQGEPSLNEEQDKRFVCKHSMVD
RGWGNGCDLFGKGGIVTCAMFRCKKNMEGKVVQPENLEYTIVITPHSGEEHAVGNDT
GKHGKEIKITPQSSITEAELTGYGTVTMECSPRTGLDFNEMVLLQMENKAWLVHRQWF
LDLPLPWLPGADTQGSNWIQKETLVTFKNPHAKKQDVVVLGSQEGVMHRWLTGATEI
QMSSGNLLWPGHLKCRLRMDKLQLKGMSYSMCTGKFKVVKEIAETQHGTIVIRVQYE
GDGSPCKIPFEIMDLEKRHVLGRLITVNPIVTEKDSPVNIEAEPPFGDSYIIIGVEPGQLKL
NWFKKGSSGGSHHHHHHHH

pD2sE_EV8_SC.28 (P4-Cm2)

DNA
ATGAAGTGGGTAACCTTTATTTCCCTTCTTTTTCTCTTTAGCTCGGCTTATTCCATGA
GGTGTATCGGCATGTCCAACAGGGACTTTGTGGAGGGAGTGAGCGGCGGCAGCT
GGGTGGACATTGTGCTGGAGCATGGAAAGTGCGTGACCGTGATGATGAAAAACAA
GCCCACCCTGGACTTCGAGCTCATCAAGACAGAGGCTAAACAGCCCGCCACCCTC
AGGAAGTACTGCATCGAGGCCAAGCTGACCAACACAACAACCGAGTCCAGATGCC
CTACACAGGGCGAACCCAGCCTCAACGAAGAGCAGGACAAGAGGTTCGTGTGCAA
ACACAGCATGGTGGACAGGGGCTGGGGCAATGGATGCGGATGTTTCGGAAAAGG
CGGCATCGTGACCTGCGCCATGTTCAGGTGTAAAAAGAACATGGAAGGCAAGGTG
GTGCAGCCCGAAAATCTGGAGTATACCATCGTGATTACCCCCCACAGCGGAGAGG
AGCACGCCGTGGGCAATGACACCGGCAAGCACGGCAAAGAGATTAAGATCACCC
CCCAGTCCTCCATTACCGAAGCTGAACTGACAGGCTACGGCACCGTGACAATGGA
GTGTAGCCCCAGGACCGGACTGGATTTCAACGAGATGGTCCTGCTACAGATGGAG
AACAAGGCCTGGCTCGTGCACAGGCAATGGTTTCTGGATCTGCCTCTGCCTTGGC
TGCCTGGCGCCGATACACAGGGCTCCAACTGGATACAGAAAGAGACCCTCGTCAC
CTTCAAGAATCCCCATGCTAAGAAGCAGGACGTGGTGGTGCTGGGCAGCCAAGAA
GGCGCCATGCACACAGCCCTGACCGGAGCTACCGAGATCCAGATGAGCTCCGGC
AACCTGCTGTTCACCGGCCATCTGAAATGTAGGCTGAGGATGGATAAGCTGCAAC
TCAAAGGCATGTCCTACTCCATGTGCACCGGAAAGTTCAAGGTGGTGAAAGAAATC
TGTGAAACACAGCACGGCACCATCGTGATCAGGGTGCAGTATGAGGGCGACGGC
TCCCCCTGTAAGATCCCCTTCGAAATCATGGACCTGGAAAAGAGGCACGTGCTGG
GCAGGCTCATCACCGTGAACCCCATTGTCACAGAGAAAGACTCCCCCGTGAACAT
CGAGGCCGAGCCTCCCTTTGGCGACTCCTACATCATCATTGGCGTGGAGCCCGGA
CAGCTCAAGCTGAACTGGTTCAAGAAGGGCAGCAGCGGCGGCAGCCATCACCAC
CATCATCACCATCAT

Protein
MKWVTFISLLFLFSSAYSMRCIGMSNRDFVEGVSGGSWVDIVLEHGKCVTVMMKNKP
TLDFELIKTEAKQPATLRKYCIEAKLTNTTTESRCPTQGEPSLNEEQDKRFVCKHSMVD
RGWGNGCGCFGKGGIVTCAMFRCKKNMEGKVVQPENLEYTIVITPHSGEEHAVGNDT
GKHGKEIKITPQSSITEAELTGYGTVTMECSPRTGLDFNEMVLLQMENKAWLVHRQWF
LDLPLPWLPGADTQGSNWIQKETLVTFKNPHAKKQDVVVLGSQEGAMHTALTGATEIQ
MSSGNLLFTGHLKCRLRMDKLQLKGMSYSMCTGKFKVVKEICETQHGTIVIRVQYEGD
GSPCKIPFEIMDLEKRHVLGRLITVNPIVTEKDSPVNIEAEPPFGDSYIIIGVEPGQLKLN
WFKKGSSGGSHHHHHHHH
